# Studying dimensions of representation: introducing the Belgian RepResent panel (2019–2021)

**DOI:** 10.1057/s41304-023-00430-z

**Published:** 2023-05-30

**Authors:** Elie Michel, Fernando Feitosa, Jonas Lefevere, Jean-Benoît Pilet, Patrick van Erkel, Emilie van Haute

**Affiliations:** 1 Université Libre de Bruxelles, Brussels, Belgium; 2 Vrije Universiteit Brussel, Brussels, Belgium; 3 University of Amsterdam, Amsterdam, The Netherlands

**Keywords:** Belgium, Elections, Political representation, Panel study, Dataset, Multi-party system

## Abstract

This research note presents the RepResent Belgian Panel (RBP). The RBP is a voter panel survey consisting of four waves fielded to a sample of voters in Belgium around the May 2019 federal, regional, and European elections in Belgium. It provides unique data on about 250 variables for a quota sample of the same respondents, pre-2019 elections (*N* = 7351), post-2019 elections (*N* = 3909), one year after the elections (*N* = 1996), and 2 years after the elections (*N* = 1119). The RBP panel dataset was designed to analyse voters’ political attitudes and behaviours, notably on different dimensions of democratic representation, and with a specific focus on democratic resentment (e.g. citizens’ attitudes towards democracy such as distrust and alienation, but also behaviours such as abstention, protest, or voting for anti-establishment parties). Its longitudinal structure allows to explore the political dynamics at play in Belgium throughout the lengthy government formation process. Finally, the last two waves of the RBP were fielded during the Covid-19 pandemic, allowing to explore public opinion before and during this global crisis. The RBP should be of interest to scholars of public opinion and electoral studies.

**Supplementary Information:**

The online version contains supplementary material available at 10.1057/s41304-023-00430-z.

## Introduction

The RBP is a four-wave voter panel survey fielded between 2019 and 2021. It provides original information on Belgian citizens before and after the May 2019 federal, regional, and European elections (which occurred on the same day). The RBP panel dataset was designed to analyse citizens’ political attitudes and behaviours, with a specific focus on the different dimensions of democratic representation (substantive, procedural, and symbolic) and on democratic resentment, which we conceive as attitudes (e.g. alienation, distrust) and behaviours (e.g. protest, abstention). Additionally, its longitudinal structure allows to explore the political dynamics at play in Belgium throughout the election cycle, but also during the lengthy government formation process, as well as after the breaktrough of the Covid-19 pandemic. The RBP data is publicly available in Open Access and documented and should be of interest to scholars of public opinion and electoral studies (Walgrave et al. 2022).

The RBP was conducted by the Excellence of Science consortium RepResent (Representation & Democratic Resentment), which is a collaboration of political scientists from five Belgian universities (KULeuven, UCLouvain, Université libre de Bruxelles, Universiteit Antwerpen, Vrije Universiteit Brussel). It was coordinated by Stefaan Walgrave (Universiteit Antwerpen). The other principal investigators and co-supervisors were Sofie Marien (KULeuven), Benoit Rihoux and Virginie Van Ingelgom (UCLouvain), Emilie van Haute and Jean-Benoit Pilet (Université libre de Bruxelles), and Karen Celis and Kris Deschouwer (Vrije Universiteit Brussel).

The RBP can be combined with other election studies fielded previously in the country (Deschouwer et al. 2010, 2015). In particular, the teams in charge of the RepResent project have already conducted longitudinal surveys during 2009 Regional and the 2014 Federal, Regional and European elections, under the projects PartiRep I (2007–2011, coordinated by Kris Deschouwer, VUB; PIs: Marc Hooghe, KULeuven, Pascal Delwit, ULB, Stefaan Walgrave, UA) and PartiRep II (2012–2017, coordinated by Kris Deschouwer, VUB; PIs: Marc Hooghe, KULeuven, Pascal Delwit, ULB, Stefaan Walgrave, UA, benoit Rihoux, UCLouvain) funded by the federal Belgian science policy, Belspo. These datasets are also fully documented and available in open access (Deschouwer et al. 2009, 2014).

Before presenting the RBP dataset, we first discuss the context of the Belgian political system. We then describe the RBP study design, its technical validation, and the main areas of investigation of the survey. Finally, we discuss potential applications of this dataset and suggest likely comparisons with existing data.

## The Belgian political system

Belgium is a parliamentary democracy and a federal system. It elects its members of parliament, at national and regional level, under a list proportional representation (PR) system with multiple multi-member districts.^1^ The party system is extremely fragmented, with an effective number of parties of 9.7 in the Chamber of Representatives after the 2019 federal elections (Delwit and van Haute 2021). Since the split of traditional party families along the French-Dutch linguistic divide, Belgium is characterized by two party systems operating separately: Dutch-speaking parties compete in Flanders, whereas French-speaking parties compete in Wallonia (Deschouwer 2012; Deschouwer et al. 2017). Consequently, Dutch- and French- speaking parties do not present a list of candidates in the same electoral district, except in the bilingual district of Brussels.

Historically, the Dutch-speaking party system leans to the right, while the French-speaking party system is more to the left (van Haute and Deschouwer 2018). Because of the extreme party system fragmentation and of the differences between the Dutch-and French-speaking party systems, forming a coalition at the federal level is very complex and requires a lot of time: the longest period to form a federal government was 541 days in 2010. It also necessitates an agreement between many parties: the federal government put in place after the 2019 elections comprises of 7 parties. Further, Belgian elections are peculiar since voting is compulsory. However, although not showing up at the polling station on the day of the elections may result in a fine, such sanctions have not been applied over the last fifteen years.

The 2019 elections that are covered by the RBP dataset, confirm the elements that we have just mentioned. The federal, regional, and European elections were held simultaneously on May 26th. First, turnout is high, and stable over time, because of compulsory voting (88.4%). Second, the party systems remained very fragmented, and twelve parties elected at least one MP in the lower house of the federal parliament. Both largest parties nationwide are located in Flanders: the Dutch-speaking Conservative Regionalists N-VA (New Flemish Alliance, *Nieuw-Vlaamse Alliantie,* 16% of all valid votes) and the radical right party VB (Flemish Interest, *Vlaams Belang,* 12% of all valid votes). In French-speaking Belgium, the largest party was the left-wing party PS (Socialist Party, *Parti socialiste*) (Table 1). Electoral results in the regional and European elections very closely match this distribution and regional divide. Finally, forming new coalition governments was extremely complex, especially at the federal level. A first agreement was achieved to put in place a temporary cabinet to manage the first wave of COVID-19. It was only in October 2020, more than one year after the 2019 elections, that a new coalition government with full prerogatives in all policy domains was formed by seven parties (Dutch- and French- speaking Liberals, Socialists, and Greens, and the Dutch-speaking Christian Democrats).

**Table 1:** Results of 2019 Belgian Federal Elections and government participation

Party	Party family	% Vote Regional Flanders	% Vote Regional Wallonia	% Vote Regional Brussels-Capital	% Vote Federal	N Seats Federal	Federal government participation
*Dutch-speaking party system*							
Nieuw-Vlaamse Alliantie (N-VA)	Regionalist	24.8		18.0	16.0	25	
Vlaams Belang (VB)	Regionalist/Radical Right	18.5		8.3	12.0	18	
Christen-Democratisch en Vlaams (CD&V)	Christian-Democratic	15.4		7.5	8.9	12	✓
Parti du Travail de Belgique/Partij van de Arbeid van België (PTB/PvdA)*	Radical Left*	5.3		4.3	8.6*	12*	
Open Vlaamse Liberalen en Democraten (Open Vld)	Liberal	13.1		15.8	8.5	12	✓
Socialistische Partij Anders (sp.a)	Social-Democratic	10.1		15.1	6.7	9	✓
Groen	Green	10.1		20.6	6.1	8	✓
*French-speaking party system*							
Parti Socialiste (PS)	Social-Democratic		26.2	22.0	9.5	20	✓
Parti du Travail de Belgique/Partij van de Arbeid van België (PTB/PvdA)*	Radical Left*		13.7	13.5	8.6*	12*	
Mouvement Réformateur (MR)	Conservative		21.4	16.9	7.6	14	✓
Ecolo	Green		14.5	19.1	6.1	13	✓
Centre Démocrate Humaniste (CDH)	Christian-Democratic		11.0	7.6	3.7	5	
Démocrate, Fédéraliste, Indépendant (DéFI)	Liberal		4.1	13.8	2.2	2	

The table only displays the scores of parties who obtained a seat in the lower Chamber

*The PTB/PvdA is the only party that presented lists and obtained seats in the two party systems of the country

*Source*: https://elections2019.belgium.be/fr

## Study design and technical validation

**Fig. 1:**
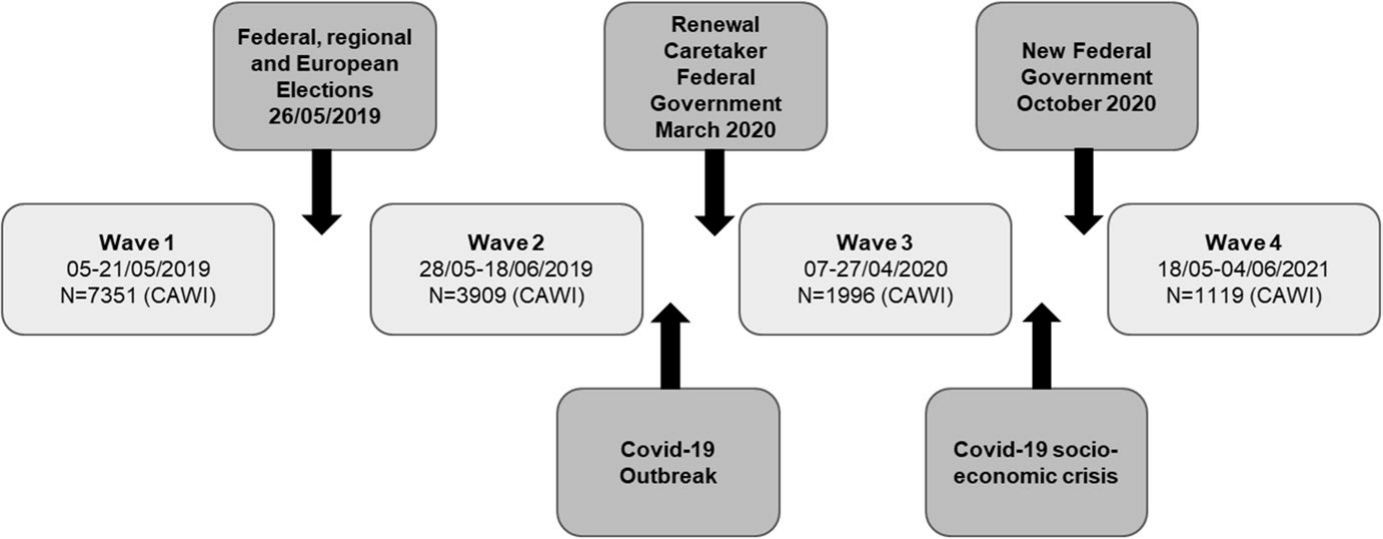
Timeline of RBP panel waves

The RBP surveyed individual voters over four waves of questionnaires fielded around the federal, regional, and European elections of May 26, 2019, and prolonged until June 2021 (Fig. 1). The RPB questionnaires were administered through CAWI (Computer Assisted Web Interview) by Kantar TNS.^2^ The sample consisted of respondents that were recruited from several online panels (Kantar’s own online panel as well as panels from other online companies such as Dynata), to allow for the largest possible recruitment of participants for the first panel wave. The target population of the study was the inhabitants of Flanders, Wallonia, and the Brussels Region that were eligible to vote for the elections of May 26, 2019. The target sample was a quota sample that would match the distribution of the population based on gender, age, and education levels in the three regions.

The four waves surveyed the same initial sample of respondents, who were repeatedly contacted with the request to participate in the survey. In the first, pre-electoral wave, respondents were questioned between April 5 and May 21, 2019 (99% were interviewed before May 6). The second, post-electoral wave surveyed the same respondents immediately after the elections (between May 28 and June 18, 2019). Respondents were surveyed a third time one year after the elections (between April 7 and April 27, 2020), and a fourth time two years after the elections (between May 18 and June 4, 2021).^3^

The unique, longitudinal character of the RBP dataset (4 waves, over 2 years) allows to further explore dynamics over time. Especially, waves 3 and 4 allow tracking the evolution of political attitudes and behaviours of Belgian citizens between elections, when governments are formed (elections + 1y) and take new policy decisions (elections + 2y), but also over the course of a unique health crisis due to the COVID-19 pandemic. Indeed, the outgoing (minority) federal government was managing the current affairs (caretaker government), and the negotiations to form a government were still ongoing when the pandemic occurred (May 2019-March 2020). An emergency caretaker government (Wilmès I) was formed in March 2020 with the support of opposition parties, with the specific task to handle the pandemic, while the negotiations to form a more stable government were occuring in parallel. It is only on October 1st, 2020 that the De Croo government was put in place (Fig. 1).

Table 2 describes the number of respondents for each wave after removing those who completed the survey in five minutes or less, who gave nonsensical responses (e.g., "fjkdqmfjdk") to open-ended questions, or straight-lined some of the matrix-style questions. Table 2 also discloses the attrition rates across the four RBP panel waves. 7351 interviews were completed in the first wave of the RPB, to maximize the responses over time and keep sizeable samples in the following panel waves. Despite the relatively important respondent attrition that can be expected in long-term panels, wave 4, that was fielded more than 2 years after wave 1, still includes 1119 respondents. Note that, due to attrition, Brussels respondents were left out of the sample after wave 2.

**Table 2:** RBP panel attrition

	Number of respondents				% Panel from preceding wave			% Panel from wave 1		
	Wave 1	Wave 2	Wave 3	Wave 4	Wave 2	Wave 3	Wave 4	Wave 2	Wave 3	Wave 4
Flanders	3.298	1.971	1.266	721	59.8	64.2	57.0	59.8	38.4	21.9
Wallonia	3.025	1.429	730	398	47.2	51.1	54.5	47.2	24.1	13.2
Brussels	1.028	509		–	49.5	–	–	49.5	–	–
Total	7.351	3.909	1.996	1.119	53.2	51.1	56.1	53.2	27.2	15.2

Due to non-responses—a problem for most contemporary surveys (De Heer and De Leeuw 2002)—and panel attrition, the final sample slightly differs from the target population. Like several other individual-level surveys (e.g., see Jackman and Spahn 2019), the initial raw sample is slightly distorted, with younger and lower educated (particularly women) individuals underrepresented. This distortion is relatively stable between the first two waves of the RBP. However, the distortion increases in the last two waves, since underrepresented individuals at the beginning of the survey are also more likely to drop out of panel surveys (see Gidron et al. 2022, for a similar dynamic regarding younger respondents). Given these differences, the RPB data includes weightings based on the distribution of the population (post-stratification). The weights were computed through iterative proportional sampling (raking) using the *ipfraking* module in STATA (Kolenikov 2017). This ensures that the weights correct the marginal distributions of the sample in order to match the population distribution of each region on gender and education (crossed, six categories), age (four categories), and voting behaviour for the federal elections in 2019.^4^ Weighting targets and coefficients are presented in Appendix A. The weighted samples of the RBP provide unique representative data over the four waves. The questions of the RBP panel survey were designed to study the substantive, procedural, and symbolic dimensions of political representation (Pitkin 1967). Furthermore, the study focused on democratic resentment (e.g. citizens’ attitudes towards democracy such as distrust and alienation, but also behaviours such as abstention, protest, or voting for anti-establishment parties). To evaluate substantive representation, the questionnaire includes a large array of standard political and policy preferences e.g. socio-cultural or socio-economic issues). Indeed, the congruence between policy and citizens’ preferences is central to the quality of democracy (Diamond and Morlino 2005). To evaluate procedural representation, the RBP questionnaire includes several variables on the workings of the political system and its potential reforms (e.g., more direct, participatory, or deliberative democratic procedures). To evaluate symbolic representation, the questionnaire includes a battery of questions on “feeling” represented and on the linkage between individuals’ concerns and the concerns of institutions, political parties, and political leaders. Finally, the questionnaire conceives democratic resentment not only by using traditional indicators of political behaviour (abstention, protest, vote choice for populist or anti-establishment parties), but also by means of wider indicators on emotions, distrust, and alienation towards democracy and politics.

Furthermore, the RBP questionnaire includes two additional sets of questions. First, it includes questions on the use of Voting Advice Applications (VAA) during the campaign, on the appreciation of the results, and on the positions on the 18 VAA statements. In our sample, about 41% of Flemish respondents declared that they had used a VAA, for only 30% in Wallonia (Brussels respondents did not have a dedicated VAA available). The RBP dataset therefore allows to test the congruence between respondents’ and parties’ preferences, but also the electoral impact of using VAAs (Talukder et al. 2021).

Second, the last two waves of the RBP were fielded respectively right after the outbreak of the Covid-19 pandemic (April 2020), and during the ensuing social and economic crisis (May 2021). An additional module on the pandemic was included, covering attitudes and emotions during the pandemic, agreement with Covid-19 governmental measures (and possible future measures), and the role of experts during the pandemic. The questionnaire includes more the 250 variables. Table 3 provides an overview of some of the main variables included and indicates how the variables are measured, as well as their availability in the various panel waves (see codebook in online Appendix for the full list of variables and question wording).

**Table 3:** Overview of the main variables included in RBP

Main variables	Answer categories	Panel wave availability
*Attitudes and behaviours*		
Left–Right	0–10	1,2,3,4
Interest in politics	0–10	1,2,3,4
Vote 2014 Federal Elections	Party List	1
Vote 2019 Federal Elections	Party List	1,2,3,4
Vote 2019 Regional Elections	Party List	1,2,3,4
Vote 2019 European Elections	Party List	1,2,3,4
Likelihood of Voting for Party (Party List)	0–10	2,3,4
Conventional and Unconventional Political Participation (9 questions)	1–4	1
Political Knowledge Institutions	4 options	1
Political Knowledge Leader Parliament	4 options	1
Political Knowledge EU	4 options	1
Political Knowledge Regional minister	4 options	1
Political Knowledge EU Parliament	4 options	1
Political Knowledge EU Issue	4 options	1
Satisfaction with Government Institutions (5 questions)	0–10	1
Evaluation of Average Satisfaction with Government Institutions (5 questions)	0–10	1
*Substantive representation*		
Political Efficacy (8 questions)	1–5	1,2,3,4
Social Values (5 questions)	0–10	1
Issue Salience (most important issue)	0–2	1,2,3,4
Perceived Positions of Political Parties (VAA statements—18 questions)	1–4	1,2,3,4
Feeling about Political Groups (9 questions)	0–100	1,2
Agreement with Expected Policy Outcome (5 questions)	0–10	1
*Symbolic representation*		
Feeling Represented (3 questions)	0–10	1,2,3,4
Satisfaction with Democracy	1–5	1,2,3,4
Estimation of Average Satisfaction with Democracy	0–100	1,2
Composition of Social Network	List	1
Satisfaction with Democracy of Social Network	1–5	1
*Procedural Representation*		
Evaluation Democratic Procedures (9 questions)	1–5	1,2,3,4
Support Democratic Reforms (9 questions)	1–5	1,2,3,4
Evaluation of Competence of Citizens and Experts (6 questions)	0–10	1,2,3,4
Decisions of Citizens vs. Politicians (6 questions)	0–10	1,2,3,4
Support for Consultative Referendum	0–100	1,2,3,4
Support Binding Referendum	0–100	1,2,3,4
Support Citizens' Forum	0–100	1,2,3,4
Support Participatory Budgeting	0–100	1,2,3,4
Support Experts' Role in Politics (6 questions)	0–10	1,2,3
Issue Positions (VAA statements—18 questions)	1–4	1,2,3,4
*Democratic resentment*		
Political Emotions (9 questions)	0–10	1,2,4
Trust in Political Institutions (4 questions)	0–10	1,3,4
Political Cynicism (7 questions)	1–5	1,2,3,4
Political Populism (7 questions)	1–5	1,2,3,4
Party Voted for: winner or loser (3 questions)	1–2	2,3,4
Party Voted for: winner or loser (after government formation)	1–2	4
Left–Right Positions of Parties	0–10	3
Satisfaction with Election Outcome	0–10	2,3

## Potential applications

The RBP panel provides two examples of potential uses, and namely the study of the evolution of the electoral behaviour over time, by merging this dataset with previous studies, and the comparison of citizens’ attitudes before and after the pandemic.

### Evolution in abstention potential: Belgian electoral panels over time (2009/2014/2019–2021)

The RBP can be combined with other voter panel studies fielded previously in the country. In particular, the teams in charge of the RBP project have already conducted longitudinal surveys during the 2009 regional and the 2014 federal, regional, and European elections. These datasets are also fully documented and available in open access (Deschouwer et al. 2009, 2014). Thus comparing datasets from RepResent I (2007–2011) and RepResent II (2012–2017) projects allow offers great potential to study the evolution of voting behaviours in Belgium over time. Over time, the datasets include standard variables of public opinion and political behaviour, as well as a (limited) number of shared variable on democratic attitudes that allow for longitudinal comparison. Indeed, the Belgian political system presents a series of features that are very relevant for country experts and comparative scholars alike, such as insights on vote choice or electoral volatility in extremely fragmented party systems, or on the electoral accountability of coalition governments (van Erkel et al. 2020; Pilet et al. 2020).

A further potential application relates to compulsory voting. Combining the RBP with the previous PartiRep I and PartiRep II datasets, we can examine the abstention potential in Belgium over the last decade (Hooghe and Deschouwer 2010; Reuchamps et al. 2015—the a question asks voters whether they would still vote if compulsory voting was abolished in Belgium^5^), or, alternatively, the causes and consequences of being a “relunctant voter” (Dassonneville et al. 2019).

**Fig. 2:**
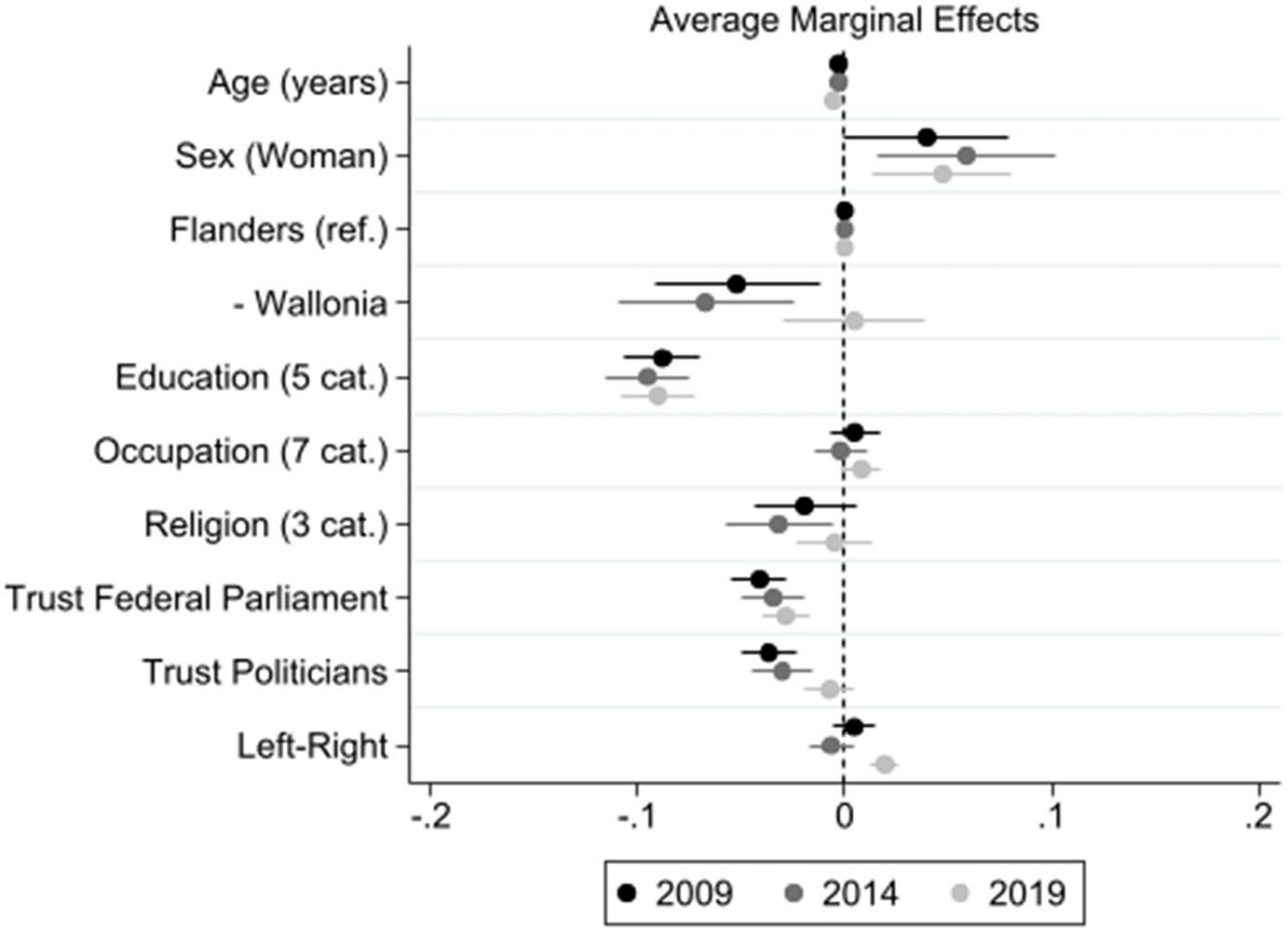
Explaining the abstention potential over time (2009–2019)

When it comes to the Belgians’ abstention potential, we can examine how answers to this question have evolved over time, and what factors influence the willingness to abstain among Belgian voters, i.e., choosing to abstain if it was legally allowed (Frognier et al. 2012). Figure 2 shows average marginal effects of major socio-demographic variables, trust, and ideology on abstention potential (see Appendix B for full model results). The association of individual-level factors with abstention potential is very stable over time: being older, male, with a higher level of education, being a student, or displaying higher levels of trust in parliament, are all negatively associated to abstention potential, to a similar extent in 2009, 2014, and 2021. However, in the most recent election of 2019, Wallonians tend to have a higher abstention potential. Interestingly, the negative effect of trusting politicians or parliament evolved over time on abstention potential is decreasing over time. These initial findings suggest that the influence of structural factors on vote choice in Belgium have evolved over time and call for further research.

### Panel surveys in the times of the Covid-19 pandemic

Because of its panel design over two years, RPB has high potential for research combined with other panel studies for comparative research. One of the potential applications is to measure the political consequences of the Covid-19 crisis, as the panel started more than a year before the outbreak of the pandemic and lasted throughout 2021. This design is particularly useful, as it allows to measure variables of interest for the study of public opinion and political behaviour in times of a sanitary crisis, while measuring potential correlates (partisanship, ideology, issue preferences) at an earlier time point, pre-pandemic (to avoid endogeneity issues associated with measuring political behaviours and attitudes, or partisanship and vote choices simultaneously, see Wlezien et al. 1997, Bartels 2002). It allows to investigate the effect of political factors on evaluations or reactions to Covid-19, such as the rally around the flag effect (Baekgaard et al. 2020; Dietz et al. 2021; Kritzinger et al. 2021; Schraff 2020). Furthermore, the RPB allows for cross-country comparison with similar Covid-19 panels (in Austria, see Kittel et al. 2020, 2022; in 11 democracies, see Brouard et al. 2022). In the RBP dataset, we observe a striking stability of political attitudes throughout the Covid-19 pandemic, which is in line with the findings in other Western countries (Altiparmakis et al. 2021).

Table 4 reports the evolution of a satisfaction with democracy and a typical socio-economic policy preference (minimum pension amount) across the four waves of the panel. Both aggregate levels of satisfaction and support remain unchanged after the outbreak of the pandemic. The stability of attitudinal indicators on all dimensions of democratic representation and on most policy preferences in times of global crisis and democratic limitations, contributes to the debate on the robustness and resilience of how citizens view and evaluate democracy (Ferrin and Kriesi 2016).

**Table 4:** Evolution of attitudes in RBP

		May 2019	June 2019	April 2020	May 2021
Satisfaction with democracy	Satisfied (%)	27.7	24.2	25.5	26.9
	Neither satisfied nor dissatisfied (%)	32.3	29.9	31.3	30.4
	Dissatisfied (%)	40	45.9	43.2	42.7
A retirement pension of at least €1500 per month should be introduced	Disagree (%)	15.5	16.7	17.9	16.7
	Agree (%)	84.5	83.3	82.1	83.3

### Electronic supplementary material

Below is the link to the electronic supplementary material.

Supplementary file1 (PDF 1811 KB)

## Appendix A: Weighting targets–Population targets are identified based on the data of Kantar TNS

**Table:**

Wave 1									
Total	Flanders (*N* = 3293)			Wallonia (*N* = 3023)			Brussels (*N* = 1024)		
	Unweighted basis (%)	Population target (%)	Weighting coefficient	Unweighted basis (%)	Population target (%)	Weighting coefficient	Unweighted basis (%)	Population target (%)	Weighting coefficient
	100.00	100.00		100.00	100.00		100.00	100.00	
**Gender and education**									
*Female*									
Lower educated	5.10	13.60	2.67	6.70	16.90	2.52	4.90	12.40	2.53
Middle educated	16.70	21.00	1.26	18.60	20.20	1.09	14.60	18.70	1.28
Higher educated	22.50	15.90	0.71	26.00	14.00	0.54	30.60	20.00	0.65
*Male*									
Lower educated	7.70	13.40	1.74	6.50	16.20	2.49	5.20	11.90	2.29
Middle educated	23.10	20.60	0.89	18.30	19.30	1.05	13.00	17.90	1.38
Higher educated	24.80	15.50	0.63	24.00	13.40	0.56	31.80	19.20	0.60
**Age**									
18–29	16.10	17.70	1.10	16.50	19.30	1.17	22.10	23.50	1.06
30–44	21.20	24.20	1.14	26.00	24.70	0.95	26.40	33.00	1.25
45–65	36.70	34.50	0.94	39.00	34.20	0.88	32.10	31.20	0.97
65 +	26.00	23.60	0.91	18.50	21.80	1.18	19.40	12.30	0.63

Wave 2									
Total	Flanders (N = 1969)			Wallonia (*N* = 1428)			Brussels (*N* = 506)		
	Unweighted basis (%)	Population target (%)	Weighting coefficient	Unweighted basis (%)	Population target (%)	Weighting coefficient	Unweighted basis (%)	Population target (%)	Weighting coefficient
	100.00	100.00		100.00	100.00		100.00	100.00	
**Gender and education**									
*Female*									
Lower educated	4.40	13.60	3.09	6.40	16.90	2.64	4.90	12.40	2.53
Middle educated	15.70	21.00	1.34	18.40	20.20	1.10	11.50	18.70	1.63
Higher educated	21.70	15.90	0.73	26.90	14.00	0.52	28.30	20.00	0.71
*Male*									
Lower educated	7.80	13.40	1.72	6.20	16.20	2.61	5.10	11.90	2.33
Middle educated	24.40	20.60	0.84	18.10	19.30	1.07	15.40	17.90	1.16
Higher educated	26.00	15.50	0.60	24.10	13.40	0.56	34.80	19.20	0.55
**Age**									
18–29	11.60	17.70	1.53	14.80	19.30	1.30	16.20	23.50	1.45
30–44	20.60	24.20	1.17	28.70	24.70	0.86	23.70	33.00	1.39
45–65	40.40	34.50	0.85	46.80	34.20	0.73	39.10	31.20	0.80
65 +	27.40	23.60	0.86	9.70	21.80	2.25	21.00	12.30	0.59

Wave 3						
Total	Flanders (*N* = 1266)			Wallonia (*N* = 730)		
	Unweighted basis (%)	Population target (%)	Weighting coefficient	Unweighted basis (%)	Population target (%)	Weighting coefficient
	100.00	100.00		100.00	100.00	
**Gender and education**						
*Female*						
Lower educated	4.50	13.60	3.02	6.30	16.90	2.68
Middle educated	15.40	21.00	1.36	18.40	20.20	1.10
Higher educated	19.30	15.90	0.82	23.20	14.00	0.60
*Male*						
Lower educated	7.00	13.40	1.91	6.60	16.20	2.45
Middle educated	25.90	20.60	0.80	18.90	19.30	1.02
Higher educated	28.00	15.50	0.55	26.70	13.40	0.50
**Age**						
18–29	7.90	17.70	2.24	11.40	19.30	1.69
30–44	19.60	24.20	1.23	25.60	24.70	0.96
45–65	43.80	34.50	0.79	50.30	34.20	0.68
65 +	28.70	23.60	0.82	12.70	21.80	1.72

Wave 4						
Total	Flanders (*N* = 721)			Wallonia (*N* = 398)		
	Unweighted basis (%)	Population target (%)	Weighting coefficient	Unweighted basis (%)	Population target (%)	Weighting coefficient
	100.00	100.00		100.00	100.00	
**Gender and education**						
*Female*						
Lower educated	5.00	13.60	2.72	5.50	16.90	3.07
Middle educated	13.70	21.00	1.53	18.10	20.20	1.12
Higher educated	17.60	15.90	0.90	18.60	14.00	0.75
*Male*						
Lower educated	7.80	13.40	1.72	5.80	16.20	2.79
Middle educated	27.30	20.60	0.75	21.60	19.30	0.89
Higher educated	28.60	15.50	0.54	30.40	13.40	0.44
**Age**						
18–29	5.40	17.70	3.28	8.30	19.30	2.33
30–44	17.90	24.20	1.35	22.40	24.70	1.10
45–65	44.10	34.50	0.78	54.00	34.20	0.63
65 +	32.60	23.60	0.72	15.30	21.80	1.42

## Appendix B: Explaining potential abstention in Belgium over time (2009–2019)

**Table:**

	2009	2014	2019
Age	−0.02^***^	−0.03^***^	−0.02***
	(−4.10)	(−5.38)	(−6.04)
Sex (= woman)	0.20^**^	0.30^***^	0.22***
	(2.07)	(2.98)	(2.85)
Flanders	0.00	0.00	0.00
	(.)	(.)	(.)
Wallonia	−0.26^***^	−0.31^***^	−0.01
	(−2.73)	(−3.09)	(−0.12)
Education (5 cat.)	−0.38^***^	−0.40^***^	−0.39^***^
	(−7.78)	(−7.83)	(−9.66)
Occupation (ref. Independent)			
Independent	0.00	0.00	0.00
	(.)	(.)	(.)
Employee	−0.36^*^	0.01	−0.27
	(−1.90)	(0.06)	(−1.29)
Worker	−0.01	0.30	0.11
	(−0.03)	(1.31)	(0.45)
Pensioner	−0.04	0.54^**^	−0.25
	(−0.18)	(2.28)	(−1.13)
Unemployed	0.44	0.33	0.56**
	(1.60)	(1.20)	(2.13)
Student	−0.81^***^	−0.75^***^	−0.68**
	(−2.91)	(−2.70)	(−2.47)
Other Inactive	0.11	0.30	0.09
	(0.48)	(1.19)	(0.42)
Religion (ref. Catholic)			
Catholic	0.00	0.00	0.00
	(.)	(.)	(.)
Other Religion	−0.13	−0.21	0.12
	(−0.94)	(−1.60)	(0.97)
No conviction	−0.18	−0.26^**^	−0.04
	(−1.51)	(−2.13)	(−0.53)
Trust Federal Parliament (0–10)	−0.18^***^	−0.14^***^	−0.12***
	(−5.47)	(−4.06)	(−4.58)
Trust Politicians (0–10)	−0.17^***^	−0.14^***^	−0.03
	(−5.64)	(−4.14)	(−1.22)
Left–Right (Left 0–10 Right)	0.02	−0.03	0.09***
	(0.98)	(−1.08)	(5.39)
Constant	3.80^***^	3.55^***^	2.27***
	(8.84)	(8.01)	(5.92)
Observations	2204	1935	3243
Aic	2771.16	2468.80	4113.49
Bic	2862.33	2557.88	4210.84

## Footnotes

1 Simultaneously to the federal and European elections, the three constitutive regions (Brussels, Flanders, Wallonia) of Belgium elected their parliaments. On the same day, the German-speaking minority elected the members of the Parliament of the German-speaking Minority, yet these voters are not included in the RBP.

2 Because of the length and layout of the questionnaires, it was decided not to allow respondents to complete the survey on a mobile device (phone, tablet, etc.). Mobile users, therefore, received a message in which they were requested to re-enter the survey via a desktop or a laptop. The median duration of the online interviews was 22 min in wave 1, 19 min in wave 2, 21 min in wave 3, and 15 min in wave 4.

3 At the start of each wave an extensive explanation was given to respondents about the privacy rules and regulations concerning the processing of personal data. In addition, respondents were informed that they could be contacted again for a future study. Only respondents that gave consent to continue with the survey, and to be contacted again in the future, were invited to proceed further. The data collection was conducted in line with the General Data Protection Regulation (GDPR) and received ethical approval from the Ethics committee for the Social Sciences and Humanities of the University of Antwerp (EA SHW_18_23).

4 Note that the weights exclude respondents that switched region across the various waves, as it is unclear to which region’s target distribution these respondents should then be counted. The impact of this exclusion is minor however: only 0.8% of the wave 2 sample changed between waves 1 and 2; 0.3% of the wave 3 sample changed between waves 1 and 3; 0.3% of the wave 4 sample changed between waves 1 and 4. The smaller sample size of the Brussels sample, combined with the large number of choice options for vote choice in the 2019 elections, would have resulted in outsized weighting coefficients. So, we decided against calculating the wave 2 weight for vote choice for respondents in that region. Also, especially for waves 3 and 4, the weighting coefficients are substantial, and thus should be used with great care.

5 The question we examine here is worded “If voting was no longer mandatory in Belgium, would you still vote for the following types of elections?”. It was recoded into a binary variable where “always going to vote” is coded 0, and any options of potential abstention (rarely/sometimes/always) is coded 1.
