# Supplementary material for: Studying dimensions of representation: introducing the Belgian RepResent panel (2019–2021)
Source: Eur Polit Sci. 2023 May 30:1–19. Online ahead of print. doi: 10.1057/s41304-023-00430-z (PMC10228433; doi:10.1057/s41304-023-00430-z)
Supplement: Supplementary file 1 — Supplementary file1 (PDF 1811 KB) [file 41304_2023_430_MOESM1_ESM.pdf]

# RepResent Voter Panel Survey 2019 – Wave 1, 2, 3 & 4

Codebook

Table of content

1. The RepResent consortium 3
2. Methodology 3
  2.1 Sample 3
    Sample size wave 1 4
    Sample size wave 2 4
    Sample size wave 3 4
    Sample size wave 4 4
  2.2 Method of data collection 5
3. Data processing – weights 5
  3.1 Representativeness 5
    Wave 1 6
    Wave 2 6
    Wave 3 7
    Wave 4 7
  3.2 Weights 7
4. Codebook questionnaire 9
  4.1 Wave 1 questionnaire 9
  4.2 Wave 2 questionnaire 54
  4.3 Wave 3 questionnaire 104
  4.4 Wave 4 questionnaire 145

1. The RepResent consortium

RepResent (Representation & Democratic Resentment) is an Excellence of Science (FWO/FNRS) funded consortium of political scientists from five Belgian universities. It is coordinated by Stefaan Walgrave, Jonas Lefevere and Patrick van Erkel (UA). The other principal investigators are Sofie Marien (KULeuven), Karen Celis and Kris Deschouwer (VUB), Virginie van Ingelgom and Benoît Rihoux (UCLouvain), Emilie Van Haute and Jean-Benoit Pilet (ULB).

The aim of RepResent is to empirically examine the relationship between the functioning of representation and popular democratic resentment. Three types of representation are distinguished: (1) substantive representation, (2) procedural representation, (3) symbolic representation. RepResent is novel in the sense that it systematically compares citizens' views with elites' views, that it tackles all three dimensions of representation at the same time to assess their individual contribution to democratic resentment, and that it does so in a dynamic over-time design. Concretely, it examines the 2019 elections in Belgium, the campaign that precedes it, and the term that follows. Using a large variety of methods all with a dynamic component and ranging from traditional panel surveys, over content analyses and experiments, to focus groups and interviews, RepResent aims to dig deep into one of the root causes of the widespread democratic resentment characterizing current politics.

This codebook describes the method and questionnaires of one specific data collection within the larger framework of RepResent, namely the *voter panel survey*, which consists of four waves of questionnaires around the elections of May 26 2019. The four waves surveyed the same initial sample of respondents, who were repeatedly contacted with the request to participate in the survey.

2. Methodology

2.1 Sample

The *voter panel study* consists of a pre- and post-electoral wave around the elections of May 26, 2019, a third wave about one year later (April 2020), and a final fourth wave. In the first, pre-electoral wave respondents were questioned between April 5 and May 21, 2019 (99% was interviewed before May 6). The second, post-electoral wave, surveyed the *same* respondents immediately after the elections (between May 28 and June 18, 2019). Respondents were surveyed a third time one year after the elections (between April 7 and April 27, 2020), and a final fourth time two years after the elections (between May 18, 2021 and June 4, 2021). These surveys were conducted by Kantar TNS at the request of the Excellence of Science consortium RepResent.

The target population of the study are the inhabitants of Flanders, Wallonia, and the Brussels Region that were eligible to vote for the elections of May 26, 2019. The gross sample consisted of respondents that were recruited from diverse online panels (Kantar's own panel as well as panels from other online companies such as Dynata). The target was a net sample that would match the distribution on gender, age and education for the voting aged population in their respective regions. Due to non-response, the final samples differ from the target population distributions somewhat (see section 3).

The initial target was to have a net sample of 2500 respondents at the end of the second wave (1000 respondents in Flanders and Wallonia and 500 respondents in the Brussels Region). During the fieldwork, however, it was decided to oversample and continue the data collection even when the

target was reached to be able to conduct a third and potentially fourth wave during the legislative term. For the third and fourth waves no target was set: the aim was simply to maximize responses.

Sample size wave 1

After concluding the fieldwork of wave 1 **7351** interviews were realized. Because the external panels do not report the number of emails sent out, it is impossible to calculate a response rate. Yet, such response rates are not comparable with traditional probability sampling designs anyhow, since respondents from maintained panels do not form a random sample of citizens.

Per region:
- Flanders: n=3298 interviews
- Wallonia: n=3025 interviews
- Brussels: n=1028 interviews

Sample size wave 2

Contacting the 7351 respondents from wave 1 ultimately led to **3909** completed interviews after the elections, which corresponds to a response rate of 53.2%.[1]

Per region:
- Flanders: n=1971 interviews = 59.8% response rate
- Wallonia: n=1429 interviews = 47.2% response rate
- Brussels: n=509 interviews = 49.5% response rate

Sample size wave 3

For the third wave, all 3406 respondents from Flanders and Wallonia that participated in both wave 1 and wave 2 were recontacted. This resulted in **1996** completed responses, which corresponds to a response rate of 58,6% compared to the second wave.[2] For the third wave we did not recontact respondents from Brussels, as this group was too small.

Per region:
- Flanders: n=1266 interviews = 64.2% response rate compared to the second wave
- Wallonia: n=730 interviews = 51.1% response rate compared to the second wave

Sample size wave 4

Finally, for wave four the 1996 Flemish and Walloon respondents that participated in wave 3 were contacted for a final questionnaire. In total, **1119 interviews were completed.**

Per region:
- Flanders: n=721 interviews = 60.0% response rate compared to the third wave

---

[1] Note that although 3917 respondents completed wave 2, we only retain respondents that completely finished both W1 and W2 in the sample, dropping 8 responses.

[2] Again, respondents were matched between the three waves via a unique ID code. 38 respondents were omitted from the dataset in wave 3 because their age did not match with the previous waves (i.e. they were younger or more than 2 years older). Moreover, four Flemish respondents indicated to have moved to the region of Brussels between the second and third wave. As wave 3 only dealt with Flanders and Wallonia, these four respondents' answers for wave 3 were removed from the dataset (their answers for wave 1 and 2 were retained).

- Wallonia: n=398 interviews = 54.5% response rate compared to the third wave

2.2 Method of data collection

In both waves, respondents were interviewed online. To this end, CAWI questionnaires were programmed by the consortium on the Qualtrics platform. Respondents were invited to participate in the study via email by Kantar TNS. Several reminders were sent by Kantar TNS to increase the response.

At the start of *each wave* an extensive explanation was given to the respondents about the privacy rules and regulations concerning the processing of personal data. In addition, respondents were informed that they could be contacted again for a future study. Only respondents that gave consent to continue with the questionnaire *and* to be contacted again in the future proceeded with the surveys. The data collection was conducted in line with the General Data Protection Regulation (GDPR) and received ethical approval from the Ethics Committee for the Social Sciences and Humanities (EA SHW) of the University of Antwerp.

The CAWI questionnaires could only be accessed via a computer or laptop. Because of the length and lay-out of the questionnaires we decided not to allow respondents to complete the survey on a mobile device (phone, tablet, …). Mobile users therefore received a message in which they were requested to re-enter the questionnaire via a computer or laptop.

The median duration of the online interviews was **22 minutes** in wave 1, **19 minutes** in wave 2, **21 minutes** in wave 3, and **15 minutes** in wave 4.

3. Data processing – weights

3.1 Representativeness

As a sample is not a perfect reflection of the population from which the sample is drawn, the RepResent team checked for the representativeness of the sample compared to the population of reference in two ways: the representativeness on age, gender and education and on voting behavior for the federal elections in 2019.

The tables below compare the distribution of age, gender & education (crossed, six categories) and voting behavior (wave 2 only) of the Flemish and Walloon samples with the population of reference in the different waves. As is clear, there are substantial differences between the sample distributions and the population, in particular on education (underrepresentation of lower educated men and women) and age (underrepresentation of age group 18 to 29 years old). The discrepancy increased in later waves due to panel attrition. In wave 2's vote choice, we see that especially the group 'Did not go out to vote' is underrepresented. Note that we omit respondents that indicated 'Other' for either education or gender from the tables, this slightly reduces the number of respondents compared to the sample sizes reported above.

Wave 1

Variable	Flanders (N=3293)		Wallonia (N=3023)		Brussels (N=1024)	
**Gender and education**	**Population**	**Survey**	**Population**	**Survey**	**Population**	**Survey**
Female						
- Lower educated	13.6%	5.1%	16.9%	6.7%	12.4%	4.9%
- Middle educated	21.0%	16.7%	20.2%	18.6%	18.7%	14.6%
- Higher educated	15.9%	22.5%	14.0%	26.0%	20.0%	30.6%
Male						
- Lower educated	13.4%	7.7%	16.2%	6.5%	11.9%	5.2%
- Middle educated	20.6%	23.1%	19.3%	18.3%	17.9%	13.0%
- Higher educated	15.5%	24.8%	13.4%	24.0%	19.2%	31.8%
**Age**						
- 18-29	17.7%	16.1%	19.3%	16.5%	23.5%	22.1%
- 30-44	24.2%	21.2%	24.7%	26.0%	33.0%	26.4%
- 45-65	34.5%	36.7%	34.2%	39.0%	31.2%	32.1%
- 65+	23.6%	26.0%	21.8%	18.5%	12.3%	19.4%

Wave 2

Variable	Flanders (N=1969)		Wallonia (N=1428)		Brussels (N=506)	
**Gender and education**	**Population**	**Survey**	**Population**	**Survey**	**Population**	**Survey**
Female						
- Lower educated	13.6%	4.4%	16.9%	6.4%	12.4%	4.9%
- Middle educated	21.0%	15.7%	20.2%	18.4%	18.7%	11.5%
- Higher educated	15.9%	21.7%	14.0%	26.9%	20.0%	28.3%
Male						
- Lower educated	13.4%	7.8%	16.2%	6.2%	11.9%	5.1%
- Middle educated	20.6%	24.4%	19.3%	18.1%	17.9%	15.4%
- Higher educated	15.5%	26.0%	13.4%	24.1%	19.2%	34.8%
**Age**						
- 18-29	17.7%	11.6%	19.3%	14.8%	23.5%	16.2%
- 30-44	24.2%	20.6%	24.7%	28.7%	33.0%	23.7%
- 45-65	34.5%	40.4%	34.2%	46.8%	31.2%	39.1%
- 65+	23.6%	27.4%	21.8%	9.7%	12.3%	21.0%

Flanders (N=1969)			Wallonia (N=1428)		
**Vote choice**	**Population**	**Survey**	**Vote choice**	**Population**	**Survey**
- CD&V	12,1%	10,2%	- cdH	8,4%	6,3%
- Groen	8,4%	8,7%	- DéFI	3,2%	6,0%
- N-VA	21,8%	26,2%	- Ecolo	11,7%	15,4%
- Open VLD	11,5%	8,7%	- MR	16,2%	17,3%
- PVDA	4,8%	6,8%	- PP	2,5%	4,1%
- Sp.a	9,3%	10,5%	- PS	20,6%	19,7%
- Vlaams Belang	15,9%	20,0%	- PTB	10,9%	13,3%
- Other party	1,7%	1,4%	- Other party	5,3%	3,2%
- Blanc	4,2%	2,5%	- Blanc	7,4%	5,7%
- Did not vote	10,3%	2,9%	- Did not vote	13,7%	4,2%
- Not allowed to vote	0,0%	0,4%	- Not allowed to vote	0,0%	0,6%
- Don’t know	0,0%	1,8%	- Don’t know	0,0%	4,1%

Wave 3

Variable	Flanders (N=1266)		Wallonia (N=730)	
**Gender and education**	**Population**	**Survey**	**Population**	**Survey**
Female				
- Lower educated	13.6%	4.5%	16.9%	6.3%
- Middle educated	21.0%	15.4%	20.2%	18.4%
- Higher educated	15.9%	19.3%	14.0%	23.2%
Male				
- Lower educated	13.4%	7.0%	16.2%	6.6%
- Middle educated	20.6%	25.9%	19.3%	18.9%
- Higher educated	15.5%	28.0%	13.4%	26.7%
**Age**				
- 18-29	17.7%	7.9%	19.3%	11.4%
- 30-44	24.2%	19.6%	24.7%	25.6%
- 45-65	34.5%	43.8%	34.2%	50.3%
- 65+	23.6%	28.7%	21.8%	12.7%

Wave 4

Variable	Flanders (N=721)		Wallonia (N=398)	
**Gender and education**	**Population**	**Survey**	**Population**	**Survey**
Female				
- Lower educated	13.6%	5.0%	16.9%	5.5%
- Middle educated	21.0%	13.7%	20.2%	18.1%
- Higher educated	15.9%	17.6%	14.0%	18.6%
Male				
- Lower educated	13.4%	7.8%	16.2%	5.8%
- Middle educated	20.6%	27.3%	19.3%	21.6%
- Higher educated	15.5%	28.6%	13.4%	30.4%
**Age**				
- 18-29	17.7%	5.4%	19.3%	8.3%
- 30-44	24.2%	17.9%	24.7%	22.4%
- 45-65	34.5%	44.1%	34.2%	54.0%
- 65+	23.6%	32.6%	21.8%	15.3%

3.2 Weights

Considering the differences between the distribution of the sample and the distribution in the population, the RepResent team has calculated weightings (post stratification). The method used is based on the known distribution of the population. The population distribution for Flanders and Wallonia were provided by Kantar TNS. The weights were computed through iterative proportional sampling (raking) using the ipfraking module in STATA. This ensures that the weights correct the marginal distributions of the sample as to match the population distribution of each region on gender and education (crossed, six categories), age (four categories), and voting behavior for the federal elections in 2019.

Note that the weights exclude respondents that switched region across the various waves, as it is unclear to which region's target distribution these respondents should then be counted. The impact

of this exclusion is minor however: only 0.8% of the wave 2 sample changed between wave 1 and 2; 0.3% of the wave 3 sample changed between wave 1 and 3; 0.3% of the wave 4 sample changed between wave 1 and 4.

The smaller sample size of the Brussels sample, combined with the large number of choice options for vote choice in the 2019 elections, would have resulted in outsized weighting coefficients. So, we decided against calculating the wave 2 weight for vote choice for respondents in that region. Also, especially for waves 3 and 4, the weighting coefficients are substantial, and thus should be used with great care.

- *weight_w1_AgeSexEdu*: age, gender and education (respondents that finished wave 1):
- *weight_w1w2_AgeSexEdu*: age, gender and education (respondents that finished wave 1 and 2):
- *weight_w1w2_AgeSexEduVote*: age, gender, education and vote for Chamber (2019) (respondents that finished wave 1 and 2, only for Flanders and Wallonia);
- *weight_w1w2w3_AgeSexEdu*: age, gender and education (respondents that finished wave 1, 2 and 3, only for Flanders and Wallonia);
- *weight_w1w2w3w4_AgeSexEdu*: age, gender and education (respondents that finished wave 1, 2, 3 and 4, only for Flanders and Wallonia).

4. Codebook questionnaire

4.1 Wave 1 questionnaire

Variable name	Label + Values [English]	Label + Values [Dutch]	Label + Values [French]	Min	Max
w1_Finished	**Did respondent complete the wave 1 questionnaire?**<br>1. Yes			1	1
w2_Finished	**Did respondent complete the wave 2 questionnaire?**<br>1. Yes			1	1
w3_Finished	**Did respondent complete the wave 3 questionnaire?**<br>1. Yes			1	1
w4_Finished	**Did respondent complete the wave 4 questionnaire?**<br>1. Yes			1	1
w1_Region	1. Brussels<br>2. Flanders<br>3. Wallonia	1. Brussel<br>2. Vlaanderen<br>3. Wallonie	1. Bruxelles<br>2. Flandre<br>3. Wallon	1	3
w1_Duration	**Duration of respondent's time to complete the questionnaire of wave 1 (in seconds)**			152	1628562
w1_RecordedDate	**Date on which the respondent's response for wave 1 was recorded**			5 April 2019	21 May 2019
w1_Q_Language	**Language setting for the respondent in wave 1**<br>0. French<br>1. Dutch			0	1
w1_Consent	**Did respondent agree with the informed consent form?**<br>1. Yes<br>2. No			1	1
w1_Stemgerechtigd	**Are you vote eligible for the elections of May 26 2019?**<br>1. Yes<br>2. No	**Bent u stemgerechtigd voor de verkiezingen van 26 mei 2019?**<br>**1.** Ja<br>**2.** Nee	**Avez-vous le droit de voter aux élections du 26 mai 2019?**<br>**1.** Oui<br>**2.** Non	1	2
w1_Age	**What is your age?**	**Wat is uw leeftijd?**	**Quel âge avez-vous?**	18	110
w1_Gender	**What is your gender?**<br>1. Male	**Wat is uw geslacht?**<br>1. Man	**Quel est votre sexe?**<br>1. Homme	1	3

	2. Female<br>3. Other	2. Vrouw<br>3. Anders	2. Femme<br>3. Autre		
w1_Gender_3_Text	**Text**	**Tekst**	**Texte**		
w1_Edu	**What is the highest level of education you have achieved?**<br>1. None or elementary education<br>2. Secondary education, incomplete (lower ASO, BSO or TSO)<br>3. Secondary education, complete (ASO, BSO or TSO)<br>4. Higher non-universitary education<br>5. Universitary education	**Wat is uw hoogst behaalde opleidingsniveau?**<br>1. Geen of lager onderwijs<br>2. Middelbaar onderwijs, niet volledig afgewerkt (lager ASO, BSO of TSO)<br>3. Middelbaar onderwijs, volledig afgewerkt (ASO, BSO of TSO)<br>4. Hoger niet-universitair onderwijs<br>5. Universitair onderwijs	**Quel est le diplôme le plus élevé que vous avez obtenu?**<br>1. Aucun ou primaire<br>2. Secondaire, incomplet<br>3. Secondaire, complet<br>4. Supérieur non-universitaire<br>5. Universitaire	1	5
w1_PostalCode	**What is the postal code of your main place of residence?**	**Wat is de postcode van uw hoofdverblijfplaats?**	**Quel est le code postal de votre lieu de résidence principale?**		
w1_Empl	**Which of the following choices best describes your current work situation?**<br>1. Student, in unremunerated training<br>2. Pre-retired<br>3. Retired<br>4. Houseman / housewife (takes care of the household and/or a person in the household: children, elderly,...)<br>5. Unemployed / jobseeker	**Hoe zou u uw professionele bezigheden omschrijven? Klik één van de mogelijkheden aan.**<br>1. Student, in onbetaalde opleiding<br>2. Vervroegd gepensioneerde<br>3. Gepensioneerde<br>4. Huisman of –vrouw (onderhoudt het huishouden en/of draagt zorg voor een persoon in het huishouden: kinderen, ouderen, …)<br>5. Werkloos / werkzoekende<br>6. Arbeidsongeschikt	**Lequel des choix suivants décrit le mieux votre condition de travail actuelle?**<br>1. Etudiant, en formation non rémunérée<br>2. Pré-retraité<br>3. Retraité, pensionné<br>4. Personne au foyer (entretient le ménage et/ou s'occupe d'une personne dans le ménage: (enfants, personnes âgées, ...)	1	13

	6. On permanent incapacity to work<br>7. Liberal profession (doctor, lawyer, notary,...) such as independent<br>8. Merchant, artisan or other independent profession<br>9. Unskilled worker<br>10. Skilled worker<br>11. Employee<br>12. Executive<br>13. Other:	7. Vrij beroep (dokter, advocaat, notaris,…), als zelfstandige<br>8. Handelaar, ambachtsman of andere zelfstandige<br>9. Ongeschoolde arbeider<br>10. Geschoolde arbeider<br>11. Bediende<br>12. Kaderfunctie<br>13. Andere:	5. Chômeur / demandeur d'emploi<br>6. En incapacité permanente<br>7. Profession libérale (médecin, avocat, notaire…), comme indépendant<br>8. Commerçant, artisan ou autre indépendant<br>9. Ouvrier non-qualifié<br>10. Ouvrier qualifié<br>11. Employé<br>12. Employé supérieur, cadre<br>13. Autre:		
w1_Empl_13_Text	**Text**	**Tekst**	**Text**		
w1_Sat_Inc	**To what extent are you satisfied with your family's total income? [0-10 scale: 0 = Very unsatisfied; 10 = Very satisfied; 99 = I do not know]**	**Hoe tevreden bent u met uw gezinsinkomen? [0-10 scale: 0 = Uiterst ontevreden; 10 = Uiterst tevreden; 99 = Weet niet]**	**Dans quelle mesure êtes-vous satisfait de la totalité du revenu de familial? [0-10 scale: 0 = Pas du tout satisfait; 10 = Tout à fait satisfait; 99 = Je ne sais pas]**	0	10
w1_Relig	**Do you consider yourself as…?**<br>1. Catholic<br>2. Protestant<br>3. Christian orthodoxe<br>4. Christian, but not catholic or orthodox<br>5. Muslim<br>6. Jewish<br>7. Lay<br>8. Agnostic<br>9. Atheïst<br>10. Other conviction	**Beschouwt u zichzelf als…?**<br>1. Katholiek<br>2. Protestants<br>3. Orthodox Christelijk<br>4. Christelijk, maar niet Katholiek, Protestants of Orthodox<br>5. Moslim<br>6. Joods<br>7. Vrijzinnig<br>8. Agnost<br>9. Atheïst<br>10. Andere overtuiging	**Vous considérez-vous personnellement comme ... ?**<br>1. Catholique<br>2. Protestant<br>3. Chrétien orthodoxe<br>4. Chrétien (ni catholique, ni protestant ou orthodoxe)<br>5. Musulman<br>6. Juif<br>7. Laïc<br>8. Agnostique<br>9. Athée	1	12

	11. No conviction<br>12. I prefer not to say	11. Geen overtuiging<br>12. Wil ik liever niet zeggen	10. Autre conviction<br>11. Pas de conviction<br>12. Je préfère ne pas le dire		
w1_Left_Right	**In politics people often talk of “left” or “right”. Can you place your own convictions on a scale from 0 to 10, with 0 meaning “left” 5 “in the centre”, and 10 “right”? [0-10 scale: 0 = Left; 5 = Center; 10 = Right]**	**In de politiek worden de termen ‘links’ en ‘rechts’ vaak gebruikt. Kan u uw eigen opvattingen plaatsen op een schaal van 0 tot 10, waarbij 0 ‘links’, 5 ‘in het centrum’ en 10 ‘rechts’ betekent? [0-10 scale: 0 = Links; 5 = In het centrum; 10 = Rechts]**	**On utilise souvent en politique les notions de 'gauche' et de 'droite'. Pouvez-vous situer vos propres opinions sur une échelle allant de 0 à 10, où 0 signifie 'gauche', 5 'le centre', et 10 'droite'? [0-10 scale: 0 = Gauche; 5 = Le centre; 10 = Droite]**	0	10
w1_PolInt	**To what extent are you interested in politics in general? [0-10 scale: 0 = Not interested at all; 10 = Extremely interested]**	**In welke mate heeft u belangstelling voor politiek in het algemeen? [0-10 scale: 0 = Helemaal geen belangstelling; 10 = Heel veel belangstelling]**	**Dans quelle mesure êtes-vous intéressé par la politique en général? [0-10 scale: 0 = Aucun interet; 10 = Énormément intéressé(e)]**	0	10
[If region = Flanders]<br>w1_VoteFed14_FL	**For which party did you vote for the Chamber at the last national elections (in May 2014)?**<br>1. CD&V<br>2. Groen<br>3. N-VA<br>4. Open VLD<br>5. PVDA<br>6. sp.a<br>7. Vlaams Belang<br>8. Other<br>9. Blank or invalid<br>10. Did not vote<br>11. Was not (yet) eligible to vote	**Op welke partij stemde u voor de Kamer bij de vorige nationale verkiezingen (in mei 2014)?**<br>1. CD&V<br>2. Groen<br>3. N-VA<br>4. Open VLD<br>5. PVDA<br>6. sp.a<br>7. Vlaams Belang<br>8. Andere<br>9. Blanco of Ongeldig gestemd<br>10. Niet gaan stemmen<br>11. Mocht (nog) niet stemmen<br>12. Ik weet het niet meer	**Pour quel parti avez-vous voté à la Chambre des Représentants lors des dernières élections fédérales (en mai 2014)?**<br>1. CD&V<br>2. Groen<br>3. N-VA<br>4. Open VLD<br>5. PVDA<br>6. sp.a<br>7. Vlaams Belang<br>8. Autre<br>9. J’ai voté blanc / invalide<br>10. Je n’ai pas voté	1	12

	12. I do not remember		11. Je ne pouvais pas (encore) voter<br>12. Je ne m'en rappelle pas		
[If region = Wallonia] w1_VoteFed14_Wal	**For which party did you vote for the Chamber at the last national elections (in May 2014)?**<br>1. cdH<br>2. Ecolo<br>3. DéFI<br>4. PP<br>5. MR<br>6. PS<br>7. PTB<br>8. Other<br>9. Blank or invalid<br>10. I did not vote<br>11. I could not (yet) vote<br>12. I do not remember	**Op welke partij stemde u voor de Kamer bij de vorige nationale verkiezingen (in mei 2014)?**<br>1. cdH<br>2. Ecolo<br>3. DéFI<br>4. PP<br>5. MR<br>6. PS<br>7. PTB<br>8. Andere<br>9. Blanco of Ongeldig gestemd<br>10. Niet gaan stemmen<br>11. Mocht (nog) niet stemmen<br>12. Ik weet het niet meer	**Pour quel parti avez-vous voté à la Chambre des Représentants lors des dernières élections fédérales (en mai 2014)?**<br>1. cdH<br>2. Ecolo<br>3. DéFI<br>4. PP<br>5. MR<br>6. PS<br>7. PTB<br>8. Autre<br>9. J'ai voté blanc / invalide<br>10. Je n'ai pas voté<br>11. Je ne pouvais pas (encore) voter<br>12. Je ne m'en rappelle pas	1	12
[If region = Brussels] w1_VoteFed14_Bxl	**For which party did you vote for the Chamber at the last national elections (in May 2014)?**<br>1. CD&V<br>2. cdH<br>3. DéFI<br>4. Ecolo-Groen<br>5. MR<br>6. N-VA<br>7. Open VLD<br>8. PP<br>9. PS<br>10. PTB-PVDA	**Op welke partij stemde u voor de Kamer bij de vorige nationale verkiezingen (in mei 2014)?**<br>1. CD&V<br>2. cdH<br>3. DéFI<br>4. Ecolo-Groen<br>5. MR<br>6. N-VA<br>7. Open VLD<br>8. PP<br>9. PS<br>10. PTB-PVDA	**Pour quel parti avez-vous voté à la Chambre des Représentants lors des dernières élections fédérales (en mai 2014)?**<br>1. CD&V<br>2. cdH<br>3. DéFI<br>4. Ecolo-Groen<br>5. MR<br>6. N-VA<br>7. Open VLD<br>8. PP<br>9. PS	1	17

	11. sp.a<br>12. Vlaams Belang<br>13. Other<br>14. Blank or invalid<br>15. I did not vote<br>16. I could not (yet) vote<br>17. I do not remember	11. sp.a<br>12. Vlaams Belang<br>13. Andere<br>14. Blanco of Ongeldig gestemd<br>15. Niet gaan stemmen<br>16. Mocht (nog) niet stemmen<br>17. Ik weet het niet meer	10. PTB-PVDA<br>11. sp.a<br>12. Vlaams Belang<br>13. Autre<br>14. J'ai voté blanc / invalide<br>15. Je n'ai pas voté<br>16. Je ne pouvais pas (encore) voter<br>17. Je ne m'en rappelle pas		
[If region = Flanders]<br>w1_VoteFed19_Fl	**If there were national elections for the Chamber today, which party would you vote?**<br>1. CD&V<br>2. Groen<br>3. N-VA<br>4. Open VLD<br>5. PVDA<br>6. sp.a<br>7. Vlaams Belang<br>8. Other<br>9. Blank<br>10. Cannot vote (yet)<br>11. I do not know	**Als er vandaag al federale verkiezingen voor de Kamer zouden zijn, op welke van de volgende partijen zou u dan stemmen?**<br>1. CD&V<br>2. Groen<br>3. N-VA<br>4. Open VLD<br>5. PVDA<br>6. sp.a<br>7. Vlaams Belang<br>8. Andere<br>9. Blanco<br>10. Mag (nog) niet stemmen<br>11. Weet ik niet	**S'il y avait des élections fédéral à la Chambre aujourd'hui, pour quel parti voteriez-vous?**<br>1. CD&V<br>2. Groen<br>3. N-VA<br>4. Open VLD<br>5. PvdA<br>6. sp.a<br>7. Vlaams Belang<br>8. Autre<br>9. Blanc<br>10. Ne peut pas (encore) voter<br>11. Je ne sais pas	1	11
[If region = Wallonia]<br>w1_VoteFed19_Wal	**If there were national elections for the Chamber today, for which party would you vote?**<br>1. cdH<br>2. Ecolo<br>3. DéFI<br>4. PP	**Als er vandaag al federale verkiezingen voor de Kamer zouden zijn, op welke van de volgende partijen zou u dan stemmen?**<br>1. cdH<br>2. Ecolo<br>3. DéFI	**S'il y avait des élections fédéral à la Chambre aujourd'hui, pour quel parti voteriez-vous?**<br>1. cdH<br>2. Ecolo<br>3. DéFI<br>4. PP	1	11

	5. MR<br>6. PS<br>7. PTB<br>8. Other<br>9. Blank<br>10. Cannot vote (yet)<br>11. I do not know	4. PP<br>5. MR<br>6. PS<br>7. PTB<br>8. Andere<br>9. Blanco<br>10. Mag (nog) niet stemmen<br>11. Weet ik niet	5. MR<br>6. PS<br>7. PTB<br>8. Autre<br>9. Blanc<br>10. Ne peut pas (encore) voter<br>11. Je ne sais pas		
[If region = Brussels]<br>w1_VoteFed19_Bxl	**If there were national elections for the Chamber today, for which party would you vote?**<br>1. CD&V<br>2. cdH<br>3. DéFI<br>4. Ecolo-Groen<br>5. MR<br>6. N-VA<br>7. Open VLD<br>8. PP<br>9. PS<br>10. PTB-PVDA<br>11. sp.a<br>12. Vlaams Belang<br>13. Other<br>14. Blank<br>15. Cannot vote (yet)<br>16. I do not know	**Als er vandaag al federale verkiezingen voor de Kamer zouden zijn, op welke van de volgende partijen zou u dan stemmen?**<br>1. CD&V<br>2. cdH<br>3. DéFI<br>4. Ecolo-Groen<br>5. MR<br>6. N-VA<br>7. Open VLD<br>8. PP<br>9. PS<br>10. PTB-PVDA<br>11. sp.a<br>12. Vlaams Belang<br>13. Andere<br>14. Blanco<br>15. Mag (nog) niet stemmen<br>16. Weet ik niet	**S'il y avait des élections fédéral à la Chambre aujourd'hui, pour quel parti voteriez-vous?**<br>1. CD&V<br>2. cdH<br>3. DéFI<br>4. Ecolo-Groen<br>5. MR<br>6. N-VA<br>7. Open VLD<br>8. PP<br>9. PS<br>10. PTB-PVDA<br>11. sp.a<br>12. Vlaams Belang<br>13. Autre<br>14. Blanc<br>15. Ne peut pas (encore) voter<br>16. Je ne sais pas	1	16
[If region = Flanders]<br>w1_VoteReg_Fl	**If there were elections at the Flemish Parliament today, for which party would you vote?**	**Als er vandaag al verkiezingen voor het Vlaams parlement zouden zijn, op**	**S'il y avait des élections au Parlement flamand aujourd'hui, pour quel parti voteriez-vous?**	1	11

	1. CD&V<br>2. Groen<br>3. N-VA<br>4. Open VLD<br>5. PVDA<br>6. sp.a<br>7. Vlaams Belang<br>8. Other<br>9. Blank<br>10. Cannot vote (yet)<br>11. I do not know	**welke van de volgende partijen zou u dan stemmen?**<br>1. CD&V<br>2. Groen<br>3. N-VA<br>4. Open VLD<br>5. PVDA<br>6. sp.a<br>7. Vlaams Belang<br>8. Andere<br>9. Blanco<br>10. Mag (nog) niet stemmen<br>11. Weet ik niet	1. CD&V<br>2. Groen<br>3. N-VA<br>4. Open VLD<br>5. PVDA<br>6. sp.a<br>7. Vlaams Belang<br>8. Autre<br>9. Blanc<br>10. Ne peut pas (encore) voter<br>11. Je ne sais pas		
[If region = Wallonia]<br>w1_VoteReg_Wal	**If there were elections at the Walloon Parliament today, for which party would you vote?**<br>1. cdH<br>2. Ecolo<br>3. DéFI<br>4. PP<br>5. MR<br>6. PS<br>7. PTB<br>8. Other<br>9. Blank<br>10. Cannot vote (yet)<br>11. I do not know	**Als er vandaag al verkiezingen voor het Waals parlement zouden zijn, op welke van de volgende partijen zou u dan stemmen?**<br>1. cdH<br>2. Ecolo<br>3. DéFI<br>4. PP<br>5. MR<br>6. PS<br>7. PTB<br>8. Andere<br>9. Blanco<br>10. Mag (nog) niet stemmen<br>11. Weet ik niet	**S'il y avait des élections au Parlement wallon aujourd'hui, pour quel parti voteriez-vous?**<br>1. cdH<br>2. Ecolo<br>3. DéFI<br>4. PP<br>5. MR<br>6. PS<br>7. PTB<br>8. Autre<br>9. Blanc<br>10. Ne peut pas (encore) voter<br>11. Je ne sais pas	1	11
[If region = Brussels]<br>w1_VoteReg_Bxl	**If there were elections at the Parliament of Brussels today, for which party would you vote?**<br>1. CD&V<br>2. cdH	**Als er vandaag al verkiezingen voor het Brussels parlement zouden zijn, op welke van de volgende partijen zou u dan stemmen?**<br>1. CD&V	**S'il y avait des élections au Parlement bruxellois aujourd'hui, pour quel parti voteriez-vous?**<br>1. CD&V<br>2. CDH	1	18

	3. DéFI<br>4. Ecolo<br>5. Groen<br>6. MR<br>7. N-VA<br>8. Open VLD<br>9. PP<br>10. PS<br>11. PTB<br>12. PVDA<br>13. sp.a<br>14. Vlaams Belang<br>15. Other<br>16. Blank<br>17. Cannot vote (yet)<br>18. I do not know	2. CDH<br>3. DéFI<br>4. Ecolo<br>5. Groen<br>6. MR<br>7. N-VA<br>8. Open VLD<br>9. PP<br>10. PS<br>11. PTB<br>12. PVDA<br>13. sp.a<br>14. Vlaams Belang<br>15. Andere<br>16. Blanco<br>17. Mag (nog) niet stemmen<br>18. Weet ik niet	3. DéFI<br>4. Ecolo<br>5. Groen<br>6. MR<br>7. N-VA<br>8. Open VLD<br>9. PP<br>10. PS<br>11. PTB<br>12. PVDA<br>13. sp.a<br>14. Vlaams Belang<br>15. Autre<br>16. Blanc<br>17. Ne peut pas (encore) voter<br>18. Je ne sais pas		
[If region = Flanders]<br>w1_VoteEU_Fl	**If there were elections at the European Parliament today, for which party would you vote?**<br>1. CD&V<br>2. Groen<br>3. N-VA<br>4. Open VLD<br>5. PVDA<br>6. sp.a<br>7. Vlaams Belang<br>8. Other<br>9. Blank<br>10. Cannot vote (yet)<br>11. I do not know	**Als er vandaag al verkiezingen voor het Europees parlement zouden zijn, op welke van de volgende partijen zou u dan stemmen?**<br>1. CD&V<br>2. Groen<br>3. N-VA<br>4. Open VLD<br>5. PVDA<br>6. sp.a<br>7. Vlaams Belang<br>8. Andere<br>9. Blanco<br>10. Mag (nog) niet stemmen<br>11. Weet ik niet	**S'il y avait des élections au Parlement européen aujourd'hui, pour quel parti voteriez-vous?**<br>1. CD&V<br>2. Groen<br>3. N-VA<br>4. Open VLD<br>5. PVDA<br>6. sp.a<br>7. Vlaams Belang<br>8. Autre<br>9. Blanc<br>10. Ne peut pas (encore) voter<br>11. Je ne sais pas	1	11

[If region = Wallonia]<br>w1_VoteEU_Wal	**If there were elections at the European Parliament today, for which party would you vote?**<br>1. cdH<br>2. Ecolo<br>3. DéFI<br>4. PP<br>5. MR<br>6. PS<br>7. PTB<br>8. Other<br>9. Blank<br>10. Cannot vote (yet)<br>11. I do not know	**Als er vandaag al verkiezingen voor het Europees parlement zouden zijn, op welke van de volgende partijen zou u dan stemmen?**<br>1. cdH<br>2. Ecolo<br>3. DéFI<br>4. PP<br>5. MR<br>6. PS<br>7. PTB<br>8. Andere<br>9. Blanco<br>10. Mag (nog) niet stemmen<br>11. Weet ik niet	**S'il y avait des élections au Parlement européen aujourd'hui, pour quel parti voteriez-vous?**<br>1. cdH<br>2. Ecolo<br>3. DéFI<br>4. PP<br>5. MR<br>6. PS<br>7. PTB<br>8. Autre<br>9. Blanc<br>10. Ne peut pas (encore) voter<br>11. Je ne sais pas	1	11
[If region = Brussels]<br>w1_VoteEU_Bxl	**If there were elections at the European Parliament today, for which party would you vote?**<br>1. CD&V<br>2. cdH<br>3. DéFI<br>4. Ecolo<br>5. Groen<br>6. MR<br>7. N-VA<br>8. Open VLD<br>9. PP<br>10. PS<br>11. PTB<br>12. PVDA<br>13. sp.a	**Als er vandaag al verkiezingen voor het Europees parlement zouden zijn, op welke van de volgende partijen zou u dan stemmen?**<br>1. CD&V<br>2. cdH<br>3. DéFI<br>4. Ecolo<br>5. Groen<br>6. MR<br>7. N-VA<br>8. Open VLD<br>9. PP<br>10. PS<br>11. PTB<br>12. PVDA	**S'il y avait des élections au Parlement européen aujourd'hui, pour quel parti voteriez-vous?**<br>1. CD&V<br>2. cdH<br>3. DéFI<br>4. Ecolo<br>5. Groen<br>6. MR<br>7. N-VA<br>8. Open VLD<br>9. PP<br>10. PS<br>11. PTB<br>12. PVDA<br>13. sp.a	1	18

	14. Vlaams Belang<br>15. Other<br>16. Blank<br>17. Cannot vote (yet)<br>18. I do not know	13. sp.a<br>14. Vlaams Belang<br>15. Andere<br>16. Blanco<br>17. Mag (nog) niet stemmen<br>18. Weet ik niet	14. Vlaams Belang<br>15. Autre<br>16. Blanc<br>17. Ne peut pas (encore) voter<br>18. Je ne sais pas		
w1_Conv/Unconv_Partic_1 to w1_Conv/Unconv_Partic_9	**There are different ways to improve things in Belgium or to be more politically active. How often did you do any of the following actions in the past 12 months? [1 = Never; 2 = Seldom; 3 = Sometimes; 4 = Often]**<br>1. Actively participated in a political party.<br>2. Contacted a politician by post or email.<br>3. Displayed a poster of a political party or politician.<br>4. Expressed your opinion on politics on social media (such as Facebook, Twitter, Instagram or blogs).<br>5. Was active in a social movement or interest group.<br>6. Signed a petition on- or offline.	**Er zijn verschillende manieren om te proberen dingen in België te verbeteren of om politiek actief te zijn. Hoe vaak heeft u in de afgelopen 12 maanden één van de volgende dingen gedaan? [1 = Nooit; 2 = Zelden; 3 = Soms; 4 = Vaak]**<br>1. Actief zijn voor een politieke partij<br>2. Een politicus contacteren via brieven of emails<br>3. Een affiche van een politieke partij of politicus ophangen<br>4. Mijn mening over politiek geven op sociale media (zoals bijvoorbeeld Facebook, Twitter, Instagram of blogs)<br>5. Actief zijn voor een sociale beweging of belangengroep<br>6. Een online of offline petitie ondertekenen<br>7. Deelnemen aan een protestactie, protestmars of betoging	**Il existe différentes façons pour tenter d'améliorer la situation en Belgique ou d'être politiquement actif. À quelle fréquence avez-vous fait l'une de choses suivantes au cours des 12 derniers mois? [1 = Jamais; 2 = Rarement; 3 = Parfois; 4 = Souvent]**<br>1. Être actif dans un parti politique<br>2. Contacter un homme ou une femme politique par courrier ou par e-mail<br>3. Placer une affiche d'un parti politique ou d'un homme/d'une femme politique<br>4. Exprimer mon opinion sur la politique sur les médias sociaux (tels que Facebook, Twitter, Instagram ou les blogs)<br>5. Être actif dans un mouvement social ou un groupe d'intérêt	1	4

	7. Participate in an action of protest, a protest march or a demonstration.<br>8. Bought or refused to buy certain products for political, ethical or environmental reasons.<br>9. Broken the rules for political reasons.	8. Bepaalde producten kopen of juist niet kopen voor politieke, ethische of milieubewuste redenen<br>9. De regels breken voor politieke redenen	6. Signer une pétition en ligne ou hors ligne<br>7. Participer à une action de protestation, une marche de protestation ou une manifestation<br>8. Acheter ou refuser d'acheter certains produits pour des raisons politiques, éthiques ou environnementales<br>9. Enfreindre les règles pour des raisons politiques		
w1_PolKnowl1	**The following six questions assess your general knowledge on politics. If you do not know the answer, you can simply respond "I do not know". The federal parliament is composed of…**<br>1. The Chamber of Representatives and the government<br>2. The Chamber of Representatives and the Senate<br>3. The Chamber of Representatives, the Senate and the parliaments of the federal entities	**Nu volgen er een zestal vragen die peilen naar uw algemene kennis over politiek. Als u het antwoord niet weet, duidt dan 'Weet niet' aan.**<br>**Het federale parlement bestaat uit...**<br>1. De Kamer en de regering<br>2. De Kamer en de Senaat<br>3. De Kamer, de Senaat en de parlementen van de deelstaten<br>4. De Kamer, de Senaat en de regering<br>99. Weet niet	**Les six questions suivantes évaluent votre connaissance générale de la politique. Si vous ne connaissez pas la réponse, vous pouvez simplement répondre 'Je ne sais pas'.**<br>**Le parlement fédéral est composé de…**<br>1. La Chambre et le gouvernement<br>2. La Chambre et le Sénat<br>3. La Chambre, le Sénat et les parlements des entités fédérées<br>4. La Chambre, le Sénat et le gouvernement<br>99. Je ne sais pas	1	4

	4. The Chamber of Representatives, the Senate and the government<br>99. I do not know				
w1_PolKnowl2	**The president of the Chamber of Representatives is...**<br>1. Jan Peumans<br>2. Siegfried Bracke<br>3. Herman Van Rompuy<br>4. Didier Reynders<br>99. I do not know	**De voorzitter van de Kamer van Volksvertegenwoordigers is...**<br>1. Jan Peumans<br>2. Siegfried Bracke<br>3. Herman Van Rompuy<br>4. Didier Reynders<br>99. Weet niet	**Le président de la Chambre des représentants est ...**<br>1. Jan Peumans<br>2. Siegfried Bracke<br>3. Herman Van Rompuy<br>4. Didier Reynders<br>99. Je ne sais pas	1	4
w1_PolKnowl3	**How many states are in the European Union?**<br>1. 16 to 20<br>2. 21 to 25<br>3. 26 to 30<br>4. 31 to 35<br>99. I do not know	**Hoeveel lidstaten telt de Europese Unie?**<br>1. 16 tot 20<br>2. 21 tot 25<br>3. 26 tot 30<br>4. 31 tot 35<br>99. Weet niet	**Combien d'Etats comporte l'Union européenne?**<br>1. 16 à 20<br>2. 21 à 25<br>3. 26 à 30<br>4. 31 à 35<br>99. Je ne sais pas	1	4
[If region = Flanders]<br>w1_PolKnowl4_VL	**Who is the Flemish Minister of Mobility?**<br>1. Geert Bourgeois<br>2. Koen Geens<br>3. Sven Gatz<br>4. Ben Weyts<br>99. I do not know	**Wie is de Vlaamse Minister van Mobiliteit?**<br>1. Geert Bourgeois<br>2. Koen Geens<br>3. Sven Gatz<br>4. Ben Weyts<br>99. Weet niet	**Qui est le ministre de la mobilité en Flandre?**<br>1. Geert Bourgeois<br>2. Koen Geens<br>3. Sven Gatz<br>4. Ben Weyts<br>99. Je ne sais pas	1	4
[If region = Wallonia]<br>w1_PolKnowl4_Wal	**Who is the Walloon Minister of Mobility?**<br>1. Willy Borsus<br>2. Marie Christine Marghem<br>3. Pierre-Yves Jeholet<br>4. Carlo di Antonio	**Wie is de Waalse Minister van Mobiliteit?**<br>1. Willy Borsus<br>2. Marie Christine Marghem<br>3. Pierre-Yves Jeholet<br>4. Carlo di Antonio	**Qui est le ministre de la mobilité en Wallonie?**<br>1. Willy Borsus<br>2. Marie Christine Marghem<br>3. Pierre-Yves Jeholet<br>4. Carlo di Antonio	1	4

	99. I do not know	99. Weet niet	99. Je ne sais pas		
[If region = Brussels] w1_PolKnowl4_Bxl	**Who is the Minister of Mobility in the Brussels Capital Region?**<br>1. Rudi Vervoort<br>2. Marie Christine Marghem<br>3. Guy Vanhengel<br>4. Pascal Smet<br>99. I do not know	**Wie is de Brusselse Minister van Mobiliteit?**<br>1. Rudi Vervoort<br>2. Marie Christine Marghem<br>3. Guy Vanhengel<br>4. Pascal Smet<br>99. Weet niet	**Qui est le ministre de la mobilité dans la région de Bruxelles-Capitale?**<br>1. Rudi Vervoort<br>2. Marie Christine Marghem<br>3. Guy Vanhengel<br>4. Pascal Smet<br>99. Je ne sais pas	1	4
w1_PolKnowl5	**Who elects the members of the European Parliament?**<br>1. The national governments of the European Union's member-states<br>2. The heads of state of the European Union's member-states<br>3. The citizens of the European Union's member-states<br>4. The European Commission	**Wie kiest de leden van het Europese Parlement?**<br>1. De nationale regeringen van de landen van de Europese Unie<br>2. De staatshoofden van de landen van de Europese Unie<br>3. De burgers in elk land van de Europese Unie<br>4. De Europese Commissie<br>99. Weet niet	**Qui élit les membres du Parlement européen?**<br>1. Les gouvernements nationaux des pays de l'Union européenne<br>2. Les chefs d'État des pays de l'Union européenne<br>3. Les citoyens de tous les pays de l'Union européenne<br>4. La Commission européenne<br>99. Je ne sais pas	1	4
w1_PolKnowl6	**Which political issue is primarily handled on a european level rather than on the national or regional level?**<br>1. Education<br>2. Trade<br>3. Social security<br>4. Defence<br>99. I do not know	**Welk beleidsdomein wordt hoofdzakelijk bepaald op het EU-niveau, in plaats van op het nationale of sub-nationale niveau?**<br>1. Onderwijs<br>2. Handel<br>3. Sociale zekerheid<br>4. Defensie<br>99. Weet niet	**Quel enjeu politique est principalement géré au niveau européen plutôt qu'au niveau national ou régional?**<br>1. Enseignement<br>2. Commerce<br>3. Sécurité sociale<br>4. Défense<br>99. Je ne sais pas	1	4

w1_Eval_Gov_1 to w1_Eval_Gov_5	**To what extent are you satisfied with the policies implemented by the following political decision-making entities in the past few years? [0-10 scale: 0 = Very unsatisfied; 10 = Very satisfied]**<br>1. The European Union<br>2. The federal government<br>3. The Flemish government<br>4. The government of Brussels<br>5. The Walloon government	**Hoe tevreden bent u over de inhoud van het beleid dat op volgende bestuursniveaus werd gevoerd in de voorbije jaren? [0-10 scale: 0 = Heel ontevreden; 10 = Heel tevreden]**<br>1. De Europese Unie<br>2. De Federale regering<br>3. De Vlaamse regering<br>4. De Brusselse regering<br>5. De Waalse regering	**Dans quelle mesure êtes-vous satisfait(e) de la politique mise en œuvre aux niveaux de décision politique suivants au cours des dernières années? [0-10 scale: 0 = Très insatisfait; 10 = Très satisfait]**<br>1. L'Union Européenne<br>2. Gouvernement fédéral<br>3. Gouvernement flamand<br>4. Gouvernement bruxellois<br>5. Gouvernement wallon	0	10
w1_Eval_Pop_1 to w1_Eval_Pop_5	**To what extent do you think that the average [Fleming/Brussels resident/Walloon] is satisfied with the policies conducted on the following levels of government in the past few years? [0-10 scale: 0 = Very unsatisfied; 10 = Very satisfied]**<br>1. The European Union<br>2. The federal government<br>3. The Flemish government<br>4. The government of Brussels<br>5. The Walloon government	**Hoe tevreden denkt u dat de gemiddelde [Vlaming/Brusselaar/Waal] is over de inhoud van het beleid dat op volgende bestuursniveaus werd gevoerd in de voorbije jaren? [0-10 scale: 0 = Heel ontevreden; 10 = Heel tevreden]**<br>1. De Europese Unie<br>2. De Federale regering<br>3. De Vlaamse regering<br>4. De Brusselse regering<br>5. De Waalse regering	**Dans quelle mesure pensez-vous que les [Flamands/Wallons/Bruxellois] sont satisfaits de la politique menée aux niveaux de pouvoir suivants au cours des dernières années? [0-10 scale: 0 = Très insatisfait; 10 = Très satisfait]**<br>1. L'Union Européenne<br>2. Gouvernement fédéral<br>3. Gouvernement flamand<br>4. Gouvernement bruxellois<br>5. Gouvernement wallon	0	10
w1_Issue_salience_1 [First issue] to w1_Issue_salience_2 [Second issue]	**Could you indicate which would be the most important issues that would determine your vote if elections took place today? You can indicate a maximum of two issues. [1 = Employment; 2 =**	**Kunt u aangeven welke van deze thema's het belangrijkst zijn bij het maken van uw stemkeuze als er nu verkiezingen zouden zijn? U kunt maximaal twee thema's aanduiden. [1 = Werkgelegenheid; 2 = Milieu; 3 =**	**Pouvez-vous indiquer quels seraient les thèmes les plus importants pour déterminer votre vote si les élections avaient lieu maintenant? Vous pouvez indiquer un maximum de deux**	1	10

	**Environment; 3 = Crime; 4 - Migration; 5 = Economy; 6 = State reforms; 7 = Defence; 8 = Taxation; 9 = Social security; 10 = Functioning of democracy]**	**Criminaliteit; 4 = Migratie; 5 = Economie; 6 = Staatshervormingen; 7 = Defensie; 8 = Belastingen; 9 = Sociale zekerheid; 10 = Functioneren van de democratie]**	**thèmes. [1 = Emploi; 2 = Environnement; 3 = Crime; 4 = Migration; 5 = Economie; 6 = Réformes de l'État; 7 = Défense; 8 = Fiscalité; 9 = Sécurité sociale; 10 = Fonctionnement de la démocratie]**		
w1_Feel_Repres_1 to w1_Feel_Repres_3	**Could you indicate the extent to which you agree or disagree with the following statements? [0-10 scale: 0 = Totally disagree; 10 = Totally agree]**<br>1. I believe that there is a political party and/or politician that shares my opinions<br>2. There is not one political party and/or politician that properly represents my interests in general.<br>3. I find that some of my main concerns are addressed in the programme of a political party.	**Kunt u aangeven in hoeverre u het eens bent met onderstaande stellingen? [0-10 scale: 0 = Helemaal oneens; 10 = Helemaal eens]**<br>1. Ik heb het gevoel dat er een politieke partij en/of politicus is die een groot deel van mijn opinies deelt.<br>2. Er is geen enkele politieke partij en/of politicus die mijn belangen in het algemeen goed vertegenwoordigt.<br>3. Een aantal zaken die ik echt belangrijk vind, vind ik terug in het programma van een politieke partij.	**Pouvez-vous indiquer dans quelle mesure vous êtes en accord avec les affirmations suivantes? [0-10 scale: 0 = Pas du tout d'accord; 10 = Tout à fait d'accord]**<br>1. Je pense qu'il y a un parti politique et/ou un homme ou une femme politique qui partage une grande partie de mes opinions.<br>2. Il n'y a pas un seul parti politique et/ou un homme ou une femme politique qui bien représenté mes intérêts en général.<br>3. Je retrouve une partie de mes préoccupations les plus importantes dans le programme d'un parti politique.	0	10
w1_Sat_Dem	**Overall, how satisfied are you with the way democracy is working in Belgium?**	**In het algemeen, bent u eerder tevreden of eerder ontevreden over de**	**En général, êtes-vous plutôt satisfait ou plutôt insatisfait de la**	1	5

	1. Very satisfied<br>2. Somewhat satisfied<br>3. Not satisfied, nor unsatisfied<br>4. Somewhat unsatisfied<br>5. Very unsatisfied	**manier waarop de democratie in België werkt?**<br>1. Heel tevreden<br>2. Eerder tevreden<br>3. Noch tevreden, noch ontevreden<br>4. Eerder ontevreden<br>5. Heel ontevreden	**façon dont la démocratie fonctionne en Belgique?**<br>1. Très satisfait<br>2. Plutôt satisfait<br>3. Ni satisfait, ni insatisfait<br>4. Plutôt insatisfait<br>5. Très insatisfait		
w1_Estim_Voters_1 to w1_Estim_Voters_3	**According to you, how many [Flemings/Walloons/Brussels residents] are satisfied and unsatisfied with the functioning of democracy in Belgium? [0-100 percentages]**<br>1. % Satisfied<br>2. % Not satisfied, nor unsatisfied<br>3. % Unsatisfied	**Volgens u, hoeveel % van de [Vlamingen/Walen/Brusselaars] zijn ontevreden en tevreden over het functioneren van de democratie in België? [0-100 percentages]**<br>1. % Tevreden<br>2. %Tevreden noch ontevreden<br>3. % Ontevreden	**Selon vous, combien de [Flamands/Wallons/Bruxellois] sont mécontents et combien sont satisfaits du fonctionnement de la démocratie en Belgique? [0-100 percentages]**<br>1. % Satisfait<br>2. % Ni satisfait, ni insatisfait<br>3. % Insatisfait	0	100
w1_Emo_Gen_Pol_1 to w1_Emo_Gen_Pol_8	**When you think of Belgian politics in general, to what extent do you feel each of the following emotions? [0-10 scale: 0 = Not at all; 10 = To a great extent]**<br>1. Anger<br>2. Bitterness<br>3. Anxiety<br>4. Fear<br>5. Hope<br>6. Relief<br>7. Happiness<br>8. Contentment	**Als u denkt aan de Belgische politiek in het algemeen, in welke mate ervaart u dan elk van de onderstaande emoties? [0-10 scale: 0 = Helemaal niet; 10 = In sterke mate]**<br>1. Boosheid<br>2. Bitterheid<br>3. Ongerustheid<br>4. Angst<br>5. Hoop<br>6. Opluchting<br>7. Blijdschap<br>8. Tevredenheid	**Lorsque vous pensez à la politique belge en général, dans quelle mesure ressentez-vous chacune des émotions suivantes? [0-10 scale: 0 = Pas du tout; 10 = Dans une grande mesure]**<br>1. La colère<br>2. l'amertume<br>3. L'inquiétude<br>4. la peur<br>5. L'espoir<br>6. Soulagement<br>7. La joie<br>8. La satisfaction	0	10

w1_Trust_Pol_1 to w1_Trust_Pol_4	**On a scale of 0 to 10, what is your level of confidence in each of the following institutions? [0-10 scale: 0 = Absolutely no confidence; 10 = Complete confidence]**<br>1. Political parties<br>2. The federal parliament<br>3. Politicians<br>4. The European Union	**Kunt u op een schaal van 0 tot 10 aangeven hoeveel vertrouwen u persoonlijk heeft in elk van de volgende instellingen? [0-10 scale: 0 = Helemaal geen vertrouwen; 10 = Volledig vertrouwen]**<br>1. De politieke partijen<br>2. Het federale parlement<br>3. Politici<br>4. De Europese Unie	**Pouvez-vous indiquer sur une échelle de 0 à 10 quel est votre niveau de confiance envers chacune des institutions suivantes? [0-10 scale: 0 = Absolument pas confiance; 10 = Totalement confiance]**<br>1. Les partis politiques<br>2. Le parlement fédéral<br>3. Les hommes/femmes politiques<br>4. L'Union européenne	0	10
w1_Cynisme_1 to w1_Cynisme_7	**Please indicate to what extent you disagree or agree with the following statements. [1 = Totally disagree; 2 = Somewhat disagree; 3 = Neither agree nor disagree; 4 = Somewhat agree; 5 = Totally agree]**<br>1. Politicians are corrupt<br>2. Most politicians are competent.<br>3. Politicians try to keep their promises.<br>4. Politicians do not understand what is going on in society.<br>5. Many politicians have been in place for too long.<br>6. The way we organise elections in this country is fair.	**Kunt u aangeven in hoeverre u het eens bent met onderstaande stellingen? [1 = Helemaal oneens; 2 = Eerder oneens; 3 = Noch eens, noch oneens; 4 = Eerder eens; 5 = Helemaal eens]**<br>1. Politici zijn corrupt.<br>2. De meeste politici zijn competent.<br>3. Politici proberen hun beloftes te houden.<br>4. Politici begrijpen niet wat er speelt in de samenleving.<br>5. Veel politici draaien al te lang mee.<br>6. De manier waarop we verkiezingen organiseren in dit land is eerlijk.<br>7. Partijen houden voldoende rekening met onafhankelijke experten wanneer ze beleid maken.	**Pouvez-vous indiquer dans quelle mesure vous êtes d'accord avec les affirmations ci-dessous? [1 = Pas du tout d'accord; 2 = Plutôt pas d'accord; 3 = Ni d'accord, ni pas d'accord; 4 = Plutôt d'accord; 5 = Tout à fait d'accord]**<br>1. Les hommes/femmes politiques sont corrompus.<br>2. La plupart des hommes/femmes politiques sont compétents.<br>3. Les hommes /femmes politiques essaient de tenir leurs promesses.<br>4. Les hommes /femmes politiques ne comprennent pas ce qui se passe dans la société.	1	5

	7. Political parties adequately take into account independent experts when they make decisions.		5. Beaucoup d'hommes/femmes politiques sont là depuis trop longtemps.<br>6. La façon dont nous organisons les élections dans ce pays est juste.<br>7. Les partis politiques tiennent suffisamment compte des experts indépendants lorsqu'ils prennent des décisions.		
w1_Populisme_1 to w1_Populisme_7	**Please indicate to what extent you disagree or agree with the following statements.** **[1 = Totally disagree; 2 = Somewhat disagree; 3 = Neither agree nor disagree; 4 = Somewhat agree; 5 = Totally agree]**<br>1. Politicians must follow the people's opinion.<br>2. Political opposition is more present between citizens and the elite than between citizens themselves.<br>3. I prefer being represented by an ordinary citizen rather than by a professional politician.	**Kunt u aangeven in hoeverre u het eens bent met onderstaande stellingen?** **[1 = Helemaal oneens; 2 = Eerder oneens; 3 = Noch eens, noch oneens; 4 = Eerder eens; 5 = Helemaal eens]**<br>1. Politici in het parlement moeten zich laten leiden door de mening van het volk.<br>2. De politieke tegenstellingen zijn groter tussen de elite en gewone burgers dan tussen burgers onderling.<br>3. Ik word liever vertegenwoordigd door een gewone burger dan door een beroepspoliticus.<br>4. Bij het nemen van besluiten geven politici om mensen zoals ik.	**Pouvez-vous indiquer dans quelle mesure vous êtes d'accord avec les affirmations ci-dessous?** **[1 = Pas du tout d'accord; 2 = Plutôt pas d'accord; 3 = Ni d'accord, ni pas d'accord; 4 = Plutôt d'accord; 5 = Tout à fait d'accord]**<br>1. Les hommes et femmes politiques doivent suivre l'avis de la population.<br>2. L'opposition politique est plus grande entre les élites et les citoyens, qu'entre les citoyens entre eux.<br>3. Je préfère être représenté par un citoyen ordinaire que par un homme/ femme politique professionnel.	1	5

	4. When making decisions, politicians care about people like me.<br>5. Politics is the result of compromise and common sense.<br>6. Rich citizens have a bigger influence on politics than do poor citizens.<br>7. In general, politics reflect rather well the people's preferences.	5. Beleid is doorgaans het resultaat van compromis en gezond verstand.<br>6. Rijke burgers hebben meer invloed op het beleid dan arme burgers.<br>7. Over het algemeen weerspiegelt het beleid de voorkeuren van het volk redelijk goed.	4. En prenant des décisions, les hommes /femmes politiques se soucient des gens comme moi.<br>5. La politique est le résultat du compromis et du bon sens.<br>6. Les citoyens riches ont plus d'influence sur les politiques que les citoyens pauvres.<br>7. En général, la politique reflète assez bien les préférences de la population.		
w1_Efficacy_1 to w1_Efficacy_8<br><br>* The Dutch and French translations to not fully overlap.	**Please indicate to what extent you disagree or agree with the following statements. [1 = Totally disagree; 2 = Somewhat disagree; 3 = Neither agree nor disagree; 4 = Somewhat agree; 5 = Totally agree]**<br>1. Most citizens do not have clear political preferences.<br>2. Political parties do not offer real political alternatives to the people.<br>3. Political parties give too much freedom to campaign advisers to	**Kunt u aangeven in hoeverre u het eens bent met onderstaande stellingen? [1 = Helemaal oneens; 2 = Eerder oneens; 3 = Noch eens, noch oneens; 4 = Eerder eens; 5 = Helemaal eens]**<br>1. De meeste burgers hebben geen duidelijke beleidsvoorkeuren.<br>2. Politieke partijen bieden het volk geen echt verschillende beleidsalternatieven.<br>3. Politieke partijen laten campagneadviseurs teveel hun visies over politieke thema's bepalen.*<br>4. De invloed van belangengroepen en lobbyisten op beleid is te groot.	**Pouvez-vous indiquer dans quelle mesure vous êtes d'accord avec les affirmations ci-dessous? [1 = Pas du tout d'accord; 2 = Plutôt pas d'accord; 3 = Ni d'accord, ni pas d'accord; 4 = Plutôt d'accord; 5 = Tout à fait d'accord]**<br>1. La plupart des citoyens n'ont pas de préférence politique claire.<br>2. Les partis politiques n'offrent pas à la population de véritables alternatives politiques.<br>3. Les partis politiques laissent trop les conseillers de campagne	1	5

	determine important political issues. * 4. The influence of interest groups and lobbyists on policies is too big. 5. Voting is pointless because parties do what they want anyway. 6. In general, our political system functions fairly. 7. Our political decision-making processes are sufficiently transparent. 8. In general, our political system functions in an efficient way.	5. Gaan stemmen is zinloos, partijen doen toch wat ze zelf willen. 6. In het algemeen werkt ons politiek systeem op een eerlijke manier. 7. Onze politieke besluitvormingsprocessen zijn voldoende transparant. 8. In het algemeen werkt ons politiek systeem op een efficiënte manier.	déterminer les thèmes politiques importants.* 4. L'influence des groupes d'intérêts et des lobbys sur les politiques est trop grande. 5. Aller voter ne sert à rien, les partis font toujours ce qu'ils veulent. 6. En général, notre système politique fonctionne de manière honnête. 7. Nos processus de décision politique sont suffisamment transparents. 8. En général, notre système politique fonctionne efficacement.		
w1_Value_1	**Some people think that the government must intervene as little as possible in the market. Other people think that the government must intervene as much as possible in the market. [0-10 scale: 0 = The government must intervene as little as possible; 10 = The government must intervene as much as possible]**	**Sommigen vinden dat de overheid zo weinig mogelijk moet ingrijpen in de economie. Anderen vinden dat de overheid zo veel mogelijk moet ingrijpen in de economie. [0-10 scale: 0 = De overheid moet zo weinig mogelijk ingrijpen; 10 = De overheid moet zo veel mogelijk ingrijpen]**	**Certaines personnes pensent que le gouvernement doit intervenir le moins possible dans l'économie, d'autres pensent que le gouvernement doit intervenir autant que possible dans l'économie. [0-10 scale: 0 = Le gouvernement doit intervenir le moins possible; 10 = Le gouvernement doit intervenir autant que possible]**	0	10
w1_Value_2	**According to some, the people that lead a good life do so due to**	**Volgens sommigen hebben mensen die het goed hebben dat vooral te danken**	**Selon certains, les personnes qui mènent une bonne vie la mènent**	0	10

	good fortune. Others think that these people lead a good life because of their own actions. [0-10 scale: 0 = The fate of people depends entirely on chance; 10 = The fate of people depends entirely on their own actions]	aan het feit dat ze gewoon geluk hebben gehad. Volgens anderen hebben mensen die het goed hebben dat vooral aan zichzelf en hun harde werk te danken. [0-10 scale: 0 = Het lot van mensen hangt volledig af van geluk; 10 = Het lot van mensen hangt volledig af van eigen verdienste]	grâce à la chance. D'autres pensent que ces personnes mènent une bonne vie grâce à leurs propres actions. [0-10 scale: 0 = Le sort des personnes dépend complètement de la chance; 10 = Le sort des personnes dépend complètement de leurs propres actions]		
w1_Value_3	Some people think that non-western immigrant must be able to live in Europe while preserving their own culture. Others think that those immigrants should adapt to the european culture. [0-10 scale: 0 = Completely preserve their own culture; 10 = Completely adapt to the European culture]	Sommigen vinden dat niet-westerse allochtonen in Europa moeten kunnen leven met behoud van hun eigen cultuur. Anderen vinden dat zij zich moeten aanpassen aan de Europese cultuur. [0-10 scale: 0 = Volledig eigen cultuur kunnen behouden; 10 = Volledig aanpassen aan de Europese cultuur]	Certains pensent que les immigrés non-occidentaux doivent pouvoir vivre en Europe tout en conservant leur culture d'origine. D'autres pensent que ces immigrés doivent s'adapter à la culture européenne. [0-10 scale: 0 = Garder totalement sa propre culture; 10 = Totalement s'intégrer à la culture européenne]	0	10
w1_Value_4<br><br>*[Note: The answers of respondents who completed the survey in French were removed, because there was an issue in the French translation of the scale (scale mismatched the question wording).*	Some think that euthanasia (choosing to end one's own life on his or her own initiative in case of incurable disease) should be allowed. Others think that euthanasia should in no case be an option. [0-10 scale: 0 = Euthanasia should not be allowed in any case; 10 = Euthanasia must be allowed]	Sommigen vinden dat euthanasie (het op eigen verzoek beëindigen van het leven in geval van ongeneeslijke ziekte) moet worden toegestaan. Anderen vinden dat euthanasie in geen enkel geval mogelijk mag zijn. [0-10 scale: 0 = Euthanasie in geen enkel geval mogelijk; 10 = Euthanasie in elk geval mogelijk]	Certains trouvent que l'euthanasie (choisir de mettre fin à ses jours de sa propre initiative en cas de maladie incurable) doit être autorisé. D'autres pensent que l'euthanasie ne devrait en aucun cas être possible. [0-10 scale: 0 = L'euthanasie ne doit en aucun cas être autorisée; 10 = L'euthanasie ne doit être autorisée en aucune circonstance]	0	10

Question was re-asked in second wave (w2_Value4]					
w1_Value_5	**Some people think that innovative technology is sufficient to halt global warming. Others think that fundamental changes in people's behavior are also necessary to halt global warming. [0-10 scale: 0 = Innovative technology is sufficient to halt global warming; 10 = Fundamental behavioral changes are absolutely necessary]**	**Sommige mensen menen dat de opwarming van de aarde kan worden gestopt door technologische innovaties alleen. Anderen denken dat ook fundamentele gedragswijzigingen nodig zijn om de opwarming van de aarde te stoppen. [0-10 scale: 0 = Technologische innovaties volstaan; 10 = Gedragswijzigingen zijn nodig]**	**Certains pensent que les innovations technologiques suffisent pour arrêter le réchauffement de la Terre. D'autres pensent que des changements de comportement fondamentaux sont aussi nécessaires pour arrêter le réchauffement de la Terre. [0-10 scale: 0 = Les innovations technologiques suffisent pour arrêter le réchauffement de la Terre; 10 = Changements comportementaux sont absolument nécessaires]**	0	10
w1_Value_6	**To what extent do you agree or disagree with the following statement? "I would give a part of my revenue if I were certain that it would help the environment". [0-10 scale: 0 = Totally disagree; 10 = Totally agree]**	**In welke mate gaat u akkoord met de volgende stelling: "Ik zou een deel van mijn inkomen afstaan als ik er zeker van was dat dit het milieu zou verbeteren". [0-10 scale: 0 = Helemaal niet akkoord; 10 = Helemaal akkoord]**	**Dans quelle mesure êtes vous d'accord avec la proposition suivante?: "Je donnerais une partie de mon revenu si j'étais certain que cela aiderait l'environnement". [0-10 scale: 0 = Pas du tout d'accord; 10 = Tout à fait d'accord]**	0	10
w1_Eval_DemProc_1 to w1_Eval_DemProc_9	**In Belgium, democracy is organized according to a number of legal rules and informal practices. We will introduce you to a few. For each of these rules that organize democracy in Belgium, can you tell us what you**	**In België is de democratie geregeld door verschillende wettelijke regels en informele gewoonten. Hieronder leggen we er een aantal aan u voor. We zouden graag uw mening hierover kennen. Kan u voor elk van deze regels of gewoonten aangeven hoe u erover**	**En Belgique, la démocratie est organisée selon un certain nombre de règles légales et de pratiques informelles. Nous allons vous en présenter quelques-unes. Pour chacune de ces règles qui organisent la**	1	4

	**think? [1 = Very negative; 2 = Somewhat negative; 3 = Somewhat positive; 4 = Very positive; 99 = I do not know/no opinion]**<br>1. Free elections are held every five years to appoint the members who will sit in Parliament.<br>2. The governments are composed of coalitions between several parties. These parties must negotiate after the elections to find an agreement in order to form a government together.<br>3. Apart from voting during elections, citizens are currently not involved in the political decision-making processes.<br>4. In Belgium, there are currently 50 ministers and 467 parliamentarians split across three levels of government (federal, regional and community level).<br>5. In Belgium, the net monthly income of a federal member is	**denkt? [1 = Heel negatief; 2 = Eerder negatief; 3 = Eerder positief; 4 = Heel positief; 99 = Weet niet/Geen mening]**<br>1. Elke vijf jaar worden er vrije verkiezingen georganiseerd om de parlementsleden te verkiezen.<br>2. Regeringen zijn coalities tussen verschillende partijen. Na de verkiezingen moeten deze partijen onderhandelen om tot een overeenkomst te komen, en zo samen een regering te vormen.<br>3. Momenteel zijn burgers zelden betrokken bij politieke beslissingen, behalve bij de verkiezingen.<br>4. In België zijn er momenteel 50 ministers en 467 parlementsleden, verspreid over 3 beleidsniveaus (federaal, gemeenschap en gewest).<br>5. In België ligt het nettoloon van een federale volksvertegenwoordiger tussen 5500 en 6000 euro per maand.<br>6. Op dit ogenblik is het mogelijk om op hetzelfde moment zowel parlementslid als burgemeester of schepen te zijn.	**démocratie en Belgique, pouvez-vous nous dire ce que vous en pensez? [1 = Très négatif; 2 = Plutôt négatif; 3 = Plutôt positif; 4 = Très positif; 99 = Ne sais pas/sans opinion]**<br>1. Des élections libres sont organisées tous les cinq ans pour désigner les député.e.s qui siègeront au Parlement.<br>2. Les gouvernements sont des coalitions entre plusieurs partis. Ces partis doivent négocier après les élections pour se mettre d'accord pour former un gouvernement ensemble.<br>3. Actuellement, en dehors des élections les citoyens sont rarement impliqués dans la prise de décision politique.<br>4. En Belgique, il y a actuellement 50 ministres, et 467 parlementaires répartis à 3 niveaux de pouvoir (fédéral, régional, communautaire).<br>5. En Belgique, le salaire mensuel net d'un(e)		

	between 5500 and 6600 net euros per month.<br>6. Currently, it is possible to be at the same time member of parliament, mayor and alderman.<br>7. Currently, it is the party leaders who decide who will be proposed to become minister.<br>8. In Belgium, in public companies, the Board of Directors is composed of persons designated by the political parties.<br>9. Currently in Belgium, political power is divided into five levels of government: the communes, the provinces, the regions, the communities and the federal state.	7. Momenteel beslissen de partijvoorzitters wie er minister wordt.<br>8. In Belgische overheidsbedrijven is de Raad van Bestuur samengesteld uit personen die benoemd zijn door de politieke partijen.<br>9. De politieke macht is in België verdeeld over vijf bevoegdheidsniveaus: het lokale niveau, de provincies, de gemeenschappen en gewesten, en het federale niveau.	député(e) fédéral(e) se situe entre 5500 et 6000 euros net par mois.<br>6. Actuellement, il est possible d'être en même temps député et bourgmestre ou échevin.<br>7. Actuellement, ce sont les présidents de parti qui décident qui sera proposé pour devenir ministre.<br>8. En Belgique, dans les entreprises publiques, le Conseil d'Administration est composé de personnes désignées par les partis politiques.<br>9. Actuellement, en Belgique, le pouvoir politique est distribué entre cinq niveaux de pouvoir : les communes, les provinces, les régions, les communautés, et l'état fédéral.		
w1_Sup_Reforms_1 to w1_Sup_Reforms_9	**Currently, several propositions for reform of the Belgian democracy are being debated. We will introduce you to a few. For each of these reforms, could you indicate if you are completely against, somewhat against,**	**Op dit moment wordt er gediscussieerd over meerdere voorstellen om de Belgische democratie te hervormen. Kan u voor onderstaande voorstellen aangeven of u er volledig voor, eerder voor, eerder tegen, of volledig tegen bent? [1 =**	**Plusieurs propositions de réformes de la démocratie belge sont débattues actuellement. Nous allons vous en citer quelques-unes. Pour chacune des réformes, pourriez-vous nous dire si vous êtes totalement pour,**	1	4

* The Dutch and French translations to not fully overlap.	**somewhat in favour, or totally in favour? [1 = Totally against; 2 = Somewhat against; 3 = Somewhat in favour; 4 = Totally in favour; 99 = I do not know/no opinion]**<br>1. Federal, regional and european elections should be held every 10 years instead of every 5 years.<br>2. The election law should be changed to give the majority to a single party that could govern alone.<br>3. The number of ministers and parliamentarians in Belgium should be cut in half.<br>4. The income of the elected should be limited to a maximum of 2500 euros gross per month.*<br>5. Cumulating the mandates of mayor and member of parliament should be banned.<br>6. Ministers should be elected directly by the voters.<br>7. The appointments for inter-communal companies, public	**Helemaal tegen; 2 = Eerder tegen; 3 = Eerder voor; 4 = Helemaal voor; 99 = Weet niet/geen mening]**<br>1. Federale, regionale en Europese verkiezingen moeten elke 10 jaar worden georganiseerd in plaats van elke 5 jaar.<br>2. De kieswet moet veranderen zodat één partij een meerderheid kan halen en dus alleen kan regeren.<br>3. Het aantal ministers en parlementsleden in België moet gehalveerd worden.<br>4. Het salaris van verkozenen moet beperkt worden tot maximaal €2500 per maand.*<br>5. Het cumuleren van het mandaat van burgemeester en dat van volksvertegenwoordiger moet verboden worden.<br>6. Ministers moeten rechtstreeks door de kiezer worden verkozen.<br>7. Alle benoemingen in intercommunales, publieke bedrijven en bij de overheid moeten beslist worden door een agentschap dat onafhankelijk is van politieke partijen.	**plutôt pour, plutôt contre, ou totalement contre? [1 = Totalement contre; 2 = Plutôt contre; 3 = Plutôt pour; 4 = Totalement pour; 99 = Ne sais pas/sans opinion]**<br>1. Les élections fédérales, régionales et européennes devraient être organisées tous les 10 ans plutôt que tous les 5 ans.<br>2. La loi électorale devrait être changée pour donner la majorité à un seul parti qui pourrait gouverner seul.<br>3. Le nombre de ministres et de parlementaires en Belgique devrait être divisé par deux.<br>4. Le salaire des élus devrait être limité à maximum 2500 euros brut par mois.*<br>5. Le cumul des mandats de bourgmestre et de député devrait être interdit.<br>6. Les ministres devraient être désignés directement par les électeurs.		

	companies and administrations should be done by an agency independent from political parties.<br>8. Provinces should be abolished.<br>9. Experts should take the major political decisions instead of politicians.	8. De provincies moeten worden afgeschaft.<br>9. Experten moeten de belangrijkste politieke beslissingen nemen in plaats van politici.	7. Toutes les nominations dans les intercommunales, les entreprises publiques et l'administration devraient être faites par une agence indépendante des partis politiques.<br>8. Les provinces devraient être supprimées.<br>9. Des experts devraient prendre les principales décisions politiques à la place des hommes et femmes politiques.		
w1_Sup_Cit_exp_1 to w1_Sup_Cit_Exp_6<br><br>* The Dutch and French translations to not fully overlap.	**We are now going to ask you a series of questions on the way in which you evaluate citizens and experts as political decision-makers. * [0-10 scale: 0 = Totally disagree; 10 = Totally agree]**<br>1. Most citizens have all the competences required to make political decisions.<br>2. Most experts have all the competences required to make political decisions.<br>3. Most citizens are honest.<br>4. Most experts are honest.<br>5. Most citizens are capable of understanding the needs of people like me.	**We leggen u enkele stellingen voor over de manier waarop burgers en experten politieke beslissingen zouden nemen. * [0-10 scale: 0 = Helemaal oneens; 10 = Helemaal eens]**<br>1. De meeste burgers zijn competent om politieke beslissingen te nemen.<br>2. De meeste experts zijn competent om politieke beslissingen te nemen.<br>3. De meeste burgers zijn eerlijk.<br>4. De meeste experts zijn eerlijk.<br>5. De meeste burgers zijn in staat om de noden van personen zoals ik te begrijpen.	**Nous allons à présent vous poser une série de questions sur la façon dont vous évaluez les citoyens et les experts comme décideurs politiques.* [0-10 scale: 0 = Pas du tout d'accord; 10 = Tout à fait d'accord]**<br>1. La plupart des citoyens ont toutes les compétences requises pour prendre des décisions politiques.<br>2. La plupart des experts ont toutes les compétences requises pour prendre des décisions politiques.	0	10

	6. Most experts are capable of understanding the needs of people like me.	6. De meeste experts zijn in staat om de noden van personen zoals ik te begrijpen.	3. La plupart des citoyens sont honnêtes.<br>4. La plupart des experts sont honnêtes.<br>5. La plupart des citoyens sont capables de comprendre les besoins de personnes comme moi.<br>6. La plupart des experts sont capables de comprendre les besoins de personnes comme moi.		
w1_Network_1 to w1_Network_5	**When you think of the most important people in your life, who are they? [text]**<br>1. [Person 1]<br>2. [Person 2]<br>3. [Person 3]<br>4. [Person 4]<br>5. [Person 5]	**Als u denkt aan de belangrijkste personen in uw leven, wie zijn dat dan? [text]**<br>1. [Persoon 1]<br>2. [Persoon 2]<br>3. [Persoon 3]<br>4. [Persoon 4]<br>5. [Persoon 5]	**Quand vous pensez aux personnes les plus importantes dans votre vie, qui sont-elles ? [text]**<br>1. [Personne 1]<br>2. [Personne 2]<br>3. [Personne 3]<br>4. [Personne 4]<br>5. [Personne 5]		
w1_Network_Sat_1 to w1_Network_Sat_5	**Could you indicate the extent to which these persons are satisfied with the functioning of democracy in Belgium? [1 = Very satisfied; 2 = Satisfied; 3 = Not satisfied, nor unsatisfied; 4 = Satisfied; 5 = Very satisfied; 99 = I do not know]**<br>1. [Person 1]<br>2. [Person 2]	**Denk aan de mensen die u juist noemde. Kunt u voor elk van hen zeggen hoe (on)tevreden ze zijn over de manier waarop de democratie in België werkt? [1 = Helemaal ontevreden; 2 = Ontevreden; 3 = Noch tevreden, noch ontevreden; 4 = Tevreden; 5 = Heel tevreden; 99 = Weet ik niet]**<br>1. [Persoon 1]	**Pourriez-vous indiquer dans quelle mesure ces personnes sont satisfaites du fonctionnement de la démocratie en Belgique? [1 = Très insatisfait; 2 = Insatisfait; 3 = Ni satisfait, ni insatisfait; 4 = Satisfait; 5 = Très satisfait; 99 = Je ne sais pas]**<br>1. [Personne 1]<br>2. [Personne 2]	1	5

	3. [Person 3]<br>4. [Person 4]<br>5. [Person 5]	2. [Persoon 2]<br>3. [Persoon 3]<br>4. [Persoon 4]<br>5. [Persoon 5]	3. [Personne 3]<br>4. [Personne 4]<br>5. [Personne 5]		
w1_Expect_Policy_Outcom_1 to w1_Expect_Policy_Outcom_5	**According to you, how likely is it that you will agree with the decisions taken on the following levels of government in the next 5 years? [0-10 scale: 0 = Very unlikely; 10 = Very likely; 99 = I do not know]**<br>1. At the european level<br>2. At the belgian level<br>3. At the flemish level<br>4. At the Brussels level<br>5. At the walloon level	**Hoe waarschijnlijk is het volgens u dat beslissingen zullen worden genomen waarmee u het eens bent op de volgende regeringsniveaus in de komende vijf jaar? [0-10 scale: 0 = Zeer onwaarschijnlijk; 10 = Zeer waarschijnlijk; 99 = Weet niet]**<br>1. Op Europees niveau<br>2. Op Belgisch niveau<br>3. Op Vlaams niveau<br>4. Op Brussels niveau<br>5. Op Waals niveau	**Selon vous, quelle est la probabilité que vous soyez d'accord avec les décisions des niveaux de gouvernement suivants au cours des cinq prochaines années? [0-10 scale: 0 = Peu probable; 10 = Très probable; 99 = Je ne sais pas]**<br>1. Au niveau européen<br>2. Au niveau belge<br>3. Au niveau flamand<br>4. Au niveau bruxellois<br>5. Au niveau wallon	0	10
w1_Burgers_politici_1 to w1_Burgers_politici_6	**To what extent are you in favour or against the following statement? ”Important political decisions should more often be taken by ordinary citizens rather than by elected politicians”. [0-10 scale: 0= Strongly against; 10 = Strongly in favour]**<br>1. At the european level<br>2. At the belgian level<br>3. At the flemish level<br>4. At the Brussels level<br>5. At the walloon level<br>6. At the local level	**In welke mate bent u voor of tegen het volgende? “Belangrijke politieke beslissingen zouden vaker gemaakt moeten worden door gewone burgers en niet door verkozen politici.” [0-10 scale: 0 = Sterk tegen; 10 = Sterk voor]**<br>1. Op Europees niveau<br>2. Op Belgisch niveau<br>3. Op Vlaams niveau<br>4. Op Brussels niveau<br>5. Op Waals niveau<br>6. Op lokaal niveau	**Dans quelle mesure êtes-vous en faveur ou contre la proposition suivante? "Les décisions politiques importantes devraient être prises plus souvent par les citoyens ordinaires et non par les hommes et femmes politiques élus." [0-10 scale: 0 = Fortement contre; 10 = Fortement pour]**<br>1. Au niveau européen<br>2. Au niveau belge<br>3. Au niveau flamand<br>4. Au niveau bruxellois<br>5. Au niveau wallon<br>6. Au niveau local	0	10

w1_advis_ref	**In general, are you for or against consultative referendums about important national issues? Citizens have the right to vote for or against a specific proposition. The parliament receives the voters' opinion but is not obliged to follow it. [0-10 scale: Strongly against; 10 = Strongly in favour; 99 = I do not know]**	**Bent u, over het algemeen, voor of tegen adviserende referenda over belangrijke nationale thema's? Burgers hebben het recht om een stem uit te brengen over een specifiek voorstel. Het parlement ontvangt het advies van de kiezers, maar is niet verplicht om dit te volgen. [0-10 scale: 0 = Sterk tegen; 10 = Sterk voor; 99 = Weet niet]**	**Êtes-vous, en général, pour ou contre les référendums consultatifs sur les thèmes nationaux importants? Les citoyens ont le droit de voter sur une proposition spécifique. Le Parlement reçoit l'avis des électeurs mais sans être obligé de le suivre. [0-10 scale: 0 = Fortement contre; 10 = Fortement pour; 99 = Je ne sais pas]**	0	10
w1_Bindend_ref	**In general, are you for or against binding referendums about important national issues? Citizens have the right to vote for or against a specific proposition. The parliament receives the voters' opinion and is obliged to follow it. [0-10 scale: 0 = Strongly against; 10 = Strongly in favour; 99 = I do not know]**	**Bent u, over het algemeen, voor of tegen bindende referenda over belangrijke nationale thema's? Burgers hebben het recht om een stem uit te brengen over een specifiek voorstel. Het parlement is verplicht het advies van de kiezers te volgen. [0-10 scale: 0 = Sterk tegen; 10 = Sterk voor; 99 = Weet niet]**	**Êtes-vous, en général, pour ou contre les référendums contraignants sur les thèmes nationaux importants? Les citoyens ont le droit de voter sur une proposition spécifique. Le Parlement est obligé de suivre l'avis de la population. [0-10 scale: 0 = Fortement contre; 10 = Fortement pour; 99 = Je ne sais pas]**	0	10
w1_Advis_burgerforum	**In general, are you for or against the organisation of consultative citizen forums on important national issues? A citizen forum is an assembly composed of around 30 to 50 citizens, selected at random, who meet and discuss a certain topic in order to formulate a recommendation that is then transmitted to the**	**Bent u, over het algemeen, voor of tegen de organisatie van adviserende burgerfora over belangrijke nationale thema's? Een burgerforum bestaat uit 30 tot 50 burgers, die op basis van toeval geselecteerd zijn. Ze komen samen en discussiëren over een bepaald thema om zo tot een advies aan het parlement te komen. [0-10**	**Êtes-vous, en général, pour ou contre l'organisation de forums consultatifs de citoyens sur les thèmes nationaux importants? Un forum citoyen est une assemblée composée d'environ 30 à 50 citoyens, tirés au sort, qui se rencontrent et qui discutent d'un sujet spécifique afin de produire une recommandation**	0	10

	**parliament.** [0-10 scale: 0 = Strongly against; 10 = Strongly in favour; 99 = I do not know]	scale: 0 = Sterk tegen; 10 = Sterk voor; 99 = Weet niet]	**transmise ensuite au Parlement.** [0-10 scale: 0 = Fortement contre; 10 = Fortement pour; 99 = Je ne sais pas]		
w1_burgerbegroting	**In general, are you for or against participative budgeting on a national level? Participative budgeting consists of citizens deciding on a portion of the belgian state's budget. The citizens involved meet and discuss the way in which they wish to spend that amount in order to support different specific projects.** [0-10 scale: 0 = Strongly against; 10 = Strongly in favour; 99 = I do not know]	**Bent u, over het algemeen, voor of tegen burgerbegrotingen op nationaal niveau? Een burgerbegroting betekent dat burgers beslissen over een deel van de Belgische begroting. De betrokken burgers komen samen en discussiëren over hoe ze het geld wensen te verdelen overheen een aantal concrete projecten.** [0-10 scale: 0 = Sterk tegen; 10 = Sterk voor; 99 = Weet niet]	**Êtes-vous, en général, pour ou contre les budgets participatifs au niveau national? Un budget participatif signifie que des citoyens décident d'une partie du budget de l'Etat belge. Les citoyens impliqués se réunissent et discutent de la façon dont ils souhaitent que l'argent soit dépensé pour soutenir différents projets concrets.** [0-10 scale: 0 = Fortement contre; 10 = Fortement pour; 99 = Je ne sais pas]	0	10
w1_Experts_politic_1<br>to<br>w1_Experts_politic_6	**To what extent do you agree or disagree with the following statement? "Important political decisions should be taken more often by experts rather than elected politicians".** [0-10 scale: 0 = Totally disagree; 10 = Totally agree]<br>1. At the european level<br>2. At the belgian level<br>3. At the the flemish level<br>4. At the walloon level<br>5. At the Brussels level<br>6. At the local level	**In welke mate bent u voor of tegen het volgende?<br>"Belangrijke politieke beslissingen zouden vaker gemaakt moeten worden door experten en niet door verkozen politici."** [0-10 scale: 0 = Sterk tegen; 10 = Sterk voor]<br>1. Op Europees niveau<br>2. Op Belgisch niveau<br>3. Op Vlaams niveau<br>4. Op Waals niveau<br>5. Op Brussels niveau<br>6. Op lokaal niveau	**Dans quelle mesure êtes-vous en faveur ou contre la proposition suivante?<br>"Les décisions politiques importantes devraient être prises plus souvent par des experts et non par des hommes et femmes politiques élus."** [0-10 scale: 0 = Fortement contre; 10 = Fortement pour]<br>1. Au niveau européen<br>2. Au niveau belge<br>3. Au niveau flamand<br>4. Au niveau wallon<br>5. Au niveau bruxellois	0	10

			6. Au niveau local		
w1_VAA_Statements _1 to w1_VAA_Statements _18	**Below you will find a list of statements. Could you indicate the extent to which you agree or disagree with the following statements?** **[1 = Totally disagree; 2 = Disagree; 3 = Agree; 4 = Totally agree]**<br>1. Hosting transit migrants must be a punishable offence.<br>2. Situation tests must be put in place to detect discrimination in employment.<br>3. There must be a test on the european values in order to obtain the belgian nationality.<br>4. If the request for asylum of families with children is rejected, these families can be placed in detention pending their repatriation.<br>5. By 2024, the company cars that run with petrol or diesel must be banned.<br>6. The VAT on electricity must be reduced from 21 to 6%.	**Hieronder vindt u enkele stellingen. Kan u voor elk van volgende beleidsvoorstellen aangeven in welke mate u het ermee eens of oneens bent?** **[1 = Helemaal oneens; 2 = Eerder oneens; 3 = Eerder eens; 4 = Helemaal eens]**<br>1. Transmigranten onderdak bieden moet strafbaar zijn.<br>2. De overheid moet praktijktesten doen om te controleren op discriminatie bij aanwervingen.<br>3. Nieuwkomers moeten eerst slagen voor een examen over de Europese waarden voor ze de Belgische nationaliteit kunnen verwerven.<br>4. Als de asielaanvraag van gezinnen met kinderen is afgewezen, mogen ze in afwachting van hun terugkeer worden opgesloten.<br>5. Bedrijfswagens die op benzine of diesel rijden moeten voor 2024 verboden worden.<br>6. De btw op elektriciteit moet verlaagd worden van 21 naar 6%.<br>7. Vliegen moet duurder worden door de tickets te belasten.	**Ci-dessous vous trouverez une série de propositions. Pouvez-vous indiquer dans quelle mesure vous êtes d'accord ou pas d'accord avec les propositions suivantes?** **[1 = Pas du tout d'accord; 2 = Pas d'accord; 3 = D'accord; 4 = Tout à fait d'accord]**<br>1. Héberger des migrants en transit doit être un délit punissable.<br>2. Il faut mettre en place des tests de situation pour détecter la discrimination à l'embauche.<br>3. Pour pouvoir obtenir la nationalité belge, il faut d'abord avoir réussi un examen sur les valeurs européennes.<br>4. Si la demande d'asile de familles avec enfants a été rejetée, ces familles peuvent être placées en détention en attendant leur rapatriement.<br>5. D'ici 2024, les voitures de société qui roulent à l'essence ou au diesel doivent être interdites.	1	4

	7. There must be a tax on plane tickets in order to raise their price.<br>8. Nuclear power plants must remain operational after 2025.<br>9. We cannot drive while having drunk alcohol.<br>10. Abortion must be allowed beyond the 12th week of a pregnancy.<br>11. Sperm donation must no longer be anonymous.<br>12. Great fortunes must be more taxed.<br>13. Wages must no longer be automatically indexed.<br>14. The fingerprints of all citizens must be kept in a central database.<br>15. Shops must be able to choose when to do sales.<br>16. A retirement pension of at least 1500€ per month must be put in place.<br>17. The government should be composed of an equal number of men and women.<br>18. Important political decisions must be handled by citizens via a referendum.	8. Ook na 2025 moeten we kerncentrales openhouden.<br>9. Als je rijdt mag je helemaal geen alcohol gedronken hebben.<br>10. Ook na 12 weken zwangerschap moet abortus nog toegestaan worden.<br>11. Spermadonatie mag niet langer anoniem gebeuren.<br>12. Grote vermogens moeten meer worden belast.<br>13. De lonen mogen niet meer automatisch aan de prijsstijgingen worden aangepast (index).<br>14. Vingerafdrukken van alle burgers moeten in een centrale databank worden bijgehouden.<br>15. Winkels mogen zelf kiezen wanneer ze solden doen.<br>16. Er moet een minimumpensioen komen van minstens €1500 euro per maand.<br>17. Er moeten in de regering evenveel mannen als vrouwen zitten.<br>18. Belangrijke politieke beslissingen moeten via een referendum aan burgers kunnen worden overgelaten.	6. La TVA sur l'électricité doit être réduite de 21 à 6%.<br>7. Il faut taxer les billets d'avion pour qu'ils soient plus chers.<br>8. Les centrales nucléaires doivent rester opérationnelles après 2025.<br>9. On ne peut pas conduire en ayant bu de l'alcool.<br>10. L'avortement doit aussi être autorisé au-delà de 12 semaines de grossesse.<br>11. Le don de sperme ne doit plus pouvoir être anonyme.<br>12. Les grandes fortunes doivent être plus taxées.<br>13. Les salaires ne doivent plus être automatiquement indexés.<br>14. Les empreintes digitales de tous les citoyens doivent être conservées dans une base de données centrale.<br>15. Les magasins doivent pouvoir choisir quand ils font leurs soldes.		

			16. Il faut instaurer une pension de retraite de 1500€ minimum par mois.<br>17. Le gouvernement doit compter autant d'hommes que de femmes.<br>18. Les décisions politiques importantes doivent pouvoir être laissées aux citoyens par le biais d'un référendum.		
[If region = Flanders] w1_Perc_PartyPos_FL_1_1 to w1_Perc_PartyPos_FL_18_7	**For each of the following statements, could you indicate the parties that are, in your opinion, in favour of each statement? You can select several parties.** **[1 = CD&V; 2 = Groen; 3 = N-VA; 4 = Open VLD; 5 = PVDA; 6 = sp.a]; 7 = Vlaams Belang]**<br>1. Hosting transit migrants must be a punishable offence.<br>2. Situation tests must be put in place to detect discrimination in employment.<br>3. There must be a test on the european values in order to obtain the belgian nationality.	**Kunt u voor elk beleidsvoorstel de partijen aanduiden waarvan u denkt dat ze het met het voorstel eens zijn? U kunt hier meerdere partijen aanklikken.** **[1 = CD&V; 2 = Groen; 3 = N-VA; 4 = Open VLD; 5 = PVDA; 6 = sp.a]; 7 = Vlaams Belang]**<br>1. Transmigranten onderdak bieden moet strafbaar zijn.<br>2. De overheid moet praktijktesten doen om te controleren op discriminatie bij aanwervingen.<br>3. Nieuwkomers moeten eerst slagen voor een examen over de Europese waarden voor ze de Belgische nationaliteit kunnen verwerven.<br>4. Als de asielaanvraag van gezinnen met kinderen is	**Pour chaque proposition de politique, pouvez-vous indiquer les partis qui, selon vous, sont en accord avec la proposition? Vous pouvez sélectionner plusieurs partis.** **[1 = CD&V; 2 = Groen; 3 = N-VA; 4 = Open VLD; 5 = PVDA; 6 = sp.a]; 7 = Vlaams Belang]**<br>1. Héberger des migrants en transit doit être un délit punissable.<br>2. Il faut mettre en place des tests de situation pour détecter la discrimination à l'embauche.<br>3. Pour pouvoir obtenir la nationalité belge, il faut d'abord avoir réussi un		

	4. If the request for asylum of families with children is rejected, these families can be placed in detention pending their repatriation.<br>5. By 2024, the company cars that run with petrol or diesel must be banned.<br>6. The VAT on electricity must be reduced from 21 to 6%.<br>7. There must be a tax on plane tickets in order to raise their price.<br>8. Nuclear power plants must remain operational after 2025.<br>9. We cannot drive while having drunk alcohol.<br>10. Abortion must be allowed beyond the 12th week of a pregnancy.<br>11. Sperm donation must no longer be anonymous.<br>12. Great fortunes must be more taxed.<br>13. Wages must no longer be automatically indexed.<br>14. The fingerprints of all citizens must be kept in a central database.	afgewezen, mogen ze in afwachting van hun terugkeer worden opgesloten.<br>5. Bedrijfswagens die op benzine of diesel rijden moeten voor 2024 verboden worden.<br>6. De btw op elektriciteit moet verlaagd worden van 21 naar 6%.<br>7. Vliegen moet duurder worden door de tickets te belasten.<br>8. Ook na 2025 moeten we kerncentrales openhouden.<br>9. Als je rijdt mag je helemaal geen alcohol gedronken hebben.<br>10. Ook na 12 weken zwangerschap moet abortus nog toegestaan worden.<br>11. Spermadonatie mag niet langer anoniem gebeuren.<br>12. Grote vermogens moeten meer worden belast.<br>13. De lonen mogen niet meer automatisch aan de prijsstijgingen worden aangepast (index).<br>14. Vingerafdrukken van alle burgers moeten in een centrale databank worden bijgehouden.<br>15. Winkels mogen zelf kiezen wanneer ze solden doen.	examen sur les valeurs européennes.<br>4. Si la demande d'asile de familles avec enfants a été rejetée, ces familles peuvent être placées en détention en attendant leur rapatriement.<br>5. D'ici 2024, les voitures de société qui roulent à l'essence ou au diesel doivent être interdites.<br>6. La TVA sur l'électricité doit être réduite de 21 à 6%.<br>7. Il faut taxer les billets d'avion pour qu'ils soient plus chers.<br>8. Les centrales nucléaires doivent rester opérationnelles après 2025.<br>9. On ne peut pas conduire en ayant bu de l'alcool.<br>10. L'avortement doit aussi être autorisé au-delà de 12 semaines de grossesse.<br>11. Le don de sperme ne doit plus pouvoir être anonyme.<br>12. Les grandes fortunes doivent être plus taxées.		

	15. Shops must be able to choose when to do sales.<br>16. A retirement pension of at least 1500€ per month must be put in place.<br>17. The government should be composed of an equal number of men and women.<br>18. Important political decisions must be handled by citizens via a referendum.	16. Er moet een minimumpensioen komen van minstens €1500 euro per maand.<br>17. Er moeten in de regering evenveel mannen als vrouwen zitten.<br>18. Belangrijke politieke beslissingen moeten via een referendum aan burgers kunnen worden overgelaten.	13. Les salaires ne doivent plus être automatiquement indexés.<br>14. Les empreintes digitales de tous les citoyens doivent être conservées dans une base de données centrale.<br>15. Les magasins doivent pouvoir choisir quand ils font leurs soldes.<br>16. Il faut instaurer une pension de retraite de 1500€ minimum par mois.<br>17. Le gouvernement doit compter autant d'hommes que de femmes.<br>18. Les décisions politiques importantes doivent pouvoir être laissées aux citoyens par le biais d'un référendum.		
[If region = Wallonia] w1_Perc_PartyPos_WAL_1 to w1_Perc_PartyPos_WAL_18	**For each of the following statements, could you indicate the parties that are, in your opinion, in favour of each statement? You can select several parties. [1 = cdH; 2 = DéFI; 3 = Ecolo; 4 = MR; 5 = PP; 6 = PS; 7 = PTB]**	**Kunt u voor elk beleidsvoorstel de partijen aanduiden waarvan u denkt dat ze het met het voorstel eens zijn? U kunt hier meerdere partijen aanklikken. [1 = cdH; 2 = DéFI; 3 = Ecolo; 4 = MR; 5 = PP; 6 = PS; 7 = PTB]**<br>1. Transmigranten onderdak bieden moet strafbaar zijn.	**Pour chaque proposition de politique, pouvez-vous indiquer les partis qui, selon vous, sont en accord avec la proposition? Vous pouvez sélectionner plusieurs partis. [1 = cdH; 2 = DéFI; 3 = Ecolo; 4 = MR; 5 = PP; 6 = PS; 7 = PTB]**		

	1. Hosting transit migrants must be a punishable offence.<br>2. Situation tests must be put in place to detect discrimination in employment.<br>3. There must be a test on the european values in order to obtain the belgian nationality.<br>4. If the request for asylum of families with children is rejected, these families can be placed in detention pending their repatriation.<br>5. By 2024, the company cars that run with petrol or diesel must be banned.<br>6. The VAT on electricity must be reduced from 21 to 6%.<br>7. There must be a tax on plane tickets in order to raise their price.<br>8. Nuclear power plants must remain operational after 2025.<br>9. We cannot drive while having drunk alcohol.	2. De overheid moet praktijktesten doen om te controleren op discriminatie bij aanwervingen.<br>3. Nieuwkomers moeten eerst slagen voor een examen over de Europese waarden voor ze de Belgische nationaliteit kunnen verwerven.<br>4. Als de asielaanvraag van gezinnen met kinderen is afgewezen, mogen ze in afwachting van hun terugkeer worden opgesloten.<br>5. Bedrijfswagens die op benzine of diesel rijden moeten voor 2024 verboden worden.<br>6. De btw op elektriciteit moet verlaagd worden van 21 naar 6%.<br>7. Vliegen moet duurder worden door de tickets te belasten.<br>8. Ook na 2025 moeten we kerncentrales openhouden.<br>9. Als je rijdt mag je helemaal geen alcohol gedronken hebben.<br>10. Ook na 12 weken zwangerschap moet abortus nog toegestaan worden.<br>11. Spermadonatie mag niet langer anoniem gebeuren.	1. Héberger des migrants en transit doit être un délit punissable.<br>2. Il faut mettre en place des tests de situation pour détecter la discrimination à l'embauche.<br>3. Pour pouvoir obtenir la nationalité belge, il faut d'abord avoir réussi un examen sur les valeurs européennes.<br>4. Si la demande d'asile de familles avec enfants a été rejetée, ces familles peuvent être placées en détention en attendant leur rapatriement.<br>5. D'ici 2024, les voitures de société qui roulent à l'essence ou au diesel doivent être interdites.<br>6. La TVA sur l'électricité doit être réduite de 21 à 6%.<br>7. Il faut taxer les billets d'avion pour qu'ils soient plus chers.<br>8. Les centrales nucléaires doivent rester opérationnelles après 2025.		

	10. Abortion must be allowed beyond the 12th week of a pregnancy.<br>11. Sperm donation must no longer be anonymous.<br>12. Great fortunes must be more taxed.<br>13. Wages must no longer be automatically indexed.<br>14. The fingerprints of all citizens must be kept in a central database.<br>15. Shops must be able to choose when to do sales.<br>16. A retirement pension of at least 1500€ per month must be put in place.<br>17. The government should be composed of an equal number of men and women.<br>18. Important political decisions must be handled by citizens via a referendum.	12. Grote vermogens moeten meer worden belast.<br>13. De lonen mogen niet meer automatisch aan de prijsstijgingen worden aangepast (index).<br>14. Vingerafdrukken van alle burgers moeten in een centrale databank worden bijgehouden.<br>15. Winkels mogen zelf kiezen wanneer ze solden doen.<br>16. Er moet een minimumpensioen komen van minstens €1500 euro per maand.<br>17. Er moeten in de regering evenveel mannen als vrouwen zitten.<br>18. Belangrijke politieke beslissingen moeten via een referendum aan burgers kunnen worden overgelaten.	9. On ne peut pas conduire en ayant bu de l'alcool.<br>10. L'avortement doit aussi être autorisé au-delà de 12 semaines de grossesse.<br>11. Le don de sperme ne doit plus pouvoir être anonyme.<br>12. Les grandes fortunes doivent être plus taxées.<br>13. Les salaires ne doivent plus être automatiquement indexés.<br>14. Les empreintes digitales de tous les citoyens doivent être conservées dans une base de données centrale.<br>15. Les magasins doivent pouvoir choisir quand ils font leurs soldes.<br>16. Il faut instaurer une pension de retraite de 1500€ minimum par mois.<br>17. Le gouvernement doit compter autant d'hommes que de femmes.<br>18. Les décisions politiques importantes doivent		

			pouvoir être laissées aux citoyens par le biais d'un référendum.		
[If region = Brussels] [If language = NL] w1_Perc_PartyPos_BXL-NL_1 to w1_Perc_PartyPos_BXL-NL_18	**For each of the following statements, could you indicate the parties that are, in your opinion, in favour of each statement? You can select several parties. [1 = CD&V; 2 = Groen; 4 = N-VA; 5 = Open VLD; 6 = PVDA; 7 = sp.a; 8 = Vlaams Belang]**<br>1. Hosting transit migrants must be a punishable offence.<br>2. Situation tests must be put in place to detect discrimination in employment.<br>3. There must be a test on the european values in order to obtain the belgian nationality.<br>4. If the request for asylum of families with children is rejected, these families can be placed in detention pending their repatriation.<br>5. By 2024, the company cars that run with petrol or diesel must be banned.	**Kunt u voor elk beleidsvoorstel de partijen aanduiden waarvan u denkt dat ze het met het voorstel eens zijn? U kunt hier meerdere partijen aanklikken. [1 = CD&V; 2 = Groen; 4 = N-VA; 5 = Open VLD; 6 = PVDA; 7 = sp.a; 8 = Vlaams Belang]**<br>1. Transmigranten onderdak bieden moet strafbaar zijn.<br>2. De overheid moet praktijktesten doen om te controleren op discriminatie bij aanwervingen.<br>3. Nieuwkomers moeten eerst slagen voor een examen over de Europese waarden voor ze de Belgische nationaliteit kunnen verwerven.<br>4. Als de asielaanvraag van gezinnen met kinderen is afgewezen, mogen ze in afwachting van hun terugkeer worden opgesloten.<br>5. Bedrijfswagens die op benzine of diesel rijden moeten voor 2024 verboden worden.<br>6. De btw op elektriciteit moet verlaagd worden van 21 naar 6%.	**Pour chaque proposition de politique, pouvez-vous indiquer les partis qui, selon vous, sont en accord avec la proposition? Vous pouvez sélectionner plusieurs partis. [1 = CD&V; 2 = Groen; 4 = N-VA; 5 = Open VLD; 6 = PVDA; 7 = sp.a; 8 = Vlaams Belang]**<br>1. Héberger des migrants en transit doit être un délit punissable.<br>2. Il faut mettre en place des tests de situation pour détecter la discrimination à l'embauche.<br>3. Pour pouvoir obtenir la nationalité belge, il faut d'abord avoir réussi un examen sur les valeurs européennes.<br>4. Si la demande d'asile de familles avec enfants a été rejetée, ces familles peuvent être placées en détention en attendant leur rapatriement.<br>5. D'ici 2024, les voitures de société qui roulent à		

	6. The VAT on electricity must be reduced from 21 to 6%.<br>7. There must be a tax on plane tickets in order to raise their price.<br>8. Nuclear power plants must remain operational after 2025.<br>9. We cannot drive while having drunk alcohol.<br>10. Abortion must be allowed beyond the 12th week of a pregnancy.<br>11. Sperm donation must no longer be anonymous.<br>12. Great fortunes must be more taxed.<br>13. Wages must no longer be automatically indexed.<br>14. The fingerprints of all citizens must be kept in a central database.<br>15. Shops must be able to choose when to do sales.<br>16. A retirement pension of at least 1500€ per month must be put in place.<br>17. The government should be composed of an equal number of men and women.	7. Vliegen moet duurder worden door de tickets te belasten.<br>8. Ook na 2025 moeten we kerncentrales openhouden.<br>9. Als je rijdt mag je helemaal geen alcohol gedronken hebben.<br>10. Ook na 12 weken zwangerschap moet abortus nog toegestaan worden.<br>11. Spermadonatie mag niet langer anoniem gebeuren.<br>12. Grote vermogens moeten meer worden belast.<br>13. De lonen mogen niet meer automatisch aan de prijsstijgingen worden aangepast (index).<br>14. Vingerafdrukken van alle burgers moeten in een centrale databank worden bijgehouden.<br>15. Winkels mogen zelf kiezen wanneer ze solden doen.<br>16. Er moet een minimumpensioen komen van minstens €1500 euro per maand.<br>17. Er moeten in de regering evenveel mannen als vrouwen zitten.<br>18. Belangrijke politieke beslissingen moeten via een referendum aan burgers kunnen worden overgelaten.	l'essence ou au diesel doivent être interdites.<br>6. La TVA sur l'électricité doit être réduite de 21 à 6%.<br>7. Il faut taxer les billets d'avion pour qu'ils soient plus chers.<br>8. Les centrales nucléaires doivent rester opérationnelles après 2025.<br>9. On ne peut pas conduire en ayant bu de l'alcool.<br>10. L'avortement doit aussi être autorisé au-delà de 12 semaines de grossesse.<br>11. Le don de sperme ne doit plus pouvoir être anonyme.<br>12. Les grandes fortunes doivent être plus taxées.<br>13. Les salaires ne doivent plus être automatiquement indexés.<br>14. Les empreintes digitales de tous les citoyens doivent être conservées dans une base de données centrale.		

	18. Important political decisions must be handled by citizens via a referendum.		15. Les magasins doivent pouvoir choisir quand ils font leurs soldes.<br>16. Il faut instaurer une pension de retraite de 1500€ minimum par mois.<br>17. Le gouvernement doit compter autant d'hommes que de femmes.<br>18. Les décisions politiques importantes doivent pouvoir être laissées aux citoyens par le biais d'un référendum.		
[If region = Brussels] [If language = FR] w1_Perc_PartyPos_BXL-FR_1 to w1_Perc_PartyPos_BXL-FR_18	**For each of the following statements, could you indicate the parties that are, in your opinion, in favour of each statement? You can select several parties. [1 = cdH; 2 = DéFI; 4 = Ecolo; 5 = MR; 6 = PP; 7 = PS; 8 = PTB]**<br>1. Hosting transit migrants must be a punishable offence.<br>2. Situation tests must be put in place to detect discrimination in employment.<br>3. There must be a test on the european values in	**Kunt u voor elk beleidsvoorstel de partijen aanduiden waarvan u denkt dat ze het met het voorstel eens zijn? U kunt hier meerdere partijen aanklikken. [1 = cdH; 2 = DéFI; 4 = Ecolo; 5 = MR; 6 = PP; 7 = PS; 8 = PTB]**<br>1. Transmigranten onderdak bieden moet strafbaar zijn.<br>2. De overheid moet praktijktesten doen om te controleren op discriminatie bij aanwervingen.<br>3. Nieuwkomers moeten eerst slagen voor een examen over de Europese waarden voor ze de Belgische nationaliteit kunnen verwerven.	**Pour chaque proposition de politique, pouvez-vous indiquer les partis qui, selon vous, sont en accord avec la proposition? Vous pouvez sélectionner plusieurs partis. [1 = cdH; 2 = DéFI; 4 = Ecolo; 5 = MR; 6 = PP; 7 = PS; 8 = PTB]**<br>1. Héberger des migrants en transit doit être un délit punissable.<br>2. Il faut mettre en place des tests de situation pour détecter la discrimination à l'embauche.		

* The Dutch and French translations to not fully overlap.	order to obtain the belgian nationality.<br>4. If the request for asylum of families with children is rejected, these families can be placed in detention pending their repatriation.<br>5. By 2024, the company cars that run with petrol or diesel must be banned.<br>6. The VAT on electricity must be reduced from 21 to 6%.<br>7. There must be a tax on plane tickets in order to raise their price.<br>8. Nuclear power plants must remain operational after 2025.<br>9. We cannot drive while having drunk alcohol.<br>10. Abortion must be allowed beyond the 12th week of a pregnancy.<br>11. Sperm donation must no longer be anonymous.<br>12. Great fortunes must be more taxed.<br>13. Wages must no longer be automatically indexed.	4. Als de asielaanvraag van gezinnen met kinderen is afgewezen, mogen ze in afwachting van hun terugkeer worden opgesloten.<br>5. Bedrijfswagens die op benzine of diesel rijden moeten voor 2024 verboden worden.<br>6. De btw op elektriciteit moet verlaagd worden van 21 naar 6%.<br>7. Vliegen moet duurder worden door de tickets te belasten.<br>8. Ook na 2025 moeten we kerncentrales openhouden.<br>9. Als je rijdt mag je helemaal geen alcohol gedronken hebben.<br>10. Ook na 12 weken zwangerschap moet abortus nog toegestaan worden.<br>11. Spermadonatie mag niet langer anoniem gebeuren.<br>12. Grote vermogens moeten meer worden belast.<br>13. De lonen mogen niet meer automatisch aan de prijsstijgingen worden aangepast (index).<br>14. Vingerafdrukken van alle burgers moeten in een centrale databank worden bijgehouden.	3. Pour pouvoir obtenir la nationalité belge, il faut d'abord avoir réussi un examen sur les valeurs européennes.<br>4. Si la demande d'asile de familles avec enfants a été rejetée, ces familles peuvent être placées en détention en attendant leur rapatriement.<br>5. D'ici 2024, les voitures de société qui roulent à l'essence ou au diesel doivent être interdites.<br>6. La TVA sur l'électricité doit être réduite de 21 à 6%.<br>7. Il faut taxer les billets d'avion pour qu'ils soient plus chers.<br>8. Les centrales nucléaires doivent rester opérationnelles après 2025.<br>9. On ne peut pas conduire en ayant bu de l'alcool.<br>10. L'avortement doit aussi être autorisé au-delà de 12 semaines de grossesse.		

	14. The fingerprints of all citizens must be kept in a central database.<br>15. Shops must be able to choose when to do sales.<br>16. More workers should again be able to get an early pension. *<br>17. The government should be composed of an equal number of men and women.<br>18. Important political decisions must be handled by citizens via a referendum.	15. Winkels mogen zelf kiezen wanneer ze solden doen.<br>16. Meer werknemers moeten weer recht krijgen op brugpensioen. *<br>17. Er moeten in de regering evenveel mannen als vrouwen zitten.<br>18. Belangrijke politieke beslissingen moeten via een referendum aan burgers kunnen worden overgelaten.	11. Le don de sperme ne doit plus pouvoir être anonyme.<br>12. Les grandes fortunes doivent être plus taxées.<br>13. Les salaires ne doivent plus être automatiquement indexés.<br>14. Les empreintes digitales de tous les citoyens doivent être conservées dans une base de données centrale.<br>15. Les magasins doivent pouvoir choisir quand ils font leurs soldes.<br>16. Plus de travailleurs doivent à nouveau avoir le droit de prendre une pension anticipée. *<br>17. Le gouvernement doit compter autant d'hommes que de femmes.<br>18. Les décisions politiques importantes doivent pouvoir être laissées aux citoyens par le biais d'un référendum.		
[If region = Flanders] w1_Affective_Pol_VL_1	**Could you use the scale below to indicate how you feel about the following groups? [0-100 scale: 0**	**Kunt u de thermometer hier beneden gebruiken om aan te geven hoe u denkt over de volgende groepen? [0-100**	**Pouvez-vous utiliser l'échelle ci-dessous pour indiquer ce que vous pensez des groupes**	0	100

to w1_Affective_Pol_VL_9	to 50 = Not very favourable; 50 = Neutral; 50 to 100 = Favourable]<br>1. Walloons<br>2. Flemish<br>3. CD&V sympathisers<br>4. Groen sympathisers<br>5. N-VA sympathisers<br>6. Open VLD sympathisers<br>7. PvdA sympathisers<br>8. sp.a sympathisers<br>9. Vlaams Belang sympathisers	percentages: 0 to 50 = Niet gunstig gezind; 50 = Neutraal; 50 to 100 = Gunstig gezind]<br>1. Walen<br>2. Vlamingen<br>3. Aanhangers van CD&V<br>4. Aanhangers van Groen<br>5. Aanhangers van N-VA<br>6. Aanhangers van Open VLD<br>7. Aanhangers van PvdA<br>8. Aanhangers van sp.a<br>9. Aanhangers van Vlaams Belang	suivants? [0-100 percentages: 0 to 50 = Pas vraiment favorable; 50 = Neutre; 50 to 100 = Favorable]<br>1. Wallons<br>2. Flamands<br>3. Les sympathisants de CD&V<br>4. Les sympathisants de Groen<br>5. Les sympathisants de N-VA<br>6. Les sympathisants de Open VLD<br>7. Les sympathisants de PvdA<br>8. Les sympathisants de sp.a<br>9. Les sympathisants de Vlaams Belang		
[If region = Wallonia] w1_Affective_Pol_Wal_1 to w1_Affective_Pol_Wal_9	**Could you use the scale below to indicate how you feel about the following groups? [0-100 scale: 0 to 50 = Not very favourable; 50 = Neutral; 50 to 100 = Favourable]**<br>1. Walloons<br>2. Flemish<br>3. cdH sympathisers<br>4. DéFI sympathisers<br>5. Ecolo sympathisers<br>6. MR sympathisers<br>7. PP sympathisers<br>8. PS sympathisers<br>9. PTB sympathisers	**Kunt u de thermometer hier beneden gebruiken om aan te geven hoe u denkt over de volgende groepen? [0-100 percentages: 0 to 50 = Niet gunstig gezind; 50 = Neutraal; 50 to 100 = Gunstig gezind]**<br>1. Walen<br>2. Vlamingen<br>3. Aanhangers van cdH<br>4. Aanhangers van DéFI<br>5. Aanhangers van Ecolo<br>6. Aanhangers van MR<br>7. Aanhangers van PP<br>8. Aanhangers van PS	**Pouvez-vous utiliser l'échelle ci-dessous pour indiquer ce que vous pensez des groupes suivants? [0-100 percentages: 0 to 50 = Pas vraiment favorable; 50 = Neutre; 50 to 100 = Favorable]**<br>1. Wallons<br>2. Flamands<br>3. Les sympathisants de cdH<br>4. Les sympathisants de DéFI<br>5. Les sympathisants de Ecolo	0	100

		9. Aanhangers van PTB	6. Les sympathisants de MR<br>7. Les sympathisants de PP<br>8. Les sympathisants de PS<br>9. Les sympathisants de PTB		
[If region = Brussels] w1_Affective_Pol_BRU	**Could you use the scale below to indicate how you feel about the following groups? [0-100 scale: 0 to 50 = Not very favourable; 50 = Neutral; 50 to 100 = Favourable]**<br>1. [Affective_Pol_1] Walloons<br>2. [Affective_Pol_2] Flemish<br>3. [Affective_Pol_3] Brusseleers<br>4. [Affective_Pol_4] Sympathisers of left wing parties<br>5. [Affective_Pol_5] Sympathisers of right wing parties<br>6. [Affective_Pol_6] Sympathisers of dutch-speaking/flemish parties<br>7. [Affective_Pol_7] Sympathisers of french-speaking parties	**Kunt u de thermometer hier beneden gebruiken om aan te geven hoe u denkt over de volgende groepen? [0-100 percentages: 0 to 50 = Niet gunstig gezind; 50 = Neutraal; 50 to 100 = Gunstig gezind]**<br>1. [Affective_Pol_1] Walen<br>2. [Affective_Pol_2] Vlamingen<br>3. [Affective_Pol_3] Brusselaars<br>4. [Affective_Pol_4] Aanhangers van linkse partijen<br>5. [Affective_Pol_5] Aanhangers van rechtse partijen<br>6. [Affective_Pol_6] Aanhangers van Nederlandstalige partijen<br>7. [Affective_Pol_7] Aanhangers van Franstalige partijen	**Pouvez-vous utiliser l'échelle ci-dessous pour indiquer ce que vous pensez des groupes suivants? [0-100 percentages: 0 to 50 = Pas vraiment favorable; 50 = Neutre; 50 to 100 = Favorable]**<br>2. [Affective_Pol_1] Wallons<br>3. [Affective_Pol_2] Flamands<br>4. [Affective_Pol_3] Bruxellois<br>5. [Affective_Pol_4] Les sympathisants des partis de gauche<br>6. [Affective_Pol_5] Les sympathisants des partis de droite<br>7. [Affective_Pol_6] Les sympathisants des partis néerlandophones/flamands<br>8. [Affective_Pol_7] Les sympathisants des partis francophones	0	100

4.2 Wave 2 questionnaire

Variable name	Label + Values [English]	Label + Values [Dutch]	Label + Values [French]	Min	Max
w2_Region	**In which region is your main residence located?**<br>1. Brussels Capital Region<br>2. Flemish Region<br>3. Walloon Region	**In welk gewest heeft u uw hoofdverblijfplaats?**<br>1. Brussels Hoofdstedelijk Gewest<br>2. Vlaams Gewest<br>3. Waals Gewest	**Dans quelle région se trouve votre lieu de résidence principale ?**<br>1. Région de Bruxelles-Capitale<br>2. Région flamande<br>3. Région wallonne	1	3
w2_Duration	**Duration of respondent's time to complete the questionnaire of wave 2 (in seconds)**			148	1009016
w2_RecordedDate	**Date on which the respondent's response for wave 2 was recorded**			28 May 2019	18 June 2019
w2_Q_Language	**Language setting for the respondent in wave 2**<br>0. French<br>1. Dutch			0	1
w2_Consent	**Did respondent agree with the informed consent form?**<br>1. Yes<br>2. No			1	1
w2_Age	**What is your age?**	**Wat is uw leeftijd?**	**Quel âge avez-vous?**	18	110
[If region = Flanders]<br>w2_VoteFed19_Fl	**For which party did you vote for the Chamber during the national elections on the 26th of May 2019?**<br>1. CD&V<br>2. Groen<br>3. N-VA<br>4. Open VLD<br>5. PVDA<br>6. sp.a<br>7. Vlaams Belang<br>8. Other<br>9. Blank or invalid	**Op welke partij stemde u voor de Kamer bij de afgelopen verkiezingen van 26 mei 2019?**<br>1. CD&V<br>2. Groen<br>3. N-VA<br>4. Open VLD<br>5. PVDA<br>6. sp.a<br>7. Vlaams Belang<br>8. Andere<br>9. Blanco of Ongeldig gestemd<br>10. Niet gaan stemmen	**Pour quel parti avez-vous voté à la Chambre des Représentants lors des élections fédérales du 26 mai 2019 ?**<br>1. CD&V<br>2. Groen<br>3. N-VA<br>4. Open VLD<br>5. PVDA<br>6. sp.a<br>7. Vlaams Belang<br>8. Autre<br>9. J'ai voté blanc / invalide	1	12

	10. Did not vote<br>11. Was not (yet) eligible to vote<br>12. I do not remember	11. Mocht (nog) niet stemmen<br>12. Ik weet het niet meer	10. Je n'ai pas voté<br>11. Je ne pouvais pas (encore) voter<br>12. Je ne m'en rappelle pas		
[If region = Wallonia]<br>w2_VoteFed19_Wal	**For which party did you vote for the Chamber during the national elections on the 26th of May 2019?**<br>1. cdH<br>2. Ecolo<br>3. DéFI<br>4. PP<br>5. MR<br>6. PS<br>7. PTB<br>8. Other<br>9. Blank or invalid<br>10. I did not vote<br>11. I could not (yet) vote<br>12. I do not remember	**Op welke partij stemde u voor de Kamer bij de afgelopen verkiezingen van 26 mei 2019?**<br>1. cdH<br>2. Ecolo<br>3. DéFI<br>4. PP<br>5. MR<br>6. PS<br>7. PTB<br>8. Andere<br>9. Blanco of Ongeldig gestemd<br>10. Niet gaan stemmen<br>11. Mocht (nog) niet stemmen<br>12. Ik weet het niet meer	**Pour quel parti avez-vous voté à la Chambre des Représentants lors des élections fédérales du 26 mai 2019 ?**<br>1. cdH<br>2. Ecolo<br>3. DéFI<br>4. PP<br>5. MR<br>6. PS<br>7. PTB<br>8. Autre<br>9. J'ai voté blanc / invalide<br>10. Je n'ai pas voté<br>11. Je ne pouvais pas (encore) voter<br>12. Je ne m'en rappelle pas	1	12

[If region = Brussels] w2_VoteFed19_Bxl	**For which party did you vote for the Chamber during the national elections on the 26th of May 2019?**<br>1. CD&V<br>2. cdH<br>3. DéFI<br>4. Ecolo-Groen<br>5. MR<br>6. N-VA<br>7. Open VLD<br>8. PP<br>9. PS<br>10. PTB-PVDA<br>11. sp.a<br>12. Vlaams Belang<br>13. Other<br>14. Blank or invalid<br>15. I did not vote<br>16. I could not (yet) vote<br>17. I do not remember	**Op welke partij stemde u voor de Kamer bij de afgelopen verkiezingen van 26 mei 2019?**<br>1. CD&V<br>2. cdH<br>3. DéFI<br>4. Ecolo-Groen<br>5. MR<br>6. N-VA<br>7. Open VLD<br>8. PP<br>9. PS<br>10. PTB-PVDA<br>11. sp.a<br>12. Vlaams Belang<br>13. Andere<br>14. Blanco of Ongeldig gestemd<br>15. Niet gaan stemmen<br>16. Mocht (nog) niet stemmen<br>17. Ik weet het niet meer	**Pour quel parti avez-vous voté à la Chambre des Représentants lors des élections fédérales du 26 mai 2019 ?**<br>1. CD&V<br>2. cdH<br>3. DéFI<br>4. Ecolo-Groen<br>5. MR<br>6. N-VA<br>7. Open VLD<br>8. PP<br>9. PS<br>10. PTB-PVDA<br>11. sp.a<br>12. Vlaams Belang<br>13. Autre<br>14. J'ai voté blanc / invalide<br>15. Je n'ai pas voté<br>16. Je ne pouvais pas (encore) voter<br>17. Je ne m'en rappelle pas	1	17
[If VoteFed19 is not blank or invalid, 'I did not vote', 'I could not (yet) vote' or 'I do not remember'] w2_Timing_vote_decision	**When did you decide for which party you were going to vote for the Chamber?**<br>1. On the day of the elections<br>2. During the campaign<br>3. Before the beginning of the campaign	**Wanneer heeft u beslist op welke partij u zou stemmen voor de Kamer?**<br>1. De dag van de verkiezingen zelf<br>2. Tijdens de campagne<br>3. Voor de start van de campagne	**Quand avez-vous décidé pour quel parti voter à la Chambre ?**<br>1. Le jour des élections<br>2. Pendant la campagne<br>3. Avant le début de la campagne	1	3
[If VoteFed19 is not blank or invalid, 'I did not vote', 'I could not	**Did you vote for a list or for one or several candidates on a list?**<br>1. I voted for a list	**Heeft u een lijststem uitgebracht of heeft u voor één of meerdere kandidaten op een lijst gestemd?**	**Avez-vous voté pour une liste ou avez-vous voté pour un ou plusieurs candidats sur une liste ?**	1	3

(yet) vote' or 'I do not remember']<br>w2_Pref_vote	2. I voted for one or several candidates<br>3. I do not remember	1. Ik heb gestemd voor de lijst<br>2. Ik heb gestemd op één of meerdere kandidaten<br>3. Ik weet het niet meer	1. J'ai voté pour une liste<br>2. J'ai voté pour un ou plusieurs candidats<br>3. Je ne m'en rappelle pas		
[If region = Flanders]<br>w2_VoteReg19_Fl	**For which party did you vote for the Flemish Parliament during the regional elections of the 26th of May 2019?**<br>1. CD&V<br>2. Groen<br>3. N-VA<br>4. Open VLD<br>5. PVDA<br>6. sp.a<br>7. Vlaams Belang<br>8. Other<br>9. Blank or invalid<br>10. I did not vote<br>11. Cannot vote (yet)<br>12. I do not remember	**Op welke partij stemde u voor het Vlaamse parlement bij de afgelopen regionale verkiezingen van 26 mei 2019?**<br>1. CD&V<br>2. Groen<br>3. N-VA<br>4. Open VLD<br>5. PVDA<br>6. sp.a<br>7. Vlaams Belang<br>8. Andere<br>9. Blanco of ongeldig gestemd<br>10. Niet gaan stemmen<br>11. Mag (nog) niet stemmen<br>12. Ik weet het niet meer	**Pour quel parti avez-vous voté au Parlement flamand lors des dernières élections régionales du 26 mai 2019 ?**<br>1. CD&V<br>2. Groen<br>3. N-VA<br>4. Open VLD<br>5. PVDA<br>6. sp.a<br>7. Vlaams Belang<br>8. Autre<br>9. Blanc ou invalide<br>10. Je n'ai pas voté<br>11. Ne peut pas (encore) voter<br>12. Je ne m'en rappelle pas	1	12
[If region = Wallonia]<br>w2_VoteReg19_Wal	**For which party did you vote for the Walloon Parliament during the regional elections of the 26th of May 2019?**<br>1. cdH<br>2. Ecolo<br>3. DéFI<br>4. PP<br>5. MR<br>6. PS<br>7. PTB<br>8. Other	**Op welke partij stemde u voor het Waalse parlement bij de afgelopen regionale verkiezingen van 26 mei 2019?**<br>1. cdH<br>2. Ecolo<br>3. DéFI<br>4. PP<br>5. MR<br>6. PS<br>7. PTB<br>8. Andere	**Pour quel parti avez-vous voté au Parlement wallon lors des dernières élections régionales du 26 mai 2019 ?**<br>1. cdH<br>2. Ecolo<br>3. DéFI<br>4. PP<br>5. MR<br>6. PS<br>7. PTB<br>8. Autre	1	12

	9. Blank or invalid<br>10. I did not vote<br>11. Cannot vote (yet)<br>12. I do not remember	9. Blanco of ongeldig gestemd<br>10. Niet gaan stemmen<br>11. Mag (nog) niet stemmen<br>12. Ik weet het niet meer	9. Blanc ou invalide<br>10. Je n'ai pas voté<br>11. Ne peut pas (encore) voter<br>12. Je ne m'en rappelle pas		
[If region = Brussels]<br>w2_VoteReg19_Bxl	**For which party did you vote for the Parliament of Brussels during the regional elections of the 26th of May 2019?**<br>1. CD&V<br>2. CDH<br>3. DéFI<br>4. Ecolo<br>5. Groen<br>6. MR<br>7. N-VA<br>8. Open VLD<br>9. PP<br>10. PS<br>11. PTB<br>12. PVDA<br>13. sp.a<br>14. Vlaams Belang<br>15. Other<br>16. Blank or invalid<br>17. I did not vote<br>18. Cannot vote (yet)<br>19. I do not remember	**Op welke partij stemde u voor het Brusselse parlement bij de afgelopen regionale verkiezingen van 26 mei 2019?**<br>1. CD&V<br>2. CDH<br>3. DéFI<br>4. Ecolo<br>5. Groen<br>6. MR<br>7. N-VA<br>8. Open VLD<br>9. PP<br>10. PS<br>11. PTB<br>12. PVDA<br>13. sp.a<br>14. Vlaams Belang<br>15. Andere<br>16. Blanco of ongeldig gestemd<br>17. Niet gaan stemmen<br>18. Mag (nog) niet stemmen<br>19. Ik weet het niet meer	**Pour quel parti avez-vous voté au Parlement bruxellois lors des dernières élections régionales du 26 mai 2019 ?**<br>1. CD&V<br>2. CDH<br>3. DéFI<br>4. Ecolo<br>5. Groen<br>6. MR<br>7. N-VA<br>8. Open VLD<br>9. PP<br>10. PS<br>11. PTB<br>12. PVDA<br>13. sp.a<br>14. Vlaams Belang<br>15. Autre<br>16. Blanc ou invalide<br>17. Je n'ai pas voté<br>18. Ne peut pas (encore) voter<br>19. Je ne m'en rappelle pas	1	19
[If region = Flanders]<br>w2_VoteEU19_Fl	**For which party did you vote for the European Parliament during the european elections on the 26th of May 2019?**	**En op welke partij stemde u voor het Europees parlement bij de afgelopen Europese verkiezingen van 26 mei 2019?**	**Pour quel parti avez-vous voté au Parlement européen lors des dernières élections européennes du 26 mai 2019 ?**	1	12

	1. CD&V<br>2. Groen<br>3. N-VA<br>4. Open VLD<br>5. PVDA<br>6. sp.a<br>7. Vlaams Belang<br>8. Other<br>9. Blank or invalid<br>10. I did not vote<br>11. Cannot vote (yet)<br>12. I do not remember	1. CD&V<br>2. Groen<br>3. N-VA<br>4. Open VLD<br>5. PVDA<br>6. sp.a<br>7. Vlaams Belang<br>8. Andere<br>9. Blanco of ongeldig gestemd<br>10. Niet gaan stemmen<br>11. Mag (nog) niet stemmen<br>12. Ik weet het niet meer	1. CD&V<br>2. Groen<br>3. N-VA<br>4. Open VLD<br>5. PVDA<br>6. sp.a<br>7. Vlaams Belang<br>8. Autre<br>9. Blanc ou invalide<br>10. Je n'ai pas voté<br>11. Ne peut pas (encore) voter<br>12. Je ne m'en rappelle pas		
[If region = Wallonia]<br>w2_VoteEU19_Wal	**For which party did you vote for the European Parliament during the european elections on the 26th of May 2019?**<br>1. cdH<br>2. Ecolo<br>3. DéFI<br>4. PP<br>5. MR<br>6. PS<br>7. PTB<br>8. Other<br>9. Blank or invalid<br>10. I did not vote<br>11. Cannot vote (yet)<br>12. I do not remember	**En op welke partij stemde u voor het Europees parlement bij de afgelopen Europese verkiezingen van 26 mei 2019?**<br>1. cdH<br>2. Ecolo<br>3. DéFI<br>4. PP<br>5. MR<br>6. PS<br>7. PTB<br>8. Andere<br>9. Blanco of ongeldig gestemd<br>10. Niet gaan stemmen<br>11. Mag (nog) niet stemmen<br>12. Ik weet het niet meer	**Pour quel parti avez-vous voté au Parlement européen lors des dernières élections européennes du 26 mai 2019 ?**<br>1. cdH<br>2. Ecolo<br>3. DéFI<br>4. PP<br>5. MR<br>6. PS<br>7. PTB<br>8. Autre<br>9. Blanc ou invalide<br>10. Je n'ai pas voté<br>11. Ne peut pas (encore) voter<br>12. Je ne m'en rappelle pas	1	12

[If region = Brussels] w2_VoteEU19_Bxl	**For which party did you vote for the European Parliament during the european elections on the 26th of May 2019?**<br>1. CD&V<br>2. cdH<br>3. DéFI<br>4. Ecolo<br>5. Groen<br>6. MR<br>7. N-VA<br>8. Open VLD<br>9. PP<br>10. PS<br>11. PTB<br>12. PVDA<br>13. sp.a<br>14. Vlaams Belang<br>15. Other<br>16. Blank or invalid<br>17. I did not vote<br>18. Cannot vote (yet)<br>19. I do not remember	**En op welke partij stemde u voor het Europees parlement bij de afgelopen Europese verkiezingen van 26 mei 2019?**<br>1. CD&V<br>2. cdH<br>3. DéFI<br>4. Ecolo<br>5. Groen<br>6. MR<br>7. N-VA<br>8. Open VLD<br>9. PP<br>10. PS<br>11. PTB<br>12. PVDA<br>13. sp.a<br>14. Vlaams Belang<br>15. Andere<br>16. Blanco of ongeldig gestemd<br>17. Niet gaan stemmen<br>18. Mag (nog) niet stemmen<br>19. Ik weet het niet meer	**Pour quel parti avez-vous voté au Parlement européen lors des dernières élections européennes du 26 mai 2019 ?**<br>1. CD&V<br>2. cdH<br>3. DéFI<br>4. Ecolo<br>5. Groen<br>6. MR<br>7. N-VA<br>8. Open VLD<br>9. PP<br>10. PS<br>11. PTB<br>12. PVDA<br>13. sp.a<br>14. Vlaams Belang<br>15. Autre<br>16. Blanc ou invalide<br>17. Je n'ai pas voté<br>18. Ne peut pas (encore) voter<br>19. Je ne m'en rappelle pas	1	19
[If region = Flanders] w2_ElecUtil_Fl_1 to w2_ElecUtil_Fl_7	**There are several political parties that are active in Flanders. Could you indicate the extent to which it is likely that you will one day vote for each of the following parties in upcoming elections? [0-10 scale: 0 = Very unlikely; 10 = Very likely]**<br>1. CD&V	**In Vlaanderen zijn verschillende politieke partijen actief. Kunt u aangeven hoe waarschijnlijk het is dat u in toekomstige verkiezingen ooit voor de onderstaande partijen zou stemmen? [0-10 scale: 0 = Heel onwaarschijnlijk; 10 = Heel waarschijnlijk]**<br>1. CD&V	**Différents partis politiques sont actifs en Flandre. Pouvez-vous indiquer dans quelle mesure il est probable que vous voterez un jour pour les partis suivants lors de prochaines élections ? [0-10 scale: 0 = Peu probable; 10 = Très probable]**<br>1. CD&V	0	10

	2. Groen<br>3. N-VA<br>4. Open VLD<br>5. PvdA<br>6. sp.a<br>7. Vlaams Belang	2. Groen<br>3. N-VA<br>4. Open VLD<br>5. PvdA<br>6. sp.a<br>7. Vlaams Belang	2. Groen<br>3. N-VA<br>4. Open VLD<br>5. PvdA<br>6. sp.a<br>7. Vlaams Belang		
[If region = Brussels] w2_Elec_Ut_BXL_1 to w2_Elec_Ut_BXL_14	**There are several political parties that are active in the Brussels Capital Region. Could you indicate the extent to which it is likely that you will one day vote for each of the following parties in upcoming elections? [0-10 scale: 0 = Very unlikely; 10 = Very likely]**<br>1. CD&V<br>2. cdH<br>3. DéFI<br>4. Ecolo<br>5. Groen<br>6. MR<br>7. N-VA<br>8. Open VLD<br>9. PP<br>10. PS<br>11. PTB<br>12. PvdA<br>13. sp.a<br>14. Vlaams Belang	**In het Brussels Hoofdstedelijk Gewest zijn verschillende politieke partijen actief. Kunt u aangeven hoe waarschijnlijk het is dat u in toekomstige verkiezingen ooit voor de onderstaande partijen zou stemmen? [0-10 scale: 0 = Heel onwaarschijnlijk; 10 = Heel waarschijnlijk]**<br>1. CD&V<br>2. cdH<br>3. DéFI<br>4. Ecolo<br>5. Groen<br>6. MR<br>7. N-VA<br>8. Open VLD<br>9. PP<br>10. PS<br>11. PTB<br>12. PvdA<br>13. sp.a<br>14. Vlaams Belang	**Différents partis politiques sont actifs dans la Région de Bruxelles-Capitale. Pouvez-vous indiquer dans quelle mesure il est probable que vous voterez un jour pour les partis suivants lors de prochaines élections ? [0-10 scale: 0 = Peu probable; 10 = Très probable]**<br>1. CD&V<br>2. cdH<br>3. DéFI<br>4. Ecolo<br>5. Groen<br>6. MR<br>7. N-VA<br>8. Open VLD<br>9. PP<br>10. PS<br>11. PTB<br>12. PvdA<br>13. sp.a<br>14. Vlaams Belang	0	10
[If region = Wallonia] w2_ElecUtil_Wal_1 to w2_ElecUtil_Wal_7	**There are several political parties that are active in Wallonia. Could you indicate the extent to which it is likely that you will one day**	**In Wallonië zijn verschillende politieke partijen actief. Kunt u aangeven hoe waarschijnlijk het is dat u in toekomstige verkiezingen ooit voor de**	**Différents partis politiques sont actifs en Wallonie. Pouvez-vous indiquer dans quelle mesure il est probable que vous voterez un**	0	10

	**vote for each of the following parties in upcoming elections? [0-10 scale: 0 = Very unlikely; 10 = Very likely]**<br>1. cdH<br>2. DéFI<br>3. Ecolo<br>4. PP<br>5. MR<br>6. PS<br>7. PTB	**onderstaande partijen zou stemmen? [0-10 scale: 0 = Heel onwaarschijnlijk; 10 = Heel waarschijnlijk]**<br>1. cdH<br>2. DéFI<br>3. Ecolo<br>4. PP<br>5. MR<br>6. PS<br>7. PTB	**jour pour les partis suivants lors de prochaines élections ? [0-10 scale: 0 = Peu probable; 10 = Très probable]**<br>1. cdH<br>2. DéFI<br>3. Ecolo<br>4. PP<br>5. MR<br>6. PS<br>7. PTB		
w2_Vote_comp_1 to w2_Vote_comp_4	**If voting was no longer mandatory in Belgium, would you still vote for the following types of elections? [1 = Never; 2 = Rarely; 3 = Seldom; 4 = Always]**<br>1. The European Parliament elections<br>2. The Belgian federal elections<br>3. The regional elections (Flanders, Wallonia, Brussels)<br>4. The local elections	**Als stemmen niet meer verplicht was in België, zou u dan nog gaan stemmen voor elk van de volgende soorten verkiezingen? [1 = Nooit; 2 = Soms; 3 = Meestal; 4 = Altijd]**<br>1. De verkiezingen voor het Europees Parlement<br>2. De Belgische federale verkiezingen<br>3. De regionale verkiezingen (Vlaams, Waals en Brussels parlement)<br>4. De gemeenteraadsverkiezingen	**Si le vote n'était plus obligatoire en Belgique, voteriez-vous encore pour chacun des types d'élections suivants ? [1 = Jamais; 2 = Parfois; 3 = Souvent; 4 = Toujours]**<br>1. Les élections du Parlement européen<br>2. Les élections fédérales belges<br>3. Les élections régionales (Flandre, Wallonie, Bruxelles)<br>4. Les élections communales	1	4
w2_Camp_Inten	**How intensively did you follow the last electoral campaign?**<br>1. Very intensively<br>2. Intensively<br>3. Not very intensively<br>4. Not at all	**Hoe intensief heeft u de voorbije verkiezingscampagne gevolgd?**<br>1. Zeer intensief<br>2. Intensief<br>3. Niet zo intensief<br>4. In het geheel niet	**Avec quelle intensité avez-vous suivi la dernière campagne électorale ?**<br>1. Très intensive<br>2. Intensive<br>3. Pas très intensive<br>4. Pas du tout	1	4

w2_Camp_expos_1 to w2_Camp_expos_7	**For each of the following channels, how many times did you see information about politics in the past month? [1 = Never; 2 = Less than once per week; 3 = 2-3 times per week; 4 = 4-5 times per week; 5 = 6-7 times per week]**<br>1. Television<br>2. News websites<br>3. Newspapers (press and online)<br>4. Social media<br>5. Posters of political parties<br>6. Advertisements of parties or candidates (in the press, social media and/or by post)<br>7. Political parties' websites	**Hoe frequent heeft u in de afgelopen maand informatie over politiek gezien via elk van de volgende kanalen? [1 = Nooit; 2 = Minder dan 1 dag per week; 3 = 2-3 dagen per week; 4 = 4-5 dagen per week; 5 = 6-7 dagen per week]**<br>1. Televisie<br>2. Nieuwswebsites<br>3. Kranten (papieren versie of online)<br>4. Sociale media<br>5. Posters van politieke partijen of kandidaten<br>6. Advertenties van partijen of kandidaten (in pers, sociale media en/of brievenbus)<br>7. Partijwebsites	**Combien de fois avez-vous vu des informations sur la politique au cours du dernier mois pour chacun des canaux suivants ? [1 = Jamais; 2 = Moins que 1 fois par semaine; 3 = 2-3 fois par semaine; 4 = 4-5 fois par semaine; 5 = 6-7 fois par semaine]**<br>1. Télévision<br>2. Sites d'actualités<br>3. Journaux (version papier ou en ligne)<br>4. Médias sociaux<br>5. Affiches de partis politiques ou de candidats<br>6. Annonces de partis ou de candidats (dans la presse, les médias sociaux et/ou dans la boîte aux lettres)<br>7. Sites web des partis	1	5
w2_Issue_salience_1 [First issue] to w2_Issue_salience_2 [second issue]	**Could you indicate which were the most important issues that determined your vote during the past elections? You can indicate a maximum of two issues. [1 = Employment; 2 = Environment; 3 = Crime; 4 - Migration; 5 = Economy; 6 = State reforms; 7 = Defence; 8 = Taxation; 9 = Social**	**Kunt u aangeven welke twee thema's het belangrijkst waren bij het maken van uw stemkeuze bij de verkiezingen? U kunt maximaal twee thema's aanduiden. [1 = Werkgelegenheid; 2 = Milieu; 3 = Criminaliteit; 4 = Migratie; 5 = Economie; 6 = Staatshervormingen; 7 = Defensie; 8 = Belastingen; 9 = Sociale zekerheid; 10 = Functioneren van de democratie]**	**Pourriez-vous indiquer quelles sont les deux thématiques qui ont été les plus importantes dans votre choix de vote lors des élections ? Vous pouvez indiquer un maximum de deux thèmes. [1 = Emploi; 2 = Environnement; 3 = Crime; 4 = Migration; 5 = Economie; 6 = Réformes de l'État; 7 = Défense; 8 = Fiscalité; 9 =**	1	10

	**security; 10 = Functioning of democracy]**		**Sécurité sociale; 10 = Fonctionnement de la démocratie]**		
[If region = Flanders] w2_IO_FLA_1 to w2_IO_FLA_8	**When you think of the following issues, which party do you think of first? [1 = CD&V; 2 = Groen; 3 = N-VA; 4 = Open VLD; 5 = PVDA; 6 = sp.a; 7 = Vlaams Belang; 8 = All the parties equally; 9 = None of the parties]**<br>1. Employment<br>2. Environment<br>3. Criminality<br>4. Migration<br>5. State reforms<br>6. Defence<br>7. Taxation<br>8. Social security	**Wanneer u denkt aan de volgende beleidsthema's, aan welke partij denkt u dan het eerst spontaan? Dit is niet per se de partij waar u het op dat thema ook mee eens bent. [1 = CD&V; 2 = Groen; 3 = N-VA; 4 = Open VLD; 5 = PVDA; 6 = sp.a; 7 = Vlaams Belang; 8 = Alle partijen gelijk; 9 = Geen enkele partij]**<br>1. Werkgelegenheid<br>2. Milieu<br>3. Criminaliteit<br>4. Migratie<br>5. Staatshervorming<br>6. Defensie<br>7. Belastingen<br>8. Sociale zekerheid	**Quand vous pensez aux thèmes politiques suivants, à quel parti pensez-vous spontanément en premier ? Ce n'est pas nécessairement le parti avec lequel vous êtes d'accord sur ce thème. [1 = CD&V; 2 = Groen; 3 = N-VA; 4 = Open VLD; 5 = PVDA; 6 = sp.a; 7 = Vlaams Belang; 8 = À tous les partis de manière égale; 9 = À aucun parti]**<br>1. Emploi<br>2. Environnement<br>3. Criminalité<br>4. Migration<br>5. Réforme d’Etat<br>6. Defense<br>7. Impôts<br>8. Sécurité sociale	1	9
[If region = Wallonia] w2_IO_WAL_1 to w2_IO_WAL_8	**When you think of the following issues, which party do you think of first? [1 = cdH; 2 = DéFI; 3 = Ecolo; 4 = MR; 5 = PP; 6 = PS; 7 = PTB; 8 = All the parties equally; 9 = None of the parties]**<br>1. Employment<br>2. Environment<br>3. Criminality<br>4. Migration<br>5. State reforms	**Wanneer u denkt aan de volgende beleidsthema's, aan welke partij denkt u dan het eerst spontaan? Dit is niet per se de partij waar u het op dat thema ook mee eens bent. [1 = cdH; 2 = DéFI; 3 = Ecolo; 4 = MR; 5 = PP; 6 = PS; 7 = PTB; 8 = Alle partijen gelijk; 9 = Geen enkele partij]**<br>1. Werkgelegenheid<br>2. Milieu<br>3. Criminaliteit	**Quand vous pensez aux thèmes politiques suivants, à quel parti pensez-vous spontanément en premier ? Ce n'est pas nécessairement le parti avec lequel vous êtes d'accord sur ce thème. [1 = cdH; 2 = Ecolo; 3 = DéFI; 4 = PP; 5 = MR; 6 = PS; 7 = PTB; 8 = À tous les partis de manière égale; 9 = À aucun parti]**<br>1. Emploi	1	9

	6. Defence<br>7. Taxation<br>8. Social security	4. Migratie<br>5. Staatshervorming<br>6. Defensie<br>7. Belastingen<br>8. Sociale zekerheid	2. Environnement<br>3. Criminalité<br>4. Migration<br>5. Réforme d'Etat<br>6. Defense<br>7. Impôts<br>8. Sécurité sociale		
[If region = Brussels] w2_IO_BXL_1 to w2_IO_BXL_8	**When you think of the following issues, which party do you think of first? [1 = CD&V; 2 = cdH; 3 = DéFI; 4 = Ecolo; 5 = Groen; 6 = MR; 7 = N-VA; 8 = Open VLD; 9 = PP; 10 = PS; 11 = PTB; 12 = PVDA; 13 = sp.a; 14 = Vlaams Belang; 15 = All the parties equally; 16 = Non of the parties]**<br>1. Employment<br>2. Environment<br>3. Criminality<br>4. Migration<br>5. State reforms<br>6. Defence<br>7. Taxation<br>8. Social security	**Wanneer u denkt aan de volgende beleidsthema's, aan welke partij denkt u dan het eerst spontaan? Dit is niet per se de partij waar u het op dat thema ook mee eens bent. [1 = CD&V; 2 = cdH; 3 = DéFI; 4 = Ecolo; 5 = Groen; 6 = MR; 7 = N-VA; 8 = Open VLD; 9 = PP; 10 = PS; 11 = PTB; 12 = PVDA; 13 = sp.a; 14 = Vlaams Belang; 15 = Alle partijen gelijk; 16 = Geen enkele partij]**<br>1. Werkgelegenheid<br>2. Milieu<br>3. Criminaliteit<br>4. Migratie<br>5. Staatshervorming<br>6. Defensie<br>7. Belastingen<br>8. Sociale zekerheid	**Quand vous pensez aux thèmes politiques suivants, à quel parti pensez-vous spontanément en premier ? Ce n'est pas nécessairement le parti avec lequel vous êtes d'accord sur ce thème. [1 = CD&V; 2 = cdH; 3 = DéFI; 4 = Ecolo; 5 = Groen; 6 = MR; 7 = N-VA; 8 = Open VLD; 9 = PP; 10 = PS; 11 = PTB; 12 = PVDA; 13 = sp.a; 14 = Vlaams Belang; 15 = À tous les partis de manière égale; 16 = À aucun parti]**<br>1. Emploi<br>2. Environnement<br>3. Criminalité<br>4. Migration<br>5. Réforme d'Etat<br>6. Defense<br>7. Impôts<br>8. Sécurité sociale	1	9
w2_Sat_dem	**Overall, how satisfied are you with the way democracy is working in Belgium?**<br>1. Very satisfied<br>2. Somewhat satisfied	**In het algemeen, bent u eerder tevreden of eerder ontevreden over de manier waarop de democratie in België werkt?**<br>1. Heel tevreden	**En général, êtes-vous plutôt satisfait ou plutôt insatisfait de la façon dont la démocratie fonctionne en Belgique?**<br>1. Très satisfait	1	5

	3. Not satisfied, nor unsatisfied<br>4. Somewhat unsatisfied<br>5. Very unsatisfied	2. Eerder tevreden<br>3. Noch tevreden, noch ontevreden<br>4. Eerder ontevreden<br>5. Heel ontevreden	2. Plutôt satisfait<br>3. Ni satisfait, ni insatisfait<br>4. Plutôt insatisfait<br>5. Très insatisfait		
w2_Estim_voters _1 to w2_Estim_voters _3	**According to you, how many Flemings/Walloons/Brussels residents are satisfied and unsatisfied with the functioning of democracy in Belgium? [0-100 percentages]**<br>1. % Satisfied<br>2. % Not satisfied, nor unsatisfied<br>3. % Unsatisfied	**Volgens u, hoeveel % van de Vlamingen/Walen/Brusselaars zijn ontevreden en tevreden over het functioneren van de democratie in België? [0-100 percentages]**<br>1. % Tevreden<br>2. %Tevreden noch ontevreden<br>3. % Ontevreden	**Selon vous, combien de Flamands/Wallons/Bruxellois sont mécontents et combien sont satisfaits du fonctionnement de la démocratie en Belgique? [0-100 percentages]**<br>1. % Satisfait<br>2. % Ni satisfait, ni insatisfait<br>3. % Insatisfait	0	100
w2_Feel_Repres_1 to w2_Feel_Repres_3	**Could you indicate the extent to which you agree or disagree with the following statements? [0-10 scale: 0 = Totally disagree; 10 = Totally agree]**<br>1. I believe that there is a political party and/or politician that shares my opinions<br>2. There is not one political party and/or politician that properly represents my interests in general.<br>3. I find that some of my main concerns are addressed in the programme of a political party.	**Kunt u aangeven in hoeverre u het eens bent met onderstaande stellingen? [0-10 scale: 0 = Helemaal oneens; 10 = Helemaal eens]**<br>1. Ik heb het gevoel dat er een politieke partij en/of politicus is die een groot deel van mijn opinies deelt.<br>2. Er is geen enkele politieke partij en/of politicus die mijn belangen in het algemeen goed vertegenwoordigt.<br>3. Een aantal zaken die ik echt belangrijk vind, vind ik terug in het programma van een politieke partij.	**Pouvez-vous indiquer dans quelle mesure vous êtes en accord avec les affirmations suivantes? [0-10 scale: 0 = Pas du tout d'accord; 10 = Tout à fait d'accord]**<br>1. Je pense qu'il y a un parti politique et/ou un homme ou une femme politique qui partage une grande partie de mes opinions.<br>2. Il n'y a pas un seul parti politique et/ou un homme ou une femme politique qui bien représenté mes intérêts en général.	0	10

			3. Je retrouve une partie de mes préoccupations les plus importantes dans le programme d'un parti politique.		
w2_Emo_Gen_Pol_1 to w2_Emo_Gen_Pol_8	**When you think of Belgian politics in general, to what extent do you feel each of the following emotions? [0-10 scale: 0 = Not at all; 10 = To a great extent]**<br>1. Anger<br>2. Bitterness<br>3. Anxiety<br>4. Fear<br>5. Hope<br>6. Relief<br>7. Happiness<br>8. Contentment	**Als u denkt aan de Belgische politiek in het algemeen, in welke mate ervaart u dan elk van de onderstaande emoties? [0-10 scale: 0 = Helemaal niet; 10 = In sterke mate]**<br>1. Boosheid<br>2. Bitterheid<br>3. Ongerustheid<br>4. Angst<br>5. Hoop<br>6. Opluchting<br>7. Blijdschap<br>8. Tevredenheid	**Lorsque vous pensez à la politique belge en général, dans quelle mesure ressentez-vous chacune des émotions suivantes? [0-10 scale: 0 = Pas du tout; 10 = Dans une grande mesure]**<br>1. La colère<br>2. l'amertume<br>3. L'inquiétude<br>4. la peur<br>5. L'espoir<br>6. Soulagement<br>7. La joie<br>8. La satisfaction	0	10
w2_Cynisme_1 to w2_Cynisme_7	**Please indicate to what extent you disagree or agree with the following statements. [1 = Totally disagree; 2 = Somewhat disagree; 3 = Neither agree nor disagree; 4 = Somewhat agree; 5 = Totally agree]**<br>1. Politicians are corrupt<br>2. Most politicians are competent.<br>3. Politicians try to keep their promises.	**Kunt u aangeven in hoeverre u het eens bent met onderstaande stellingen? [1 = Helemaal oneens; 2 = Eerder oneens; 3 = Noch eens, noch oneens; 4 = Eerder eens; 5 = Helemaal eens]**<br>1. Politici zijn corrupt.<br>2. De meeste politici zijn competent.<br>3. Politici proberen hun beloftes te houden.<br>4. Politici begrijpen niet wat er speelt in de samenleving.<br>5. Veel politici draaien al te lang mee.	**Pouvez-vous indiquer dans quelle mesure vous êtes d'accord avec les affirmations ci-dessous? [1 = Pas du tout d'accord; 2 = Plutôt pas d'accord; 3 = Ni d'accord, ni pas d'accord; 4 = Plutôt d'accord; 5 = Tout à fait d'accord]**<br>1. Les hommes/femmes politiques sont corrompus.<br>2. La plupart des hommes/femmes politiques sont compétents.	1	5

	4. Politicians do not understand what is going on in society.<br>5. Many politicians have been in place for too long.<br>6. The way we organise elections in this country is fair.<br>7. Political parties adequately take into account independent experts when they make decisions.	6. De manier waarop we verkiezingen organiseren in dit land is eerlijk.<br>7. Partijen houden voldoende rekening met onafhankelijke experten wanneer ze beleid maken.	3. Les hommes /femmes politiques essaient de tenir leurs promesses.<br>4. Les hommes /femmes politiques ne comprennent pas ce qui se passe dans la société.<br>5. Beaucoup d'hommes/femmes politiques sont là depuis trop longtemps.<br>6. La façon dont nous organisons les élections dans ce pays est juste.<br>7. Les partis politiques tiennent suffisamment compte des experts indépendants lorsqu'ils prennent des décisions.		
w2_Populisme_1 to w2_Populisme_7	**Please indicate to what extent you disagree or agree with the following statements. [1 = Totally disagree; 2 = Somewhat disagree; 3 = Neither agree nor disagree; 4 = Somewhat agree; 5 = Totally agree]**<br>1. Politicians must follow the people's opinion.<br>2. Political opposition is more present between citizens and the elite than between citizens themselves.	**Kunt u aangeven in hoeverre u het eens bent met onderstaande stellingen? [1 = Helemaal oneens; 2 = Eerder oneens; 3 = Noch eens, noch oneens; 4 = Eerder eens; 5 = Helemaal eens]**<br>1. Politici in het parlement moeten zich laten leiden door de mening van het volk.<br>2. De politieke tegenstellingen zijn groter tussen de elite en gewone burgers dan tussen burgers onderling.<br>3. Ik word liever vertegenwoordigd door een	**Pouvez-vous indiquer dans quelle mesure vous êtes d'accord avec les affirmations ci-dessous? [1 = Pas du tout d'accord; 2 = Plutôt pas d'accord; 3 = Ni d'accord, ni pas d'accord; 4 = Plutôt d'accord; 5 = Tout à fait d'accord]**<br>1. Les hommes et femmes politiques doivent suivre l'avis de la population.<br>2. L'opposition politique est plus grande entre les élites et les citoyens,	1	5

	3. I prefer being represented by an ordinary citizen rather than a professional politician.<br>4. When making decisions, politicians care about people like me.<br>5. Politics is the result of compromise and common sense.<br>6. Rich citizens have a bigger influence on politics than do poor citizens.<br>7. In general, politics reflect rather well the people's preferences.	gewone burger dan door een beroepspoliticus.<br>4. Bij het nemen van besluiten geven politici om mensen zoals ik.<br>5. Beleid is doorgaans het resultaat van compromis en gezond verstand.<br>6. Rijke burgers hebben meer invloed op het beleid dan arme burgers.<br>7. Over het algemeen weerspiegelt het beleid de voorkeuren van het volk redelijk goed.	qu'entre les citoyens entre eux.<br>3. Je préfère être représenté par un citoyen ordinaire que par un homme/ femme politique professionnel.<br>4. En prenant des décisions, les hommes /femmes politiques se soucient des gens comme moi.<br>5. La politique est le résultat du compromis et du bon sens.<br>6. Les citoyens riches ont plus d'influence sur les politiques que les citoyens pauvres.<br>7. En général, la politique reflète assez bien les préférences de la population.		
w2_Efficacy_1<br>to<br>w2_Efficacy_8<br><br>* The Dutch and French translations to not fully overlap.	**Please indicate to what extent you disagree or agree with the following statements. [1 = Totally disagree; 2 = Somewhat disagree; 3 = Neither agree nor disagree; 4 = Somewhat agree; 5 = Totally agree]**<br>1. Most of the citizens do not have a clear political preference.	**Kunt u aangeven in hoeverre u het eens bent met onderstaande stellingen? [1 = Helemaal oneens; 2 = Eerder oneens; 3 = Noch eens, noch oneens; 4 = Eerder eens; 5 = Helemaal eens]**<br>1. De meeste burgers hebben geen duidelijke beleidsvoorkeuren.<br>2. Politieke partijen bieden het volk geen echt verschillende beleidsalternatieven.	**Pouvez-vous indiquer dans quelle mesure vous êtes d'accord avec les affirmations ci-dessous? [1 = Pas du tout d'accord; 2 = Plutôt pas d'accord; 3 = Ni d'accord, ni pas d'accord; 4 = Plutôt d'accord; 5 = Tout à fait d'accord]**<br>1. La plupart des citoyens n'ont pas de préférence politique claire.	1	5

	2. Political parties do not offer real political alternatives to the people.<br>3. Political parties give too much freedom to campaign advisers to determine important political issues. *<br>4. The influence of interest groups and lobbyists on policies is too large.<br>5. Voting is pointless because parties do what they want anyway.<br>6. In general, our political system functions fairly.<br>7. Our political decision-making processes are sufficiently transparent.<br>8. In general, our political system functions in an efficient way.<br>9. I have the feeling that I am capable to understand what the most important political questions in Belgium are.	3. Politieke partijen laten campagneadviseurs teveel hun visies over politieke thema's bepalen. *<br>4. De invloed van belangengroepen en lobbyisten op beleid is te groot.<br>5. Gaan stemmen is zinloos, partijen doen toch wat ze zelf willen.<br>6. In het algemeen werkt ons politiek systeem op een eerlijke manier.<br>7. Onze politieke besluitvormingsprocessen zijn voldoende transparant.<br>8. In het algemeen werkt ons politiek systeem op een efficiënte manier.<br>9. Ik heb het gevoel dat ik redelijk goed begrijp wat de belangrijke politieke vraagstukken in België zijn.	2. Les partis politiques n'offrent pas à la population de véritables alternatives politiques.<br>3. Les partis politiques laissent trop les conseillers de campagne déterminer les thèmes politiques importants. *<br>4. L'influence des groupes d'intérêts et des lobbys sur les politiques est trop grande.<br>5. Aller voter ne sert à rien, les partis font toujours ce qu'ils veulent.<br>6. En général, notre système politique fonctionne de manière honnête.<br>7. Nos processus de décision politique sont suffisamment transparents.<br>8. En général, notre système politique fonctionne efficacement.<br>9. J'ai le sentiment d'avoir une assez bonne compréhension des questions politiques importantes en Belgique.		

w2_Eval_DemProc_1 to w2_Eval_DemProc_9	**In Belgium, democracy is organized according to a number of legal rules and informal practices. We will introduce you to a few. For each of these rules that organize democracy in Belgium, can you tell us what you think? [1 = Very negative; 2 = Somewhat negative; 3 = Somewhat positive; 4 = Very positive; 99 = I do not know/no opinion]**<br>1. Free elections are held every five years to appoint the members who will sit in Parliament.<br>2. Governments are composed of coalitions between several parties. These parties must negotiate after the elections to find an agreement in order to form a government together.<br>3. Apart from voting during elections, citizens are currently not involved in the political decision-making processes.<br>4. In Belgium, there are currently 50 ministers and 467 parliamentarians	**In België is de democratie geregeld door verschillende wettelijke regels en informele gewoonten. Hieronder leggen we er een aantal aan u voor. We zouden graag uw mening hierover kennen. Kan u voor elk van deze regels of gewoonten aangeven hoe u er over denkt? [1 = Heel negatief; 2 = Eerder negatief; 3 = Eerder positief; 4 = Heel positief; 99 = Weet niet/Geen mening]**<br>1. Elke vijf jaar worden er vrije verkiezingen georganiseerd om de parlementsleden te verkiezen.<br>2. Regeringen zijn coalities tussen verschillende partijen. Na de verkiezingen moeten deze partijen onderhandelen om tot een overeenkomst te komen, en zo samen een regering te vormen.<br>3. Momenteel zijn burgers zelden betrokken bij politieke beslissingen, behalve bij de verkiezingen.<br>4. In België zijn er momenteel 50 ministers en 467 parlementsleden, verspreid over 3 beleidsniveaus (federaal, gemeenschap en gewest).<br>5. In België ligt het nettoloon van een federale	**En Belgique, la démocratie est organisée selon un certain nombre de règles légales et de pratiques informelles. Nous allons vous en présenter quelques-unes. Pour chacune de ces règles qui organisent la démocratie en Belgique, pouvez-vous nous dire ce que vous en pensez? [1 = Très négatif; 2 = Plutôt négatif; 3 = Plutôt positif; 4 = Très positif; 99 = Ne sais pas/sans opinion]**<br>1. Des élections libres sont organisées tous les cinq ans pour désigner les député.e.s qui siègeront au Parlement.<br>2. Les gouvernements sont des coalitions entre plusieurs partis. Ces partis doivent négocier après les élections pour se mettre d'accord pour former un gouvernement ensemble.<br>3. Actuellement, en dehors des élections les citoyens sont rarement impliqués dans la prise de décision politique.<br>4. En Belgique, il y a actuellement 50	1	4

	split across three levels of government (federal, regional and community level).<br>5. In Belgium, the net monthly income of a federal member is between 5500 and 6600 net euros per month.<br>6. Currently, it is possible to be at the same time member of parliament, mayor and alderman.<br>7. Currently, it is the party leaders who decide who will be proposed to become minister.<br>8. In Belgium, in public companies, the Board of Directors is composed of persons designated by the political parties.<br>9. Currently in Belgium, political power is divided into five levels of government: the communes, the provinces, the regions, the communities and the federal state.	volksvertegenwoordiger tussen 5500 en 6000 euro per maand.<br>6. Op dit ogenblik is het mogelijk om op hetzelfde moment zowel parlementslid als burgemeester of schepen te zijn.<br>7. Momenteel beslissen de partijvoorzitters wie er minister wordt.<br>8. In Belgische overheidsbedrijven is de Raad van Bestuur samengesteld uit personen die benoemd zijn door de politieke partijen.<br>9. De politieke macht is in België verdeeld over vijf bevoegdheidsniveaus: het lokale niveau, de provincies, de gemeenschappen en gewesten, en het federale niveau.	ministres, et 467 parlementaires répartis à 3 niveaux de pouvoir (fédéral, régional, communautaire).<br>5. En Belgique, le salaire mensuel net d'un(e) député(e) fédéral(e) se situe entre 5500 et 6000 euros net par mois.<br>6. Actuellement, il est possible d'être en même temps député et bourgmestre ou échevin.<br>7. Actuellement, ce sont les présidents de parti qui décident qui sera proposé pour devenir ministre.<br>8. En Belgique, dans les entreprises publiques, le Conseil d'Administration est composé de personnes désignées par les partis politiques.<br>9. Actuellement, en Belgique, le pouvoir politique est distribué entre cinq niveaux de pouvoir : les communes, les provinces, les régions, les communautés, et l'état fédéral.		

w2_Sup_Reforms_1 to w2_Sup_Reforms_9<br><br>* The Dutch and French translations to not fully overlap.	**Currently, several propositions for reform of the Belgian democracy are being debated. We will introduce you to a few. For each of these reforms, could you indicate if you are completely against, somewhat against, somewhat in favour, or totally in favour? [1 = Totally against; 2 = Somewhat against; 3 = Somewhat in favour; 4 = Totally in favour; 99 = I do not know/no opinion]**<br>1. Federal, regional and european elections should be held every 10 years instead of every 5 years.<br>2. The election law should be changed to give the majority to a single party that could govern alone.<br>3. The number of ministers and parliamentarians in Belgium should be cut in half.<br>4. The income of the elected should be limited to a maximum of 2500 euros gross per month. *<br>5. Cumulating the mandates of mayor and	**Op dit moment wordt er gediscussieerd over meerdere voorstellen om de Belgische democratie te hervormen. Kan u voor onderstaande voorstellen aangeven of u er volledig voor, eerder voor, eerder tegen, of volledig tegen bent? [1 = Helemaal tegen; 2 = Eerder tegen; 3 = Eerder voor; 4 = Helemaal voor; 99 = Weet niet/geen mening]**<br>1. Federale, regionale en Europese verkiezingen moeten elke 10 jaar worden georganiseerd in plaats van elke 5 jaar.<br>2. De kieswet moet veranderen zodat één partij een meerderheid kan halen en dus alleen kan regeren.<br>3. Het aantal ministers en parlementsleden in België moet gehalveerd worden.<br>4. Het salaris van verkozenen moet beperkt worden tot maximaal €2500 per maand. *<br>5. Het cumuleren van het mandaat van burgemeester en dat van volksvertegenwoordiger moet verboden worden.<br>6. Ministers moeten rechtstreeks door de kiezer worden verkozen.	**Plusieurs propositions de réformes de la démocratie belge sont débattues actuellement. Nous allons vous en citer quelques-unes. Pour chacune des réformes, pourriez-vous nous dire si vous êtes totalement pour, plutôt pour, plutôt contre, ou totalement contre? [1 = Totalement contre; 2 = Plutôt contre; 3 = Plutôt pour; 4 = Totalement pour; 99 = Ne sais pas/sans opinion]**<br>1. Les élections fédérales, régionales et européennes devraient être organisées tous les 10 ans plutôt que tous les 5 ans.<br>2. La loi électorale devrait être changée pour donner la majorité à un seul parti qui pourrait gouverner seul.<br>3. Le nombre de ministres et de parlementaires en Belgique devrait être divisé par deux.<br>4. Le salaire des élus devrait être limité à maximum 2500 euros brut par mois. *	1	4

	member of parliament should be banned.<br>6. Ministers should be elected directly by the voters.<br>7. The appointments for inter-communal companies, public companies and administrations should be done by an agency independent from political parties.<br>8. Provinces should be abolished.<br>9. Experts should take the major political decisions instead of politicians.	7. Alle benoemingen in intercommunales, publieke bedrijven en bij de overheid moeten beslist worden door een agentschap dat onafhankelijk is van politieke partijen.<br>8. De provincies moeten worden afgeschaft.<br>9. Experten moeten de belangrijkste politieke beslissingen nemen in plaats van politici.	5. Le cumul des mandats de bourgmestre et de député devrait être interdit.<br>6. Les ministres devraient être désignés directement par les électeurs.<br>7. Toutes les nominations dans les intercommunales, les entreprises publiques et l'administration devraient être faites par une agence indépendante des partis politiques.<br>8. Les provinces devraient être supprimées.<br>9. Des experts devraient prendre les principales décisions politiques à la place des hommes et femmes politiques.		
w2_Sup_Cit_Exp_1 to w2_Sup_Cit_Exp_6	**We are now going to ask you a series of questions on the way in which you evaluate citizens and experts as political decision-makers. [0-10 scale: 0 = Totally disagree; 10 = Totally agree]**<br>1. Most citizens have all the competences required to make political decisions.	**We leggen u enkele stellingen voor over de manier waarop burgers en experten politieke beslissingen zouden nemen. [0-10 scale: 0 = Helemaal oneens; 10 = Helemaal eens]**<br>1. De meeste burgers zijn competent om politieke beslissingen te nemen.	**Nous allons à présent vous poser une série de questions sur la façon dont vous évaluez les citoyens et les experts comme décideurs politiques. [0-10 scale: 0 = Pas du tout d'accord; 10 = Tout à fait d'accord]**<br>1. La plupart des citoyens ont toutes les	0	10

	2. Most experts have all the competences required to make political decisions.<br>3. Most citizens are honest.<br>4. Most experts are honest.<br>5. Most citizens are capable of understanding the needs of people like me.<br>6. Most experts are capable of understanding the needs of people like me.	2. De meeste experts zijn competent om politieke beslissingen te nemen.<br>3. De meeste burgers zijn eerlijk.<br>4. De meeste experts zijn eerlijk.<br>5. De meeste burgers zijn in staat om de noden van personen zoals ik te begrijpen.<br>6. De meeste experts zijn in staat om de noden van personen zoals ik te begrijpen.	compétences requises pour prendre des décisions politiques.<br>2. La plupart des experts ont toutes les compétences requises pour prendre des décisions politiques.<br>3. La plupart des citoyens sont honnêtes.<br>4. La plupart des experts sont honnêtes.<br>5. La plupart des citoyens sont capables de comprendre les besoins de personnes comme moi.<br>6. La plupart des experts sont capables de comprendre les besoins de personnes comme moi.		
w2_Exp_Policy_Outcom_1 to w2_Exp_Policy_Outcom_5	**According to you, how likely is it that you will agree with the decisions taken on the following levels of government in the next 5 years? [0-10 scale: 0 = Very unlikely; 10 = Very likely; 99 = I do not know]**<br>1. At the european level<br>2. At the belgian level<br>3. At the flemish level<br>4. At the Brussels level	**Hoe waarschijnlijk is het volgens u dat beslissingen zullen worden genomen waarmee u het eens bent op de volgende regeringsniveaus in de komende vijf jaar? [0-10 scale: 0 = Zeer onwaarschijnlijk; 10 = Zeer waarschijnlijk; 99 = Weet niet]**<br>1. Op Europees niveau<br>2. Op Belgisch niveau<br>3. Op Vlaams niveau<br>4. Op Brussels niveau	**Selon vous, quelle est la probabilité que vous soyez d'accord avec les décisions des niveaux de gouvernement suivants au cours des cinq prochaines années? [0-10 scale: 0 = Peu probable; 10 = Très probable; 99 = Je ne sais pas]**<br>1. Au niveau européen<br>2. Au niveau belge<br>3. Au niveau flamand	0	10

	5. At the walloon level	5. Op Waals niveau	4. Au niveau bruxellois<br>5. Au niveau wallon		
w2_Win_Los	**Would you say that the party for which you voted…**<br>1. Won the elections<br>2. Lost the elections<br>99. I do not know	**Zou u zeggen dat de partij waarop u heeft gestemd…**<br>1. de verkiezingen heeft gewonnen.<br>2. de verkiezingen heeft verloren.<br>99. weet ik niet.	**Diriez-vous que le parti pour lequel vous avez voté...**<br>1. a gagné les élections.<br>2. a perdu les élections.<br>99. Je ne sais pas.	1	2
w2_Sat_election	**To what extent are you satisfied with the outcome of the last elections? [0-10 scale: 0 = Very unsatisfied; 10 = Very satisfied; 99 = I do not know]**	**In hoeverre bent u tevreden met de uitkomst van de afgelopen verkiezingen? [0-10 scale: 0 = Zeer ontevreden; 10 = Zeer tevreden; 99 = Weet ik niet]**	**Dans quelle mesure êtes-vous satisfait(e) du résultat des dernières élections ? [0-10 scale: 0 = Très insatisfait(e); 10 = Très satisfait(e); 99 = Je ne sais pas]**	0	10
w2_Burgers_politici_1 to w2_Burgers_politici_6	**To what extent are you in favour or against the following statement? ”Important political decisions should more often be taken by ordinary citizens rather than by elected politicians”. [0-10 scale: 0= Strongly against; 10 = Strongly in favour]**<br>1. At the european level<br>2. At the belgian level<br>3. At the flemish level<br>4. At the Brussels level<br>5. At the walloon level<br>6. At the local level	**In welke mate bent u voor of tegen het volgende? “Belangrijke politieke beslissingen zouden vaker gemaakt moeten worden door gewone burgers en niet door verkozen politici.” [0-10 scale: 0 = Sterk tegen; 10 = Sterk voor]**<br>1. Op Europees niveau<br>2. Op Belgisch niveau<br>3. Op Vlaams niveau<br>4. Op Brussels niveau<br>5. Op Waals niveau<br>6. Op lokaal niveau	**Dans quelle mesure êtes-vous en faveur ou contre la proposition suivante? "Les décisions politiques importantes devraient être prises plus souvent par les citoyens ordinaires et non par les hommes et femmes politiques élus." [0-10 scale: 0 = Fortement contre; 10 = Fortement pour]**<br>1. Au niveau européen<br>2. Au niveau belge<br>3. Au niveau flamand<br>4. Au niveau bruxellois<br>5. Au niveau wallon<br>6. Au niveau local	0	10
w2_advis_ref	**In general, are you for or against consultative referendums about**	**Bent u, over het algemeen, voor of tegen adviserende referenda over**	**Êtes-vous, en général, pour ou contre les référendums**	0	10

	important national issues? Citizens have the right to vote for or against a specific proposition. The parliament receives the voters' opinion but is not obliged to follow it. [0-10 scale: Strongly against; 10 = Strongly in favour; 99 = I do not know]	belangrijke nationale thema's? Burgers hebben het recht om een stem uit te brengen over een specifiek voorstel. Het parlement ontvangt het advies van de kiezers, maar is niet verplicht om dit te volgen. [0-10 scale: 0 = Sterk tegen; 10 = Sterk voor; 99 = Weet niet]	consultatifs sur les thèmes nationaux importants? Les citoyens ont le droit de voter sur une proposition spécifique. Le Parlement reçoit l'avis des électeurs mais sans être obligé de le suivre. [0-10 scale: 0 = Fortement contre; 10 = Fortement pour; 99 = Je ne sais pas]		
w2_Bindend_ref	In general, are you for or against binding referendums about important national issues? Citizens have the right to vote for or against a specific proposition. The parliament receives the voters' opinion and is obliged to follow it. [0-10 scale: 0 = Strongly against; 10 = Strongly in favour; 99 = I do not know]	Bent u, over het algemeen, voor of tegen bindende referenda over belangrijke nationale thema's? Burgers hebben het recht om een stem uit te brengen over een specifiek voorstel. Het parlement is verplicht het advies van de kiezers te volgen. [0-10 scale: 0 = Sterk tegen; 10 = Sterk voor; 99 = Weet niet]	Êtes-vous, en général, pour ou contre les référendums contraignants sur les thèmes nationaux importants? Les citoyens ont le droit de voter sur une proposition spécifique. Le Parlement est obligé de suivre l'avis de la population. [0-10 scale: 0 = Fortement contre; 10 = Fortement pour; 99 = Je ne sais pas]	0	10
w2_Advis_burgerforum	In general, are you for or against the organisation of consultative citizen forums on important national issues? A citizen forum is an assembly composed of around 30 to 50 citizens, selected at random, who meet and discuss a certain topic in order to formulate a recommendation that is then transmitted to the parliament. [0-10 scale: 0 =	Bent u, over het algemeen, voor of tegen de organisatie van adviserende burgerfora over belangrijke nationale thema's? Een burgerforum bestaat uit 30 tot 50 burgers, die op basis van toeval geselecteerd zijn. Ze komen samen en discussiëren over een bepaald thema om zo tot een advies aan het parlement te komen. [0-10 scale: 0 = Sterk tegen; 10 = Sterk voor; 99 = Weet niet]	Êtes-vous, en général, pour ou contre l'organisation de forums consultatifs de citoyens sur les thèmes nationaux importants? Un forum citoyen est une assemblée composée d'environ 30 à 50 citoyens, tirés au sort, qui se rencontrent et qui discutent d'un sujet spécifique afin de produire une recommandation transmise ensuite au Parlement. [0-10 scale: 0 = Fortement contre;	0	10

	Strongly against; 10 = Strongly in favour; 99 = I do not know]		10 = Fortement pour; 99 = Je ne sais pas]		
w2_burgerbegroting	**In general, are you for or against participative budgeting on a national level? Participative budgeting consists of citizens deciding on a portion of the Belgian state's budget. The citizens involved meet and discuss the way in which they wish to spend that amount in order to support different specific projects.** [0-10 scale: 0 = Strongly against; 10 = Strongly in favour; 99 = I do not know]	**Bent u, over het algemeen, voor of tegen burgerbegrotingen op nationaal niveau? Een burgerbegroting betekent dat burgers beslissen over een deel van de Belgische begroting. De betrokken burgers komen samen en discussiëren over hoe ze het geld wensen te verdelen overheen een aantal concrete projecten.** [0-10 scale: 0 = Sterk tegen; 10 = Sterk voor; 99 = Weet niet]	**Êtes-vous, en général, pour ou contre les budgets participatifs au niveau national? Un budget participatif signifie que des citoyens décident d'une partie du budget de l'Etat belge. Les citoyens impliqués se réunissent et discutent de la façon dont ils souhaitent que l'argent soit dépensé pour soutenir différents projets concrets.** [0-10 scale: 0 = Fortement contre; 10 = Fortement pour; 99 = Je ne sais pas]	0	10
w2_Experts_politic_1<br>to<br>w2_Experts_politic_6	**To what extent do you agree or disagree with the following statement? "Important political decisions should be taken more often by experts rather than elected politicians".** [0-10 scale: 0 = Totally disagree; 10 = Totally agree]<br>1. At the european level<br>2. At the belgian level<br>3. At the the flemish level<br>4. At the walloon level<br>5. At the Brussels level<br>6. At the local level	**In welke mate bent u voor of tegen het volgende?<br>"Belangrijke politieke beslissingen zouden vaker gemaakt moeten worden door experten en niet door verkozen politici."** [0-10 scale: 0 = Sterk tegen; 10 = Sterk voor]<br>1. Op Europees niveau<br>2. Op Belgisch niveau<br>3. Op Vlaams niveau<br>4. Op Waals niveau<br>5. Op Brussels niveau<br>6. Op lokaal niveau	**Dans quelle mesure êtes-vous en faveur ou contre la proposition suivante?<br>"Les décisions politiques importantes devraient être prises plus souvent par des experts et non par des hommes et femmes politiques élus."** [0-10 scale: 0 = Fortement contre; 10 = Fortement pour]<br>1. Au niveau européen<br>2. Au niveau belge<br>3. Au niveau flamand<br>4. Au niveau wallon<br>5. Au niveau bruxellois<br>6. Au niveau local	0	10

[If region = Flanders] OR [If region = Wallonia]: w2_VAA_1 to w2_VAA_16<br><br>[note: Flemish region only got Flemish VAA's; Wallonia only Walloon VAA's]	**During the campaign several voting advice applications were available. Which voting advice applications did you use? Multiple answers are possible.**<br>1. De Stemtest (VRT, De Standaard) - Federal test<br>2. De Stemtest (VRT, De Standaard) - Flemish test<br>3. De Stemtest (VRT, De Standaard) - European test<br>4. Test électoral (RBTF, La Libre Belgique) - Federal test<br>5. Test électoral (RBTF, La Libre Belgique) - Walloon test<br>6. Test électoral (RBTF, La Libre Belgique) - European test<br>7. MNM Stemtest<br>8. De Stem van Vlaanderen (VTM Nieuws, Het Laatste Nieuws)<br>9. Stemchecker (De Morgen)<br>10. Stemwijzer (Knack)<br>11. De Stemcheck (Het Nieuwsblad)<br>12. KiesWijzer<br>13. La voix des Belges - RTL<br>14. Le GPS électoral	**Tijdens de campagne waren er verschillende stemtesten beschikbaar. Aan welke daarvan heeft u deelgenomen? Meerdere antwoorden zijn mogelijk.**<br>1. De Stemtest (VRT, De Standaard) - Federale stemtest<br>2. De Stemtest (VRT, De Standaard) - Vlaamse stemtest<br>3. De Stemtest (VRT, De Standaard) - Europese stemtest<br>4. Test électoral (RBTF, La Libre Belgique) - Federale stemtest<br>5. Test électoral (RBTF, La Libre Belgique) - Waalse stemtest<br>6. Test électoral (RBTF, La Libre Belgique) - Europese stemtest<br>7. MNM Stemtest<br>8. De Stem van Vlaanderen (VTM Nieuws, Het Laatste Nieuws)<br>9. Stemchecker (De Morgen)<br>10. Stemwijzer (Knack)<br>11. De Stemcheck (Het Nieuwsblad)<br>12. KiesWijzer<br>13. La voix des Belges - RTL<br>14. Le GPS électoral<br>15. euandi 2019<br>16. Geen van allen	**Pendant la campagne, plusieurs tests électoraux étaient disponibles. A quels tests avez-vous participé? Plusieurs réponses sont possibles.**<br>1. De Stemtest (VRT, De Standaard) - Test fédéral<br>2. De Stemtest (VRT, De Standaard) - Test flamand<br>3. De Stemtest (VRT, De Standaard) - Test européen<br>4. Test électoral (RBTF, La Libre Belgique) - Test fédéral<br>5. Test électoral (RBTF, La Libre Belgique) - Test wallon<br>6. Test électoral (RBTF, La Libre Belgique) - Test européen<br>7. MNM Stemtest<br>8. De Stem van Vlaanderen (VTM Nieuws, Het Laatste Nieuws)<br>9. Stemchecker (De Morgen)<br>10. Stemwijzer (Knack)<br>11. De Stemcheck (Het Nieuwsblad)<br>12. KiesWijzer<br>13. La voix des Belges - RTL<br>14. Le GPS électoral	0	1

	15. euandi 2019<br>16. None of those		15. euandi 2019<br>16. Aucun d'eux		
[If VAA_1 is selected]<br>w2_Stemtest_fed_VL	**Do you remember which was the first party to appear in the results of the Stemtest 2019 of the VRT or De Standaard for the federal elections?**<br>1. CD&V<br>2. Groen<br>3. N-VA<br>4. Open VLD<br>5. PVDA<br>6. sp.a<br>7. Vlaams Belang<br>99. I do not remember	**En weet u nog welke partij bij u op de eerste plaats uitkwam in De Stemtest van VRT en De Standaard voor de federale verkiezingen?**<br>1. CD&V<br>2. Groen<br>3. N-VA<br>4. Open VLD<br>5. PVDA<br>6. sp.a<br>7. Vlaams Belang<br>99. Ik weet het niet meer	**Et vous rappelez-vous quel était le premier parti qui sortait du résultat du Stemtest 2019 du VRT ou De Standaard pour les élections fédérales ?**<br>1. CD&V<br>2. Groen<br>3. N-VA<br>4. Open VLD<br>5. PVDA<br>6. sp.a<br>7. Vlaams Belang<br>99. Je ne sais plus	1	7
[If answer to VAA _4 is selected]<br>w2_Stemtest_fed_Wal	**Do you remember which was the first party to appear in the results of the Stemtest 2019 of the RTBF or La Libre Belgique for the federal elections?**<br>1. cdH<br>2. DéFI<br>3. Ecolo<br>4. MR<br>5. PP<br>6. PS<br>7. PTB<br>99. I do not remember	**En weet u nog welke partij bij u op de eerste plaats uitkwam in Test électoral van RBTF en La Libre Belgique voor de federale verkiezingen?**<br>1. cdH<br>2. DéFI<br>3. Ecolo<br>4. MR<br>5. PP<br>6. PS<br>7. PTB<br>99. Ik weet het niet meer	**Et vous rappelez-vous quel était le premier parti qui sortait du résultat du Test électoral 2019 de la RTBF ou La Libre Belgique pour les élections fédérales ?**<br>1. cdH<br>2. DéFI<br>3. Ecolo<br>4. MR<br>5. PP<br>6. PS<br>7. PTB<br>99. Je ne sais plus	1	7
[If VAA_2 is selected] AND [If VAA_1 is NOT selected]	**Do you remember which was the first party to appear in the results of the Stemtest 2019 of the VRT**	**En weet u nog welke partij bij u op de eerste plaats uitkwam in De Stemtest van VRT en De Standaard voor de Vlaamse regionale verkiezingen?**	**Et vous rappelez-vous quel était le premier parti qui sortait du résultat du Stemtest 2019 du VRT**	1	7

w2_Stemtest_reg_VL	**or De Standaard for the Flemish regional elections?**<br>1. CD&V<br>2. Groen<br>3. N-VA<br>4. Open VLD<br>5. PVDA<br>6. sp.a<br>7. Vlaams Belang<br>99. I do not remember	1. CD&V<br>2. Groen<br>3. N-VA<br>4. Open VLD<br>5. PVDA<br>6. sp.a<br>7. Vlaams Belang<br>99. Ik weet het niet meer	**ou De Standaard pour les élections régionaux flamands ?**<br>1. CD&V<br>2. Groen<br>3. N-VA<br>4. Open VLD<br>5. PVDA<br>6. sp.a<br>7. Vlaams Belang<br>99. Je ne sais plus		
[If VAA_5 is selected] AND [If VAA_4 is NOT selected]<br>w2_Stemtest_Reg_Wal	**Do you remember which was the first party to appear in the results of the Stemtest 2019 of the RTBF or La Libre Belgique for the Walloon regional elections?**<br>1. cdH<br>2. DéFI<br>3. Ecolo<br>4. MR<br>5. PP<br>6. PS<br>7. PTB<br>99. I do not remember	**En weet u nog welke partij bij u op de eerste plaats uitkwam in De Stemtest van VRT en De Standaard voor de Waalse regionale verkiezingen?**<br>1. cdH<br>2. DéFI<br>3. Ecolo<br>4. MR<br>5. PP<br>6. PS<br>7. PTB<br>99. Ik weet het niet meer	**Et vous rappelez-vous quel était le premier parti qui sortait du résultat du Test électoral 2019 de la RTBF ou La Libre Belgique pour les élections régionaux wallons ?**<br>1. cdH<br>2. DéFI<br>3. Ecolo<br>4. MR<br>5. PP<br>6. PS<br>7. PTB<br>99. Je ne sais plus	1	7
[If VAA_3 is selected] AND [If VAA_1 is NOT selected] AND [If VAA_2 is NOT selected]<br>w2_Stemtest_Europ_VL	**Do you remember which was the first party to appear in the results of the Stemtest 2019 of the VRT or De Standaard for the european elections?**<br>1. CD&V<br>2. Groen<br>3. N-VA<br>4. Open VLD	**En weet u nog welke partij bij u op de eerste plaats uitkwam in De Stemtest van VRT en De Standaard voor de Europese verkiezingen?**<br>1. CD&V<br>2. Groen<br>3. N-VA<br>4. Open VLD<br>5. PVDA	**Et vous rappelez-vous quel était le premier parti qui sortait du résultat du Stemtest 2019 du VRT ou De Standaard pour les élections européennes ?**<br>1. CD&V<br>2. Groen<br>3. N-VA<br>4. Open VLD	1	7

	5. PVDA<br>6. sp.a<br>7. Vlaams Belang<br>99. I do not remember	6. sp.a<br>7. Vlaams Belang<br>99. Ik weet het niet meer	5. PVDA<br>6. sp.a<br>7. Vlaams Belang<br>99. Je ne sais plus		
[If VAA_6 is selected] AND [If VAA_4 is NOT selected] AND [If VAA_5 is NOT selected] w2_Stemtest_Europ_Wal	**Do you remember which was the first party to appear in the results of the Stemtest 2019 of the RTBF or La Libre Belgique for the european elections?**<br>1. cdH<br>2. DéFI<br>3. Ecolo<br>4. MR<br>5. PP<br>6. PS<br>7. PTB<br>99. I do not remember	**En weet u nog welke partij bij u op de eerste plaats uitkwam in De Stemtest van RTBF en La Libre Belgique voor de Europese verkiezingen?**<br>1. cdH<br>2. DéFI<br>3. Ecolo<br>4. MR<br>5. PP<br>6. PS<br>7. PTB<br>99. Ik weet het niet meer	**Et vous rappelez-vous quel était le premier parti qui sortait du résultat du Stemtest 2019 de la RTBF ou La Libre Belgique pour les élections européennes ?**<br>1. cdH<br>2. DéFI<br>3. Ecolo<br>4. MR<br>5. PP<br>6. PS<br>7. PTB<br>99. Je ne sais plus	1	7
[If VAA_7 is selected] AND [If VAA_1 is NOT selected] AND [If VAA_2 is NOT selected] AND [If VAA_3 is NOT selected] w2_Stemtest_MNM	**Do you remember which was the first party to appear in the results of the MNM electoral test?**<br>1. CD&V<br>2. Groen<br>3. N-VA<br>4. Open VLD<br>5. PVDA<br>6. sp.a<br>7. Vlaams Belang<br>99. I do not remember	**En weet u nog welke partij bij u op de eerste plaats uitkwam in De Stemtest van MNM?**<br>1. CD&V<br>2. Groen<br>3. N-VA<br>4. Open VLD<br>5. PVDA<br>6. sp.a<br>7. Vlaams Belang<br>99. Ik weet het niet meer	**Et vous rappelez-vous quel était le premier parti qui sortait du résultat du test électoral de MNM ?**<br>1. CD&V<br>2. Groen<br>3. N-VA<br>4. Open VLD<br>5. PVDA<br>6. sp.a<br>7. Vlaams Belang<br>99. Je ne sais plus	1	7
[If there is more than one answer AND VAA_1 OR VAA_2 OR VAA_3 OR VAA_4 OR	**After completing other electoral tests, did you get the same party as first result?**<br>1. Yes	**Kwam u bij de andere stemtest(en) waaraan u deelnam bij dezelfde partij terecht?**<br>1. Ja	**Après avoir fait d'autres tests électoraux, avez-vous obtenu le même parti en premier ?**<br>1. Oui	1	2

VAA_5 OR VAA_6 OR VAA_7 is selected]<br>w2_Other_VAA	2. No<br>99. I do not know	2. Nee<br>99. Weet ik niet	2. Non<br>99. Je ne sais pas		
w2_Value4	**Some think that euthanasia (choosing to end one's own life on his or her own initiative in case of incurable disease) should be allowed. Others think that euthanasia should in no case be an option. Where would you place yourself on a scale going from 0 to 10, where 0 means that euthanasia should not be possible in any case and 10 means that euthanasia should be possible in any case. [0-10 scale: 0 = Euthanasia should not be allowed in any case; 10 = Euthanasia must be allowed]**	**Sommigen vinden dat euthanasie (het op eigen verzoek beëindigen van het leven in geval van ongeneeslijke ziekte) moet worden toegestaan. Anderen vinden dat euthanasie in geen enkel geval mogelijk mag zijn. Waar zou u uzelf plaatsen op een schaal van 0 tot 10, waarbij 0 betekent dat euthanasie in geen enkel geval mogelijk mag zijn en 10 dat euthanasie in elk geval mogelijk moet zijn. [0-10 scale: 0 = Euthanasie in geen enkel geval mogelijk; 10 = Euthanasie in elk geval mogelijk]**	**Certains trouvent que l'euthanasie (choisir de mettre fin à ses jours de sa propre initiative en cas de maladie incurable) doit être autorisé. D'autres pensent que l'euthanasie ne devrait en aucun cas être possible. Pouvez-vous situer votre opinion sur une échelle allant de 0 à 10, où 0 signifie que l'euthanasie ne devrait en aucun cas être possible et 10 que l'euthanasie devrait être possible dans tous les cas. [0-10 scale: 0 = L'euthanasie ne doit en aucun cas être autorisée; 10 = L'euthanasie ne doit être autorisée en aucune circonstance]**	0	10
w2_VAA_statement s_1<br>to<br>w2_VAA_statement s_18	**Below you find a list of statements. Could you indicate the extent to which you agree or disagree with the following statements? [1 = Totally disagree; 2 = Disagree; 3 = Agree; 4 = Totally agree]**<br>1. Hosting transit migrants must be a punishable offence.	**Hieronder vindt u enkele stellingen. Kan u voor elk van volgende beleidsvoorstellen aangeven in welke mate u het ermee eens of oneens bent? [1 = Helemaal oneens; 2 = Eerder oneens; 3 = Eerder eens; 4 = Helemaal eens]**<br>1. Transmigranten onderdak bieden moet strafbaar zijn.	**Ci-dessous vous trouverez une série de propositions. Pouvez-vous indiquer dans quelle mesure vous êtes d'accord ou pas d'accord avec les propositions suivantes? [1 = Pas du tout d'accord; 2 = Pas d'accord; 3 = D'accord; 4 = Tout à fait d'accord]**	1	4

	2. Situation tests must be put in place to detect discrimination in employment.<br>3. There must be a test on the European values in order to obtain the Belgian nationality.<br>4. If the request for asylum of families with children is rejected, these families can be placed in detention pending their repatriation.<br>5. By 2024, the company cars that run with petrol or diesel must be banned.<br>6. The VAT on electricity must be reduced from 21 to 6%.<br>7. There must be a tax on plane tickets in order to raise their price.<br>8. Nuclear power plants must remain operational after 2025.<br>9. We cannot drive while having drunk alcohol.<br>10. Abortion must be allowed beyond the 12th week of a pregnancy.	2. De overheid moet praktijktesten doen om te controleren op discriminatie bij aanwervingen.<br>3. Nieuwkomers moeten eerst slagen voor een examen over de Europese waarden voor ze de Belgische nationaliteit kunnen verwerven.<br>4. Als de asielaanvraag van gezinnen met kinderen is afgewezen, mogen ze in afwachting van hun terugkeer worden opgesloten.<br>5. Bedrijfswagens die op benzine of diesel rijden moeten voor 2024 verboden worden.<br>6. De btw op elektriciteit moet verlaagd worden van 21 naar 6%.<br>7. Vliegen moet duurder worden door de tickets te belasten.<br>8. Ook na 2025 moeten we kerncentrales openhouden.<br>9. Als je rijdt mag je helemaal geen alcohol gedronken hebben.<br>10. Ook na 12 weken zwangerschap moet abortus nog toegestaan worden.<br>11. Spermadonatie mag niet langer anoniem gebeuren.	1. Héberger des migrants en transit doit être un délit punissable.<br>2. Il faut mettre en place des tests de situation pour détecter la discrimination à l'embauche.<br>3. Pour pouvoir obtenir la nationalité belge, il faut d'abord avoir réussi un examen sur les valeurs européennes.<br>4. Si la demande d'asile de familles avec enfants a été rejetée, ces familles peuvent être placées en détention en attendant leur rapatriement.<br>5. D'ici 2024, les voitures de société qui roulent à l'essence ou au diesel doivent être interdites.<br>6. La TVA sur l'électricité doit être réduite de 21 à 6%.<br>7. Il faut taxer les billets d'avion pour qu'ils soient plus chers.<br>8. Les centrales nucléaires doivent rester opérationnelles après 2025.		

	11. Sperm donation must no longer be anonymous.<br>12. Great fortunes must be more taxed.<br>13. Wages must no longer be automatically indexed.<br>14. The fingerprints of all citizens must be kept in a central database.<br>15. Shops must be able to choose when to do sales.<br>16. A retirement pension of at least 1500€ per month must be put in place.<br>17. The government should be composed of an equal number of men and women.<br>18. Important political decisions must be handled by citizens via a referendum.	12. Grote vermogens moeten meer worden belast.<br>13. De lonen mogen niet meer automatisch aan de prijsstijgingen worden aangepast (index).<br>14. Vingerafdrukken van alle burgers moeten in een centrale databank worden bijgehouden.<br>15. Winkels mogen zelf kiezen wanneer ze solden doen.<br>16. Er moet een minimumpensioen komen van minstens €1500 euro per maand.<br>17. Er moeten in de regering evenveel mannen als vrouwen zitten.<br>18. Belangrijke politieke beslissingen moeten via een referendum aan burgers kunnen worden overgelaten.	9. On ne peut pas conduire en ayant bu de l'alcool.<br>10. L'avortement doit aussi être autorisé au-delà de 12 semaines de grossesse.<br>11. Le don de sperme ne doit plus pouvoir être anonyme.<br>12. Les grandes fortunes doivent être plus taxées.<br>13. Les salaires ne doivent plus être automatiquement indexés.<br>14. Les empreintes digitales de tous les citoyens doivent être conservées dans une base de données centrale.<br>15. Les magasins doivent pouvoir choisir quand ils font leurs soldes.<br>16. Il faut instaurer une pension de retraite de 1500€ minimum par mois.<br>17. Le gouvernement doit compter autant d'hommes que de femmes.<br>18. Les décisions politiques importantes doivent		

			pouvoir être laissées aux citoyens par le biais d'un référendum.		
w2_VAA_salience_1 to w2_VAA_salience_18	**Could you select the three to five policy proposals that you consider most important out of the list of proposals presented below ?**<br>1. Hosting transit migrants must be a punishable offence.<br>2. Situation tests must be put in place to detect discrimination in employment.<br>3. There must be a test on the european values in order to obtain the belgian nationality.<br>4. If the request for asylum of families with children is rejected, these families can be placed in detention pending their repatriation.<br>5. By 2024, the company cars that run with petrol or diesel must be banned.<br>6. The VAT on electricity must be reduced from 21 to 6%.	**Kunt u vervolgens uit de eerder vermelde beleidsvoorstellen drie tot vijf voorstellen aanduiden die u het belangrijkst vindt?**<br>1. Transmigranten onderdak bieden moet strafbaar zijn.<br>2. De overheid moet praktijktesten doen om te controleren op discriminatie bij aanwervingen.<br>3. Nieuwkomers moeten eerst slagen voor een examen over de Europese waarden voor ze de Belgische nationaliteit kunnen verwerven.<br>4. Als de asielaanvraag van gezinnen met kinderen is afgewezen, mogen ze in afwachting van hun terugkeer worden opgesloten.<br>5. Bedrijfswagens die op benzine of diesel rijden moeten voor 2024 verboden worden.<br>6. De btw op elektriciteit moet verlaagd worden van 21 naar 6%.<br>7. Vliegen moet duurder worden door de tickets te belasten.<br>8. Ook na 2025 moeten we kerncentrales openhouden.	**Pourriez-vous ensuite identifier trois à cinq propositions parmi les propositions de politiques mentionnées ci-dessus que vous considérez comme les plus importantes ?**<br>1. Héberger des migrants en transit doit être un délit punissable.<br>2. Il faut mettre en place des tests de situation pour détecter la discrimination à l'embauche.<br>3. Pour pouvoir obtenir la nationalité belge, il faut d'abord avoir réussi un examen sur les valeurs européennes.<br>4. Si la demande d'asile de familles avec enfants a été rejetée, ces familles peuvent être placées en détention en attendant leur rapatriement.<br>5. D'ici 2024, les voitures de société qui roulent à l'essence ou au diesel doivent être interdites.	0	1

	7. There must be a tax on plane tickets in order to raise their price.<br>8. Nuclear power plants must remain operational after 2025.<br>9. We cannot drive while having drunk alcohol.<br>10. Abortion must be allowed beyond the 12th week of a pregnancy.<br>11. Sperm donation must no longer be anonymous.<br>12. Great fortunes must be more taxed.<br>13. Wages must no longer be automatically indexed.<br>14. The fingerprints of all citizens must be kept in a central database.<br>15. Shops must be able to choose when to do sales.<br>16. A retirement pension of at least 1500€ per month must be put in place.<br>17. The government should be composed of an equal number of men and women.<br>18. Important political decisions must be handled by citizens via a referendum.	9. Als je rijdt mag je helemaal geen alcohol gedronken hebben.<br>10. Ook na 12 weken zwangerschap moet abortus nog toegestaan worden.<br>11. Spermadonatie mag niet langer anoniem gebeuren.<br>12. Grote vermogens moeten meer worden belast.<br>13. De lonen mogen niet meer automatisch aan de prijsstijgingen worden aangepast (index).<br>14. Vingerafdrukken van alle burgers moeten in een centrale databank worden bijgehouden.<br>15. Winkels mogen zelf kiezen wanneer ze solden doen.<br>16. Er moet een minimumpensioen komen van minstens €1500 euro per maand.<br>17. Er moeten in de regering evenveel mannen als vrouwen zitten.<br>18. Belangrijke politieke beslissingen moeten via een referendum aan burgers kunnen worden overgelaten.	6. La TVA sur l'électricité doit être réduite de 21 à 6%.<br>7. Il faut taxer les billets d'avion pour qu'ils soient plus chers.<br>8. Les centrales nucléaires doivent rester opérationnelles après 2025.<br>9. On ne peut pas conduire en ayant bu de l'alcool.<br>10. L'avortement doit aussi être autorisé au-delà de 12 semaines de grossesse.<br>11. Le don de sperme ne doit plus pouvoir être anonyme.<br>12. Les grandes fortunes doivent être plus taxées.<br>13. Les salaires ne doivent plus être automatiquement indexés.<br>14. Les empreintes digitales de tous les citoyens doivent être conservées dans une base de données centrale.<br>15. Les magasins doivent pouvoir choisir quand ils font leurs soldes.		

			16. Il faut instaurer une pension de retraite de 1500€ minimum par mois.<br>17. Le gouvernement doit compter autant d'hommes que de femmes.<br>18. Les décisions politiques importantes doivent pouvoir être laissées aux citoyens par le biais d'un référendum.		
[If region = Flanders] w2_Perc_PartyPos_w2_1 to w2_Perc_PartyPos_FL_18	**For each of the following statements, could you indicate the parties that are, in your opinion, in favour of each statement? You can select several parties. [1 = CD&V; 2 = Groen; 3 = N-VA; 4 = Open VLD; 5 = PVDA; 6 = sp.a]; 7 = Vlaams Belang]**<br>1. Hosting transit migrants must be a punishable offence.<br>2. Situation tests must be put in place to detect discrimination in employment.<br>3. There must be a test on the european values in	**Kunt u voor elk beleidsvoorstel de partijen aanduiden waarvan u denkt dat ze het met het voorstel eens zijn? U kunt hier meerdere partijen aanklikken. [1 = CD&V; 2 = Groen; 3 = N-VA; 4 = Open VLD; 5 = PVDA; 6 = sp.a]; 7 = Vlaams Belang]**<br>1. Transmigranten onderdak bieden moet strafbaar zijn.<br>2. De overheid moet praktijktesten doen om te controleren op discriminatie bij aanwervingen.<br>3. Nieuwkomers moeten eerst slagen voor een examen over de Europese waarden voor ze de Belgische nationaliteit kunnen verwerven.	**Pour chaque proposition de politique, pouvez-vous indiquer les partis qui, selon vous, sont en accord avec la proposition? Vous pouvez sélectionner plusieurs partis. [1 = CD&V; 2 = Groen; 3 = N-VA; 4 = Open VLD; 5 = PVDA; 6 = sp.a]; 7 = Vlaams Belang]**<br>1. Héberger des migrants en transit doit être un délit punissable.<br>2. Il faut mettre en place des tests de situation pour détecter la discrimination à l'embauche.<br>3. Pour pouvoir obtenir la nationalité belge, il faut d'abord avoir réussi un	1	7

	order to obtain the belgian nationality.<br>4. If the request for asylum of families with children is rejected, these families can be placed in detention pending their repatriation.<br>5. By 2024, the company cars that run with petrol or diesel must be banned.<br>6. The VAT on electricity must be reduced from 21 to 6%.<br>7. There must be a tax on plane tickets in order to raise their price.<br>8. Nuclear power plants must remain operational after 2025.<br>9. We cannot drive while having drunk alcohol.<br>10. Abortion must be allowed beyond the 12th week of a pregnancy.<br>11. Sperm donation must no longer be anonymous.<br>12. Great fortunes must be more taxed.<br>13. Wages must no longer be automatically indexed.	4. Als de asielaanvraag van gezinnen met kinderen is afgewezen, mogen ze in afwachting van hun terugkeer worden opgesloten.<br>5. Bedrijfswagens die op benzine of diesel rijden moeten voor 2024 verboden worden.<br>6. De btw op elektriciteit moet verlaagd worden van 21 naar 6%.<br>7. Vliegen moet duurder worden door de tickets te belasten.<br>8. Ook na 2025 moeten we kerncentrales openhouden.<br>9. Als je rijdt mag je helemaal geen alcohol gedronken hebben.<br>10. Ook na 12 weken zwangerschap moet abortus nog toegestaan worden.<br>11. Spermadonatie mag niet langer anoniem gebeuren.<br>12. Grote vermogens moeten meer worden belast.<br>13. De lonen mogen niet meer automatisch aan de prijsstijgingen worden aangepast (index).<br>14. Vingerafdrukken van alle burgers moeten in een centrale databank worden bijgehouden.	examen sur les valeurs européennes.<br>4. Si la demande d'asile de familles avec enfants a été rejetée, ces familles peuvent être placées en détention en attendant leur rapatriement.<br>5. D'ici 2024, les voitures de société qui roulent à l'essence ou au diesel doivent être interdites.<br>6. La TVA sur l'électricité doit être réduite de 21 à 6%.<br>7. Il faut taxer les billets d'avion pour qu'ils soient plus chers.<br>8. Les centrales nucléaires doivent rester opérationnelles après 2025.<br>9. On ne peut pas conduire en ayant bu de l'alcool.<br>10. L'avortement doit aussi être autorisé au-delà de 12 semaines de grossesse.<br>11. Le don de sperme ne doit plus pouvoir être anonyme.<br>12. Les grandes fortunes doivent être plus taxées.		

	14. The fingerprints of all citizens must be kept in a central database.<br>15. Shops must be able to choose when to do sales.<br>16. A retirement pension of at least 1500€ per month must be put in place.<br>17. The government should be composed of an equal number of men and women.<br>18. Important political decisions must be handled by citizens via a referendum.	15. Winkels mogen zelf kiezen wanneer ze solden doen.<br>16. Er moet een minimumpensioen komen van minstens €1500 euro per maand.<br>17. Er moeten in de regering evenveel mannen als vrouwen zitten.<br>18. Belangrijke politieke beslissingen moeten via een referendum aan burgers kunnen worden overgelaten.	13. Les salaires ne doivent plus être automatiquement indexés.<br>14. Les empreintes digitales de tous les citoyens doivent être conservées dans une base de données centrale.<br>15. Les magasins doivent pouvoir choisir quand ils font leurs soldes.<br>16. Il faut instaurer une pension de retraite de 1500€ minimum par mois.<br>17. Le gouvernement doit compter autant d'hommes que de femmes.<br>18. Les décisions politiques importantes doivent pouvoir être laissées aux citoyens par le biais d'un référendum.		
[If region = Wallonia] w2_Perc_PartyPos_WAL_1 to w2_Perc_PartyPos_WAL_18	**For each of the following statements, could you indicate the parties that are, in your opinion, in favour of each statement? You can select several parties. [1 = cdH; 2 = DéFI; 3 = Ecolo; 4 = MR; 5 = PP; 6 = PS; 7 = PTB]**	**Kunt u voor elk beleidsvoorstel de partijen aanduiden waarvan u denkt dat ze het met het voorstel eens zijn? U kunt hier meerdere partijen aanklikken. [1 = cdH; 2 = DéFI; 3 = Ecolo; 4 = MR; 5 = PP; 6 = PS; 7 = PTB]**<br>1. Transmigranten onderdak bieden moet strafbaar zijn.	**Pour chaque proposition de politique, pouvez-vous indiquer les partis qui, selon vous, sont en accord avec la proposition? Vous pouvez sélectionner plusieurs partis. [1 = cdH; 2 = DéFI; 3 = Ecolo; 4 = MR; 5 = PP; 6 = PS; 7 = PTB]**	1	7

	1. Hosting transit migrants must be a punishable offence.<br>2. Situation tests must be put in place to detect discrimination in employment.<br>3. There must be a test on the european values in order to obtain the belgian nationality.<br>4. If the request for asylum of families with children is rejected, these families can be placed in detention pending their repatriation.<br>5. By 2024, the company cars that run with petrol or diesel must be banned.<br>6. The VAT on electricity must be reduced from 21 to 6%.<br>7. There must be a tax on plane tickets in order to raise their price.<br>8. Nuclear power plants must remain operational after 2025.<br>9. We cannot drive while having drunk alcohol.	2. De overheid moet praktijktesten doen om te controleren op discriminatie bij aanwervingen.<br>3. Nieuwkomers moeten eerst slagen voor een examen over de Europese waarden voor ze de Belgische nationaliteit kunnen verwerven.<br>4. Als de asielaanvraag van gezinnen met kinderen is afgewezen, mogen ze in afwachting van hun terugkeer worden opgesloten.<br>5. Bedrijfswagens die op benzine of diesel rijden moeten voor 2024 verboden worden.<br>6. De btw op elektriciteit moet verlaagd worden van 21 naar 6%.<br>7. Vliegen moet duurder worden door de tickets te belasten.<br>8. Ook na 2025 moeten we kerncentrales openhouden.<br>9. Als je rijdt mag je helemaal geen alcohol gedronken hebben.<br>10. Ook na 12 weken zwangerschap moet abortus nog toegestaan worden.<br>11. Spermadonatie mag niet langer anoniem gebeuren.	1. Héberger des migrants en transit doit être un délit punissable.<br>2. Il faut mettre en place des tests de situation pour détecter la discrimination à l'embauche.<br>3. Pour pouvoir obtenir la nationalité belge, il faut d'abord avoir réussi un examen sur les valeurs européennes.<br>4. Si la demande d'asile de familles avec enfants a été rejetée, ces familles peuvent être placées en détention en attendant leur rapatriement.<br>5. D'ici 2024, les voitures de société qui roulent à l'essence ou au diesel doivent être interdites.<br>6. La TVA sur l'électricité doit être réduite de 21 à 6%.<br>7. Il faut taxer les billets d'avion pour qu'ils soient plus chers.<br>8. Les centrales nucléaires doivent rester opérationnelles après 2025.		

	10. Abortion must be allowed beyond the 12th week of a pregnancy.<br>11. Sperm donation must no longer be anonymous.<br>12. Great fortunes must be more taxed.<br>13. Wages must no longer be automatically indexed.<br>14. The fingerprints of all citizens must be kept in a central database.<br>15. Shops must be able to choose when to do sales.<br>16. A retirement pension of at least 1500€ per month must be put in place.<br>17. The government should be composed of an equal number of men and women.<br>18. Important political decisions must be handled by citizens via a referendum.	12. Grote vermogens moeten meer worden belast.<br>13. De lonen mogen niet meer automatisch aan de prijsstijgingen worden aangepast (index).<br>14. Vingerafdrukken van alle burgers moeten in een centrale databank worden bijgehouden.<br>15. Winkels mogen zelf kiezen wanneer ze solden doen.<br>16. Er moet een minimumpensioen komen van minstens €1500 euro per maand.<br>17. Er moeten in de regering evenveel mannen als vrouwen zitten.<br>18. Belangrijke politieke beslissingen moeten via een referendum aan burgers kunnen worden overgelaten.	9. On ne peut pas conduire en ayant bu de l'alcool.<br>10. L'avortement doit aussi être autorisé au-delà de 12 semaines de grossesse.<br>11. Le don de sperme ne doit plus pouvoir être anonyme.<br>12. Les grandes fortunes doivent être plus taxées.<br>13. Les salaires ne doivent plus être automatiquement indexés.<br>14. Les empreintes digitales de tous les citoyens doivent être conservées dans une base de données centrale.<br>15. Les magasins doivent pouvoir choisir quand ils font leurs soldes.<br>16. Il faut instaurer une pension de retraite de 1500€ minimum par mois.<br>17. Le gouvernement doit compter autant d'hommes que de femmes.<br>18. Les décisions politiques importantes doivent		

			pouvoir être laissées aux citoyens par le biais d'un référendum.		
[If region = Brussels] [If language = NL] w2_Per_Par_pos_BX-NL_1 to w2_Per_Par_pos_BX-NL_18	**For each of the following statements, could you indicate the parties that are, in your opinion, in favour of each statement? You can select several parties. [1 = CD&V; 2 = Groen; 4 = N-VA; 5 = Open VLD; 6 = PVDA; 7 = sp.a; 8 = Vlaams Belang]**<br>1. Hosting transit migrants must be a punishable offence.<br>2. Situation tests must be put in place to detect discrimination in employment.<br>3. There must be a test on the european values in order to obtain the belgian nationality.<br>4. If the request for asylum of families with children is rejected, these families can be placed in detention pending their repatriation.<br>5. By 2024, the company cars that run with petrol or diesel must be banned.	**Kunt u voor elk beleidsvoorstel de partijen aanduiden waarvan u denkt dat ze het met het voorstel eens zijn? U kunt hier meerdere partijen aanklikken. [1 = CD&V; 2 = Groen; 4 = N-VA; 5 = Open VLD; 6 = PVDA; 7 = sp.a; 8 = Vlaams Belang]**<br>1. Transmigranten onderdak bieden moet strafbaar zijn.<br>2. De overheid moet praktijktesten doen om te controleren op discriminatie bij aanwervingen.<br>3. Nieuwkomers moeten eerst slagen voor een examen over de Europese waarden voor ze de Belgische nationaliteit kunnen verwerven.<br>4. Als de asielaanvraag van gezinnen met kinderen is afgewezen, mogen ze in afwachting van hun terugkeer worden opgesloten.<br>5. Bedrijfswagens die op benzine of diesel rijden moeten voor 2024 verboden worden.<br>6. De btw op elektriciteit moet verlaagd worden van 21 naar 6%.	**Pour chaque proposition de politique, pouvez-vous indiquer les partis qui, selon vous, sont en accord avec la proposition? Vous pouvez sélectionner plusieurs partis. [1 = CD&V; 2 = Groen; 4 = N-VA; 5 = Open VLD; 6 = PVDA; 7 = sp.a; 8 = Vlaams Belang]**<br>1. Héberger des migrants en transit doit être un délit punissable.<br>2. Il faut mettre en place des tests de situation pour détecter la discrimination à l'embauche.<br>3. Pour pouvoir obtenir la nationalité belge, il faut d'abord avoir réussi un examen sur les valeurs européennes.<br>4. Si la demande d'asile de familles avec enfants a été rejetée, ces familles peuvent être placées en détention en attendant leur rapatriement.<br>5. D'ici 2024, les voitures de société qui roulent à	1	8

	6. The VAT on electricity must be reduced from 21 to 6%.<br>7. There must be a tax on plane tickets in order to raise their price.<br>8. Nuclear power plants must remain operational after 2025.<br>9. We cannot drive while having drunk alcohol.<br>10. Abortion must be allowed beyond the 12th week of a pregnancy.<br>11. Sperm donation must no longer be anonymous.<br>12. Great fortunes must be more taxed.<br>13. Wages must no longer be automatically indexed.<br>14. The fingerprints of all citizens must be kept in a central database.<br>15. Shops must be able to choose when to do sales.<br>16. A retirement pension of at least 1500€ per month must be put in place.<br>17. The government should be composed of an equal number of men and women.	7. Vliegen moet duurder worden door de tickets te belasten.<br>8. Ook na 2025 moeten we kerncentrales openhouden.<br>9. Als je rijdt mag je helemaal geen alcohol gedronken hebben.<br>10. Ook na 12 weken zwangerschap moet abortus nog toegestaan worden.<br>11. Spermadonatie mag niet langer anoniem gebeuren.<br>12. Grote vermogens moeten meer worden belast.<br>13. De lonen mogen niet meer automatisch aan de prijsstijgingen worden aangepast (index).<br>14. Vingerafdrukken van alle burgers moeten in een centrale databank worden bijgehouden.<br>15. Winkels mogen zelf kiezen wanneer ze solden doen.<br>16. Er moet een minimumpensioen komen van minstens €1500 euro per maand.<br>17. Er moeten in de regering evenveel mannen als vrouwen zitten.<br>18. Belangrijke politieke beslissingen moeten via een referendum aan burgers kunnen worden overgelaten.	l'essence ou au diesel doivent être interdites.<br>6. La TVA sur l'électricité doit être réduite de 21 à 6%.<br>7. Il faut taxer les billets d'avion pour qu'ils soient plus chers.<br>8. Les centrales nucléaires doivent rester opérationnelles après 2025.<br>9. On ne peut pas conduire en ayant bu de l'alcool.<br>10. L'avortement doit aussi être autorisé au-delà de 12 semaines de grossesse.<br>11. Le don de sperme ne doit plus pouvoir être anonyme.<br>12. Les grandes fortunes doivent être plus taxées.<br>13. Les salaires ne doivent plus être automatiquement indexés.<br>14. Les empreintes digitales de tous les citoyens doivent être conservées dans une base de données centrale.		

	18. Important political decisions must be handled by citizens via a referendum.		15. Les magasins doivent pouvoir choisir quand ils font leurs soldes.<br>16. Il faut instaurer une pension de retraite de 1500€ minimum par mois.<br>17. Le gouvernement doit compter autant d'hommes que de femmes.<br>18. Les décisions politiques importantes doivent pouvoir être laissées aux citoyens par le biais d'un référendum.		
[If region = Brussels] [If language = FR] w2_Per_Par_pos_BX_FR_1 to w2_Per_Par_pos_BX_FR_18	**For each of the following statements, could you indicate the parties that are, in your opinion, in favour of each statement? You can select several parties.** **[1 = cdH; 2 = DéFI; 4 = Ecolo; 5 = MR; 6 = PP; 7 = PS; 8 = PTB]**<br>1. Hosting transit migrants must be a punishable offence.<br>2. Situation tests must be put in place to detect discrimination in employment.<br>3. There must be a test on the european values in	**Kunt u voor elk beleidsvoorstel de partijen aanduiden waarvan u denkt dat ze het met het voorstel eens zijn? U kunt hier meerdere partijen aanklikken.** **[1 = cdH; 2 = DéFI; 4 = Ecolo; 5 = MR; 6 = PP; 7 = PS; 8 = PTB]**<br>1. Transmigranten onderdak bieden moet strafbaar zijn.<br>2. De overheid moet praktijktesten doen om te controleren op discriminatie bij aanwervingen.<br>3. Nieuwkomers moeten eerst slagen voor een examen over de Europese waarden voor ze de Belgische nationaliteit kunnen verwerven.	**Pour chaque proposition de politique, pouvez-vous indiquer les partis qui, selon vous, sont en accord avec la proposition? Vous pouvez sélectionner plusieurs partis.** **[1 = cdH; 2 = DéFI; 4 = Ecolo; 5 = MR; 6 = PP; 7 = PS; 8 = PTB]**<br>1. Héberger des migrants en transit doit être un délit punissable.<br>2. Il faut mettre en place des tests de situation pour détecter la discrimination à l'embauche.	1	8

	order to obtain the belgian nationality.<br>4. If the request for asylum of families with children is rejected, these families can be placed in detention pending their repatriation.<br>5. By 2024, the company cars that run with petrol or diesel must be banned.<br>6. The VAT on electricity must be reduced from 21 to 6%.<br>7. There must be a tax on plane tickets in order to raise their price.<br>8. Nuclear power plants must remain operational after 2025.<br>9. We cannot drive while having drunk alcohol.<br>10. Abortion must be allowed beyond the 12th week of a pregnancy.<br>11. Sperm donation must no longer be anonymous.<br>12. Great fortunes must be more taxed.<br>13. Wages must no longer be automatically indexed.	4. Als de asielaanvraag van gezinnen met kinderen is afgewezen, mogen ze in afwachting van hun terugkeer worden opgesloten.<br>5. Bedrijfswagens die op benzine of diesel rijden moeten voor 2024 verboden worden.<br>6. De btw op elektriciteit moet verlaagd worden van 21 naar 6%.<br>7. Vliegen moet duurder worden door de tickets te belasten.<br>8. Ook na 2025 moeten we kerncentrales openhouden.<br>9. Als je rijdt mag je helemaal geen alcohol gedronken hebben.<br>10. Ook na 12 weken zwangerschap moet abortus nog toegestaan worden.<br>11. Spermadonatie mag niet langer anoniem gebeuren.<br>12. Grote vermogens moeten meer worden belast.<br>13. De lonen mogen niet meer automatisch aan de prijsstijgingen worden aangepast (index).<br>14. Vingerafdrukken van alle burgers moeten in een centrale databank worden bijgehouden.	3. Pour pouvoir obtenir la nationalité belge, il faut d'abord avoir réussi un examen sur les valeurs européennes.<br>4. Si la demande d'asile de familles avec enfants a été rejetée, ces familles peuvent être placées en détention en attendant leur rapatriement.<br>5. D'ici 2024, les voitures de société qui roulent à l'essence ou au diesel doivent être interdites.<br>6. La TVA sur l'électricité doit être réduite de 21 à 6%.<br>7. Il faut taxer les billets d'avion pour qu'ils soient plus chers.<br>8. Les centrales nucléaires doivent rester opérationnelles après 2025.<br>9. On ne peut pas conduire en ayant bu de l'alcool.<br>10. L'avortement doit aussi être autorisé au-delà de 12 semaines de grossesse.		

	14. The fingerprints of all citizens must be kept in a central database.<br>15. Shops must be able to choose when to do sales.<br>16. A retirement pension of at least 1500€ per month must be put in place.<br>17. The government should be composed of an equal number of men and women.<br>18. Important political decisions must be handled by citizens via a referendum.	15. Winkels mogen zelf kiezen wanneer ze solden doen.<br>16. Er moet een minimumpensioen komen van minstens €1500 euro per maand.<br>17. Er moeten in de regering evenveel mannen als vrouwen zitten.<br>18. Belangrijke politieke beslissingen moeten via een referendum aan burgers kunnen worden overgelaten.	11. Le don de sperme ne doit plus pouvoir être anonyme.<br>12. Les grandes fortunes doivent être plus taxées.<br>13. Les salaires ne doivent plus être automatiquement indexés.<br>14. Les empreintes digitales de tous les citoyens doivent être conservées dans une base de données centrale.<br>15. Les magasins doivent pouvoir choisir quand ils font leurs soldes.<br>16. Il faut instaurer une pension de retraite de 1500€ minimum par mois.<br>17. e gouvernement doit compter autant d'hommes que de femmes.<br>18. Les décisions politiques importantes doivent pouvoir être laissées aux citoyens par le biais d'un référendum.		
w2_Per_par_certain	**To what extent were you certain of your assessment of whether**	**Hoe zeker was u in het algemeen bij het beoordelen of de partijen het al dan**	**Dans quelle mesure étiez-vous certain de votre évaluation si les partis sont d'accord ou non avec**	1	4

	**parties are in favour of the policy proposals listed before?**<br>1. Very uncertain<br>2. Somewhat uncertain<br>3. Somewhat certain<br>4. Very certain	**niet met de beleidsvoorstellen eens zijn?**<br>1. Heel onzeker<br>2. Eerder onzeker<br>3. Eerder zeker<br>4. Heel zeker	**les propositions de politiques à mener ?**<br>1. Très incertain<br>2. Plutôt incertain<br>3. Plutôt certain<br>4. Très certain		
[If region = Flanders] w2_Affective_Pol_VL_1 to w2_Affective_Pol_VL_9	**Could you use the scale below to indicate how you feel about the following groups? [0-100 scale: 0 to 50 = Not very favourable; 50 = Neutral; 50 to 100 = Favourable]**<br>1. Walloons<br>2. Flemish<br>3. CD&V sympathisers<br>4. Groen sympathisers<br>5. N-VA sympathisers<br>6. Open VLD sympathisers<br>7. PvdA sympathisers<br>8. sp.a sympathisers<br>9. Vlaams Belang sympathisers	**Kunt u de thermometer hier beneden gebruiken om aan te geven hoe u denkt over de volgende groepen? [0-100 percentages: 0 to 50 = Niet gunstig gezind; 50 = Neutraal; 50 to 100 = Gunstig gezind]**<br>1. Walen<br>2. Vlamingen<br>3. Aanhangers van CD&V<br>4. Aanhangers van Groen<br>5. Aanhangers van N-VA<br>6. Aanhangers van Open VLD<br>7. Aanhangers van PvdA<br>8. Aanhangers van sp.a<br>9. Aanhangers van Vlaams Belang	**Pouvez-vous utiliser l'échelle ci-dessous pour indiquer ce que vous pensez des groupes suivants? [0-100 percentages: 0 to 50 = Pas vraiment favorable; 50 = Neutre; 50 to 100 = Favorable]**<br>1. Wallons<br>2. Flamands<br>3. Les sympathisants de CD&V<br>4. Les sympathisants de Groen<br>5. Les sympathisants de N-VA<br>6. Les sympathisants de Open VLD<br>7. Les sympathisants de PvdA<br>8. Les sympathisants de sp.a<br>9. Les sympathisants de Vlaams Belang	0	100
[If region = Wallonia] w2_Affective_Pol_Wal_1 to	**Could you use the scale below to indicate how you feel about the following groups? [0-100 scale: 0 to 50 = Not very favourable; 50 = Neutral; 50 to 100 = Favourable]**	**Kunt u de thermometer hier beneden gebruiken om aan te geven hoe u denkt over de volgende groepen? [0-100 percentages: 0 to 50 = Niet gunstig**	**Pouvez-vous utiliser l'échelle ci-dessous pour indiquer ce que vous pensez des groupes suivants? [0-100 percentages: 0 to 50 = Pas vraiment favorable;**	0	100

w2_Affective_Pol_Wal_9	10. Walloons<br>11. Flemish<br>12. cdH sympathisers<br>13. DéFI sympathisers<br>14. Ecolo sympathisers<br>15. MR sympathisers<br>16. PP sympathisers<br>17. PS sympathisers<br>18. PTB sympathisers	**gezind; 50 = Neutraal; 50 to 100 = Gunstig gezind]**<br>10. Walen<br>11. Vlamingen<br>12. Aanhangers van cdH<br>13. Aanhangers van DéFI<br>14. Aanhangers van Ecolo<br>15. Aanhangers van MR<br>16. Aanhangers van PP<br>17. Aanhangers van PS<br>18. Aanhangers van PTB	**50 = Neutre; 50 to 100 = Favorable]**<br>10. Wallons<br>11. Flamands<br>12. Les sympathisants de cdH<br>13. Les sympathisants de DéFI<br>14. Les sympathisants de Ecolo<br>15. Les sympathisants de MR<br>16. Les sympathisants de PP<br>17. Les sympathisants de PS<br>18. Les sympathisants de PTB		
[If region = Brussels] w2_Affective_Pol_Bxl	**Could you use the scale below to indicate how you feel about the following groups? [0-100 scale: 0 to 50 = Not very favourable; 50 = Neutral; 50 to 100 = Favourable]**<br>1. [Affective_Pol_1] Walloons<br>2. [Affective_Pol_2] Flemish<br>3. [Affective_Pol_3] Brusseleers<br>4. [Affective_Pol_4] Sympathisers of left wing parties<br>5. [Affective_Pol_5] Sympathisers of right wing parties<br>6. [Affective_Pol_6] Sympathisers of dutch-speaking/flemish parties	**Kunt u de thermometer hier beneden gebruiken om aan te geven hoe u denkt over de volgende groepen? [0-100 percentages: 0 to 50 = Niet gunstig gezind; 50 = Neutraal; 50 to 100 = Gunstig gezind]**<br>1. [Affective_Pol_1] Walen<br>2. [Affective_Pol_2] Vlamingen<br>3. [Affective_Pol_3] Brusselaars<br>4. [Affective_Pol_4] Aanhangers van linkse partijen<br>5. [Affective_Pol_5] Aanhangers van rechtse partijen<br>6. [Affective_Pol_6] Aanhangers van Nederlandstalige partijen<br>7. [Affective_Pol_7] Aanhangers van Franstalige partijen	**Pouvez-vous utiliser l'échelle ci-dessous pour indiquer ce que vous pensez des groupes suivants? [0-100 percentages: 0 to 50 = Pas vraiment favorable; 50 = Neutre; 50 to 100 = Favorable]**<br>1. [Affective_Pol_1] Wallons<br>2. [Affective_Pol_2] Flamands<br>3. [Affective_Pol_3] Bruxellois<br>4. [Affective_Pol_4] Les sympathisants des partis de gauche<br>5. [Affective_Pol_5] Les sympathisants des partis de droite<br>6. [Affective_Pol_6] Les sympathisants des partis	0	100

	7. [Affective_Pol_7] Sympathisers of french-speaking parties		néerlandophones/flamands<br>7. [Affective_Pol_7] Les sympathisants des partis francophones		
w2_Social_distance_VL_1 to w2_Social_distance_VL_9	**Suppose that you have a daughter or son who marries. Could you indicate the extent to which you would be satisfied if the fiancé(e) was a… [0-10 scale: 0 = Very unsatisfied; 5 = Neutral; 10 = Very satisfied]**<br>1. Flemish<br>2. Walloon<br>3. CD&V sympathiser<br>4. Groen sympathiser<br>5. N-VA sympathiser<br>6. Open VLD sympathisers<br>7. PvdA sympathiser<br>8. sp.a sympathiser<br>9. Vlaams Belang sympathiser	**Stel u heeft een zoon of dochter die gaat trouwen. Kunt u aangeven hoe tevreden u zou zijn als de verloofde een... [0-10 scale: 0 = Zeer ontevreden; 5 = Neutraal; 10 = Zeer tevreden]**<br>1. Vlaming is.<br>2. Waal is.<br>3. aanhang(st)er van CD&V is.<br>4. aanhang(st)er van Groen is.<br>5. aanhang(st)er van N-VA is.<br>6. aanhang(st)er van Open VLD is.<br>7. aanhang(st)er van PVDA is.<br>8. aanhang(st)er van sp.a is.<br>9. aanhang(st)er van Vlaams Belang is.	**Supposons que vous ayez un fils ou une fille qui se marie. Pouvez-vous indiquer dans quelle mesure vous seriez satisfait(e) si le/la fiancé(e) était un... [0-10 scale: 0 = Très insatisfait(e); 5 = Neutre; 10 = Très insatisfait(e)]**<br>1. Flamand<br>2. Wallon<br>3. Un sympathisant de CD&V<br>4. Un sympathisant de Groen<br>5. Un sympathisant de N-VA<br>6. Un sympathisant de Open VLD<br>7. Un sympathisant de PvdA<br>8. Un sympathisant de sp.a<br>9. Un sympathisant de Vlaams Belang	0	10
w2_Social_distance_WAL_1 to w2_Social_distance_WAL_9	**Suppose that you have a daughter or son who marries. Could you indicate the extent to which you would be satisfied if the fiancé(e) was a… [0-10 scale: 0 = Very unsatisfied; 5 = Neutral; 10 = Very satisfied]**<br>1. Flemish	**Stel u heeft een zoon of dochter die gaat trouwen. Kunt u aangeven hoe tevreden u zou zijn als de verloofde een... [0-10 scale: 0 = Zeer ontevreden; 5 = Neutraal; 10 = Zeer tevreden]**<br>1. Vlaming is.<br>2. Waal is.	**Supposons que vous ayez un fils ou une fille qui se marie. Pouvez-vous indiquer dans quelle mesure vous seriez satisfait(e) si le/la fiancé(e) était un... [0-10 scale: 0 = Très insatisfait(e); 5 = Neutre; 10 = Très insatisfait(e)]**<br>1. Flamand	0	10

	2. Walloon<br>3. cdH sympathiser<br>4. DéFI sympathiser<br>5. Ecolo sympathiser<br>6. MR sympathisers<br>7. PP sympathiser<br>8. PS sympathiser<br>9. PTB sympathiser	3. aanhang(st)er van cdH is.<br>4. aanhang(st)er van DéFI is.<br>5. aanhang(st)er van Ecolo is.<br>6. aanhang(st)er van MR is.<br>7. aanhang(st)er van PP is.<br>8. aanhang(st)er van PS is.<br>9. aanhang(st)er van PTB is.	2. Wallon<br>3. Un sympathisant de cdH<br>4. Un sympathisant de DéFI<br>5. Un sympathisant de Ecolo<br>6. Un sympathisant de MR<br>7. Un sympathisant de PP<br>8. Un sympathisant de PS<br>9. Un sympathisant de PTB		
w2_Social_distance_BXL_1 to w2_Social_dicstance_BXL_9	**Suppose that you have a daughter or son who marries. Could you indicate the extent to which you would be satisfied if the fiancé(e) was a… [0-10 scale: 0 = Very unsatisfied; 5 = Neutral; 10 = Very satisfied]**<br>1. Flemish.<br>2. Walloon.<br>3. A person from Brussels.<br>4. A sympathiser of a leftwing party.<br>5. A sympathiser of a rightwing party.<br>6. A sympathiser of a dutch-speaking party.<br>7. A sympathiser of a french-speaking party.	**Stel u heeft een zoon of dochter die gaat trouwen. Kunt u aangeven hoe tevreden u zou zijn als de verloofde een... [0-10 scale: 0 = Zeer ontevreden; 5 = Neutraal; 10 = Zeer tevreden]**<br>1. Vlaming is.<br>2. Waal is.<br>3. Brusselaar is.<br>4. aanhang(st)er van een linkse partij is.<br>5. aanhang(st)er van een rechtse partij is.<br>6. aanhang(st)er van een Nederlandstalige partij is.<br>7. aanhang(st)er van een Franstalige partij is.	**Supposons que vous ayez un fils ou une fille qui se marie. Pouvez-vous indiquer dans quelle mesure vous seriez satisfait(e) si le/la fiancé(e) était un... [0-10 scale: 0 = Très insatisfait(e); 5 = Neutre; 10 = Très insatisfait(e)]**<br>1. Flamand.<br>2. Wallon.<br>3. Bruxellois.<br>4. un sympathisant d'un parti de gauche<br>5. un sympathisant d'un parti de droite.<br>6. Uun sympathisant d'un parti néerlandophone.<br>7. un sympathisant d'un parti néerlandophone.	0	10
w2_sub_ideo_pos_VL_1 to w2_sub_ideo_pos_VL_7	**In politics people often talk of "left" or "right". Can you place the convictions of the following sympathisers on a scale from 0 to 10, with 0 meaning "left" 5 "in the centre", and 10 "right"? [0-10**	**In de politiek worden de termen 'links' en 'rechts' vaak gebruikt. Kunt u aangeven waar u de volgende partij-aanhangers, gemiddeld genomen, zou plaatsen op een schaal van 0 tot 10, waarbij 0 'links', 5 'in het centrum' en**	**On utilise souvent en politique les notions de 'gauche' et de 'droite'. Pouvez-vous situer les opinions des sympathisants ci-dessous sur une échelle allant de**	0	10

	**scale: 0 = Left; 5 = Center; 10 = Right]**<br>1. Sympathisers of CD&V<br>2. Sympathisers of Groen<br>3. Sympathisers of N-VA<br>4. Sympathisers of Open VLD<br>5. Sympathisers of PVDA<br>6. Sympathisers of sp.a<br>7. Sympathisers of Vlaams Belang	**10 'rechts' betekent? [0-10 scale: 0 = Links; 5 = Centrum; 10 = Rechts]**<br>1. Aanhangers van CD&V<br>2. Aanhangers van Groen<br>3. Aanhangers van N-VA<br>4. Aanhangers van Open VLD<br>5. Aanhangers van PVDA<br>6. Aanhangers van sp.a<br>7. Aanhangers van Vlaams Belang	**0 à 10, où 0 signifie 'gauche', 5 'le centre', et 10 'droite' ? [0-10 scale: 0 = Gauche; 5 = Le centre; 10 = Droite]**<br>1. les sympathisants du CD&V<br>2. les sympathisants de Groen<br>3. les sympathisants de la N-VA<br>4. les sympathisants d'Open VLD<br>5. les sympathisants du PVDA<br>6. les sympathisants du sp.a<br>7. les sympathisants du Vlaams Belang		
w2_sub_ideo_pos_WAL_1 to w2_sub_ideo_pos_WAL_7	**In politics people often talk of "left" or "right". Can you place the convictions of the following sympathisers on a scale from 0 to 10, with 0 meaning "left" 5 "in the centre", and 10 "right"? [0-10 scale: 0 = Left; 5 = Center; 10 = Right]**<br>1. Sympathisers of cdH<br>2. Sympathisers of DéFI<br>3. Sympathisers of Ecolo<br>4. Sympathisers of MR<br>5. Sympathisers of PP<br>6. Sympathisers of PS<br>7. Sympathisers of PTB	**In de politiek worden de termen 'links' en 'rechts' vaak gebruikt. Kunt u aangeven waar u de volgende partij-aanhangers, gemiddeld genomen, zou plaatsen op een schaal van 0 tot 10, waarbij 0 'links', 5 'in het centrum' en 10 'rechts' betekent? [0-10 scale: 0 = Links; 5 = Centrum; 10 = Rechts]**<br>1. Aanhangers van cdH<br>2. Aanhangers van DéFI<br>3. Aanhangers van Ecolo<br>4. Aanhangers van MR<br>5. Aanhangers van PP<br>6. Aanhangers van PS<br>7. Aanhangers van PTB	**On utilise souvent en politique les notions de 'gauche' et de 'droite'. Pouvez-vous situer les opinions des sympathisants ci-dessous sur une échelle allant de 0 à 10, où 0 signifie 'gauche', 5 'le centre', et 10 'droite' ? [0-10 scale: 0 = Gauche; 5 = Le centre; 10 = Droite]**<br>1. les sympathisants du cdH<br>2. les sympathisants de DéFI<br>3. les sympathisants d'Ecolo<br>4. les sympathisants de MR<br>5. les sympathisants du PP<br>6. les sympathisants du PS	0	10

			7. les sympathisants de PTB		

4.3 Wave 3 questionnaire

Variable name	Label + Values [English]	Label + Values [Dutch]	Label + Values [French]	Min	Max
w3_Region	**In which region is your main residence located?**<br>1. Brussels Capital Region<br>2. Flemish Region<br>3. Walloon Region	**In welk gewest heeft u uw hoofdverblijfplaats?**<br>1. Brussels Hoofdstedelijk Gewest<br>2. Vlaams Gewest<br>3. Waals Gewest	**Dans quelle région se trouve votre lieu de résidence principale ?**<br>1. Région de Bruxelles-Capitale<br>2. Région flamande<br>3. Région wallonne	2	3
w3_Duration	**Duration of respondent's time to complete the questionnaire of wave 3 (in seconds)**			148	1225891
w3_RecordedDate	**Date on which the respondent's response for wave 3 was recorded**			7 April 2020	27 April 2020
w3_Q_Language	**Language setting for the respondent in wave 3**<br>0. French<br>1. Dutch			0	1
w3_Consent	**Did respondent agree with the informed consent form?**<br>1. Yes<br>2. No			1	1
w3_Age_control	**What is your age?**	**Wat is uw leeftijd?**	**Quel âge avez-vous?**	0	100
w3_COVID19_1<br>To<br>w3_COVID19_6	**To what extent do you agree with the following statements relating to the coronavirus crisis? [1-5 scale: 1 = Totally disagree; 2 = Somewhat disagree; 3 = Neither agree nor disagree; 4 = Somewhat agree; 5 = Totally agree]**<br>1. The federal government is managing the emergence of the coronavirus crisis in a good way.<br>2. I feel very worried when I think about the coronavirus.	**In welke mate bent u het eens met de volgende stellingen over de coronacrisis? [1-5 scale : 1 = Helemaal oneens; 2 = Eerder oneens ; 3 = Noch eens, noch oneens ; 4 = Eerder eens ; 5 = Helemaal eens]**<br>1. De federale regering pakt de uitbraak van het coronavirus op een goede manier aan.<br>2. Ik voel mij ongerust als ik aan het coronavirus denk.<br>3. Ik ben bang dat mijn persoonlijke financiële	**Dans quelle mesure êtes-vous d'accord avec les propositions suivantes relatives à la crise du coronavirus ? [1-5 scale : 1 = Pas du tout d'accord ; 2 = Plutôt pas d'accord ; 3 = Ni d'accord, ni pas d'accord ; 4 = Plutôt d'accord ; 5 = Tout à fait d'accord]**<br>1. Le gouvernement fédéral gère l'apparition de la crise du coronavirus de la bonne manière.<br>2. Je me sens très inquiet(e) quand je pense au coronavirus.	1	5

	3. I am afraid that my personal financial situation will be negatively affected by the various measures taken since the beginning of the coronavirus crisis.<br>4. I am concerned about my health and that of my family.<br>5. I am concerned that my personal freedoms, which are currently limited in order to fight the coronavirus, will not be fully restored once the situation is under control.<br>6. I believe that in times of crisis the government should not waste time in debates and discussions but should act as quickly as possible.	situatie slechter zal worden door de maatregelen rondom de uitbraak van het coronavirus.<br>4. Ik maak mij zorgen over de gezondheid van mijn familie en mijzelf.<br>5. Ik maak mij zorgen dat mijn persoonlijke vrijheden die momenteel ingeperkt zijn om het coronavirus te bestrijden, niet volledig zullen worden hersteld nadat de uitbraak onder controle is.<br>6. Ik denk dat de regering in tijden van crisis niet te veel tijd moet spenderen aan debatteren maar snel moet handelen.	3. J'ai peur que ma situation financière personnelle soit affectée négativement par les différentes mesures prises depuis le début de la crise du coronavirus.<br>4. Je me fais du souci pour ma santé et celle de ma famille.<br>5. Je m'inquiète du fait que mes libertés individuelles qui sont pour l'instant limitées afin de lutter contre le coronavirus ne soient pas totalement rétablies une fois que la situation sera sous contrôle.<br>6. Je trouve que le gouvernement en période de crise ne doit pas perdre de temps en débats et discussions mais doit agir le plus vite possible.		
w3_COVID19_measures_1 to w3_COVID19_measures_6	**The Belgian Government has adopted a series of measures to limit the spread of the coronavirus. To what extent do you agree or disagree with the following measures? [1-4 scale: 1 = Totally disagree; 2 = Somewhat**	**De Belgische regering heeft een aantal maatregelen genomen om de verspreiding van het coronavirus tegen te gaan. In welke mate bent u het eens of oneens met deze maatregelen?**	**Le gouvernement belge a adopté toute une série de mesures pour limiter la propagation du coronavirus. Dans quelle mesure êtes-vous d'accord ou pas d'accord avec les mesures suivantes ? [1-4 scale : 1 = Pas du tout d'accord ; 2 =**	1	4

	disagree; 3 = Somewhat agree; 4 = Totally agree]<br>1. The closure of non-essential businesses.<br>2. The prohibition to go to one's secondary residence.<br>3. The prohibition to have friends over at your own house.<br>4. Closure of schools.<br>5. Ban on non-essential travel.<br>6. Police patrols and fines for people who nevertheless travel.	[1-4 scale : 1 = Helemaal oneens; 2 = Eerder oneens ; 3 = Eerder eens ; 4 = Helemaal eens]<br>1. Het sluiten van niet-essentiële winkels.<br>2. Het verbod om naar tweede verblijven te gaan.<br>3. Het verbod om thuis vrienden te ontvangen.<br>4. Het sluiten van de scholen.<br>5. Verbieden van niet-essentiële verplaatsingen.<br>6. Politiepatrouilles en boetes voor mensen die zich toch verplaatsen.	Plutôt pas d'accord ; 3 = Plutôt d'accord ; 4 = Tout à fait d'accord]<br>1. La fermeture des commerces non-essentiels.<br>2. L'interdiction d'aller dans sa résidence secondaire.<br>3. L'interdiction de recevoir des amis à la maison.<br>4. La fermeture des écoles.<br>5. L'interdiction des déplacements non-essentiels.<br>6. Les patrouilles de police et les amendes pour les gens qui malgré tout se déplacent.		
w3_COVID19_future_meas_1<br>To<br>w3_COVID19_future_meas_5	**If the coronavirus epidemic were to continue to spread, to what extent would you agree or disagree with the adoption of the following measures?** **[1-4 scale: 1 = Totally disagree; 2 = Somewhat disagree; 3 = Somewhat agree; 4 = Totally agree]**<br>1. Mandatory quarantine of all infected persons on places that are controlled by the government.<br>2. Stricter rules on going out of the house (e.g. a maximum distance of 1km from home).<br>3. Closure of all shops.	**Als het coronavirus zich nog verder zou verspreiden, in welke mate zou u het dan eens of oneens zijn met volgende bijkomende maatregelen?** **[1-4 scale : 1 = Helemaal oneens; 2 = Eerder oneens ; 3 = Eerder eens ; 4 = Helemaal eens]**<br>1. Verplichte quarantaine van alle besmette personen op plaatsen die door de overheid worden gecontroleerd.<br>2. Strengere maatregelen over het verlaten van je huis (bijv. maximaal 1 km).	**Si l'épidémie du coronavirus devait continuer de s'étendre, dans quelle mesure seriez-vous d'accord ou pas d'accord avec l'adoption des mesures suivantes ?** **[1-4 scale : 1 = Pas du tout d'accord ; 2 = Plutôt pas d'accord ; 3 = Plutôt d'accord ; 4 = Tout à fait d'accord]**<br>1. Une mise en quarantaine obligatoire de toutes les personnes infectées avec un contrôle par les autorités de tous leurs déplacements.<br>2. Des règles plus strictes sur les sorties en dehors de la maison (par exemple, une	1	4

	4. The use of telephone data to control citizens' movements.<br>5. The closure of all public transport.	3. Het sluiten van alle winkels.<br>4. Het gebruik maken van telefoongegevens om de bewegingen van mensen te controleren.<br>5. Het sluiten van alle openbaar vervoer.	distance maximale de 1km de son domicile).<br>3. La fermeture de tous les magasins.<br>4. L'utilisation des données téléphoniques pour contrôler les déplacements des citoyens.<br>5. La fermeture des transports en commun.		
w3_Principles_Governmen_1<br>To<br>w3_Principles_Governmen_3	**Recently, a minority government was formed in the context of the coronavirus crisis.**<br><br>**In the formation of a government, several principles can play a role. For each of the following principles, can you tell us how important you think it is to follow them or not to form a government?** **[1-7 scale: 1 = Not at all important; 7 = Very important]**<br>1.That the largest party from each language group should be part of the government.<br>2.That the coalition formed has a majority in both language groups.<br>3.That the coalition formed has a majority in Belgium as a whole, but not necessarily in each language group.	**Recent werd er in de context van de coronacrisis een minderheidsregering gevormd op Belgisch niveau.**<br><br>**Bij de vorming van een coalitieregering kunnen verschillende principes een rol spelen. Kan u aangeven in welke mate elk van de volgende principes voor u belangrijk of onbelangrijk zijn bij de vorming van een regering?** **[1-7 scale : 1 = Helemaal niet belangrijk; 7 = Heel belangrijk**<br>1. De grootste partij in elke taalgroep moet altijd deel uitmaken van de regering.<br>2. De coalitie moet een meerderheid hebben in beide taalgroepen.<br>3. De coalitie moet een meerderheid hebben in	**Récemment, un gouvernement minoritaire a été formé dans le contexte de la crise du coronavirus.**<br><br>**Dans la formation d'un gouvernement, plusieurs principes peuvent jouer un rôle. Pouvez vous, pour chacun des principes suivants nous dire dans quelle mesure vous trouvez important de les suivre ou pas pour former un gouvernement ?** **[1-7 scale : 1 = Pas du tout important; 7 = Très important]**<br>1. Que le plus grand parti de chaque groupe linguistique fasse partie du gouvernement.<br>2. Que la coalition formée dispose d'une majorité dans les deux groupes linguistiques.<br>3. Que la coalition formée dispose d'une majorité sur	1	7

		België, en niet in elke taalgroep.	l'ensemble de la Belgique, mais pas nécessairement dans chaque groupe linguistique.		
w3_Coalition_options_1<br>To<br>w3_Coalition_options_6	**As part of the negotiations to form a government in the context of the coronavirus crisis, various coalition options were considered. Can you tell us for each of the following options whether you would have approved it or not? [0-10 scale: 0 = Not a good choice at all; 10 = A very good choice]**<br>1. The current minority government of Sophie Wilmès, which consists of the MR, Open VLD and CD&V with external support from the PS, sp.a, Groen, Ecolo and cdH.<br>2. A government of all parties (N-VA, Open VLD, MR, sp.a, PS, CD&V, cdH, Groen, Ecolo, Défl) except the PTB and Vlaams Belang.<br>3. An all-party government, including the PTB and Vlaams Belang.<br>4. A government composed of the parties already present in the Walloon and Flemish governments (N-VA, Open VLD, CD&V, PS, MR, Ecolo).	**Bij de regeringsonderhandelingen in de context van de coronacrisis werden verschillende opties in overweging genomen. Kan u voor elk van de onderstaande opties aangeven in welke mate u ze al dan niet verkiest? [0-10 scale : 0 = Helemaal geen goede keuze ; 10 = Heel goede keuze]**<br>1. De huidige minderheidsregering geleid door Sophie Wilmès, bestaande uit MR, Open VLD en CD&V en ondersteund door PS, sp.a, Groen, Ecolo en cdH.<br>2. Een regering met alle partijen (N-VA, Open VLD, MR, sp.a, PS, CD&V, cdH, Groen, Ecolo, DéFI) behalve Vlaams Belang en PVDA.<br>3. Een regering met alle partijen, Vlaams Belang en PVDA inbegrepen.<br>4. Een regering met de partijen uit de Vlaamse en Waalse regeringen (N-VA,	**Dans le cadre des négociations pour former un gouvernement dans le contexte de la crise du coronavirus, différentes options de coalition ont été envisagées. Pouvez-vous nous dire pour chacune des formules suivantes si vous l'auriez approuvée ou pas ? [0-10 scale : 0 = Pas du tout un bon choix ; 10 = Un très bon choix]**<br>1. Le gouvernement minoritaire actuel de Sophie Wilmès, qui se compose du MR, de l'Open VLD et du CD&V avec un soutien extérieur du PS, du sp.a, de Groen, d'Ecolo et du cdH.<br>2. Un gouvernement de tous les partis (N-VA, Open VLD, MR, sp.a, PS, CD&V, cdH, Groen, Ecolo, Défl) sauf le PTB et le Vlaams Belang.<br>3. Un gouvernement de tous les partis, y compris le PTB et le Vlaams Belang.<br>4. Un gouvernement composé des partis déjà présents au gouvernement wallon et au gouvernement flamand (N-	0	10

	5. A PS, sp.a, CD&V, MR, Open VLD and N-VA government.<br>6. A Vivaldi government with PS, sp.a, Open VLD, MR, CD&V, Ecolo and Groen.	Open VLD, CD&V, PS, MR, Ecolo).<br>5. Paars-geel: een regering met PS, sp.a, CD&V, MR, Open VLD en N-VA.<br>6. Vivaldi: een regering met PS, sp.a, Open VLD, MR, CD&V, Ecolo en Groen.	VA, Open VLD, CD&V, PS, MR, Ecolo).<br>5. Un gouvernement PS, sp.a, CD&V, MR, Open VLD et N-VA.<br>6. Un gouvernement Vivaldi avec le PS, le sp.a, l'Open VLD, le MR, le CD&V, Ecolo et Groen.		
w3_Experts_covid19_eco_1<br>To<br>w3_Experts_covid19_eco_4	**Can you tell us for each of the following statements to what extent you agree or disagree with it? [1-5 scale: 1 = Totally disagree; 2 = Somewhat disagree; 3 = Neither agree nor disagree; 4 = Somewhat agree; 5 = Totally agree]**<br>**1.** To manage the current coronavirus crisis, the government must simply implement the recommendations of health experts, without question.<br>2. In the current coronavirus crisis, the role of health experts is to advise the government. It is the elected politicians who should remain the ones that make the actual decisions.<br>3. If Belgium were to face a serious economic crisis in the near future and in order to resolve it, the government	**Kan u voor elke van de volgende stellingen aangeven in welke mate u het ermee eens, dan wel oneens bent? [1-5 scale : 1 = Helemaal oneens; 2 = Eerder oneens ; 3 = Noch eens, noch oneens ; 4 = Eerder eens ; 5 = Helemaal eens]**<br>1. Om de huidige coronacrisis aan te pakken, moet de regering de aanbevelingen van gezondheidsexperts gewoon toepassen, zonder ze in vraag te stellen.<br>2. In de huidige coronacrisis is het de rol van gezondheidsexperts om advies te geven. Verkozen politici moeten degenen blijven die de eigenlijk beslissingen nemen.	**Pouvez-vous nous dire pour chacune des affirmations suivantes dans quelle mesure vous êtes d'accord ou non avec elle? [1-5 scale : 1 = Pas du tout d'accord ; 2 = Plutôt pas d'accord ; 3 = Ni d'accord, ni pas d'accord ; 4 = Plutôt d'accord ; 5 = Tout à fait d'accord]**<br>1. Pour gérer la crise actuelle du coronavirus, le gouvernement doit se contenter d'appliquer les recommandations des experts de la santé, sans poser de question.<br>2. Dans la crise actuelle du coronavirus, le rôle des experts de la santé est de conseiller le gouvernement. Ce sont les hommes et femmes politiques élus qui doivent prendre les décisions finales.	1	5

	would simply have to implement the recommendations of health experts, without question.<br>4. If Belgium were to face a serious economic crisis in the near future and in order to resolve it, the role of the economic experts is to advise the government. It is the elected politicians who should remain the ones that make the actual decisions.	3. In het geval dat België in de nabije toekomst in een zware economische crisis geraakt, dan moet de regering, om deze economische crisis aan te pakken, de aanbevelingen van economische experts gewoon toepassen, zonder ze in vraag te stellen.<br>4. In het geval dat België in de nabije toekomst in een zware economische crisis geraakt, dan is het de rol van economische experts om advies te geven. Verkozen politici moeten degenen blijven die de eigenlijke beslissingen nemen.	3. Si la Belgique devait faire face dans un avenir proche à une grave crise économique et afin de la résoudre, le gouvernement devrait se contenter d'appliquer les recommandations d'experts économiques, sans poser de question.<br>4. Si la Belgique devait faire face dans un avenir proche à une grave crise économique et afin de la résoudre, le rôle des experts économiques est de conseiller le gouvernement. Ce sont les hommes et femmes politiques qui doivent prendre les décisions finales.		
[If region = Flanders]<br>w3_VoteFed19_Fl	**For which party did you vote for <u>the Chamber</u> during the national elections on the 26th of May 2019?**<br>1. CD&V<br>2. Groen<br>3. N-VA<br>4. Open VLD<br>5. PVDA<br>6. sp.a<br>7. Vlaams Belang<br>8. Other	**Op welke partij stemde u voor <u>de Kamer</u> bij de afgelopen verkiezingen van 26 mei 2019?**<br>1. CD&V<br>2. Groen<br>3. N-VA<br>4. Open VLD<br>5. PVDA<br>6. sp.a<br>7. Vlaams Belang<br>8. Andere	**Pour quel parti avez-vous voté <u>à la Chambre des Représentants</u> lors des élections fédérales du 26 mai 2019 ?**<br>1. CD&V<br>2. Groen<br>3. N-VA<br>4. Open VLD<br>5. PVDA<br>6. sp.a<br>7. Vlaams Belang<br>8. Autre<br>9. J'ai voté blanc / invalide	1	12

	9. Blank or invalid<br>10. Did not vote<br>11. Was not (yet) eligible to vote<br>12. I do not remember	9. Blanco of Ongeldig gestemd<br>10. Niet gaan stemmen<br>11. Mocht (nog) niet stemmen<br>12. Ik weet het niet meer	10. Je n'ai pas voté<br>11. Je ne pouvais pas (encore) voter<br>12. Je ne m'en rappelle pas		
[If region = Wallonia]<br>w3_VoteFed19_Wal	**For which party did you vote for <u>the Chamber</u> during the national elections on the 26th of May 2019?**<br>1. cdH<br>2. Ecolo<br>3. DéFI<br>4. PP<br>5. MR<br>6. PS<br>7. PTB<br>8. Other<br>9. Blank or invalid<br>10. I did not vote<br>11. I could not (yet) vote<br>12. I do not remember	**Op welke partij stemde u voor <u>de Kamer</u> bij de afgelopen verkiezingen van 26 mei 2019?**<br>1. cdH<br>2. Ecolo<br>3. DéFI<br>4. PP<br>5. MR<br>6. PS<br>7. PTB<br>8. Andere<br>9. Blanco of Ongeldig gestemd<br>10. Niet gaan stemmen<br>11. Mocht (nog) niet stemmen<br>12. Ik weet het niet meer	**Pour quel parti avez-vous voté <u>à la Chambre des Représentants</u> lors des élections fédérales du 26 mai 2019 ?**<br>1. cdH<br>2. Ecolo<br>3. DéFI<br>4. PP<br>5. MR<br>6. PS<br>7. PTB<br>8. Autre<br>9. J'ai voté blanc / invalide<br>10. Je n'ai pas voté<br>11. Je ne pouvais pas (encore) voter<br>12. Je ne m'en rappelle pas	1	12

[If region = Brussels] w3_VoteFed19_Bxl	**For which party did you vote for <u>the Chamber</u> during the national elections on the 26th of May 2019?**<br>1. CD&V<br>2. cdH<br>3. DéFI<br>4. Ecolo-Groen<br>5. MR<br>6. N-VA<br>7. Open VLD<br>8. PP<br>9. PS<br>10. PTB-PVDA<br>11. sp.a<br>12. Vlaams Belang<br>13. Other<br>14. Blank or invalid<br>15. I did not vote<br>16. I could not (yet) vote<br>17. I do not remember	**Op welke partij stemde u voor <u>de Kamer</u> bij de afgelopen verkiezingen van 26 mei 2019?**<br>1. CD&V<br>2. cdH<br>3. DéFI<br>4. Ecolo-Groen<br>5. MR<br>6. N-VA<br>7. Open VLD<br>8. PP<br>9. PS<br>10. PTB-PVDA<br>11. sp.a<br>12. Vlaams Belang<br>13. Andere<br>14. Blanco of Ongeldig gestemd<br>15. Niet gaan stemmen<br>16. Mocht (nog) niet stemmen<br>17. Ik weet het niet meer	**Pour quel parti avez-vous voté à <u>la Chambre des Représentants</u> lors des élections fédérales du 26 mai 2019 ?**<br>1. CD&V<br>2. cdH<br>3. DéFI<br>4. Ecolo-Groen<br>5. MR<br>6. N-VA<br>7. Open VLD<br>8. PP<br>9. PS<br>10. PTB-PVDA<br>11. sp.a<br>12. Vlaams Belang<br>13. Autre<br>14. J'ai voté blanc / invalide<br>15. Je n'ai pas voté<br>16. Je ne pouvais pas (encore) voter<br>17. Je ne m'en rappelle pas	1	17
w3_Win_Los	**Would you say that the party you voted for...**<br>1. Won the election.<br>2. Lost the election.<br>99. I don't know**.**	**Zou u zeggen dat de partij waarop u heeft gestemd...**<br>1. de verkiezingen heeft gewonnen.<br>2. de verkiezingen heeft verloren.<br>99. Weet ik niet.	**Diriez-vous que le parti pour lequel vous avez voté...**<br>1. a gagné les élections.<br>2. a perdu les élections.<br>99. Je ne sais pas.	1	2

w3_Win_los_level_1 To w3_Win_los_level_3	**If you think back to the elections of May 26, 2019, would you say that the party you voted for won or lost those elections?**<br><br>**The party I voted for... [1 = won; 2 = lost; 99 = don't know]**<br>1. In the European elections<br>2. In the federal election<br>3. In the regional elections	**En als u terugdenkt aan de verschillende verkiezingen die plaatsvonden op 26 mei 2019, zou u zeggen dat de partij waarop u heeft gestemd de verkiezingen heeft gewonnen of verloren?**<br><br>**De partij waarop ik stemde tijdens de ...**<br>**[1 = gewonnen; 2 = verloren; 99 = weet ik niet]**<br>1. Europese verkiezingen heeft<br>2. Federale verkiezingen heeft<br>3. Regionale verkiezingen heeft	**Si vous repensez aux élections du 26 mai 2019, diriez vous que le parti pour lequel vous aviez voté a gagné ou perdu ces élections ?**<br><br>**Le parti pour lequel j'ai voté … [1 = gagné ; 2 = perdu ; 99 = je ne sais pas]**<br>1. Aux élections européennes a<br>2. Aux élections fédérales a<br>3. Aux élections régionales a	1	3
w3_Sat_election	**How satisfied are you with the outcome of the last elections on 26 May 2019? [0-10 scale: 0 = Very dissatisfied; 10 = Very satisfied; 99 = Don't know]**	**In hoeverre bent u tevreden met de uitkomst van de afgelopen verkiezingen van 26 mei 2019? [0-10 scale: 0 = Zeer ontevreden; 10 = Zeer tevreden; 99 = Weet ik niet]**	**Dans quelle mesure êtes-vous satisfait(e) du résultat des dernières élections du 26 mai 2019 ? [0-10 scale : 0 = Très insatisfait(e) ; 10 = Très satisfait ; 99 = Je ne sais pas]**	0	10
If region = Flanders] w3_Vote_history_Fl	**We asked you earlier which party you voted for on May 26, 2019. But we would like to know if you always voted for that party. Below you will find a list of parties. Can you indicate for each of these parties whether you have voted for that party at least once in the**	**We vroegen al eerder op welke partij u in 2019 stemde. Maar we zouden ook graag weten of u altijd al op deze partij hebt gestemd. Hieronder ziet u een lijst met partijen, kunt u aangeven op welke van deze partijen u ooit heeft gestemd? Opgelet, uw**	**Nous vous avons déjà demandé plus tôt pour quel parti vous avez voté le 26 mai 2019. Mais nous aimerions savoir si vous votez toujours pour ce parti. Ci-dessous vous trouverez une liste de partis. Pouvez vous indiquer pour chacun de ces partis si vous avez déjà voté au moins une fois**	1	14

	**past? Please do not take into account the vote in the local elections.**<br><br>**We are well aware that this is a complicated question, but we hope that you can help us by doing your best to remember which parties you have voted for in the past.**<br>1. CD&V (CVP)<br>2. Groen (Agalev)<br>3. Lijst Dedecker (LDD)<br>4. N-VA<br>5. Open VLD<br>6. PVDA<br>7. ROSSEM<br>8. Sp.a (SP)<br>9. Spirit (ID21)<br>10. Volksunie<br>11. Vlaams Belang (Vlaams Blok)<br>12. Other party<br>13. None of these parties<br>14. Not applicable. I voted for the first time in 2019.	**stemmen voor de gemeenteraadsverkiezingen tellen niet mee.**<br><br>**We realiseren ons dat dit een heel lastige vraag is die uw geheugen op de proef stelt, maar hopen dat u uw best doet om het u zo precies mogelijk te herinneren.**<br>1. CD&V (CVP)<br>2. Groen (Agalev)<br>3. Lijst Dedecker (LDD)<br>4. N-VA<br>5. Open VLD (VLD)<br>6. PVDA<br>7. ROSSEM<br>8. Sp.a (SP)<br>9. Spirit<br>10. Volksunie<br>11. Vlaams Belang (Vlaams Blok)<br>12. Andere partij<br>13. Geen van deze partijen<br>14. Niet van toepassing: 2019 was de eerste keer dat ik mocht stemmen	**dans le passé pour ce parti ? Attention, ne tenez paz compte du vote aux élections communales.**<br><br>**Nous sommes bien conscients qu'il s'agit d'une question compliquée mais nous espérons que vous pourrez nous aider en faisant de votre mieux pour vous souvenir pour quels partis vous avez déjà voté par le passé.**<br>1. CD&V (CVP)<br>2. Groen (Agalev)<br>3. Lijst Dedecker (LDD)<br>4. N-VA<br>5. Open VLD (VLD)<br>6. PVDA<br>7. ROSSEM<br>8. Sp.a (SP)<br>9. Spirit (ID21)<br>10. Volksunie<br>11. Vlaams Belang (Vlaams Blok)<br>12. Autre parti<br>13. Aucun de ces partis<br>14. Pas d'application. J'ai voté pour la première fois en 2019.		
[If region = Wallonia] w3_Vote_history_Wal	**We asked you earlier which party you voted for on May 26, 2019. But we would like to know if you always voted for that party. Below you will find a list of parties. Can you indicate for each of these**	**We vroegen al eerder op welke partij u in 2019 stemde. Maar we zouden ook graag weten of u altijd al op deze partij hebt gestemd. Hieronder ziet u een lijst met partijen, kunt u aangeven op**	**Nous vous avons déjà demandé plus tôt pour quel parti vous avez voté le 26 mai 2019. Mais nous aimerions savoir si vous votez toujours pour ce parti. Ci-dessous vous trouverez une liste de partis. Pouvez vous indiquer**	1	14

	**parties whether you have voted for that party at least once in the past? Please do not take into account the vote in the local elections.**<br><br>**We are well aware that this is a complicated question, but we hope that you can help us by doing your best to remember which parties you have voted for in the past.**<br>1. cdH (PSC)<br>2. DéFI (FDF)<br>3. Ecolo<br>4. Front National (FN, FNB)<br>5. MR (PRL, PRL-FDF, PRL-FDF-MCC)<br>6. Parti Communiste de Belgique (PCB)<br>7. Parti Populaire (PP)<br>8. PS<br>9. PTB<br>10. Rassemblement Wallon (RW)<br>11. UDRT<br>12. Other party<br>13. None of these parties.<br>14. Not applicable. I voted for the first time in 2019.	**welke van deze partijen u ooit heeft gestemd? Opgelet, uw stemmen voor de gemeenteraadsverkiezingen tellen niet mee.**<br><br>**We realiseren ons dat dit een heel lastige vraag is die uw geheugen op de proef stelt, maar hopen dat u uw best doet om het u zo precies mogelijk te herinneren.**<br>1. cdH (PSC)<br>2. DéFI (FDF)<br>3. Ecolo<br>4. Front National (FN, FNB)<br>5. MR (PRL, PRL-FDF, PRL-FDF-MCC)<br>6. Parti Communiste de Belgique (PCB)<br>7. Parti Populaire (PP)<br>8. PS<br>9. PTB<br>10. Rassemblement Wallon (RW)<br>11. UDRT<br>12. Andere partij<br>13. Geen van deze partijen<br>14. Niet van toepassing: 2019 was de eerste keer dat ik mocht stemmen	**pour chacun de ces partis si vous avez déjà voté au moins une fois dans le passé pour ce parti ? Attention, ne tenez paz compte du vote aux élections communales.**<br><br>**Nous sommes bien conscients qu'il s'agit d'une question compliquée mais nous espérons que vous pourrez nous aider en faisant de votre mieux pour vous souvenir pour quels partis vous avez déjà voté par le passé.**<br>1. cdH (PSC)<br>2. DéFI (FDF)<br>3. Ecolo<br>4. Front National (FN, FNB)<br>5. MR (PRL, PRL-FDF, PRL-FDF-MCC)<br>6. Parti Communiste de Belgique (PCB)<br>7. Parti Populaire (PP)<br>8. PS<br>9. PTB<br>10. Rassemblement Wallon (RW)<br>11. UDRT<br>12. Autre parti<br>13. Aucun de ces partis<br>14. Pas d'application. J'ai voté pour la première fois en 2019.		

[If region = Flanders] w3_ElecUtil_Fl_1 to w3_ElecUtil_Fl_7	**Different political parties are active in Flanders. Can you indicate how likely it is that you will one day vote for the following parties in the next elections? [0-10 scale: 0 = Not very likely; 10 = Very likely]**<br>1. CD&V<br>2. Groen<br>3. N-VA<br>4. Open VLD<br>5. PVDA<br>6. Sp.a<br>7. Vlaams Belang	**In Vlaanderen zijn verschillende politieke partijen actief. Kunt u aangeven hoe waarschijnlijk het is dat u in toekomstige verkiezingen ooit voor de onderstaande partijen zou stemmen? [0-10 scale: 0 = Heel onwaarschjinlijk; 10 = Heel waarschijnlijk]**<br>1. CD&V<br>2. Groen<br>3. N-VA<br>4. Open VLD<br>5. PVDA<br>6. Sp.a<br>7. Vlaams Belang	**Différents partis politiques sont actifs en Flandre. Pouvez-vous indiquer dans quelle mesure il est probable que vous voterez un jour pour les partis suivants lors de prochaines élections ? [0-10 scale : 0 = Peu probable ; 10 = Très probable]**<br>1. CD&V<br>2. Groen<br>3. N-VA<br>4. Open VLD<br>5. PVDA<br>6. Sp.a<br>7. Vlaams Belang	0	10

[If region = Brussels] w3_Elec-Ut-BXL_1 to w3_Elec-Ut-BXL_13	**Different political parties are active in the Brussels-Capital Region. Can you indicate how likely it is that you will one day vote for the following parties in the next elections? [0-10 scale: 0 = Unlikely; 10 = Very likely]**<br>1. CD&V<br>2. cdH<br>3. DéFI<br>4. Ecolo<br>5. Groen<br>6. MR<br>7. N-VA<br>8. Open VLD<br>9. PS<br>10. PTB<br>11. PVDA<br>12. Sp.a<br>13. Vlaams Belang	**In het Brussels Hoofdstedelijk Gewest zijn verschillende politieke partijen actief. Kunt u aangeven hoe waarschijnlijk het is dat u in toekomstige verkiezingen ooit voor de onderstaande partijen zou stemmen? [0-10 scale: 0 = Heel onwaarschjinlijk; 10 = Heel waarschijnlijk]**<br>1. CD&V<br>2. cdH<br>3. DéFI<br>4. Ecolo<br>5. Groen<br>6. MR<br>7. N-VA<br>8. Open VLD<br>9. PS<br>10. PTB<br>11. PVDA<br>12. Sp.a<br>13. Vlaams Belang	**Différents partis politiques sont actifs dans la Région de Bruxelles-Capitale. Pouvez-vous indiquer dans quelle mesure il est probable que vous voterez un jour pour les partis suivants lors de prochaines élections ? [0-10 scale : 0 = Peu probable ; 10 = Très probable]**<br>1. CD&V<br>2. cdH<br>3. DéFI<br>4. Ecolo<br>5. Groen<br>6. MR<br>7. N-VA<br>8. Open VLD<br>9. PS<br>10. PTB<br>11. PVDA<br>12. Sp.a<br>13. Vlaams Belang	0	10

[If region = Wallonia] w3_ElecUtill_Wal_1 to w3_ElecUtill_Wal_6	**Different political parties are active in Wallonia. Can you indicate how likely it is that you will one day vote for the following parties in the next elections? [0-10 scale: 0 = Unlikely; 10 = Very likely]**<br>1. cdH<br>2. DéFI<br>3. Ecolo<br>4. MR<br>5. PS<br>6. PTB	**In Wallonië zijn verschillende politieke partijen actief. Kunt u aangeven hoe waarschijnlijk het is dat u in toekomstige verkiezingen ooit voor de onderstaande partijen zou stemmen? [0-10 scale: 0 = Heel onwaarschijnlijk; 10 = Heel waarschijnlijk]**<br>1. cdH<br>2. DéFI<br>3. Ecolo<br>4. MR<br>5. PS<br>6. PTB	**Différents partis politiques sont actifs en Wallonie. Pouvez-vous indiquer dans quelle mesure il est probable que vous voterez un jour pour les partis suivants lors de prochaines élections ? [0-10 scale : 0 = Peu probable ; 10 = Très probable]**<br>1. cdH<br>2. DéFI<br>3. Ecolo<br>4. MR<br>5. PS<br>6. PTB	0	10
[If region = Flanders] w3_Prospective_FL	**If there were elections for the Chamber today, which party would you vote for?**<br>1. CD&V<br>2. Groen<br>3. N-VA<br>4. Open VLD<br>5. PvdA<br>6. sp.a<br>7. Vlaams Belang<br>8. Other<br>9. Blank<br>10. Not eligible to vote (yet)<br>11. I don't know.	**Stel dat er morgen verkiezingen voor de Kamer zouden zijn, op welke van de volgende partijen zou u dan stemmen?**<br>1. CD&V<br>2. Groen<br>3. N-VA<br>4. Open VLD<br>5. PvdA<br>6. sp.a<br>7. Vlaams Belang<br>8. Andere<br>9. Blanco<br>10. Mag (nog) niet stemmen<br>11. Weet ik niet	**S'il y avait des élections à la Chambre aujourd'hui, pour quel parti voteriez-vous?**<br>1. CD&V<br>2. Groen<br>3. N-VA<br>4. Open VLD<br>5. PvdA<br>6. sp.a<br>7. Vlaams Belang<br>8. Autre<br>9. Blanc<br>10. Ne peut pas (encore) voter<br>11. Je ne sais pas	1	11

[If region = Wallonia] w3_Prospective_Wal<br><br>*(Note: Parti Populaire not listed anymore, as no longer relevant)*	**If there were elections for the Chamber today, which party would you vote for?**<br>1. cdH<br>2. DéFI<br>3. Ecolo<br>4. MR<br>6. PS<br>7. PTB<br>8. Autre<br>9. Blank<br>10. Cannot (yet) vote<br>11. I don't know.	**Stel dat er morgen verkiezingen voor de Kamer zouden zijn, op welke van de volgende partijen zou u dan stemmen?**<br>1. cdH<br>2. DéFI<br>3. Ecolo<br>4. MR<br>6. PS<br>7. PTB<br>8. Andere<br>9. Blanco<br>10. Mag (nog) niet stemmen<br>11. Weet ik niet	**S'il y avait des élections à la Chambre aujourd'hui, pour quel parti voteriez-vous?**<br>1. cdH<br>2. DéFI<br>3. Ecolo<br>4. MR<br>6. PS<br>7. PTB<br>8. Autre<br>9. Blanc<br>10. Ne peut pas (encore) voter<br>11. Je ne sais pas	1	11
w3_Issue_salience_1<br>To<br>w3_Issue_salience_2	**Can you indicate what would be the most important issues in determining your vote if the elections were held now? You can indicate a maximum of two themes. [1 = Employment; 2 = Environment; 3 = Crime; 4 = Migration; 5 = Economy; 6 = State Reform; 7 = Defense; 8 = Taxes; 9 = Social Security; 10 = Functioning of Democracy; 11 = Public Health]**<br>**1.** Theme 1<br>2. Theme 2	**Kunt u aangeven welke van deze thema's het belangrijkst zijn bij het maken van uw stemkeuze als er nu verkiezingen zouden zijn? U kunt maximaal twee thema's aanduiden door het dropdownmenu aan te klikken. [1 = Werkgelenheid; 2 = Milieu; 3 = Criminaliteit; 4 = Migratie; 5 = Economie; 6 = Staatshervormingen; 7 = Defensie; 8 = Belastingen; 9 = Sociale zekerheid; 10 = Functioneren van de democratie; 11 = Volksgezondheid]**<br>1. Thema 1<br>2. Thema 2	**Pouvez-vous indiquer quels seraient les thèmes les plus importants pour déterminer votre vote si les élections avaient lieu maintenant? Vous pouvez indiquer un maximum de deux thèmes. [1 = Emploi; 2 = Environnement ; 3 = Criminalité ; 4 = Migration ; 5 = Economie ; 6 = Réforme de L'Etat ; 7 = Défense ; 8 = Impôts ; 9 = Sécurite Sociale ; 10 = Fonctionnement de la démocratie ; 11 = La santé de la population]**<br>1. Thème 1<br>2. Thème 2	1	11

[If region = Flanders]<br>w3_LR_parties_FLA_1<br>To<br>w3_LR_parties_FLA_7	**In politics, the terms 'left' and 'right' are often used. On a scale from 0 to 10, can you tell us where you think the following parties stand? 0 is the most left, 10 is the most right, and 5 is the centre. [0-10 scale: 0 = Left; 5 = Center; 10 = Right]**<br>1. CD&V<br>2. Groen<br>3. N-VA<br>4. Open VLD<br>5. PVDA<br>6. Sp.a<br>7. Vlaams Belang	**In de politiek worden vaak de termen 'links' en 'rechts' gebruikt. Kunt u op een schaal van 0 tot 10 aangeven waar volgens u de volgende partijen staan, waarbij 0 'links', 5 'in het centrum' en 10 'rechts' betekent? [0-10 scale: 0 = Links; 5 = In het midden; 10 = Rechts]**<br>1. CD&V<br>2. Groen<br>3. N-VA<br>4. Open VLD<br>5. PVDA<br>6. Sp.a<br>7. Vlaams Belang	**En politique, on utilise souvent les termes de 'gauche' et de 'droite'. Pouvez-vous sur une échelle allant de 0 à 10 nous dire où se situent d'après vous les partis suivants ? 0 est la position la plus à gauche, 10 la plus à droite, et 5 est le centre. [0-10 scale : 0 = Gauche ; 5 = Le centre ; 10 = Droite]**<br>1. CD&V<br>2. Groen<br>3. N-VA<br>4. Open VLD<br>5. PVDA<br>6. Sp.a<br>7. Vlaams Belang	0	10
[If region = Wallonia]<br>w3_LR_parties_WAL_1<br>To<br>w3_LR_parties_WAL_7	**In politics, the terms 'left' and 'right' are often used. On a scale from 0 to 10, can you tell us where you think the following parties stand? 0 is the most left, 10 is the most right, and 5 is the centre. [0-10 scale: 0 = Left; 5 = Center; 10 = Right]**<br>1. cdH<br>2. DéFI<br>3. Ecolo<br>4. MR<br>5. PP<br>6. PS<br>7. PTB	**In de politiek worden vaak de termen 'links' en 'rechts' gebruikt. Kunt u op een schaal van 0 tot 10 aangeven waar volgens u de volgende partijen staan, waarbij 0 'links', 5 'in het centrum' en 10 'rechts' betekent? [0-10 scale: 0 = Links; 5 = In het midden; 10 = Rechts]**<br>1. cdH<br>2. DéFI<br>3. Ecolo<br>4. MR<br>5. PP<br>6. PS<br>7. PTB	**En politique, on utilise souvent les termes de 'gauche' et de 'droite'. Pouvez-vous sur une échelle allant de 0 à 10 nous dire où se situent d'après vous les partis suivants ? 0 est la position la plus à gauche, 10 la plus à droite, et 5 est le centre. [0-10 scale : 0 = Gauche ; 5 = Le centre ; 10 = Droite]**<br>1. cdH<br>2. DéFI<br>3. Ecolo<br>4. MR<br>5. PP<br>6. PS<br>7. PTB	0	10

w3_Feel_Repres_1 To w3_Feel_Repres_2	**Can you indicate the extent to which you agree with the following statements? [0-10 scale: 0 = Totally disagree; 10 = Totally agree]**<br>**1.** I feel that there is a political party and/or a politician who shares most of my opinions.<br>2. I find some of my most important concerns in the manifesto of a political party**.**	**Kunt u aangeven in hoeverre u het eens bent met onderstaande stellingen? [0-10 scale: 0 = Helemaal oneens; 10 = Helemaal eens]**<br>1. Ik heb het gevoel dat er een politieke partij en/of politicus is die een groot deel van mijn opinies deelt.<br>2. Een aantal zaken die ik echt belangrijk vind, vind ik terug in het programma van een politieke partij.	**Pouvez-vous indiquer dans quelle mesure vous êtes en accord avec les affirmations suivantes ? [0-10 scale : 0 = Pas du tout d’accord ; 10 = Tout à fait d’accord]**<br>1. J'ai le sentiment qu'il y a un parti politique et/ou un homme ou une femme politique qui partage une grande partie de mes opinions.<br>2. Je retrouve une partie de mes préoccupations les plus importantes dans le programme d'un parti politique.	0	10

w3_Feel_Rep_new_1 To w3_Feel_Rep_new_11	**To what extent do you agree with the following proposals? [1-5 scsle: 1 = Totally disagree; 2 = Somewhat disagree; 3 = Neither agree nor disagree; 4 = Somewhat agree; 5 = Totally agree]**<br>**1.** There is not a single politician or political party who cares about me.<br>2. I agree with most of the policies that are put in place by politicians and political parties.<br>3. Most politicians and parties care/are concerned about ordinary citizens.<br>4. Most politicians and parties don't really know what my bigest worries are.<br>5. There are politicians and parties who care about ordinary citizens.<br>6. There is not a single politician who shares my interests and political preferences.<br>7. The political proposals of most politicians and parties are not in line with the preferences of citizens.<br>8. There are politicians and parties who understand my interests.<br>9. Most politicians and parties do not know what citizens want.	**En in welke mate bent u het eens met onderstaande stellingen? [1-5 scale: 1 = Helemaal oneens; 2 = Eerder oneens; 3 = Noch eens, noch oneens; 4 = Eerder eens; 5 = Helemaal eens]**<br>1. Er is geen enkele politicus of politieke partij die om mij geeft.<br>2. Ik ben het eens met het grootste deel van het beleid dat door de politici en politieke partijen wordt gevoerd.<br>3. De meeste politici en partijen bekommeren zich om gewone burgers.<br>4. De meeste politici of politieke partijen weten niet echt wat mijn grootste zorgen zijn.<br>5. Er zijn politici of partijen die geven om gewone burgers.<br>6. Er is geen enkele politicus of partij die mijn belangen en voorkeuren naar behoren verdedigt.<br>7. De beleidsvoorstellen van de meeste politici en partijen zijn niet in overeenstemming met de	**Dans quelle mesure êtes-vous d'accord avec les propositions suivantes ? [1-5 scsle : 1 = Pas du tout d'accord ; 2 = Plutôt pas d'accord ; 3 = Ni d'accord, ni pas d'accord ; 4 = Plutôt d'accord ; 5 = Tout à fait d'accord]**<br>1. Il n'y a pas un seul homme ou une seule femme politique qui se soucie de moi/s'intéresse à moi.<br>2. Je suis d'accord avec la plupart des politiques qui sont mises en place par les hommes et femmes politiques et par les partis politiques.<br>3. La plupart des hommes et femmes politiques et des partis se soucient/préoccupent des citoyens ordinaires.<br>4. La plupart des hommes et femmes politiques et des partis ne savent pas ce que mes besoins/soucis sont.<br>5. Il y a des hommes et femmes politiques et des partis qui se soucient des citoyens ordinaires.<br>6. Il n'y a pas un seul homme ou une seule femme politique	1	5

	10. Politicians or parties that defend my interests are often not able to get their proposals accepted.<br>11. There are politicians and parties that act the way citizens want them to act.	voorkeuren van de burgers.<br>8. Er zijn politici of partijen die mijn belangen begrijpen.<br>9. De meeste politici en partijen weten niet wat de burgers willen.<br>10. De politici of partijen die mijn belangen verdedigen slagen er vaak niet in om hun beleidsvoorstellen geaccepteerd te krijgen.<br>11. Er zijn politici of partijen die handelen in overeenstemming met wat burgers willen dat ze doen.	qui partage mes intérêts et mes préférences politiques.<br>7. Les propositions politiques de la plupart des hommes et femmes politiques et des partis ne sont pas en accord avec les préférences des citoyens.<br>8. Il y a des hommes et femmes politiques et des partis qui comprennent mes besoins/intérêts.<br>9. La plupart des hommes et femmes politiques et des partis ne savent pas ce que les citoyens veulent.<br>10. Les hommes et femmes politiques ou les partis qui partagent mes préoccupations ne réussissent le plus souvent pas à mettre en œuvre leur programme et leurs propositions politiques.<br>11. Il y a des hommes et femmes politiques et des partis qui s'agissent comme les citoyens veulent qu'ils le fassent.		
w3_Feel_rep_open1	**Can you name a politician whom you feel he or she represents you particularly well?**	**Kunt u een politicus noemen door wie u zich bijzonder vertegenwoordigd voelt?**	**Pouvez-vous citer un homme politique ou une femme politique dont vous avez le sentiment qu'il ou elle vous représente bien ?**	TXT	TXT

w3_Feel_rep_open2	**Can you explain why this is the case?**	**Kunt u uitleggen waarom dit het geval is?**	**Pouvez-vous expliquer pourquoi c'est le cas ?**	TXT	TXT
w3_Sat_dem	**In general, are you rather satisfied or rather dissatisfied with the way democracy works in Belgium?**<br>1. Very satisfied<br>2. Somewhat satisfied<br>3. Neither satisfied nor dissatisfied<br>4. Somewhat dissatisfied<br>5. Very dissatisfied	**In het algemeen, bent u eerder tevreden of eerder ontevreden over de manier waarop de democratie in België werkt?**<br>1. Heel tevreden<br>2. Eerder tevreden<br>3. Noch tevreden, noch ontevreden<br>4. Eerder ontevreden<br>5. Heel ontevreden	**En général, êtes-vous plutôt satisfait(e) ou plutôt insatisfait(e) de la façon dont la démocratie fonctionne en Belgique ?**<br>1. Très satisfait(e)<br>2. Plutôt satisfait(e)<br>3. Ni satisfait(e), ni insatisfait(e)<br>4. Plutôt insatisfait(e)<br>5. Très insatisfait(e)	1	5
w3_Trust_Pol_1<br>To<br>w3_Trust_Pol_4	**Can you indicate the extent to which you agree with the statements below? [0-10 scale: 0 = No trust at all, 10 = Full trust]**<br>1. Political Parties<br>2. The Federal Parliament<br>3. Politicians<br>4. The European Union	**Kunt u op een schaal van 0 tot 10 aangeven hoeveel vertrouwen u persoonlijk heeft in elk van de volgende instellingen? [0-10 scale: 0 = Helemaal geen vertrouwen, 10 = Volledig vertrouwen]**<br>1. De politieke partijen<br>2. Het federale parlement<br>3. Politici<br>4. De Europese Unie	**Pouvez-vous indiquer dans quelle mesure vous êtes d'accord avec les affirmations ci-dessous ? [0-10 scale : 0 = Absolument pas confiance, 10 = Totalement confiance]**<br>1. Les partis politiques<br>2. Le parlement fédéral<br>3. Les hommes/femmes politiques<br>4. L'Union européenne	0	10

w3_Cynisme_1<br>To<br>w3_Cynisme_7	**Can you indicate the extent to which you agree with the statements below? [1-5 scale: 1 = Totally disagree; 2 = Somewhat disagree; 3 = Neither agree nor disagree; 4 = Somewhat agree; 5 = Totally agree]**<br>1. Politicians are corrupt.<br>2. Most politicians are competent.<br>3. Politicians are trying to keep their promises.<br>4. Politicians do not understand what is going on in society.<br>5. Many politicians have been around too long.<br>6. The way we organize elections in this country is fair.<br>7. Political parties take sufficient account of independent experts when making decisions.	**Kunt u aangeven in hoeverre u het eens bent met onderstaande stellingen? (1-5 scale: 1 = Helemaal oneens; 2 = Eerder oneens; 3 = Noch eens, noch oneens; 4 = Eerder eens; 5 = Helemaal eens]**<br>1. Politici zijn corrupt.<br>2. De meeste politici zijn competent.<br>3. Politici proberen hun beloftes te houden.<br>4. Politici begrijpen niet wat er speelt in de samenleving.<br>5. Veel politici draaien al te lang mee.<br>6. De manier waarop we verkiezingen organiseren in dit land is eerlijk.<br>7. Partijen houden voldoende rekening met onafhankelijke experten wanneer ze beleid maken.	**Pouvez-vous indiquer dans quelle mesure vous êtes d'accord avec les affirmations ci-dessous ? [1-5 scale : 1 = Pas du tout d'accord ; 2 = Plutôt pas d'accord ; 3 = Ni d'accord, ni pas d'accord ; 4= Plutôt d'accord ; 5 = Tout à fait d'accord]**<br>1. Les hommes/femmes politiques sont corrompus.<br>2. La plupart des hommes/femmes politiques sont compétents.<br>3. Les hommes/femmes politiques essaient de tenir leurs promesses.<br>4. Les hommes/femmes politiques ne comprennent pas ce qui se passe dans la société.<br>5. Beaucoup d'hommes/femmes politiques sont là depuis trop longtemps.<br>6. La façon dont nous organisons les élections dans ce pays est juste.<br>7. Les partis politiques tiennent suffisamment compte des experts indépendants lorsqu'ils prennent des décisions.	1	5
w3_Populisme_1<br>to	**Can you indicate the extent to which you agree with the**	**Kunt u aangeven in hoeverre u het eens bent met onderstaande**	**Pouvez-vous indiquer dans quelle mesure vous êtes d'accord avec les**	1	5

w3_Populisme_7	**statements below? [1-5 scale: 1 = Totally disagree; 2 = Somewhat disagree; 3 = Neither agree nor disagree; 4 = Somewhat agree; 5 = Totally agree]**<br>**1.** Politicians must follow the advice of the people.<br>2. Political opposition is greater between elites and citizens than between citizens among themselves.<br>3. I would rather be represented by an ordinary citizen than by a professional politician .<br>4. When making decisions, politicians care about people like me.<br>5. Politics is the result of compromise and common sense.<br>6. Rich citizens have more influence on politics than poor citizens.<br>7. In general, politics reflects people's preferences fairly well.	**stellingen? (1-5 scale: 1 = Helemaal oneens; 2 = Eerder oneens; 3 = Noch eens, noch oneens; 4 = Eerder eens; 5 = Helemaal eens]**<br>1. Politici in het parlement moeten zich laten leiden door de mening van het volk.<br>2. De politieke tegenstellingen tussen de elite en de gewone burgers zijn groter dan die tussen burgers onderling.<br>3. Ik word liever vertegenwoordigd door een gewone burger dan door een beroepspoliticus.<br>4. Bij het nemen van besluiten geven politici om mensen zoals ik.<br>5. Beleid is doorgaans het resultaat van compromis en gezond verstand.<br>6. Rijke burgers hebben meer invloed op het beleid dan arme burgers.<br>7. Over het algemeen weerspiegelt het beleid de voorkeuren van het volk redelijk goed.	**affirmations ci-dessous? [1-5 scale : 1 = Pas du tout d'accord ; 2 = Plutôt pas d'accord ; 3 = Ni d'accord, ni pas d'accord ; 4= Plutôt d'accord ; 5 = Tout à fait d'accord]**<br>1. Les hommes et femmes politiques doivent suivre l'avis de la population.<br>2. L'opposition politique est plus grande entre les élites et les citoyens, qu'entre les citoyens entre eux.<br>3. Je préfère être représenté par un(e) citoyen(ne) ordinaire que par un homme/ femme politique professionnel(le)..<br>4. En prenant des décisions, les hommes /femmes politiques se soucient des gens comme moi.<br>5. La politique est le résultat du compromis et du bon sens.<br>6. Les citoyens riches ont plus d'influence sur les politiques que les citoyens pauvres.<br>7. En général, la politique reflète assez bien les préférences de la population.		

w3_Efficacy_1<br>To<br>w3_Efficacy_9	**Can you indicate the extent to which you agree with the statements below? [1-5 scale: 1 = Totally disagree; 2 = Somewhat disagree; 3 = Neither agree nor disagree; 4 = Somewhat agree; 5 = Totally agree]**<br>1. Most citizens do not have a clear political preference.<br>2. Political parties do not offer the population real political alternatives.<br>3. Political parties leave too much to the campaign advisers to determine important political issues.<br>4. The influence of interest groups and lobbies on policies is too great.<br>5. Going to the polls is useless, parties always do what they want.<br>6. In general, our political system works honestly.<br>7. Our political decision-making processes are sufficiently transparent.<br>8. In general, our political system functions effectively.<br>9. I feel that I have a fairly good understanding of important political issues in Belgium.	**Kunt u aangeven in hoeverre u het eens bent met onderstaande stellingen? [1-5 scale: 1 = Helemaal oneens; 2 = Eerder oneens; 3 = Noch eens, noch oneens; 4 = Eerder eens; 5 = Helemaal eens]**<br>1. De meeste burgers hebben geen duidelijke beleidsvoorkeuren.<br>2. Politieke partijen bieden het volk geen echt verschillende beleidsalternatieven.<br>3. Politieke partijen laten campagneadviseurs teveel hun visie over politieke thema's bepalen.<br>4. De invloed van belangengroepen en lobbyisten op het beleid is te groot.<br>5. Gaan stemmen is zinloos, partijen doen toch wat ze zelf willen.<br>6. In het algemeen werkt ons politiek systeem op een eerlijke manier.<br>7. Onze politieke besluitvormingsprocessen zijn voldoende transparant.	**Pouvez-vous indiquer dans quelle mesure vous êtes d'accord avec les affirmations ci-dessous ? [1-5 scale : 1 = Pas du tout d'accord ; 2 = Plutôt pas d'accord ; 3 = Ni d'accord, ni pas d'accord ; 4= Plutôt d'accord ; 5 = Tout à fait d'accord]**<br>1. La plupart des citoyens n'ont pas de préférence politique claire.<br>2. Les partis politiques n'offrent pas à la population de véritables alternatives politiques.<br>3. Les partis politiques laissent trop les conseillers de campagne déterminer les thèmes politiques importants.<br>4. L'influence des groupes d'intérêts et des lobbys sur les politiques est trop grande.<br>5. Aller voter ne sert à rien, les partis font toujours ce qu'ils veulent.<br>6. En général, notre système politique fonctionne de manière honnête.<br>7. Nos processus de décision politique sont suffisamment transparents.	1	5

		8. In het algemeen werkt ons politiek systeem op een efficiënte manier.<br>9. Ik heb het gevoel dat ik redelijk goed begrijp wat de belangrijke politieke vraagstukken in België zijn.	8. En général, notre système politique fonctionne efficacement.<br>9. J'ai le sentiment d'avoir une assez bonne compréhension des questions politiques importantes en Belgique.		
w3_Eval_DemProc_1 To w3_Eval_DemProc_9	**In Belgium, democracy is organized according to a number of legal rules and informal practices. We will introduce you to a few. For each of these rules that organize democracy in Belgium, can you tell us what you think? [1 = Very negative; 2 = Somewhat negative; 3 = Somewhat positive; 4 = Very positive; 99 = I do not know/no opinion]**<br>1. Free elections are held every five years to appoint the members who will sit in Parliament.<br>2. The governments are composed of coalitions between several parties. These parties must negotiate after the elections to find an agreement in order to form a government together.	**In België is de democratie geregeld door verschillende wettelijke regels en informele gewoonten. Hieronder leggen we er een aantal aan u voor. We zouden graag uw mening hierover kennen. Kan u voor elk van deze regels of gewoonten aangeven hoe u er over denkt? [1-4 scale: 1 = Heel negatief; 2 = Eerder negatief; 3 = Eerder positief; 4 = Heel positief; 99 = Weet niet/Geen mening]**<br>1. Elke vijf jaar worden er vrije verkiezingen georganiseerd om de parlementsleden te verkiezen.<br>2. Regeringen zijn coalities tussen verschillende partijen. Na de verkiezingen moeten deze partijen onderhandelen om tot een overeenkomst	**En Belgique, la démocratie est organisée selon un certain nombre de règles légales et de pratiques informelles. Nous allons vous en présenter quelques-unes. Pour chacune de ces règles qui organisent la démocratie en Belgique, pouvez-vous nous dire ce que vous en pensez ? [1-4 scale : 1 = Très négatif ; 2 = Plutôt négatif ; 3 = Plutôt positif ; 4 = Très positif ; 99 = Je ne sais pas/sans opinion]**<br>1. Des élections libres sont organisées tous les cinq ans pour désigner les député(es) qui siègeront au Parlement.<br>2. Les gouvernements sont des coalitions entre plusieurs partis. Ces partis doivent négocier après les élections pour se mettre d’accord pour former un gouvernement ensemble.	1	4

	3. Apart from voting during elections, citizens are currently not involved in the political decision-making processes.<br>4. In Belgium, there are currently 50 ministers and 467 parliamentarians split across three levels of government (federal, regional and community level).<br>5. In Belgium, the net monthly income of a federal member is between 5500 and 6600 net euros per month.<br>6. Currently, it is possible to be at the same time member of parliament, mayor and alderman.<br>7. Currently, it is the party leaders who decide who will be proposed to become minister.<br>8. In Belgium, in public companies, the Board of Directors is composed of persons designated by the political parties.<br>**9.** Currently in Belgium, political power is divided into five levels of	te komen, en zo samen een regering te vormen.<br>3. Momenteel zijn burgers zelden betrokken bij politieke beslissingen, behalve bij de verkiezingen.<br>4. In België zijn er momenteel 47 ministers en 467 parlementsleden, verspreid over 3 beleidsniveaus (federaal, gemeenschap en gewest).<br>5. In België ligt het nettoloon van een federale volksvertegenwoordiger tussen 5500 en 6000 euro per maand.<br>6. Op dit ogenblik is het mogelijk om op hetzelfde moment zowel parlementslid als burgemeester of schepen te zijn.<br>7. Momenteel beslissen de partijvoorzitters wie er minister wordt.<br>8. In Belgische overheidsbedrijven is de Raad van Bestuur samengesteld uit personen die benoemd	3. Actuellement, en dehors des élections les citoyens sont rarement impliqués dans la prise de décision politique.<br>4. En Belgique, il y a actuellement 47 ministres, et 467 parlementaires répartis à 3 niveaux de pouvoir (fédéral, régional, communautaire).<br>5. En Belgique, le salaire mensuel net d'un(e) député(e) fédéral(e) se situe entre 5500 et 6000 euros net par mois.<br>6. Actuellement, il est possible d'être en même temps député et bourgmestre ou échevin.<br>7. Actuellement, ce sont les présidents de parti qui décident qui sera proposé pour devenir ministre.<br>8. En Belgique, dans les entreprises publiques, le Conseil d'Administration est composé de personnes désignées par les partis politiques.<br>9. Actuellement, en Belgique, le pouvoir politique est distribué entre cinq niveaux de pouvoir : les communes,		

	government: the communes, the provinces, the regions, the communities and the federal state.	zijn door de politieke partijen.<br>9. De politieke macht is in België verdeeld over vijf bevoegdheidsniveaus: het lokale niveau, de provincies, de gemeenschappen en gewesten, en het federale niveau.	les provinces, les régions, les communautés, et l'état fédéral.		
w3_Sup_Reforms_1 To w3_Sup_Reforms_9	**Currently, several propositions for reform of the Belgian democracy are being debated. We will introduce you to a few. For each of these reforms, could you indicate if you are completely against, somewhat against, somewhat in favour, or totally in favour?** **[1 = Totally against; 2 = Somewhat against; 3 = Somewhat in favour; 4 = Totally in favour; 99 = I do not know/no opinion]**<br>1. Federal, regional and european elections should be held every 10 years instead of every 5 years.<br>2. The election law should be changed to give the majority to a single party that could govern alone.<br>3. The number of ministers and parliamentarians in	**Op dit moment wordt er gediscussieerd over meerdere voorstellen om de Belgische democratie te hervormen. Kan u voor onderstaande voorstellen aangeven of u er volledig voor, eerder voor, eerder tegen, of volledig tegen bent?** **[1-4 scale: 1 = Helemaal tegen; 2 = Eerder tegen; 3 = Eerder voor; 4 = Helemaal voor; 99 = Weet niet/geen mening]**<br>1. Federale, regionale en Europese verkiezingen moeten elke 10 jaar worden georganiseerd in plaats van elke 5 jaar.<br>2. De kieswet moet veranderen zodat één partij een meerderheid kan halen en dus alleen kan regeren.	**Plusieurs propositions de réformes de la démocratie belge sont débattues actuellement. Nous allons vous en citer quelques-unes. Pour chacune des réformes, pourriez-vous nous dire si vous êtes totalement pour, plutôt pour, plutôt contre, ou totalement contre ?** **[1-4 scale : 1 = Totalement contre ; 2 = Plutôt contre ; 3 = Plutôt pour ; 4 = Totalement pour ; 99 = Ne sais pas/sans opinion]**<br>1. Les élections fédérales, régionales et européennes devraient être organisées tous les 10 ans plutôt que tous les 5 ans.<br>2. La loi électorale devrait être changée pour donner la majorité à un seul parti qui pourrait gouverner seul.<br>3. Le nombre de ministres et de parlementaires en	1	4

	Belgium should be cut in half.<br>4. The income of the elected should be limited to a maximum of 2500 euros gross per month. *<br>5. Cumulating the mandates of mayor and member of parliament should be banned.<br>6. Ministers should be elected directly by the voters.<br>7. The appointments for inter-communal companies, public companies and administrations should be done by an agency independent from political parties.<br>8. Provinces should be abolished.<br>**9.** Experts should take the major political decisions instead of politicians.	3. Het aantal ministers en parlementsleden in België moet gehalveerd worden.<br>4. Het salaris van verkozenen moet beperkt worden tot maximaal €2500 per maand.<br>5. Het cumuleren van het mandaat van burgemeester en dat van volksvertegenwoordiger moet verboden worden.<br>6. Ministers moeten rechtstreeks door de kiezer worden verkozen.<br>7. Alle benoemingen in intercommunales, publieke bedrijven en bij de overheid moeten beslist worden door een agentschap dat onafhankelijk is van politieke partijen.<br>8. De provincies moeten worden afgeschaft.<br>9. Experten moeten de belangrijkste politieke beslissingen nemen in plaats van politici.	Belgique devrait être divisé par deux.<br>4. Le salaire des élus devrait être limité à maximum 2500 euros brut par mois.<br>5. Le cumul des mandats de bourgmestre et de député devrait être interdit.<br>6. Les ministres devraient être désignés directement par les électeurs.<br>7. Toutes les nominations dans les intercommunales, les entreprises publiques et l'administration devraient être faites par une agence indépendante des partis politiques.<br>8. Les provinces devraient être supprimées.<br>9. Des experts devraient prendre les principales décisions politiques à la place des hommes et femmes politiques.		
w3_Sup_Cit_Exp_1<br>To<br>w3_Sup_Cit_Exp_6	**For each one, can you, on a scale of 0 to 10, tell us how much you agree with the following**	**Kan u voor elk van de volgende stellingen aangeven, op een schaal van 0 tot 10, in welke mate u het eens of oneens bent met de**	**Pour chacune, pouvez-vous, sur une échelle allant de 0 à 10, nous dire à quel point vous êtes d'accord avec l'affirmation suivante ? [0-10 scale :**	0	10

	**statement?** **[0-10 scale: 0 = Totally disagree; 10 = Totally agree]**<br>1. Most citizens have all the skills required to make political decisions.<br>2. Most experts have all the skills needed to make political decisions.<br>3. Most citizens are honest.<br>4. Most experts are honest.<br>5. Most citizens are able to understand the needs of people like me.<br>6. Most experts are able to understand the needs of people like me.	**stelling?** **[0-10 scale: 0 = Helemaal oneens; 10 = Helemaal eens]**<br>1. De meeste burgers zijn competent om politieke beslissingen te nemen.<br>2. De meeste experts zijn competent om politieke beslissingen te nemen.<br>3. De meeste burgers zijn eerlijk.<br>4. De meeste experts zijn eerlijk.<br>5. De meeste burgers zijn in staat om de noden van personen zoals ik te begrijpen.<br>6. De meeste experts zijn in staat om de noden van personen zoals ik te begrijpen.	**0 = Pas du tout d'accord ; 10 = Tout à fait d'accord]**<br>1. La plupart des citoyens ont toutes les compétences requises pour prendre des décisions politiques.<br>2. La plupart des experts ont toutes les compétences requises pour prendre des décisions politiques.<br>3. La plupart des citoyens sont honnêtes.<br>4. La plupart des experts sont honnêtes.<br>5. La plupart des citoyens sont capables de comprendre les besoins de personnes comme moi.<br>6. La plupart des experts sont capables de comprendre les besoins de personnes comme moi.		
w3_Exp_Policy_Outcom_1 to w3_Exp_Policy_Outcom_5	**According to you, how likely is it that you will agree with the decisions taken on the following levels of government in the next 5 years?** **[0-10 scale: 0 = Very unlikely; 10 = Very likely; 99 = I do not know]**<br>1. At the european level<br>2. At the Belgian level<br>3. At the flemish level<br>4. At the Brussels level	**Hoe waarschijnlijk is het volgens u dat beslissingen zullen worden genomen waarmee u het eens bent op de volgende regeringsniveaus in de komende vijf jaar?** **[0-10 scale: 0 = Zeer onwaarschijnlijk; 10 = Zeer waarschijnlijk; 99 = Weet niet]**<br>1. Op Europees niveau<br>2. Op Belgisch niveau<br>3. Op Vlaams niveau	**Selon vous, quelle est la probabilité que vous soyez d'accord avec les décisions des niveaux de gouvernement suivants au cours des cinq prochaines années?** **[0-10 scale: 0 = Peu probable; 10 = Très probable; 99 = Je ne sais pas]**<br>1. Au niveau européen<br>2. Au niveau belge<br>3. Au niveau flamand<br>4. Au niveau bruxellois	0	10

	**5.** At the walloon level	4. Op Brussels niveau<br>5. Op Waals niveau	5. Au niveau wallon		
w3_Burgers_politici_1<br>to<br>w3_Burgers_politici_7	**To what extent are you in favour or against the following statement? "Important political decisions should more often be taken by ordinary citizens rather than by elected politicians". [0-10 scale: 0= Totally against; 10 = Totally in favour]**<br>1. At the european level<br>2. At the belgian level<br>3. At the flemish level<br>4. At the Brussels level<br>5. At the walloon level<br>**6.** At the local level	**In welke mate bent u voor of tegen het volgende? "Belangrijke politieke beslissingen zouden vaker gemaakt moeten worden door gewone burgers en niet door verkozen politici." [0-10 scale: 0 = Sterk tegen; 10 = Sterk voor]**<br>1. Op Europees niveau<br>2. Op Belgisch niveau<br>3. Op Vlaams niveau<br>4. Op Brussels niveau<br>5. Op Waals niveau<br>6. Op lokaal niveau	**Dans quelle mesure êtes-vous en faveur ou contre la proposition suivante? "Les décisions politiques importantes devraient être prises plus souvent par les citoyens ordinaires et non par les hommes et femmes politiques élus." [0-10 scale: 0 = Fortement contre; 10 = Fortement pour]**<br>1. Au niveau européen<br>2. Au niveau belge<br>3. Au niveau flamand<br>4. Au niveau bruxellois<br>5. Au niveau wallon<br>6. Au niveau local	0	10
w3_advis_ref	**Are you, in general, for or against consultative referendums on important national issues?** Citizens have the right to vote on a specific proposal. Parliament receives the voters' opinion but is not obliged to follow it. **[0-10 scale: 0 = Totally against; 10 = Totally in favour; 99 = Don't know]**	**Bent u, over het algemeen, voor of tegen adviserende referenda over belangrijke nationale thema's?** Burgers hebben dan het recht om een stem uit te brengen over een specifiek voorstel. Het parlement ontvangt het advies van de kiezers, maar is niet verplicht om dit te volgen. **[0-10 scale: 0 = Sterk tegen; 10 = Sterk voor; 99 = Weet niet]**	**Êtes-vous, en général, pour ou contre les référendums consultatifs sur les thèmes nationaux importants ?** Les citoyens ont le droit de voter sur une proposition spécifique. Le Parlement reçoit l'avis des électeurs mais sans être obligé de le suivre. **[0-10 scale : 0 = Totalement contre ; 10 = Totalement pour ; 99 = Je ne sais pas]**	0	10

w3_Bindend_ref	**Are you, in general, for or against binding referendums on important national issues?** Citizens have the right to vote on a specific proposal. Parliament is obliged to follow the opinion of the people. **[0-10 scale: 0 = Totally against; 10 = Totally in favour; 99 = Don't know]**	**Bent u, over het algemeen, voor of tegen bindende referenda over belangrijke nationale thema's?** Burgers hebben dan het recht om een stem uit te brengen over een specifiek voorstel. Het parlement is verplicht het advies van de kiezers te volgen. **[0-10 scale: 0 = Sterk tegen; 10 = Sterk voor; 99 = Weet niet]**	**Êtes-vous, en général, pour ou contre les référendums contraignants sur les thèmes nationaux importants ?** Les citoyens ont le droit de voter sur une proposition spécifique. Le Parlement est obligé de suivre l'avis de la population. **[0-10 scale : 0 = Fortement contre ; 10 = Fortement pour ; 99 = Je ne sais pas]**	0	10
w3_Advis_burgerforum	**Are you, in general, for or against the organisation of citizens' consultative forums on important national issues?** A citizens' forum is an assembly of about 30-50 citizens, drawn by lot, who meet and discuss a specific topic in order to produce a recommendation which is then forwarded to Parliament. **[0-10 scale: 0 = Totally against; 10 = Totally in favour; 99 = Don't know]**	**Bent u, over het algemeen, voor of tegen de organisatie van adviserende burgerfora over belangrijke nationale thema's?** Een burgerforum bestaat uit 30 tot 50 burgers, die op basis van toeval geselecteerd zijn. Ze komen samen en discussiëren over een bepaald thema om zo tot een advies aan het parlement te komen. **[0-10 scale: 0 = Sterk tegen; 10 = Sterk voor; 99 = Weet niet]**	**Êtes-vous, en général, pour ou contre l'organisation de forums consultatifs de citoyens sur les thèmes nationaux importants ?** Un forum citoyen est une assemblée composée d'environ 30 à 50 citoyens, tirés au sort, qui se rencontrent et qui discutent d'un sujet spécifique afin de produire une recommandation transmise ensuite au Parlement. **[0-10 scale : 0 = Fortement contre ; 10 = Fortement pour ; 99 = Je ne sais pas]**	0	10
w3_burgerbegroting	**Are you, in general, for or against participatory budgets at the national level?** A participatory budget means that citizens decide on a part of the Belgian state budget. The citizens involved meet and discuss how they want the money to be spent to support different concrete projects. **[0-10**	**Bent u, over het algemeen, voor of tegen burgerbegrotingen op nationaal niveau?** Een burgerbegroting betekent dat burgers beslissen over een deel van de Belgische begroting. De betrokken burgers komen samen en discussiëren over hoe ze het geld wensen te verdelen overheen een aantal concrete projecten. **[0-**	**Êtes-vous, en général, pour ou contre les budgets participatifs au niveau national ?** Un budget participatif signifie que des citoyens décident d'une partie du budget de l'Etat belge. Les citoyens impliqués se réunissent et discutent de la façon dont ils souhaitent que l'argent soit dépensé pour soutenir différents projets concrets. **[0-10 scale : 0 =**	0	10

	scale: 0 = Totally against; 10 = Totally in favour; 99 = Don't know]	10 scale: 0 = Sterk tegen; 10 = Sterk voor; 99 = Weet niet]	Fortement contre ; 10 = Fortement pour ; 99 = Je ne sais pas]		
w3_Experts_politic_1 to w3_Experts_politic_6	**To what extent do you support or oppose the following proposal?** Important political decisions should be taken more often by experts and not by elected politicians. **[0-10 scale: 0 = Totally oppose; 10 = Totally support; 99 = Don't know]** 1. At the European level 2. At the Belgian level 3. At the Flemish level 4. At the Walloon level 2. At the Brussels level **3.** At the local level	**In welke mate bent u voor of tegen de volgende stelling?** Belangrijke politieke beslissingen zouden vaker gemaakt moeten worden door experten en niet door verkozen politici. **[0-10 scale: 0 = Sterk tegen; 10 = Sterk voor; 99 = Weet niet]** **1.** Op Europees niveau **2.** Op Belgisch niveau 3. Op Vlaams niveau 4. Op Waals niveau 5. Op Brussels niveau 6. Op lokaal niveau	**Dans quelle mesure êtes-vous en faveur ou contre la proposition suivante ?** Les décisions politiques importantes devraient être prises plus souvent par des experts et non par des hommes et femmes politiques élus. **[0-10 scale : 0 = Fortement contre ; 10 = Fortement pour ; 99 = Je ne sais pas]** 1. Au niveau européen 2. Au niveau belge 3. Au niveau flamand 4. Au niveau wallon 5. Au niveau bruxellois 6. Au niveau local	0	10
w3_VAA_Statements_1 to w3_VAA_Statements_18	**Below you will find a series of proposals. Can you indicate the extent to which you agree or disagree with the following proposals? [1-4 scale: 1 = Totally Disagree; 2 = Disagree; 3 = Agree; 4 = Totally Agree]** **1.** Hosting migrants in transit should be a punishable offence. 2. Situational testing must be introduced to detect discrimination in employment.	**Hieronder vindt u enkele beleidsvoorstellen. Kan u voor elk van volgende beleidsvoorstellen aangeven in welke mate u het ermee eens of oneens bent? [1-4 scale: 1 = Helemaal oneens; 2 = Eerder oneens; 3 = Eerder eens; 4 = Helemaal eens]** 1. Transmigranten onderdak bieden moet strafbaar zijn. 2. De overheid moet praktijktesten doen om te controleren op	**Ci-dessous vous trouverez une série de propositions. Pouvez-vous indiquer dans quelle mesure vous êtes d'accord ou pas d'accord avec les propositions suivantes ? [1-4 scale : 1 = Pas du tout d'accord ; 2 = Pas d'accord ; 3 = D'accord ; 4 = Tout à fait d'accord]** 1. Héberger des migrants en transit doit être un délit punissable. 2. Il faut mettre en place des tests de situation pour détecter la discrimination à l'embauche.	1	4

	3. In order to be able to obtain Belgian nationality, one must first have passed an examination on European values.<br>4. If the asylum applications of families with children have been rejected, these families may be placed in detention pending repatriation.<br>5. By 2024, company cars running on petrol or diesel must be banned.<br>6. VAT on electricity must be reduced from 21% to 6%.<br>7. Airline tickets should be taxed to make them more expensive.<br>8. Nuclear power plants must remain operational after 2025.<br>9. You can't drive while drinking alcohol.<br>10. Abortion must also be allowed beyond 12 weeks of pregnancy.<br>11. It must no longer be possible to donate sperm anonymously.<br>12. Big money should be taxed more.<br>13. Wages should no longer be automatically indexed.	discriminatie bij aanwervingen.<br>3. Nieuwkomers moeten eerst slagen voor een examen over de Europese waarden voor ze de Belgische nationaliteit kunnen verwerven.<br>4. Als de asielaanvraag van gezinnen met kinderen is afgewezen, mogen ze in afwachting van hun terugkeer worden opgesloten.<br>5. Bedrijfswagens die op benzine of diesel rijden moeten voor 2024 verboden worden.<br>6. De btw op elektriciteit moet verlaagd worden van 21 naar 6%.<br>7. Vliegen moet duurder worden door de tickets te belasten.<br>8. Ook na 2025 moeten we kerncentrales openhouden.<br>9. Als je rijdt mag je helemaal geen alcohol gedronken hebben.<br>10. Ook na 12 weken zwangerschap moet	3. Pour pouvoir obtenir la nationalité belge, il faut d'abord avoir réussi un examen sur les valeurs européennes.<br>4. Si la demande d'asile de familles avec enfants a été rejetée, ces familles peuvent être placées en détention en attendant leur rapatriement.<br>5. D'ici 2024, les voitures de société qui roulent à l'essence ou au diesel doivent être interdites.<br>6. La TVA sur l'électricité doit être réduite de 21 à 6%.<br>7. Il faut taxer les billets d'avion pour qu'ils soient plus chers.<br>8. Les centrales nucléaires doivent rester opérationnelles après 2025.<br>9. On ne peut pas conduire en ayant bu de l'alcool.<br>10. L'avortement doit aussi être autorisé au-delà de 12 semaines de grossesse.<br>11. Le don de sperme ne doit plus pouvoir être anonyme.<br>12. Les grandes fortunes doivent être plus taxées.		

	14. The fingerprints of all citizens should be stored in a central database.<br>15. Stores must be able to choose when they do their sales.<br>16. A retirement pension of at least €1500 per month should be introduced.<br>17. The government must have as many men as women.<br>18. It must be possible to leave important political decisions to the citizens by means of a referendum.	abortus nog toegestaan worden.<br>11. Spermadonatie mag niet langer anoniem gebeuren.<br>12. Grote vermogens moeten meer worden belast.<br>13. De lonen mogen niet meer automatisch aan de prijsstijgingen worden aangepast (index).<br>14. Vingerafdrukken van alle burgers moeten in een centrale databank worden bijgehouden.<br>15. Winkels mogen zelf kiezen wanneer ze solden doen.<br>16. Er moet een minimumpensioen komen van minstens €1500 euro per maand.<br>17. Er moeten in de regering evenveel mannen als vrouwen zitten.<br>18. Belangrijke politieke beslissingen moeten via een referendum aan burgers kunnen worden overgelaten.	13. Les salaires ne doivent plus être automatiquement indexés.<br>14. Les empreintes digitales de tous les citoyens doivent être conservées dans une base de données centrale.<br>15. Les magasins doivent pouvoir choisir quand ils font leurs soldes.<br>16. Il faut instaurer une pension de retraite de 1500€ minimum par mois<br>17. Le gouvernement doit compter autant d'hommes que de femmes.<br>18. Les décisions politiques importantes doivent pouvoir être laissées aux citoyens par le biais d'un référendum.		

[If region = Flanders]<br><br>w3_Perc_PartyPos_FL_1_1 to w3_Perc_PartyPos_FL_18_7	**For each policy proposal, can you indicate the parties that you think agree with the proposal?** You can select more than one party. **[1 = CD&V; 2 = Groen; 3 = N-VA; 4 = Open VLD; 5 = PVDA; 6 = sp.a; 7 = Vlaams Belang]**<br>1. Hosting migrants in transit should be a punishable offence.<br>2. Situational testing must be introduced to detect discrimination in employment.<br>3. In order to be able to obtain Belgian nationality, one must first have passed an examination on European values.<br>4. If the asylum applications of families with children have been rejected, these families may be placed in detention pending repatriation.<br>5. By 2024, company cars running on petrol or diesel must be banned.<br>6. VAT on electricity must be reduced from 21% to 6%.<br>7. Airline tickets should be taxed to make them more expensive.	**Kunt u voor elk beleidsvoorstel de partijen aanduiden waarvan u denkt dat ze het met het voorstel eens zijn?** U kunt voor elk voorstel meerdere partijen aanklikken. **[1 = CD&V; 2 = Groen; 3 = N-VA; 4 = Open VLD; 5 = PVDA; 6 = sp.a; 7 = Vlaams Belang]**<br>1. Transmigranten onderdak bieden moet strafbaar zijn.<br>2. De overheid moet praktijktesten doen om te controleren op discriminatie bij aanwervingen.<br>3. Nieuwkomers moeten eerst slagen voor een examen over de Europese waarden voor ze de Belgische nationaliteit kunnen verwerven.<br>4. Als de asielaanvraag van gezinnen met kinderen is afgewezen, mogen ze in afwachting van hun terugkeer worden opgesloten.<br>5. Bedrijfswagens die op benzine of diesel rijden moeten voor 2024 verboden worden.	**Pour chaque proposition de politique, pouvez-vous indiquer les partis qui, selon vous, sont en accord avec la proposition ?** Vous pouvez sélectionner plusieurs partis. **[1 = CD&V; 2 = Groen; 3 = N-VA; 4 = Open VLD; 5 = PVDA; 6 = sp.a; 7 = Vlaams Belang]**<br>1. Héberger des migrants en transit doit être un délit punissable.<br>2. Il faut mettre en place des tests de situation pour détecter la discrimination à l'embauche.<br>3. Pour pouvoir obtenir la nationalité belge, il faut d'abord avoir réussi un examen sur les valeurs européennes.<br>4. Si la demande d'asile de familles avec enfants a été rejetée, ces familles peuvent être placées en détention en attendant leur rapatriement.<br>5. D'ici 2024, les voitures de société qui roulent à l'essence ou au diesel doivent être interdites.<br>6. La TVA sur l'électricité doit être réduite de 21 à 6%.	1	7

	8. Nuclear power plants must remain operational after 2025.<br>9. You can't drive while drinking alcohol.<br>10. Abortion must also be allowed beyond 12 weeks of pregnancy.<br>11. It must no longer be possible to donate sperm anonymously.<br>12. Big money should be taxed more.<br>13. Wages should no longer be automatically indexed.<br>14. The fingerprints of all citizens should be stored in a central database.<br>15. Stores must be able to choose when they do their sales.<br>16. A retirement pension of at least €1500 per month should be introduced.<br>17. The government must have as many men as women.<br>18. It must be possible to leave important political decisions to the citizens by means of a referendum.	6. De btw op elektriciteit moet verlaagd worden van 21 naar 6%.<br>7. Vliegen moet duurder worden door de tickets te belasten.<br>8. Ook na 2025 moeten we kerncentrales openhouden.<br>9. Als je rijdt mag je helemaal geen alcohol gedronken hebben.<br>10. Ook na 12 weken zwangerschap moet abortus nog toegestaan worden.<br>11. Spermadonatie mag niet langer anoniem gebeuren.<br>12. Grote vermogens moeten meer worden belast.<br>13. De lonen mogen niet meer automatisch aan de prijsstijgingen worden aangepast (index).<br>14. Vingerafdrukken van alle burgers moeten in een centrale databank worden bijgehouden.<br>15. Winkels mogen zelf kiezen wanneer ze solden doen.<br>16. Er moet een minimumpensioen komen	7. Il faut taxer les billets d'avion pour qu'ils soient plus chers.<br>8. Les centrales nucléaires doivent rester opérationnelles après 2025.<br>9. On ne peut pas conduire en ayant bu de l'alcool.<br>10. L'avortement doit aussi être autorisé au-delà de 12 semaines de grossesse.<br>11. Le don de sperme ne doit plus pouvoir être anonyme.<br>12. Les grandes fortunes doivent être plus taxées.<br>13. Les salaires ne doivent plus être automatiquement indexés.<br>14. Les empreintes digitales de tous les citoyens doivent être conservées dans une base de données centrale.<br>15. Les magasins doivent pouvoir choisir quand ils font leurs soldes.<br>16. Il faut instaurer une pension de retraite de 1500€ minimum par mois<br>17. Le gouvernement doit compter autant d'hommes que de femmes.<br>18. Les décisions politiques importantes doivent pouvoir		

		van minstens €1500 euro per maand.<br>17. Er moeten in de regering evenveel mannen als vrouwen zitten.<br>18. Belangrijke politieke beslissingen moeten via een referendum aan burgers kunnen worden overgelaten.	être laissées aux citoyens par le biais d'un référendum.		
[If region = Wallonia] w3_Perc_PartyPos_ WAL_1_1 to w3_Perc_PartyPos_ WAL_18_7	**For each policy proposal, can you indicate the parties that you think agree with the proposal?** You can select more than one party**.** **[1 =cdH; 2 =DéFI; 3 = Ecolo; 4 = MR; 6 = PS; 7 = PTB]**<br>1. Hosting migrants in transit should be a punishable offence.<br>2. Situational testing must be introduced to detect discrimination in employment.<br>3. In order to be able to obtain Belgian nationality, one must first have passed an examination on European values.<br>4. If the asylum applications of families with children have been rejected, these families may be placed in	**Kunt u voor elk beleidsvoorstel de partijen aanduiden waarvan u denkt dat ze het met het voorstel eens zijn?** U kunt voor elk voorstel meerdere partijen aanklikken. **[1 =cdH; 2 =DéFI; 3 = Ecolo; 4 = MR; 6 = PS; 7 = PTB]**<br>1. Transmigranten onderdak bieden moet strafbaar zijn.<br>2. De overheid moet praktijktesten doen om te controleren op discriminatie bij aanwervingen.<br>3. Nieuwkomers moeten eerst slagen voor een examen over de Europese waarden voor ze de Belgische nationaliteit kunnen verwerven.<br>4. Als de asielaanvraag van gezinnen met kinderen is	**Pour chaque proposition de politique, pouvez-vous indiquer les partis qui, selon vous, sont en accord avec la proposition ?** Vous pouvez sélectionner plusieurs partis. **[1 =cdH; 2 =DéFI; 3 = Ecolo; 4 = MR; 6 = PS; 7 = PTB]**<br>1. Héberger des migrants en transit doit être un délit punissable.<br>2. Il faut mettre en place des tests de situation pour détecter la discrimination à l'embauche.<br>3. Pour pouvoir obtenir la nationalité belge, il faut d'abord avoir réussi un examen sur les valeurs européennes.<br>4. Si la demande d'asile de familles avec enfants a été rejetée, ces familles peuvent être placées en détention en	1	7

	detention pending repatriation.<br>5. By 2024, company cars running on petrol or diesel must be banned.<br>6. VAT on electricity must be reduced from 21% to 6%.<br>7. Airline tickets should be taxed to make them more expensive.<br>8. Nuclear power plants must remain operational after 2025.<br>9. You can't drive while drinking alcohol.<br>10. Abortion must also be allowed beyond 12 weeks of pregnancy.<br>11. It must no longer be possible to donate sperm anonymously.<br>12. Big money should be taxed more.<br>13. Wages should no longer be automatically indexed.<br>14. The fingerprints of all citizens should be stored in a central database.<br>15. Stores must be able to choose when they do their sales.	afgewezen, mogen ze in afwachting van hun terugkeer worden opgesloten.<br>5. Bedrijfswagens die op benzine of diesel rijden moeten voor 2024 verboden worden.<br>6. De btw op elektriciteit moet verlaagd worden van 21 naar 6%.<br>7. Vliegen moet duurder worden door de tickets te belasten.<br>8. Ook na 2025 moeten we kerncentrales openhouden.<br>9. Als je rijdt mag je helemaal geen alcohol gedronken hebben.<br>10. Ook na 12 weken zwangerschap moet abortus nog toegestaan worden.<br>11. Spermadonatie mag niet langer anoniem gebeuren.<br>12. Grote vermogens moeten meer worden belast.<br>13. De lonen mogen niet meer automatisch aan de prijsstijgingen worden aangepast (index).	attendant leur rapatriement.<br>5. D'ici 2024, les voitures de société qui roulent à l'essence ou au diesel doivent être interdites.<br>6. La TVA sur l'électricité doit être réduite de 21 à 6%.<br>7. Il faut taxer les billets d'avion pour qu'ils soient plus chers.<br>8. Les centrales nucléaires doivent rester opérationnelles après 2025.<br>9. On ne peut pas conduire en ayant bu de l'alcool.<br>10. L'avortement doit aussi être autorisé au-delà de 12 semaines de grossesse.<br>11. Le don de sperme ne doit plus pouvoir être anonyme.<br>12. Les grandes fortunes doivent être plus taxées.<br>13. Les salaires ne doivent plus être automatiquement indexés.<br>14. Les empreintes digitales de tous les citoyens doivent être conservées dans une base de données centrale.<br>15. Les magasins doivent pouvoir choisir quand ils font leurs soldes.		

	16. A retirement pension of at least €1500 per month should be introduced.<br>17. The government must have as many men as women.<br>18. It must be possible to leave important political decisions to the citizens by means of a referendum.	14. Vingerafdrukken van alle burgers moeten in een centrale databank worden bijgehouden.<br>15. Winkels mogen zelf kiezen wanneer ze solden doen.<br>16. Er moet een minimumpensioen komen van minstens €1500 euro per maand.<br>**17.** Er moeten in de regering evenveel mannen als vrouwen zitten.<br>**18.** Belangrijke politieke beslissingen moeten via een referendum aan burgers kunnen worden overgelaten.	16. Il faut instaurer une pension de retraite de 1500€ minimum par mois<br>**17.** Le gouvernement doit compter autant d'hommes que de femmes.<br>**18.** Les décisions politiques importantes doivent pouvoir être laissées aux citoyens par le biais d'un référendum		
w3_EU_1	**To what extent do you agree with the following statement:**<br><br>The European Union leaves sufficient room for the Belgian government to conduct economic policy. **[1-5 scale: 1 = Totally disagree; 2 = Rather disagree; 3 = Neither disagree, nor agree; 4 = Rather agree; 5 = Totally agree]**	**In welke mate bent u het eens met de volgende stelling:**<br><br>De Europese Unie laat voldoende ruimte aan de Belgische overheid om economisch beleid te voeren. **[1-5 scale: 1 = Helemaal oneens; 2 = Eerder oneens; 3 = Noch oneens, noch eens; 4 = Eerder eens; 5 = Helemaal eens]**	**Dans quelle mesure êtes-vous d'accord avec la phrase suivante :**<br><br>L'Union européenne laisse assez de marge de manœuvre au gouvernement belge dans le domaine économique. **[1-5 scale : 1 = Pas du tout d'accord ; 2 = Plutôt pas d'accord ; 3 = Ni d'accord, ni pas d'accord ; 4 = Plutôt d'accord ; 5 = Tout à fait d'accord]**	1	5

w3_EU_2	**To what extent is the European Union responsible for economic conditions in Belgium? Please indicate your position using a number on a scale of 0 to 10, where 0 means "no responsibility" and 10 means "full responsibility". [0-10 scale: 0 = No responsibility; 10 = Total responsibility]**	**In welke mate vindt u dat de Europese Unie verantwoordelijk is voor de economische toestand in België? Geef uw mening op een schaal van 0 tot 10, waarbij 0 "niet verantwoordelijk" betekent, en 10 "volledig verantwoordelijk". [0-10 scale: 0 = Niet verantwoordelijk; 10 = Volledig verantwoordelijk]**	**Dans quelle mesure l'Union européenne est-elle responsable des conditions économiques en Belgique ? Veuillez indiquer votre position en utilisant un chiffre sur une échelle de 0 à 10, 0 signifiant "aucune responsabilité" et 10 signifiant "responsabilité totale". [0-10 scale : 0 = Aucune responsabilité ; 10 = Responsabilité totale]**	0	10
w3_Glob_1	**To what extent do you agree with the following statement:**<br><br>Globalization leaves sufficient room for the Belgian government to conduct economic policy. **[1-5 scale: 1 = Totally disagree; 2 = Rather disagree; 3 = Neither disagree, nor agree; 4 = Rather agree; 5 = Totally agree]**	**In welke mate bent u het eens met de volgende stelling:**<br><br>De globalisering laat voldoende ruimte aan de Belgische overheid om economisch beleid te voeren. **[1-5 scale: 1 = Helemaal oneens; 2 = Eerder oneens; 3 = Noch oneens, noch eens; 4 = Eerder eens; 5 = Helemaal eens]**	**Dans quelle mesure êtes-vous d'accord avec la phrase suivante:**<br><br>La mondialisation laisse assez de marge de manœuvre au gouvernement belge dans le domaine économique. **[1-5 scale : 1 = Pas du tout d'accord ; 2 = Plutôt pas d'accord ; 3 = Ni d'accord, ni pas d'accord ; 4 = Plutôt d'accord ; 5 = Tout à fait d'accord]**	1	5
w3_Glob_2	**As far as the economy is concerned, to what extent is the Belgian government responsible for economic conditions in Belgium? Please indicate your position using a number on a scale of 0 to 10, where 0 means "no responsibility" and 10 means "full responsibility". [0-10 scale: 0 = No**	**In welke mate vindt u dat de Belgische overheid verantwoordelijk is voor de economische toestand in België? Geef uw mening op een schaal van 0 tot 10, waarbij 0 "niet verantwoordelijk" betekent, en 10 "volledig verantwoordelijk". [0-10 scale: 0 = Niet**	**En ce qui concerne l'économie, dans quelle mesure le gouvernement belge est-il responsable des conditions économiques en Belgique ? Veuillez indiquer votre position en utilisant un chiffre sur une échelle de 0 à 10, 0 signifiant "aucune responsabilité" et 10 signifiant "responsabilité totale". [0-**	0	10

	responsibility; 10 = Total responsibility]	verantwoordelijk; 10 = Volledig verantwoordelijk]	10 scale : 0 = Aucune responsabilité ; 10 = Responsabilité totale]		

4.4 Wave 4 questionnaire

Variable name	Label + Values [English]	Label + Values [Dutch]	Label + Values [French]	Min	Max
w4_Region	**In which region is your main residence located?**<br>1. Brussels Capital Region<br>2. Flemish Region<br>3. Walloon Region	**In welk gewest heeft u uw hoofdverblijfplaats?**<br>1. Brussels Hoofdstedelijk Gewest<br>2. Vlaams Gewest<br>3. Waals Gewest	**Dans quelle région se trouve votre lieu de résidence principale ?**<br>1. Région de Bruxelles-Capitale<br>2. Région flamande<br>3. Région wallonne	1	3
w4_Duration	**Duration of respondent's time to complete the questionnaire of wave 3 (in seconds)**			110	1206299
w4_RecordedDate	**Date on which the respondent's response for wave 3 was recorded**			18 May 2021	4 June 2021
w4_Q_Language	**Language setting for the respondent in wave 4**<br>0. French<br>1. Dutch			0	1
w4_Consent	**Did respondent agree with the informed consent form?**<br>1. Yes<br>2. No			1	1
w4_Age_control	**What is your age?**	**Wat is uw leeftijd?**	**Quel âge avez-vous?**	18	110
w4_Corona _1<br>To<br>w4_Corona_3	**Below we provide you with some statements about the Corona / COVID-19 pandemic. For each statement, can you indicate to what extent you agree with or disagree? [1-7 scale: 1 = Totally disagree; 4 = Neither agree nor disagree; 7 = Totally agree]**<br>1. I'm worried when I think about the coronavirus.<br>2. I am afraid that my personal financial	**Hieronder leggen we u enkele stellingen omtrent de Corona / COVID-19 pandemie voor. Kan u voor elke stelling aangeven in welke mate u het ermee eens, dan wel oneens bent? [1-7 scale: 1 = Helemaal oneens; 4 = Noch eens, noch oneens; 7 = Helemaal eens]**<br>1. Ik voel mij ongerust als ik aan het coronavirus denk.<br>2. Ik ben bang dat mijn persoonlijke financiele situatie slechter zal worden	**Nous vous présenterons ci-dessous quelques observations sur la pandémie de Corona / COVID-19. Pour chaque thèse, pouvez-vous indiquer dans quelle mesure vous êtes d'accord ou non? [1-7 scale: 1 = Complètement en désaccord; 4 = Ni l'un, ni l'autre; 7 = Complètement d'accord]**<br>1. Je m'inquiète quand je pense au coronavirus.<br>2. Je crains que ma situation financière personnelle ne	1	7

	situation will be worsened by the measures surrounding the coronavirus outbreak.<br>3. I am concerned about the health of my family and myself.	door de maatregelen rondom de uitbraak van het coronavirus.<br>3. Ik maak mij zorgen over de gezondheid van mijn familie en mijzelf.	s'aggrave à cause des mesures prises à la suite de l'épidémie de coronavirus.<br>3. Je m'inquiète pour la santé de ma famille et de moi-même.		
[If region = Flanders] w4_VoteFed19_Fl	**For which party did you vote for <u>the Chamber</u> during the national elections on the 26th of May 2019?**<br>1. CD&V<br>2. Groen<br>3. N-VA<br>4. Open VLD<br>5. PVDA<br>6. sp.a<br>7. Vlaams Belang<br>8. Other<br>9. Blank or invalid<br>10. Did not vote<br>11. Was not (yet) eligible to vote<br>12. I do not remember	**Op welke partij stemde u voor <u>de Kamer</u> bij de afgelopen verkiezingen van 26 mei 2019?**<br>1. CD&V<br>2. Groen<br>3. N-VA<br>4. Open VLD<br>5. PVDA<br>6. sp.a<br>7. Vlaams Belang<br>8. Andere<br>9. Blanco of ongeldig gestemd<br>10. Niet gaan stemmen<br>11. Mocht (nog) niet stemmen<br>12. Ik weet het niet meer	**Pour quel parti avez-vous voté <u>à la Chambre des Représentants</u> lors des élections fédérales du 26 mai 2019 ?**<br>1. CD&V<br>2. Groen<br>3. N-VA<br>4. Open VLD<br>5. PVDA<br>6. sp.a<br>7. Vlaams Belang<br>8. Autre<br>9. J'ai voté blanc / invalide<br>10. Je n'ai pas voté<br>11. Je ne pouvais pas (encore) voter<br>12. Je ne m'en rappelle pas	1	12
[If region = Wallonia] w4_VoteFed19_Wal	**For which party did you vote for <u>the Chamber</u> during the national elections on the 26th of May 2019?**<br>1. cdH<br>2. DéFI<br>3. Ecolo	**Op welke partij stemde u voor <u>de Kamer</u> bij de afgelopen verkiezingen van 26 mei 2019?**<br>1. cdH<br>2. DéFI<br>3. Ecolo<br>4. MR	**Pour quel parti avez-vous voté <u>à la Chambre des Représentants</u> lors des élections fédérales du 26 mai 2019 ?**<br>1. cdH<br>2. DéFI<br>3. Ecolo	1	12

	4. MR<br>5. PP<br>6. PS<br>7. PTB<br>8. Other<br>9. Blank or invalid<br>10. I did not vote<br>11. I could not (yet) vote<br>12. I do not remember	5. PP<br>6. PS<br>7. PTB<br>8. Andere<br>9. Blanco of ongeldig gestemd<br>10. Niet gaan stemmen<br>11. Mocht (nog) niet stemmen<br>12. Ik weet het niet meer	4. MR<br>5. PP<br>6. PS<br>7. PTB<br>8. Autre<br>9. J'ai voté blanc / invalide<br>10. Je n'ai pas voté<br>11. Je ne pouvais pas (encore) voter<br>12. Je ne m'en rappelle pas		
w4_Win_lose_elec	**If you recall the federal elections that took place on 26 May 2019, would you say that the party you voted for won or lost the elections?**<br><br>**The party I voted for in the federal election...**<br><br>1. Won the election.<br>2. Lost the election.<br>99. I don't know**.**	**Als u terugdenkt aan de federale verkiezingen die plaatsvonden op 26 mei 2019, zou u zeggen dat de partij waarop u heeft gestemd de verkiezingen heeft gewonnen of verloren?**<br><br>**De partij waarop ik stemde tijdens de federale verkiezingen heeft…**<br><br>1. de verkiezingen heeft gewonnen.<br>2. de verkiezingen heeft verloren.<br>99. Weet ik niet.	**Si vous pensez aux élections fédérales du 26 mai 2019, diriez-vous que le parti sur lequel vous avez voté a gagné ou perdu les élections?**<br><br>**Le parti pour lequel j'ai voté aux élections fédérales...**<br><br>1. a gagné les élections.<br>2. a perdu les élections.<br>99. Je ne sais pas.	1	2
w4_Win_lose_form	**If you remember the formation of the government that led to the present federal government, you would say that the party you voted for won or lost the formation.**<br><br>**The party I voted for...**	**Als u terugdenkt aan de regeringsformatie die resulteerde in de huidige federale regering, zou u zeggen dat de partij waarop u heeft gestemd de formatie heeft gewonnen of verloren.**<br><br>**De partij waarop ik stemde heeft…**	**Si vous pensez à la formation du gouvernement qui a donné naissance à l'actuel gouvernement fédéral, vous diriez que le parti pour lequel vous avez voté a gagné ou perdu la formation.**<br><br>**Le parti pour lequel j'ai voté...**	1	2

	1. Won the election.<br>2. Lost the election.<br>99. I don't know.	1. de verkiezingen heeft gewonnen.<br>2. de verkiezingen heeft verloren.<br>99. Weet ik niet.	1. a gagné les élections.<br>2. a perdu les élections<br>99. Je ne sais pas.		
w4_Win_lose_Reg	**We just asked you about your feelings about the formation of the government for the federal government. If you now remember the formation of the government that resulted in the current Flemish Government, you would say that the party you voted for won or lost the formation.**<br><br>**The party I voted for...**<br><br>1. Won the election.<br>2. Lost the election.<br>99. I don't know.	**We vroegen u net naar uw gevoel over de regeringsformatie voor de federale regering. Als u nu terugdenkt aan de regeringsformatie die resulteerde in de huidige [region] regering, zou u zeggen dat de partij waarop u heeft gestemd de formatie heeft gewonnen of verloren.**<br><br>**De partij waarop ik stemde heeft...**<br><br>1. de verkiezingen heeft gewonnen.<br>2. de verkiezingen heeft verloren.<br>99. Weet ik niet.	**Nous venons de vous demander ce que vous pensiez de la formation du gouvernement fédéral. Si vous vous souvenez de la formation gouvernementale qui a mené à l'actuel gouvernement flamand, vous diriez que le parti pour lequel vous avez voté a gagné ou perdu la formation.**<br><br>**Le parti pour lequel j'ai voté...**<br><br>1. a gagné les élections.<br>2. a perdu les élections<br>99. Je ne sais pas.	1	2

[If region = Flanders] w4_ElecUtil_Fl_1 To w4_ElecUtil_Fl _7	**Different political parties are active in Flanders. Can you indicate how likely it is that you will one day vote for the following parties in the next elections? [0-10 scale: 0 = Not very likely; 10 = Very likely]**<br>1. CD&V<br>2. Groen<br>3. N-VA<br>4. Open VLD<br>5. PVDA<br>6. Vooruit (former Sp.a)<br>7. Vlaams Belang	**In Vlaanderen zijn verschillende politieke partijen actief. Kunt u aangeven hoe waarschijnlijk het is dat u in toekomstige verkiezingen ooit voor de onderstaande partijen zou stemmen? [0-10 scale: 0 = Heel onwaarschjinlijk; 10 = Heel waarschijnlijk]**<br>1. CD&V<br>2. Groen<br>3. N-VA<br>4. Open VLD<br>5. PVDA<br>6. Vooruit (vroeger Sp.a)<br>7. Vlaams Belang	**Différents partis politiques sont actifs en Flandre. Pouvez-vous indiquer dans quelle mesure il est probable que vous voterez un jour pour les partis suivants lors de prochaines élections ? [0-10 scale : 0 = Peu probable ; 10 = Très probable]**<br>1. CD&V<br>2. Groen<br>3. N-VA<br>4. Open VLD<br>5. PVDA<br>6. Vooruit (ancien Sp.a)<br>7. Vlaams Belang	0	10
[If region = Wallonia] w4_ElecUtil_Wal_1 to w4_ElecUtil_Wal_6	**Different political parties are active in Wallonia. Can you indicate how likely it is that you will one day vote for the following parties in the next elections? [0-10 scale: 0 = Unlikely; 10 = Very likely]**<br>1. cdH<br>2. DéFI<br>3. Ecolo<br>4. MR<br>5. PS<br>6. PTB	**In Wallonië zijn verschillende politieke partijen actief. Kunt u aangeven hoe waarschijnlijk het is dat u in toekomstige verkiezingen ooit voor de onderstaande partijen zou stemmen? [0-10 scale: 0 = Heel onwaarschijnlijk; 10 = Heel waarschijnlijk]**<br>1. cdH<br>2. DéFI<br>3. Ecolo<br>4. MR<br>5. PS<br>6. PTB	**Différents partis politiques sont actifs en Wallonie. Pouvez-vous indiquer dans quelle mesure il est probable que vous voterez un jour pour les partis suivants lors de prochaines élections ? [0-10 scale : 0 = Peu probable ; 10 = Très probable]**<br>1. cdH<br>2. DéFI<br>3. Ecolo<br>4. MR<br>5. PS<br>6. PTB	0	10

[If region = Flanders]<br>w4_Prospective_FL	**If there were elections for the Chamber today, which party would you vote for?**<br>1. CD&V<br>2. Groen<br>3. N-VA<br>4. Open VLD<br>5. PvdA<br>6. Vooruit (former sp.a)<br>7. Vlaams Belang<br>8. Other<br>9. Blank<br>10. Not eligible to vote (yet)<br>11. I don't know.	**Stel dat er morgen verkiezingen voor de Kamer zouden zijn, op welke van de volgende partijen zou u dan stemmen?**<br>1. CD&V<br>2. Groen<br>3. N-VA<br>4. Open VLD<br>5. PvdA<br>6. Vooruit (vroeger sp.a)<br>7. Vlaams Belang<br>8. Andere<br>9. Blanco<br>10. Mag (nog) niet stemmen<br>11. Weet ik niet	**S'il y avait des élections à la Chambre aujourd'hui, pour quel parti voteriez-vous?**<br>1. CD&V<br>2. Groen<br>3. N-VA<br>4. Open VLD<br>5. PvdA<br>6. Vooruit (ancien sp.a)<br>7. Vlaams Belang<br>8. Autre<br>9. Blanc<br>10. Ne peut pas (encore) voter<br>11. Je ne sais pas	1	11
[If region = Wallonia]<br>w4_Prospective_Wal<br><br>*(Note: Parti Populaire not listed anymore, as no longer relevant)*	**If there were elections for the Chamber today, which party would you vote for?**<br>1. cdH<br>2. DéFI<br>3. Ecolo<br>4. MR<br>5. PS<br>6. PTB<br>7. Autre<br>8. Blank<br>9. Cannot (yet) vote<br>10. I don't know.	**Stel dat er morgen verkiezingen voor de Kamer zouden zijn, op welke van de volgende partijen zou u dan stemmen?**<br>1. cdH<br>2. DéFI<br>3. Ecolo<br>4. MR<br>5. PS<br>6. PTB<br>7. Andere<br>8. Blanco<br>9. Mag (nog) niet stemmen<br>10. Weet ik niet	**S'il y avait des élections à la Chambre aujourd'hui, pour quel parti voteriez-vous?**<br>1. cdH<br>2. DéFI<br>3. Ecolo<br>4. MR<br>5. PS<br>6. PTB<br>7. Autre<br>8. Blanc<br>9. Ne peut pas (encore) voter<br>10. Je ne sais pas	1	11

w4_Issue_salience_1 To w4_Issue_salience_2	**Can you indicate what would be the most important issues in determining your vote if the elections were held now? You can indicate a maximum of two themes. [1 = Employment; 2 = Environment; 3 = Crime; 4 = Migration; 5 = Economy; 6 = State Reform; 7 = Defense; 8 = Taxes; 9 = Social Security; 10 = Functioning of Democracy; 11 = Public Health]**<br>1. Theme 1<br>2. Theme 2	**Kunt u aangeven welke van deze thema's het belangrijkst zijn bij het maken van uw stemkeuze als er nu verkiezingen zouden zijn? U kunt maximaal twee thema's aanduiden door het dropdownmenu aan te klikken. [1 = Werkgelenheid; 2 = Milieu; 3 = Criminaliteit; 4 = Migratie; 5 = Economie; 6 = Staatshervormingen; 7 = Defensie; 8 = Belastingen; 9 = Sociale zekerheid; 10 = Functioneren van de democratie; 11 = Volksgezondheid]**<br>1. Thema 1<br>2. Thema 2	**Pouvez-vous indiquer quels seraient les thèmes les plus importants pour déterminer votre vote si les élections avaient lieu maintenant? Vous pouvez indiquer un maximum de deux thèmes. [1 = Emploi; 2 = Environnement ; 3 = Criminalité ; 4 = Migration ; 5 = Economie ; 6 = Réforme de L'Etat ; 7 = Défense ; 8 = Impôts ; 9 = Sécurite Sociale ; 10 = Fonctionnement de la démocratie ; 11 = La santé de la population]**<br>1. Thème 1<br>2. Thème 2	1	11
w4_Feel_Repres_1 & w4_Feel_Repres_2	**Can you indicate the extent to which you agree with the following statements? [0-10 scale: 0 = Totally disagree; 10 = Totally agree]**<br>1. I feel that there is a political party and/or a politician who shares most of my opinions.<br>2. I find some of my most important concerns in the manifesto of a political party.	**Kunt u aangeven in hoeverre u het eens bent met onderstaande stellingen? [0-10 scale: 0 = Helemaal oneens; 10 = Helemaal eens]**<br>1. Ik heb het gevoel dat er een politieke partij en/of politicus is die een groot deel van mijn opinies deelt.<br>2. Een aantal zaken die ik echt belangrijk vind, vind ik terug in het programma van een politieke partij.	**Pouvez-vous indiquer dans quelle mesure vous êtes en accord avec les affirmations suivantes ? [0-10 scale : 0 = Pas du tout d'accord ; 10 = Tout à fait d'accord]**<br>1. J'ai le sentiment qu'il y a un parti politique et/ou un homme ou une femme politique qui partage une grande partie de mes opinions.<br>2. Je retrouve une partie de mes préoccupations les plus importantes dans le programme d'un parti politique.	0	10

w4_Feel_Rep_new_1<br>To<br>w4_Feel_Rep_new_11	**To what extent do you agree with the following proposals? [1-5 scsle: 1 = Totally disagree; 2 = Somewhat disagree; 3 = Neither agree nor disagree; 4 = Somewhat agree; 5 = Totally agree]**<br>1. There is not a single politician or political party who cares about me.<br>2. I agree with most of the policies that are put in place by politicians and political parties.<br>3. Most politicians and parties care/are concerned about ordinary citizens.<br>4. Most politicians and parties don't really know what my biggest worries are.<br>5. There are politicians and parties who care about ordinary citizens.<br>6. There is not a single politician who shares my interests and political preferences.<br>7. The political proposals of most politicians and parties are not in line with the preferences of citizens.<br>8. There are politicians and parties who understand my interests.<br>9. Most politicians and parties do not know what citizens want.	**En in welke mate bent u het eens met onderstaande stellingen? [1-5 scale: 1 = Helemaal oneens; 2 = Eerder oneens; 3 = Noch eens, noch oneens; 4 = Eerder eens; 5 = Helemaal eens]**<br>1. Er is geen enkele politicus of politieke partij die om mij geeft.<br>2. Ik ben het eens met het grootste deel van het beleid dat door de politici en politieke partijen wordt gevoerd.<br>3. De meeste politici en partijen bekommeren zich om gewone burgers.<br>4. De meeste politici of politieke partijen weten niet echt wat mijn grootste zorgen zijn.<br>5. Er zijn politici of partijen die geven om gewone burgers.<br>6. Er is geen enkele politicus of partij die mijn belangen en voorkeuren naar behoren verdedigt.<br>7. De beleidsvoorstellen van de meeste politici en partijen zijn niet in overeenstemming met de voorkeuren van de burgers.	**Dans quelle mesure êtes-vous d'accord avec les propositions suivantes ? [1-5 scsle : 1 = Pas du tout d'accord ; 2 = Plutôt pas d'accord ; 3 = Ni d'accord, ni pas d'accord ; 4 = Plutôt d'accord ; 5 = Tout à fait d'accord]**<br>1. Il n'y a pas un seul homme ou une seule femme politique qui se soucie de moi/s'intéresse à moi.<br>2. Je suis d'accord avec la plupart des politiques qui sont mises en place par les hommes et femmes politiques et par les partis politiques.<br>3. La plupart des hommes et femmes politiques et des partis se soucient/préoccupent des citoyens ordinaires.<br>4. La plupart des hommes et femmes politiques et des partis ne savent pas ce que mes besoins/soucis sont.<br>5. Il y a des hommes et femmes politiques et des partis qui se soucient des citoyens ordinaires.<br>6. Il n'y a pas un seul homme ou une seule femme politique qui partage mes	1	5

	10. Politicians or parties that defend my interests are often not able to get their proposals accepted.<br>11. There are politicians and parties that act the way citizens want them to act.	8. Er zijn politici of partijen die mijn belangen begrijpen.<br>9. De meeste politici en partijen weten niet wat de burgers willen.<br>10. De politici of partijen die mijn belangen verdedigen slagen er vaak niet in om hun beleidsvoorstellen geaccepteerd te krijgen.<br>11. Er zijn politici of partijen die handelen in overeenstemming met wat burgers willen dat ze doen.	intérêts et mes préférences politiques.<br>7. Les propositions politiques de la plupart des hommes et femmes politiques et des partis ne sont pas en accord avec les préférences des citoyens.<br>8. Il y a des hommes et femmes politiques et des partis qui comprennent mes besoins/intérêts.<br>9. La plupart des hommes et femmes politiques et des partis ne savent pas ce que les citoyens veulent.<br>10. Les hommes et femmes politiques ou les partis qui partagent mes préoccupations ne réussissent le plus souvent pas à mettre en œuvre leur programme et leurs propositions politiques.<br>11. Il y a des hommes et femmes politiques et des partis qui s'agissent comme les citoyens veulent qu'ils le fassent.		
w4_Sat_dem	**In general, are you rather satisfied or rather dissatisfied with the way democracy works in Belgium?**<br>1. Very satisfied	**In het algemeen, bent u eerder tevreden of eerder ontevreden over de manier waarop de democratie in België werkt?**	**En général, êtes-vous plutôt satisfait(e) ou plutôt insatisfait(e) de la façon dont la démocratie fonctionne en Belgique ?**	1	5

	2. Somewhat satisfied<br>3. Neither satisfied nor dissatisfied<br>4. Somewhat dissatisfied<br>5. Very dissatisfied	1. Heel tevreden<br>2. Eerder tevreden<br>3. Noch tevreden, noch ontevreden<br>4. Eerder ontevreden<br>5. Heel ontevreden	1. Très satisfait(e)<br>2. Plutôt satisfait(e)<br>3. Ni satisfait(e), ni insatisfait(e)<br>4. Plutôt insatisfait(e)<br>5. Très insatisfait(e)		
w4_Emotions_1<br>To<br>w4_Emotions_8	**When you think about Belgian politics in general, to what extent do you experience each of the following emotions? [0-10 scale: 0 = Not at all; 10 = Extremely]**<br><br>1. Anger<br>2. Bitterness<br>3. Anxiety<br>4. Fear<br>5. Hope<br>6. Relief<br>7. Joy<br>8. Satisfaction	**Als u denkt aan de Belgische politiek in het algemeen, in welke mate ervaart u dan elk van de onderstaande emoties? [0-10 scale: 0 = Helemaal niet; 10 = In sterke mate]**<br><br>1. Boosheid<br>2. Bitterheid<br>3. Ongerustheid<br>4. Angst<br>5. Hoop<br>6. Opluchting<br>7. Blijdschap<br>8. Tevredenheid	**Si vous pensez à la politique belge en général, dans quelle mesure ressentez-vous chacune des émotions suivantes? [0-10 scale: 0 = Pas du tout; 10 = Extrêmement]**<br><br>1. Colère<br>2. Amertume<br>3. Anxiété<br>4. Peur<br>5. Espoir<br>6. Soulagement<br>7. Joie<br>8. Satisfaction	0	10
w4_Trust_Pol_1<br>to<br>w4_Trust_Pol_4	**Can you indicate the extent to which you agree with the statements below? [0-10 scale: 0 = No trust at all, 10 = Full trust]**<br>1. Political Parties<br>2. The Federal Parliament<br>3. Politicians<br>4. The European Union	**Kunt u op een schaal van 0 tot 10 aangeven hoeveel vertrouwen u persoonlijk heeft in elk van de volgende instellingen? [0-10 scale: 0 = Helemaal geen vertrouwen, 10 = Volledig vertrouwen]**<br>1. De politieke partijen<br>2. Het federale parlement<br>3. Politici<br>4. De Europese Unie	**Pouvez-vous indiquer dans quelle mesure vous êtes d'accord avec les affirmations ci-dessous ? [0-10 scale : 0 = Absolument pas confiance, 10 = Totalement confiance]**<br>1. Les partis politiques<br>2. Le parlement fédéral<br>3. Les hommes/femmes politiques<br>4. L'Union européenne	0	10

w4_Cynisme_1<br>To<br>w4_Cynisme_7	**Can you indicate the extent to which you agree with the statements below? [1-5 scale: 1 = Totally disagree; 2 = Somewhat disagree; 3 = Neither agree nor disagree; 4 = Somewhat agree; 5 = Totally agree]**<br>1. Politicians are corrupt.<br>2. Most politicians are competent.<br>3. Politicians are trying to keep their promises.<br>4. Politicians do not understand what is going on in society.<br>5. Many politicians have been around too long.<br>6. The way we organize elections in this country is fair.<br>7. Political parties take sufficient account of independent experts when making decisions.	**Kunt u aangeven in hoeverre u het eens bent met onderstaande stellingen? (1-5 scale: 1 = Helemaal oneens; 2 = Eerder oneens; 3 = Noch eens, noch oneens; 4 = Eerder eens; 5 = Helemaal eens]**<br>1. Politici zijn corrupt.<br>2. De meeste politici zijn competent.<br>3. Politici proberen hun beloftes te houden.<br>4. Politici begrijpen niet wat er speelt in de samenleving.<br>5. Veel politici draaien al te lang mee.<br>6. De manier waarop we verkiezingen organiseren in dit land is eerlijk.<br>7. Partijen houden voldoende rekening met onafhankelijke experten wanneer ze beleid maken.	**Pouvez-vous indiquer dans quelle mesure vous êtes d'accord avec les affirmations ci-dessous ? [1-5 scale : 1 = Pas du tout d'accord ; 2 = Plutôt pas d'accord ; 3 = Ni d'accord, ni pas d'accord ; 4= Plutôt d'accord ; 5 = Tout à fait d'accord]**<br>1. Les hommes/femmes politiques sont corrompus.<br>2. La plupart des hommes/femmes politiques sont compétents.<br>3. Les hommes/femmes politiques essaient de tenir leurs promesses.<br>4. Les hommes/femmes politiques ne comprennent pas ce qui se passe dans la société.<br>5. Beaucoup d'hommes/femmes politiques sont là depuis trop longtemps.<br>6. La façon dont nous organisons les élections dans ce pays est juste.<br>7. Les partis politiques tiennent suffisamment compte des experts indépendants lorsqu'ils prennent des décisions.	1	5

w4_Populisme_1<br>To<br>w4_Populisme_7	**Can you indicate the extent to which you agree with the statements below? [1-5 scale: 1 = Totally disagree; 2 = Somewhat disagree; 3 = Neither agree nor disagree; 4 = Somewhat agree; 5 = Totally agree]**<br>1. Politicians must follow the advice of the people.<br>2. Political opposition is greater between elites and citizens than between citizens among themselves.<br>3. I would rather be represented by an ordinary citizen than by a professional politician .<br>4. When making decisions, politicians care about people like me.<br>5. Politics is the result of compromise and common sense.<br>6. Rich citizens have more influence on politics than poor citizens.<br>7. In general, politics reflects people's preferences fairly well.	**Kunt u aangeven in hoeverre u het eens bent met onderstaande stellingen? (1-5 scale: 1 = Helemaal oneens; 2 = Eerder oneens; 3 = Noch eens, noch oneens; 4 = Eerder eens; 5 = Helemaal eens]**<br>1. Politici in het parlement moeten zich laten leiden door de mening van het volk.<br>2. De politieke tegenstellingen tussen de elite en de gewone burgers zijn groter dan die tussen burgers onderling.<br>3. Ik word liever vertegenwoordigd door een gewone burger dan door een beroepspoliticus.<br>4. Bij het nemen van besluiten geven politici om mensen zoals ik.<br>5. Beleid is doorgaans het resultaat van compromis en gezond verstand.<br>6. Rijke burgers hebben meer invloed op het beleid dan arme burgers.<br>7. Over het algemeen weerspiegelt het beleid de voorkeuren van het volk redelijk goed.	**Pouvez-vous indiquer dans quelle mesure vous êtes d'accord avec les affirmations ci-dessous? [1-5 scale : 1 = Pas du tout d'accord ; 2 = Plutôt pas d'accord ; 3 = Ni d'accord, ni pas d'accord ; 4= Plutôt d'accord ; 5 = Tout à fait d'accord]**<br>1. Les hommes et femmes politiques doivent suivre l'avis de la population.<br>2. L'opposition politique est plus grande entre les élites et les citoyens, qu'entre les citoyens entre eux.<br>3. Je préfère être représenté par un(e) citoyen(ne) ordinaire que par un homme/ femme politique professionnel(le)..<br>4. En prenant des décisions, les hommes /femmes politiques se soucient des gens comme moi.<br>5. La politique est le résultat du compromis et du bon sens.<br>6. Les citoyens riches ont plus d'influence sur les politiques que les citoyens pauvres.<br>7. En général, la politique reflète assez bien les	1	5

			préférences de la population.		
w4_Efficacy_1 To w4_Efficacy_9	**Can you indicate the extent to which you agree with the statements below? [1-5 scale: 1 = Totally disagree; 2 = Somewhat disagree; 3 = Neither agree nor disagree; 4 = Somewhat agree; 5 = Totally agree]**<br>1. Most citizens do not have a clear political preference.<br>2. Political parties do not offer the population real political alternatives.<br>3. Political parties leave too much to the campaign advisers to determine important political issues.<br>4. The influence of interest groups and lobbies on policies is too great.<br>5. Going to the polls is useless, parties always do what they want.<br>6. In general, our political system works honestly.<br>7. Our political decision-making processes are sufficiently transparent.<br>8. In general, our political system functions effectively.	**Kunt u aangeven in hoeverre u het eens bent met onderstaande stellingen? [1-5 scale: 1 = Helemaal oneens; 2 = Eerder oneens; 3 = Noch eens, noch oneens; 4 = Eerder eens; 5 = Helemaal eens]**<br>1. De meeste burgers hebben geen duidelijke beleidsvoorkeuren.<br>2. Politieke partijen bieden het volk geen echt verschillende beleidsalternatieven.<br>3. Politieke partijen laten campagneadviseurs teveel hun visie over politieke thema's bepalen.<br>4. De invloed van belangengroepen en lobbyisten op het beleid is te groot.<br>5. Gaan stemmen is zinloos, partijen doen toch wat ze zelf willen.<br>6. In het algemeen werkt ons politiek systeem op een eerlijke manier.<br>7. Onze politieke besluitvormingsprocessen zijn voldoende transparant.	**Pouvez-vous indiquer dans quelle mesure vous êtes d'accord avec les affirmations ci-dessous ? [1-5 scale : 1 = Pas du tout d'accord ; 2 = Plutôt pas d'accord ; 3 = Ni d'accord, ni pas d'accord ; 4= Plutôt d'accord ; 5 = Tout à fait d'accord]**<br>1. La plupart des citoyens n'ont pas de préférence politique claire.<br>2. Les partis politiques n'offrent pas à la population de véritables alternatives politiques.<br>3. Les partis politiques laissent trop les conseillers de campagne déterminer les thèmes politiques importants.<br>4. L'influence des groupes d'intérêts et des lobbys sur les politiques est trop grande.<br>5. Aller voter ne sert à rien, les partis font toujours ce qu'ils veulent.<br>6. En général, notre système politique fonctionne de manière honnête.	1	5

	9. I feel that I have a fairly good understanding of important political issues in Belgium.	8. In het algemeen werkt ons politiek systeem op een efficiënte manier.<br>9. Ik heb het gevoel dat ik redelijk goed begrijp wat de belangrijke politieke vraagstukken in België zijn.	7. Nos processus de décision politique sont suffisamment transparents.<br>8. En général, notre système politique fonctionne efficacement.<br>9. J'ai le sentiment d'avoir une assez bonne compréhension des questions politiques importantes en Belgique.		
**ULB**					
w4_Eval_DemProc_1 To w4_Eval_DemProc_9	**In Belgium, democracy is organized according to a number of legal rules and informal practices. We will introduce you to a few. For each of these rules that organize democracy in Belgium, can you tell us what you think? [1 = Very negative; 2 = Somewhat negative; 3 = Somewhat positive; 4 = Very positive; 99 = I do not know/no opinion]**<br>1. Free elections are held every five years to appoint the members who will sit in Parliament.<br>2. The governments are composed of coalitions between several parties. These parties must	**In België is de democratie geregeld door verschillende wettelijke regels en informele gewoonten. Hieronder leggen we er een aantal aan u voor. We zouden graag uw mening hierover kennen. Kan u voor elk van deze regels of gewoonten aangeven hoe u er over denkt? [1-4 scale: 1 = Heel negatief; 2 = Eerder negatief; 3 = Eerder positief; 4 = Heel positief; 99 = Weet niet/Geen mening]**<br>1. Elke vijf jaar worden er vrije verkiezingen georganiseerd om de parlementsleden te verkiezen.<br>2. Regeringen zijn coalities tussen verschillende partijen. Na de	**En Belgique, la démocratie est organisée selon un certain nombre de règles légales et de pratiques informelles. Nous allons vous en présenter quelques-unes. Pour chacune de ces règles qui organisent la démocratie en Belgique, pouvez-vous nous dire ce que vous en pensez ? [1-4 scale : 1 = Très négatif ; 2 = Plutôt négatif ; 3 = Plutôt positif ; 4 = Très positif ; 99 = Je ne sais pas/sans opinion]**<br>1. Des élections libres sont organisées tous les cinq ans pour désigner les député(es) qui siègeront au Parlement.<br>2. Les gouvernements sont des coalitions entre plusieurs partis. Ces partis	1	4

	negotiate after the elections to find an agreement in order to form a government together.<br>3. Apart from voting during elections, citizens are currently not involved in the political decision-making processes.<br>4. In Belgium, there are currently 50 ministers and 467 parliamentarians split across three levels of government (federal, regional and community level).<br>5. In Belgium, the net monthly income of a federal member is between 5500 and 6600 net euros per month.<br>6. Currently, it is possible to be at the same time member of parliament, mayor and alderman.<br>7. Currently, it is the party leaders who decide who will be proposed to become minister.<br>8. In Belgium, in public companies, the Board of Directors is composed of	verkiezingen moeten deze partijen onderhandelen om tot een overeenkomst te komen, en zo samen een regering te vormen.<br>3. Momenteel zijn burgers zelden betrokken bij politieke beslissingen, behalve bij de verkiezingen.<br>4. In België zijn er momenteel 47 ministers en 467 parlementsleden, verspreid over 3 beleidsniveaus (federaal, gemeenschap en gewest).<br>5. In België ligt het nettoloon van een federale volksvertegenwoordiger tussen 5500 en 6000 euro per maand.<br>6. Op dit ogenblik is het mogelijk om op hetzelfde moment zowel parlementslid als burgemeester of schepen te zijn.<br>7. Momenteel beslissen de partijvoorzitters wie er minister wordt.<br>8. In Belgische overheidsbedrijven is de Raad van Bestuur	doivent négocier après les élections pour se mettre d’accord pour former un gouvernement ensemble.<br>3. Actuellement, en dehors des élections, les citoyens sont rarement impliqués dans la prise de décision politique.<br>4. En Belgique, il y a actuellement 47 ministres, et 467 parlementaires répartis à 3 niveaux de pouvoir (fédéral, régional, communautaire).<br>5. En Belgique, le salaire mensuel net d’un(e) député(e) fédéral(e) se situe entre 5500 et 6000 euros net par mois.<br>6. Actuellement, il est possible d’être en même temps député et bourgmestre ou échevin.<br>7. Actuellement, ce sont les présidents de parti qui décident qui sera proposé pour devenir ministre.<br>8. En Belgique, dans les entreprises publiques, le Conseil d’Administration est composé de personnes		

	persons designated by the political parties.<br>9. Currently in Belgium, political power is divided into five levels of government: the communes, the provinces, the regions, the communities and the federal state.	samengesteld uit personen die benoemd zijn door de politieke partijen.<br>9. De politieke macht is in België verdeeld over vijf bevoegdheidsniveaus: het lokale niveau, de provincies, de gemeenschappen en gewesten, en het federale niveau.	désignées par les partis politiques.<br>9. Actuellement, en Belgique, le pouvoir politique est distribué entre cinq niveaux de pouvoir : les communes, les provinces, les régions, les communautés, et l'état fédéral.		
w4_Sup_Reforms_1 To w4_Sup_Reforms_9	**Currently, several propositions for reform of the Belgian democracy are being debated. We will introduce you to a few. For each of these reforms, could you indicate if you are completely against, somewhat against, somewhat in favour, or totally in favour? [1 = Totally against; 2 = Somewhat against; 3 = Somewhat in favour; 4 = Totally in favour; 99 = I do not know/no opinion]**<br>1. Federal, regional and european elections should be held every 10 years instead of every 5 years.<br>2. The election law should be changed to give the majority to a single party that could govern alone.<br>3. The number of ministers and parliamentarians in	**Op dit moment wordt er gediscussieerd over meerdere voorstellen om de Belgische democratie te hervormen. Kan u voor onderstaande voorstellen aangeven of u er volledig voor, eerder voor, eerder tegen, of volledig tegen bent? [1-4 scale: 1 = Helemaal tegen; 2 = Eerder tegen; 3 = Eerder voor; 4 = Helemaal voor; 99 = Weet niet/geen mening]**<br>1. Federale, regionale en Europese verkiezingen moeten elke 10 jaar worden georganiseerd in plaats van elke 5 jaar.<br>2. De kieswet moet veranderen zodat één partij een meerderheid kan halen en dus alleen kan regeren.	**Plusieurs propositions de réformes de la démocratie belge sont débattues actuellement. Nous allons vous en citer quelques-unes. Pour chacune des réformes, pourriez-vous nous dire si vous êtes totalement pour, plutôt pour, plutôt contre, ou totalement contre ? [1-4 scale : 1 = Totalement contre ; 2 = Plutôt contre ; 3 = Plutôt pour ; 4 = Totalement pour ; 99 = Ne sais pas/sans opinion]**<br>1. Les élections fédérales, régionales et européennes devraient être organisées tous les 10 ans plutôt que tous les 5 ans.<br>2. La loi électorale devrait être changée pour donner la majorité à un seul parti qui pourrait gouverner seul.	1	4

	Belgium should be cut in half.<br>4. The income of the elected should be limited to a maximum of 2500 euros gross per month. *<br>5. Cumulating the mandates of mayor and member of parliament should be banned.<br>6. Ministers should be elected directly by the voters.<br>7. The appointments for inter-communal companies, public companies and administrations should be done by an agency independent from political parties.<br>8. Provinces should be abolished.<br>9. Experts should take the major political decisions instead of politicians.	3. Het aantal ministers en parlementsleden in België moet gehalveerd worden.<br>4. Het salaris van verkozenen moet beperkt worden tot maximaal €2500 per maand.<br>5. Het cumuleren van het mandaat van burgemeester en dat van volksvertegenwoordiger moet verboden worden.<br>6. Ministers moeten rechtstreeks door de kiezer worden verkozen.<br>7. Alle benoemingen in intercommunales, publieke bedrijven en bij de overheid moeten beslist worden door een agentschap dat onafhankelijk is van politieke partijen.<br>8. De provincies moeten worden afgeschaft.<br>9. Experten moeten de belangrijkste politieke beslissingen nemen in plaats van politici.	3. Le nombre de ministres et de parlementaires en Belgique devrait être divisé par deux.<br>4. Le salaire des élus devrait être limité à maximum 2500 euros brut par mois.<br>5. Le cumul des mandats de bourgmestre et de député devrait être interdit.<br>6. Les ministres devraient être désignés directement par les électeurs.<br>7. Toutes les nominations dans les intercommunales, les entreprises publiques et l'administration devraient être faites par une agence indépendante des partis politiques.<br>8. Les provinces devraient être supprimées.<br>9. Des experts devraient prendre les principales décisions politiques à la place des hommes et femmes politiques.		
w4_Sup_Cit_Exp_1 To w4_Sup_Cit_Exp_6	**For each one, can you, on a scale of 0 to 10, tell us how much you agree with the following**	**Kan u voor elk van de volgende stellingen aangeven, op een schaal van 0 tot 10, in welke mate u het eens of oneens bent met de**	**Pour chacune, pouvez-vous, sur une échelle allant de 0 à 10, nous dire à quel point vous êtes d'accord avec l'affirmation suivante ? [0-10**	0	10

	**statement?** **[0-10 scale: 0 = Totally disagree; 10 = Totally agree]**<br>1. Most citizens have all the skills required to make political decisions.<br>2. Most experts have all the skills needed to make political decisions.<br>3. Most citizens are honest.<br>4. Most experts are honest.<br>5. Most citizens are able to understand the needs of people like me.<br>6. Most experts are able to understand the needs of people like me.	**stelling?** **[0-10 scale: 0 = Helemaal oneens; 10 = Helemaal eens]**<br>1. De meeste burgers zijn competent om politieke beslissingen te nemen.<br>2. De meeste experts zijn competent om politieke beslissingen te nemen.<br>3. De meeste burgers zijn eerlijk.<br>4. De meeste experts zijn eerlijk.<br>5. De meeste burgers zijn in staat om de noden van personen zoals ik te begrijpen.<br>6. De meeste experts zijn in staat om de noden van personen zoals ik te begrijpen.	**scale : 0 = Pas du tout d'accord ; 10 = Tout à fait d'accord]**<br>1. La plupart des citoyens ont toutes les compétences requises pour prendre des décisions politiques.<br>2. La plupart des experts ont toutes les compétences requises pour prendre des décisions politiques.<br>3. La plupart des citoyens sont honnêtes.<br>4. La plupart des experts sont honnêtes.<br>5. La plupart des citoyens sont capables de comprendre les besoins de personnes comme moi.<br>6. La plupart des experts sont capables de comprendre les besoins de personnes comme moi.		
w4_Exp_Policy_Outcom_1<br>to<br>w4_Exp_Policy_Outcom_5	**According to you, how likely is it that you will agree with the decisions taken on the following levels of government in the next 5 years?** **[0-10 scale: 0 = Very unlikely; 10 = Very likely; 99 = I do not know]**<br>1. At the european level<br>2. At the Belgian level<br>3. At the flemish level<br>4. At the Brussels level	**Hoe waarschijnlijk is het volgens u dat beslissingen zullen worden genomen waarmee u het eens bent op de volgende regeringsniveaus in de komende vijf jaar?** **[0-10 scale: 0 = Zeer onwaarschijnlijk; 10 = Zeer waarschijnlijk; 99 = Weet niet]**<br>1. Op Europees niveau<br>2. Op Belgisch niveau<br>3. Op Vlaams niveau<br>4. Op Brussels niveau	**Selon vous, quelle est la probabilité que vous soyez d'accord avec les décisions des niveaux de gouvernement suivants au cours des cinq prochaines années?** **[0-10 scale: 0 = Peu probable; 10 = Très probable; 99 = Je ne sais pas]**<br>1. Au niveau européen<br>2. Au niveau belge<br>3. Au niveau flamand<br>4. Au niveau bruxellois	0	10

	**5.** At the walloon level	5. Op Waals niveau	5. Au niveau wallon		
w4_Burgers_politici_1<br>To<br>w4_Burgers_politici_6	**To what extent are you in favour or against the following statement? "Important political decisions should more often be taken by ordinary citizens rather than by elected politicians". [0-10 scale: 0= Totally against; 10 = Totally in favour]**<br>1. At the european level<br>2. At the belgian level<br>3. At the flemish level<br>4. At the Brussels level<br>5. At the walloon level<br>**6.** At the local level	**In welke mate bent u voor of tegen het volgende? "Belangrijke politieke beslissingen zouden vaker gemaakt moeten worden door gewone burgers en niet door verkozen politici." [0-10 scale: 0 = Sterk tegen; 10 = Sterk voor]**<br>1. Op Europees niveau<br>2. Op Belgisch niveau<br>3. Op Vlaams niveau<br>4. Op Brussels niveau<br>5. Op Waals niveau<br>6. Op lokaal niveau	**Dans quelle mesure êtes-vous en faveur ou contre la proposition suivante? "Les décisions politiques importantes devraient être prises plus souvent par les citoyens ordinaires et non par les hommes et femmes politiques élus." [0-10 scale: 0 = Fortement contre; 10 = Fortement pour]**<br>1. Au niveau européen<br>2. Au niveau belge<br>3. Au niveau flamand<br>4. Au niveau bruxellois<br>5. Au niveau wallon<br>6. Au niveau local	0	10
w4_advis_ref	**Are you, in general, for or against consultative referendums on important national issues?** Citizens have the right to vote on a specific proposal. Parliament receives the voters' opinion but is not obliged to follow it. **[0-10 scale: 0 = Totally against; 10 = Totally in favour; 99 = Don't know]**	**Bent u, over het algemeen, voor of tegen adviserende referenda over belangrijke nationale thema's?** Burgers hebben dan het recht om een stem uit te brengen over een specifiek voorstel. Het parlement ontvangt het advies van de kiezers, maar is niet verplicht om dit te volgen. **[0-10 scale: 0 = Sterk tegen; 10 = Sterk voor; 99 = Weet niet]**	**Êtes-vous, en général, pour ou contre les référendums consultatifs sur les thèmes nationaux importants ?** Les citoyens ont le droit de voter sur une proposition spécifique. Le Parlement reçoit l'avis des électeurs mais sans être obligé de le suivre. **[0-10 scale : 0 = Totalement contre ; 10 = Totalement pour ; 99 = Je ne sais pas]**	0	10

w4_Bindend_ref	**Are you, in general, for or against binding referendums on important national issues?** Citizens have the right to vote on a specific proposal. Parliament is obliged to follow the opinion of the people. **[0-10 scale: 0 = Totally against; 10 = Totally in favour; 99 = Don't know]**	**Bent u, over het algemeen, voor of tegen bindende referenda over belangrijke nationale thema's?** Burgers hebben dan het recht om een stem uit te brengen over een specifiek voorstel. Het parlement is verplicht het advies van de kiezers te volgen. **[0-10 scale: 0 = Sterk tegen; 10 = Sterk voor; 99 = Weet niet]**	**Êtes-vous, en général, pour ou contre les référendums contraignants sur les thèmes nationaux importants ?** Les citoyens ont le droit de voter sur une proposition spécifique. Le Parlement est obligé de suivre l'avis de la population. **[0-10 scale : 0 = Fortement contre ; 10 = Fortement pour ; 99 = Je ne sais pas]**	0	10
w4_Advis_burgerforum	**Are you, in general, for or against the organisation of citizens' consultative forums on important national issues?** A citizens' forum is an assembly of about 30-50 citizens, drawn by lot, who meet and discuss a specific topic in order to produce a recommendation which is then forwarded to Parliament. **[0-10 scale: 0 = Totally against; 10 = Totally in favour; 99 = Don't know]**	**Bent u, over het algemeen, voor of tegen de organisatie van adviserende burgerfora over belangrijke nationale thema's?** Een burgerforum bestaat uit 30 tot 50 burgers, die op basis van toeval geselecteerd zijn. Ze komen samen en discussiëren over een bepaald thema om zo tot een advies aan het parlement te komen. **[0-10 scale: 0 = Sterk tegen; 10 = Sterk voor; 99 = Weet niet]**	**Êtes-vous, en général, pour ou contre l'organisation de forums consultatifs de citoyens sur les thèmes nationaux importants ?** Un forum citoyen est une assemblée composée d'environ 30 à 50 citoyens, tirés au sort, qui se rencontrent et qui discutent d'un sujet spécifique afin de produire une recommandation transmise ensuite au Parlement. **[0-10 scale : 0 = Fortement contre ; 10 = Fortement pour ; 99 = Je ne sais pas]**	0	10
w4_burgerbegroting	**Are you, in general, for or against participatory budgets at the national level?** A participatory budget means that citizens decide on a part of the Belgian state budget. The citizens involved meet and discuss how they want the money to be spent to support different concrete projects. **[0-10**	**Bent u, over het algemeen, voor of tegen burgerbegrotingen op nationaal niveau?** Een burgerbegroting betekent dat burgers beslissen over een deel van de Belgische begroting. De betrokken burgers komen samen en discussiëren over hoe ze het geld wensen te verdelen overheen een	**Êtes-vous, en général, pour ou contre les budgets participatifs au niveau national ?** Un budget participatif signifie que des citoyens décident d'une partie du budget de l'Etat belge. Les citoyens impliqués se réunissent et discutent de la façon dont ils souhaitent que l'argent soit dépensé pour soutenir	0	10

	scale: 0 = Totally against; 10 = Totally in favour; 99 = Don't know]	aantal concrete projecten. [0-10 scale: 0 = Sterk tegen; 10 = Sterk voor; 99 = Weet niet]	différents projets concrets. [0-10 scale : 0 = Fortement contre ; 10 = Fortement pour ; 99 = Je ne sais pas]		
w4_VAA_statements_1<br>To<br>w4_VAA_statements_18	**Below you will find a series of proposals. Can you indicate the extent to which you agree or disagree with the following proposals?** [1-4 scale: 1 = Totally Disagree; 2 = Disagree; 3 = Agree; 4 = Totally Agree]<br>1. Hosting migrants in transit should be a punishable offence.<br>2. Situational testing must be introduced to detect discrimination in employment.<br>3. In order to be able to obtain Belgian nationality, one must first have passed an examination on European values.<br>4. If the asylum applications of families with children have been rejected, these families may be placed in detention pending repatriation.<br>5. By 2024, company cars running on petrol or diesel must be banned.<br>6. VAT on electricity must be reduced from 21% to 6%.	**Hieronder vindt u enkele beleidsvoorstellen. Kan u voor elk van volgende beleidsvoorstellen aangeven in welke mate u het ermee eens of oneens bent?** [1-4 scale: 1 = Helemaal oneens; 2 = Eerder oneens; 3 = Eerder eens; 4 = Helemaal eens]<br>1. Transmigranten onderdak bieden moet strafbaar zijn.<br>2. De overheid moet praktijktesten doen om te controleren op discriminatie bij aanwervingen.<br>3. Nieuwkomers moeten eerst slagen voor een examen over de Europese waarden voor ze de Belgische nationaliteit kunnen verwerven.<br>4. Als de asielaanvraag van gezinnen met kinderen is afgewezen, mogen ze in afwachting van hun terugkeer worden opgesloten.<br>5. Bedrijfswagens die op benzine of diesel rijden	**Ci-dessous vous trouverez une série de propositions. Pouvez-vous indiquer dans quelle mesure vous êtes d'accord ou pas d'accord avec les propositions suivantes ?** [1-4 scale : 1 = Pas du tout d'accord ; 2 = Pas d'accord ; 3 = D'accord ; 4 = Tout à fait d'accord]<br>1. Héberger des migrants en transit doit être un délit punissable.<br>2. Il faut mettre en place des tests de situation pour détecter la discrimination à l'embauche.<br>3. Pour pouvoir obtenir la nationalité belge, il faut d'abord avoir réussi un examen sur les valeurs européennes.<br>4. Si la demande d'asile de familles avec enfants a été rejetée, ces familles peuvent être placées en détention en attendant leur rapatriement.<br>5. D'ici 2024, les voitures de société qui roulent à	1	4

	7. Airline tickets should be taxed to make them more expensive.<br>8. Nuclear power plants must remain operational after 2025.<br>9. You can't drive while drinking alcohol.<br>10. Abortion must also be allowed beyond 12 weeks of pregnancy.<br>11. It must no longer be possible to donate sperm anonymously.<br>12. Big money should be taxed more.<br>13. Wages should no longer be automatically indexed.<br>14. The fingerprints of all citizens should be stored in a central database.<br>15. Stores must be able to choose when they do their sales.<br>16. A retirement pension of at least €1500 per month should be introduced.<br>17. The government must have as many men as women.<br>18. It must be possible to leave important political decisions to the citizens by means of a referendum.	moeten voor 2024 verboden worden.<br>6. De btw op elektriciteit moet verlaagd worden van 21 naar 6%.<br>7. Vliegen moet duurder worden door de tickets te belasten.<br>8. Ook na 2025 moeten we kerncentrales openhouden.<br>9. Als je rijdt mag je helemaal geen alcohol gedronken hebben.<br>10. Ook na 12 weken zwangerschap moet abortus nog toegestaan worden.<br>11. Spermadonatie mag niet langer anoniem gebeuren.<br>12. Grote vermogens moeten meer worden belast.<br>13. De lonen mogen niet meer automatisch aan de prijsstijgingen worden aangepast (index).<br>14. Vingerafdrukken van alle burgers moeten in een centrale databank worden bijgehouden.<br>15. Winkels mogen zelf kiezen wanneer ze solden doen.	l'essence ou au diesel doivent être interdites.<br>6. La TVA sur l'électricité doit être réduite de 21 à 6%.<br>7. Il faut taxer les billets d'avion pour qu'ils soient plus chers.<br>8. Les centrales nucléaires doivent rester opérationnelles après 2025.<br>9. On ne peut pas conduire en ayant bu de l'alcool.<br>10. L'avortement doit aussi être autorisé au-delà de 12 semaines de grossesse.<br>11. Le don de sperme ne doit plus pouvoir être anonyme.<br>12. Les grandes fortunes doivent être plus taxées.<br>13. Les salaires ne doivent plus être automatiquement indexés.<br>14. Les empreintes digitales de tous les citoyens doivent être conservées dans une base de données centrale.<br>15. Les magasins doivent pouvoir choisir quand ils font leurs soldes.<br>16. Il faut instaurer une pension de retraite de 1500€ minimum par mois		

		16. Er moet een minimumpensioen komen van minstens €1500 euro per maand.<br>17. Er moeten in de regering evenveel mannen als vrouwen zitten.<br>18. Belangrijke politieke beslissingen moeten via een referendum aan burgers kunnen worden overgelaten.	17. Le gouvernement doit compter autant d'hommes que de femmes.<br>18. Les décisions politiques importantes doivent pouvoir être laissées aux citoyens par le biais d'un référendum.		
[If region = Flanders]<br><br>w4_Perc_PartyPos_FL_1_1 to w4_Perc_PartyPos_FL_18_7	**For each policy proposal, can you indicate the parties that you think agree with the proposal?** You can select more than one party. **[1 = CD&V; 2 = Groen; 3 = N-VA; 4 = Open VLD; 5 = PVDA; 6 = sp.a; 7 = Vlaams Belang]**<br>1. Hosting migrants in transit should be a punishable offence.<br>2. Situational testing must be introduced to detect discrimination in employment.<br>3. In order to be able to obtain Belgian nationality, one must first have passed an examination on European values.	**Kunt u voor elk beleidsvoorstel de partijen aanduiden waarvan u denkt dat ze het met het voorstel eens zijn?** U kunt voor elk voorstel meerdere partijen aanklikken. **[1 = CD&V; 2 = Groen; 3 = N-VA; 4 = Open VLD; 5 = PVDA; 6 = sp.a; 7 = Vlaams Belang]**<br>1. Transmigranten onderdak bieden moet strafbaar zijn.<br>2. De overheid moet praktijktesten doen om te controleren op discriminatie bij aanwervingen.<br>3. Nieuwkomers moeten eerst slagen voor een examen over de Europese waarden voor ze de	**Pour chaque proposition de politique, pouvez-vous indiquer les partis qui, selon vous, sont en accord avec la proposition ?** Vous pouvez sélectionner plusieurs partis. **[1 = CD&V; 2 = Groen; 3 = N-VA; 4 = Open VLD; 5 = PVDA; 6 = sp.a; 7 = Vlaams Belang]**<br>1. Héberger des migrants en transit doit être un délit punissable.<br>2. Il faut mettre en place des tests de situation pour détecter la discrimination à l'embauche.<br>3. Pour pouvoir obtenir la nationalité belge, il faut d'abord avoir réussi un examen sur les valeurs européennes.	1	7

	4. If the asylum applications of families with children have been rejected, these families may be placed in detention pending repatriation.<br>5. By 2024, company cars running on petrol or diesel must be banned.<br>6. VAT on electricity must be reduced from 21% to 6%.<br>7. Airline tickets should be taxed to make them more expensive.<br>8. Nuclear power plants must remain operational after 2025.<br>9. You can't drive while drinking alcohol.<br>10. Abortion must also be allowed beyond 12 weeks of pregnancy.<br>11. It must no longer be possible to donate sperm anonymously.<br>12. Big money should be taxed more.<br>13. Wages should no longer be automatically indexed.<br>14. The fingerprints of all citizens should be stored in a central database.<br>15. Stores must be able to choose when they do their sales.	Belgische nationaliteit kunnen verwerven.<br>4. Als de asielaanvraag van gezinnen met kinderen is afgewezen, mogen ze in afwachting van hun terugkeer worden opgesloten.<br>5. Bedrijfswagens die op benzine of diesel rijden moeten voor 2024 verboden worden.<br>6. De btw op elektriciteit moet verlaagd worden van 21 naar 6%.<br>7. Vliegen moet duurder worden door de tickets te belasten.<br>8. Ook na 2025 moeten we kerncentrales openhouden.<br>9. Als je rijdt mag je helemaal geen alcohol gedronken hebben.<br>10. Ook na 12 weken zwangerschap moet abortus nog toegestaan worden.<br>11. Spermadonatie mag niet langer anoniem gebeuren.<br>12. Grote vermogens moeten meer worden belast.	4. Si la demande d'asile de familles avec enfants a été rejetée, ces familles peuvent être placées en détention en attendant leur rapatriement.<br>5. D'ici 2024, les voitures de société qui roulent à l'essence ou au diesel doivent être interdites.<br>6. La TVA sur l'électricité doit être réduite de 21 à 6%.<br>7. Il faut taxer les billets d'avion pour qu'ils soient plus chers.<br>8. Les centrales nucléaires doivent rester opérationnelles après 2025.<br>9. On ne peut pas conduire en ayant bu de l'alcool.<br>10. L'avortement doit aussi être autorisé au-delà de 12 semaines de grossesse.<br>11. Le don de sperme ne doit plus pouvoir être anonyme.<br>12. Les grandes fortunes doivent être plus taxées.<br>13. Les salaires ne doivent plus être automatiquement indexés.<br>14. Les empreintes digitales de tous les citoyens doivent		

	16. A retirement pension of at least €1500 per month should be introduced.<br>17. The government must have as many men as women.<br>18. It must be possible to leave important political decisions to the citizens by means of a referendum.	13. De lonen mogen niet meer automatisch aan de prijsstijgingen worden aangepast (index).<br>14. Vingerafdrukken van alle burgers moeten in een centrale databank worden bijgehouden.<br>15. Winkels mogen zelf kiezen wanneer ze solden doen.<br>16. Er moet een minimumpensioen komen van minstens €1500 euro per maand.<br>17. Er moeten in de regering evenveel mannen als vrouwen zitten.<br>18. Belangrijke politieke beslissingen moeten via een referendum aan burgers kunnen worden overgelaten.	être conservées dans une base de données centrale.<br>15. Les magasins doivent pouvoir choisir quand ils font leurs soldes.<br>16. Il faut instaurer une pension de retraite de 1500€ minimum par mois<br>17. Le gouvernement doit compter autant d'hommes que de femmes.<br>18. Les décisions politiques importantes doivent pouvoir être laissées aux citoyens par le biais d'un référendum.		
[If region = Wallonia]<br>w4_Perc_PartyPos_WAL_1_1<br>To<br>w4_Perc_PartyPos_WAL_18_7	**For each policy proposal, can you indicate the parties that you think agree with the proposal?** You can select more than one party**.** **[1 =cdH; 2 =DéFI; 3 = Ecolo; 4 = MR; 6 = PS; 7 = PTB]**<br>1. Hosting migrants in transit should be a punishable offence.<br>2. Situational testing must be introduced to detect	**Kunt u voor elk beleidsvoorstel de partijen aanduiden waarvan u denkt dat ze het met het voorstel eens zijn?** U kunt voor elk voorstel meerdere partijen aanklikken. **[1 =cdH; 2 =DéFI; 3 = Ecolo; 4 = MR; 6 = PS; 7 = PTB]**<br>1. Transmigranten onderdak bieden moet strafbaar zijn.<br>2. De overheid moet praktijktesten doen om te	**Pour chaque proposition de politique, pouvez-vous indiquer les partis qui, selon vous, sont en accord avec la proposition ?** Vous pouvez sélectionner plusieurs partis. **[1 =cdH; 2 =DéFI; 3 = Ecolo; 4 = MR; 6 = PS; 7 = PTB]**<br>1. Héberger des migrants en transit doit être un délit punissable.	1	7

	discrimination in employment.<br>3. In order to be able to obtain Belgian nationality, one must first have passed an examination on European values.<br>4. If the asylum applications of families with children have been rejected, these families may be placed in detention pending repatriation.<br>5. By 2024, company cars running on petrol or diesel must be banned.<br>6. VAT on electricity must be reduced from 21% to 6%.<br>7. Airline tickets should be taxed to make them more expensive.<br>8. Nuclear power plants must remain operational after 2025.<br>9. You can't drive while drinking alcohol.<br>10. Abortion must also be allowed beyond 12 weeks of pregnancy.<br>11. It must no longer be possible to donate sperm anonymously.	controleren op discriminatie bij aanwervingen.<br>3. Nieuwkomers moeten eerst slagen voor een examen over de Europese waarden voor ze de Belgische nationaliteit kunnen verwerven.<br>4. Als de asielaanvraag van gezinnen met kinderen is afgewezen, mogen ze in afwachting van hun terugkeer worden opgesloten.<br>5. Bedrijfswagens die op benzine of diesel rijden moeten voor 2024 verboden worden.<br>6. De btw op elektriciteit moet verlaagd worden van 21 naar 6%.<br>7. Vliegen moet duurder worden door de tickets te belasten.<br>8. Ook na 2025 moeten we kerncentrales openhouden.<br>9. Als je rijdt mag je helemaal geen alcohol gedronken hebben.<br>10. Ook na 12 weken zwangerschap moet	2. Il faut mettre en place des tests de situation pour détecter la discrimination à l'embauche.<br>3. Pour pouvoir obtenir la nationalité belge, il faut d'abord avoir réussi un examen sur les valeurs européennes.<br>4. Si la demande d'asile de familles avec enfants a été rejetée, ces familles peuvent être placées en détention en attendant leur rapatriement.<br>5. D'ici 2024, les voitures de société qui roulent à l'essence ou au diesel doivent être interdites.<br>6. La TVA sur l'électricité doit être réduite de 21 à 6%.<br>7. Il faut taxer les billets d'avion pour qu'ils soient plus chers.<br>8. Les centrales nucléaires doivent rester opérationnelles après 2025.<br>9. On ne peut pas conduire en ayant bu de l'alcool.<br>10. L'avortement doit aussi être autorisé au-delà de 12 semaines de grossesse.		

	12. Big money should be taxed more.<br>13. Wages should no longer be automatically indexed.<br>14. The fingerprints of all citizens should be stored in a central database.<br>15. Stores must be able to choose when they do their sales.<br>16. A retirement pension of at least €1500 per month should be introduced.<br>17. The government must have as many men as women.<br>18. It must be possible to leave important political decisions to the citizens by means of a referendum.	abortus nog toegestaan worden.<br>11. Spermadonatie mag niet langer anoniem gebeuren.<br>12. Grote vermogens moeten meer worden belast.<br>13. De lonen mogen niet meer automatisch aan de prijsstijgingen worden aangepast (index).<br>14. Vingerafdrukken van alle burgers moeten in een centrale databank worden bijgehouden.<br>15. Winkels mogen zelf kiezen wanneer ze solden doen.<br>16. Er moet een minimumpensioen komen van minstens €1500 euro per maand.<br>17. Er moeten in de regering evenveel mannen als vrouwen zitten.<br>18. Belangrijke politieke beslissingen moeten via een referendum aan burgers kunnen worden overgelaten.	11. Le don de sperme ne doit plus pouvoir être anonyme.<br>12. Les grandes fortunes doivent être plus taxées.<br>13. Les salaires ne doivent plus être automatiquement indexés.<br>14. Les empreintes digitales de tous les citoyens doivent être conservées dans une base de données centrale.<br>15. Les magasins doivent pouvoir choisir quand ils font leurs soldes.<br>16. Il faut instaurer une pension de retraite de 1500€ minimum par mois<br>17. Le gouvernement doit compter autant d'hommes que de femmes.<br>18. Les décisions politiques importantes doivent pouvoir être laissées aux citoyens par le biais d'un référendum		

END OF SURVEY
